# Development and validation of a novel circular RNA as an independent prognostic factor in acute myeloid leukemia

**DOI:** 10.1186/s12916-020-01898-y

**Published:** 2021-02-01

**Authors:** Jinghan Wang, Jiajia Pan, Shujuan Huang, Fenglin Li, Jiansong Huang, Xia Li, Qing Ling, Wenle Ye, Yungui Wang, Wenjuan Yu, Jie Jin

**Affiliations:** 1 Department of Hematology, The First Affiliated Hospital, Zhejiang University College of Medicine, No. 79 Qingchun Road, Hangzhou, 310003 Zhejiang People’s Republic of China; 2 Key Laboratory of Hematologic Malignancies, Diagnosis and Treatment, Hangzhou, Zhejiang People’s Republic of China; 3 Institute of Hematology, Zhejiang University, Hangzhou, People’s Republic of China; 4 Zhejiang University Cancer Center Zhejiang University , Zhejiang Hangzhou, People’s Republic of China

**Keywords:** Acute myeloid leukemia, Prognosis, Circular RNAs, RNA sequencing

## Abstract

**Background:**

Although there are many clinical and molecular biomarkers in acute myeloid leukemia (AML), the novel and reliable biomarkers are still required to predict the overall survival at the time of disease diagnosis.

**Methods:**

In order to identify independent predictors, we firstly selected 60 cytogenetically normal AML (CN-AML) patients using the propensity score analysis to balance the confounders and performed circular RNA (circRNA) sequencing. Next, one outcome related to circRNA was selected and validated in the independent cohort of 218 CN-AML patients. We then constructed circRNA-miRNA-mRNA regulated network and performed cellular metabolomic analysis to decipher the underlying biological insights.

**Results:**

We identified 308 circRNAs as independent candidate predictors of overall survival. Hsa_circ_0075451 expression was validated as an independent predictor with a weak predictive ability for overall survival. The regulated network of this circular RNA indicated 84 hub genes that appear to be regulated by 10 miRNAs sponged by hsa_circ_0075451. The regulatory axis of hsa_circ_0075451 -| miR-330-5p/miR-326 -| *PRDM16* was validated by the dual luciferase report assay, fluorescence in situ hybridization, and ShRNA interference assay.

**Conclusions:**

Our data demonstrates that hsa_circ_0075451 expression may independently contribute to the poor prognosis of AML and present a novel therapeutic target.

**Supplementary Information:**

The online version contains supplementary material available at 10.1186/s12916-020-01898-y.

## Background

Acute myeloid leukemia (AML) is a group of hematologic malignancies with diverse genetic features and different clinical outcomes. Despite the high efficacy of intensive chemotherapy for young patients and novel targeted drugs for unfitted elderly patients, many patients still relapse and subsequently die within 2 years [1]. Thus, it is critical for making an alternative treatment decision to identify high-risk patients at the time of diagnosis. Currently, the well-established predictors include clinical parameters such as old age, white blood cell count, lactate dehydrogenase level, and poor performance [2]. Concomitantly, molecular markers are becoming more and more important to stratify patients into different risk subgroups [2, 3]. They include cytogenetic risk subgroups, molecular genetic alterations such as *FLT3*-ITD, *NPM1*, and *CEBPA* mutations [2]. Notably, numerous high-throughput analyses including DNA microarray, RNA sequencing, proteomics, metabolomics, and many others are still under investigation. Thus, the exploration of novel prognostic markers for AML patients has become the main research topic among scientists and doctors.

Recent reports have shown that circular RNAs (circRNAs) have the oncogenic function and prognostic significance in solid tumors such as hepatocellular carcinoma and lung cancer as well as hematologic malignancies including leukemia [4–7]. circRNAs are generated from back-splicing of nucleotide sequence to form a circular closed structure, which are different from linear RNAs [8]. This means they may be more stable than linear RNAs and cannot be degraded by endonucleases. Because of their abundance, stability, and tissue specificity, circRNAs could be potentially used as biomarkers for outcome prediction and therapeutic targets. It was reported that hsa_circ_0004277 and circ-Foxo3 were lowly expressed in AML [9, 10], whereas hsa_circ_0000488 and hsa_circ_0009910 were upregulated and were associated with decreased overall survival rate [11, 12]. Also, an association has been established between circPAN3 and doxorubicin resistance in AML cell lines [13]. Additionally, some of the circRNAs (i.e., hsa_circ_0075001 and CircMYBL2) are linked with gene mutations (e.g., *NPM1* and *FLT3*) and can be used as drug targets [6, 7]. Moreover, some circRNAs are linked to the extramedullary infiltration in leukemia and at the same time correlated to poor survival in AML [14, 15]. In sum, these previous studies show circRNAs linked to the other predictors can also be used as a potential predictor for AML. However, the independent prognostic impact of circRNAs on the context of the well-established markers has not been studied in AML patients.

In this study, the major goal was to search for a circRNA signature independently to predict the patient outcome at the time of diagnosis. We first searched for survival-relevant circRNAs in 60 cytogenetically normal acute myeloid leukemia (CN-AML) patients from the prospective clinical trial using RNA sequencing. We then validated a candidate circRNA in the independent cohort of 218 CN-AML patients. Finally, we identified a regulatory network and a unique metabolic feature to decipher the biologic insights of this circRNA. Herein, we present a reliable and independent prognostic predictor and a novel drug target for AML patients.

## Methods

### Patients and treatments

**Fig. 1:**
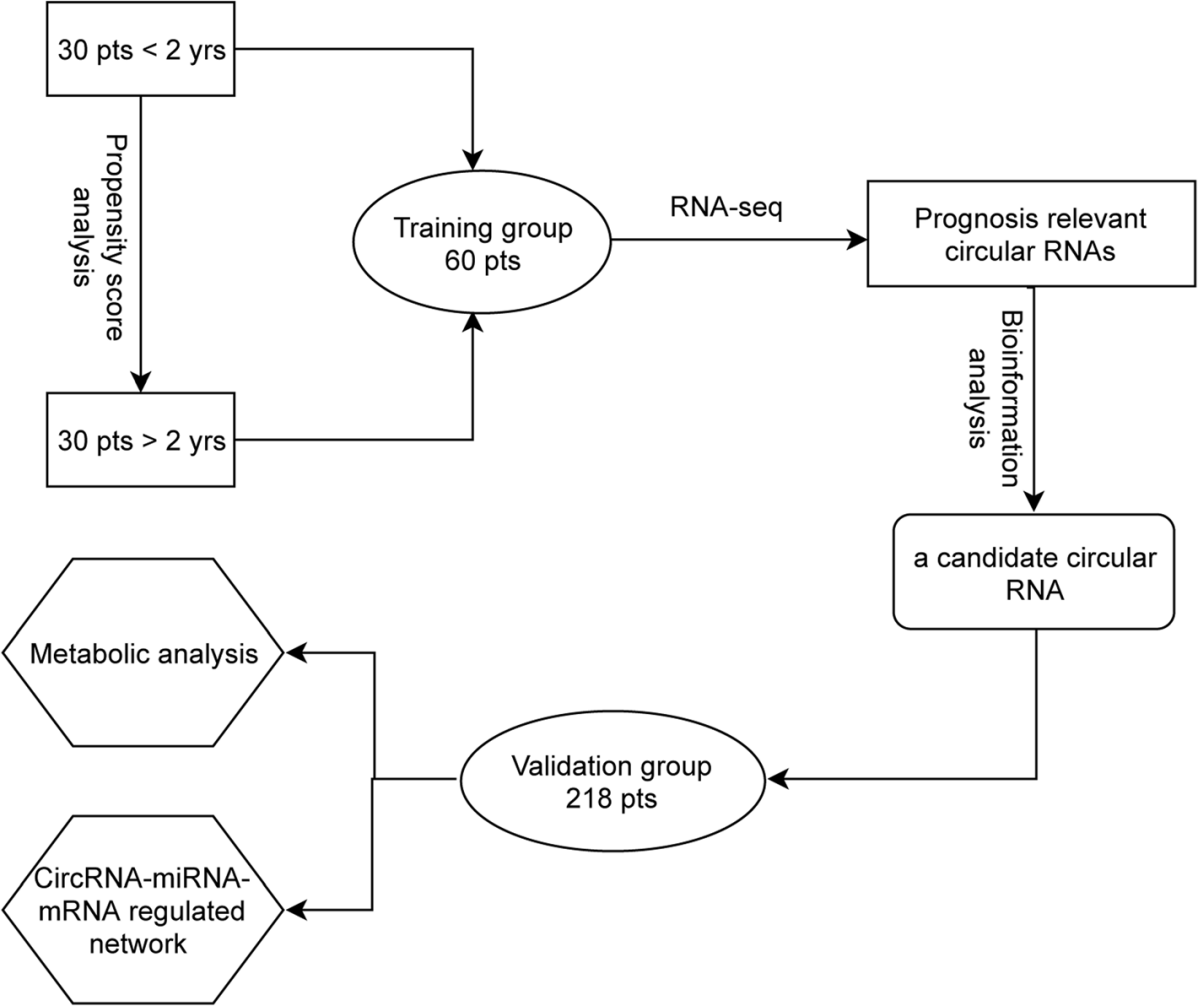
The experimental procedure of the study. We selected 30 patients (pts) who survived less than 2 years (< 2 years) as the unfavorable group. Propensity score analysis was used to select another 30 pts that survived more than 2 years (> 2 years) as the favorable group. In total, 60 patients (60 pts) were assigned to the training group and perform the circRNAs’ expression profile analysis. Here, one candidate circular RNA was identified and validated in the independent cohort of 218 pts. Finally, circRNA-miRNA-mRNA regulatory networks and cellular metabolic analysis were conducted to decipher the biological feature of this circular RNA.

In order to identify an independent predictor for the risk stratification in CN-AML patients, we conducted this study using a discovery and validation design. The experimental procedure is summarized in Fig. 1. In our clinical trial (ChiCTR-IPR-17012643), 75 CN-AML patients’ high-quality bone marrow samples were taken for RNA sequencing. Of these patients, thirty survived less than 2 years and so were assigned to the unfavorable group. In order to exclude the potential confounders, we conducted propensity score analysis to match 30 patients that survived more than 2 years based on age, gender, white blood cell count, and mutations in *FLT3*-ITD, *NPM1*, *CEBPA*, *DNMT3A*, *IDH1*, and *IDH2* genes (Additional file 1:Fig. S1 and Table S1). The circRNA expression profile was analyzed in bone marrow samples from these 60 patients as the training group. The validated group consisted of 218 CN-AML patients who underwent intensive induction therapy. Details of the treatment protocols of the validated group are provided in Additional file 1. The clinical data of these patients was obtained from medical records between January 2014 and July 2018 in the hematology department of our hospital.

### Circular RNA sequencing analysis

Total RNA from the fresh frozen samples was isolated by TRIzol Reagent (Invitrogen Life Technologies). RNA quality was evaluated using a Nanodrop ND-1000 (Thermo Fisher Scientific, Waltham, MA, USA). Transcriptome high-throughput sequencing was done by Cloud-Seq Biotech (Shanghai, China). Briefly, total RNA was used for removing the rRNAs using Ribo-Zero rRNA Removal Kits (Illumina, USA) following the manufacturer’s instructions. RNA libraries were constructed by using rRNA-depleted RNAs with TruSeq Stranded Total RNA Library Prep Kit (Illumina, USA) according to the manufacturer’s instructions. Libraries were controlled for quality and quantified using the BioAnalyzer 2100 system (Agilent Technologies, USA). Paired-end reads were harvested from Illumina HiSeq 4000 sequencer and were quality controlled by Q30. Low-quality reads and 3′ adaptor trimming were removed by the “cutadapt” software (v1.9.3). The high-quality trimmed reads were used in circRNA analysis.

### Cytogenetic and gene mutation analysis

Bone marrow (BM) blasts were collected at the time of diagnosis. To be considered cytogenetically normal, at least 20 metaphase blasts had to be evaluated by standard banding cytogenetic analysis. Cytogenetic and molecular analyses were carried out centrally by our molecular laboratories. Mutation studies of *FLT3-*ITD, *NPM1*, *CEBPA*, *DNMT3A*, *IDH1*, and *IDH2* were performed as previously described [16].

### Gene expression arrays and cellular metabolic profiling

Fourteen BM samples of CN-AML patients were used to assess the mRNA expression profile. Additional 14 BM samples with high-quality blasts were used to perform the cellular metabolic profiling research. Details of the methods are described in Additional file 1.

### Quantitative reverse transcriptase PCR for patients’ samples

Total RNA was isolated using RNAiso plus (TaKaRa). Five hundred nanograms of RNA was used to synthesize cDNA using the PrimeScriptTM RT reagent kit (TaKaRa) according to the manufacturer’s instructions. Quantitative RT-PCR was performed using a 2× Taq PCR mix. The primers are listed as follows: hsa_circ_0075451 F: 5′-ACTGACAGTACCTGCCTTGTG-3′, hsa_circ_0075451 R: 5′-AAATGAACTGGACCGCCGTA-3′; beta-actin F: 5′-CATGTACGTTGCTATCCAGGC-3′, beta-actin R: 5′-CTCCTTAATGTCACGCACGAT-3′.

### Quantitative reverse transcriptase PCR for leukemia cell lines

Total RNA was isolated using RNAiso plus (TaKaRa, Japan) according to the manufacturer’s instructions. For RNase R treatment, 2 mg of total RNA of THP-1 was incubated 20 min at 37 °C with or without 3 U/μg RNase R (Epicentre Technologies, USA). Reverse transcription of mRNA and miRNA was conducted using random primers system (TaKaRa, Japan) and stem-loop primers in the GeneCopoeia system (MD, USA), respectively. gDNA was extracted by QuickGene DNA whole blood kit S (KURBO, Japan). PCR was performed using PrimerSTAR HS DNA Polymerase (TaKaRa, Japan) system. After PCR amplification, 10 μl amplified product was mixed with loading buffer (TaKaRa) and ran on 1% agarose gel with gel red. The amplified product was visualized under UV. Quantitative RT-PCR was performed using a 2× Taq PCR mix (TaKaRa). The All-in-One miRNA qRT-PCR Detection Kit (GeneCopoeia) was used for miRNA RT-PCR. The primers are listed in Additional file 1: Table S2.

### Luciferase reporter assay

Sense and antisense sequences of hsa_circ_0075451 or 3′-UTR of *PRDM16* were synthesized, annealed, and inserted into the pmirGLO reporter plasmid. These oligonucleotides contain the wild-type or mutated binding sites of miR-515-5p, miR-873, miR-766-3p, miR-940, miR-661, miR-492, miR-330-5p, miR-326, miR-512-5p, and miR-338-3p genes. The sequences were listed in Additional file 1:Table S3. The insertion was confirmed to be corrective by Sanger sequencing (Additional file 1: Fig. S2). For the luciferase reporter assays, HEK293 cells were cultured in 24-well plates, and each well was transfected with 100 ng pmirGLO plasmid and equal amounts (50 nM) of mimic-miRs/miRs or mimic-miR-control using Lipofectamine 3000 (Invitrogen). After 48 h, the cells were assayed using the dual-luciferase reporter assay system (Promega, WI, USA).

### Fluorescence in situ hybridization and ShRNA interference assay

The detailed methods for fluorescence in situ hybridization and ShRNA interference assay were seen in the Additional file 1.

### Statistical analysis

Patient characteristics were summarized using descriptive statistics, which included frequency counts, median, and interquartile range. Categorical variables were compared using Fisher’s exact test, and continuous variables were analyzed using a non-parameter *t* test. The main objective of this study was to evaluate the prognostic impacts of one selected circRNA on the overall survival (OS) of AML patients. OS was defined as the time from the date of diagnosis until death due to any cause or the last follow-up. The cutoff value of hsa_circ_0075451 was determined by Cutoff Finder (http://molpath.charite.de/cutoff/). Univariate and multivariate analyses with Cox proportional hazards models were performed to assess significant predictors. The proportional hazards assumption was checked for each variable before fitting Cox models. We estimated the sample size for the validated group based on the beta value (0.3) of hsa_circ_0075451 in the Cox regression of the training group. A total of 200 patients achieved a Cox regression coefficient of 0.30 at a 0.05 significance level with 85% power. Also, we enrolled 18 (less than 10%) cases as lost to follow-up. We searched for candidate circRNAs related to the outcome using the “edgeR.” The high-quality reads were aligned to the reference transcriptome with the “STAR” software, and circRNAs were detected and identified with the “DCC” software. Hierarchical clustering based on the expression levels of these circular RNAs was performed and visualized by heatmap. The top gene analysis was analyzed on the platform (https://toppgene.cchmc.org/prioritization.jsp). Interaction of miRNA and mRNA integrative analysis was performed in silico using the mirtar platform (http://mirtar.mbc.nctu.edu.tw/human/index.php) and multiple databases by the “multiMiR” and “miRNAtap” packages (DOI:10.18129/B9.bioc.miRNAtap) [17]. Hub genes were selected in the STRING database (http://string-db.org). circRNA-miRNA-mRNA network was visualized by the Cytoscape software, and the detailed procedure to construct the network was shown in Additional file 1: Fig. S3 [18]. *t* test was used to establish the difference of cellular metabolite signatures among those with high and low hsa_circ_0075451 expression. The underlying metabolic pathways were enriched in silico using the platform (http://210.46.80.7:8080/MPINet). All statistical analyses were conducted using the R software, version 3.6.1 (www.r-project.org). The two-sided level of significance was set at -value < 0.05.

## Results

### Clinical characteristics of patients in the training and validated group

Leukemia disease recurrence occurs mainly during the first 2 years after post-remission therapy, and patients who survive after this period are generally considered to have a favorable prognosis [1]. In our clinical trial, we selected CN-AML patients who survived less than 2 years (30 cases) and more than 2 years (30 cases) and also balanced with the clinical and molecular predictors by propensity score analysis (Additional file 1: Fig. S1 and Table S1). circRNA sequencing was analyzed in the bone marrow samples from these 60 patients that comprised the training group. We also enrolled 218 patients as the validated group to test the interesting biomarker using the quantitative real-time PCR. The detailed information of patients is summarized in Table 1 and Fig. 1. Patients are older in the validated cohort than in the training cohort, because the patients in the training cohort from the clinical trial are exclusively younger patients aged less than 60 years. No significant differences in other clinical (i.e., sex, WBC, hemoglobin, and platelet count) and molecular (i.e., *FLT3*-ITD, *NPM1*, *CEBPA*, *IDH1*, and *IDH2* mutations) characteristics were observed between patients of the training group and the validated group.

**Table 1:** Characteristics of CN-AML in this study

Variable	Training group	Validated group	*P* value
Number	60	218	
Sex, male, *n* (%)	32 (53.3)	127 (58.3)	0.556
Age, median (range), years	48.00 [37.75, 53.00]	52.50 [39.00, 63.00]	0.001
WBC, median (IQR), × 10^9^/L	19.05 [7.87, 40.23]	14.20 [3.70, 60.02]	0.746
HB, median (IQR), g/L	84.00 [69.00, 97.00]	84.50 [67.00, 103.75]	0.760
PLT, median (IQR), × 10^9^/L	40.00 [27.00, 65.00]	48.00 [25.25, 85.50]	0.225
BM blast, median (IQR), %	63.00 [44.00, 81.75]	68.25 [43.25, 83.38]	0.367
FAB classification, *n* (%)			0.674
M0	5 (8.3)	26 (11.9)	
M1	7 (11.7)	24 (11.0)	
M2	26 (43.3)	103 (47.2)	
M4	4 (6.7)	9 (4.1)	
M5	17 (28.3)	55 (25.2)	
M6	1 (1.7)	1 (0.5)	
Gene mutations, *n* (%)			
*FLT3*-ITD	14 (23.3)	45 (20.6)	0.722
*NPM1*	17 (28.3)	54 (25.8)	0.740
*CEBPA*^DM^	4 (6.7)	34 (16.8)	0.060
*DNMT3A*	8 (13.3)	28 (13.9)	1.000
*IDH1*	12 (20.0)	32 (16.0)	0.556
*IDH2*	11 (18.3)	24 (12.6)	0.288
ENL favorable group	17 (28.3)	59 (27.1)	0.871

European LeukemiaNet (ELN) favorable genotype represents *NPM1* mutant and *FLT3*-ITD negative or double allele *CEBPA* mutations

*WBC* white blood cell count, *HB* hemoglobin, *PLT* platelet count, *BM* bone marrow, *FAB* Franch-American-British, *DM* double allele, *IQR* interquartile range

### Prognosis relevant circular RNAs in the training group

**Fig. 2:**
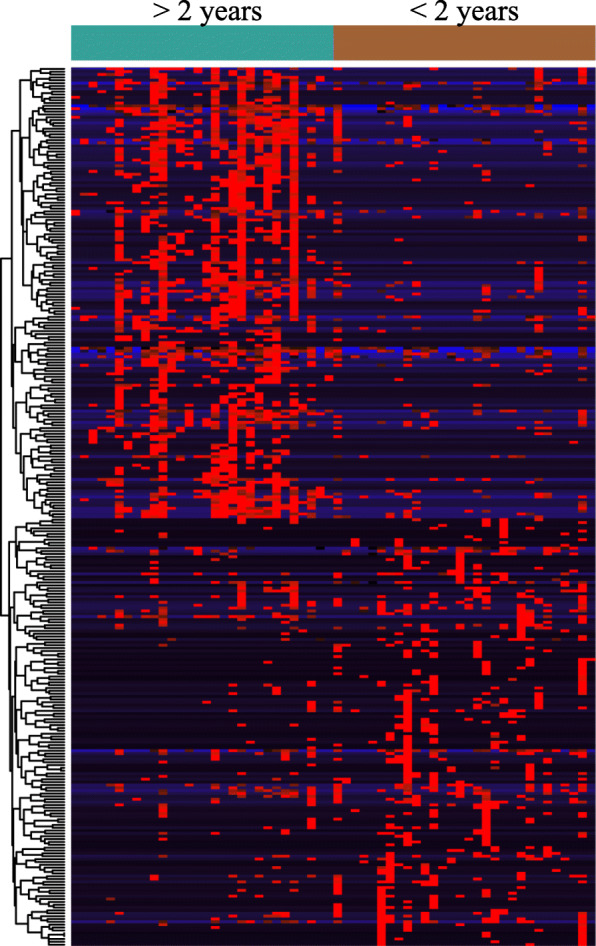
Heatmap visualizing differentially expressed circRNAs in CN-AML patients who survived more than and less than 2 years. Red and black colors represent the high and low expression, respectively. Cyan bar represents patients who survived > 2 years, and brown bar represents patients who survived < 2 years.

We found 2539 differently expressed circRNAs in the training set, including 1257 upregulated and 1282 downregulated circRNAs in the unfavorable group (Additional file 1:Table S4). Next, we used Cox regression analysis to test these differently expressed circRNAs and identified 308 circRNAs closely related to the clinical outcome (Fig. 2 and Additional file 1:Table S5). The molecular functions of these parent genes of circRNAs were analyzed by Gene Ontology (GO) annotation and KEGG/Reactome pathway analyses (Additional file 1:Table S6). This resulted in the identification of 30 GO terms and 20 KEGG/Reactome pathways. Our primary focus was carbon-oxygen lyase activity (GO:0016835), which was clustered by ten parent genes (Additional file 1:Table S7). Notably, hsa_circ_0075451 encoded by GMDS was the most significantly upregulated in patients from the unfavorable group (FDR < 0.001, logFC = 3.28, Additional file 1:Table S7). Therefore, we selected hsa_circ_0075451 for further study.

### Validation of hsa_circ_0075451 as an independent prognostic factor

**Fig. 3:**
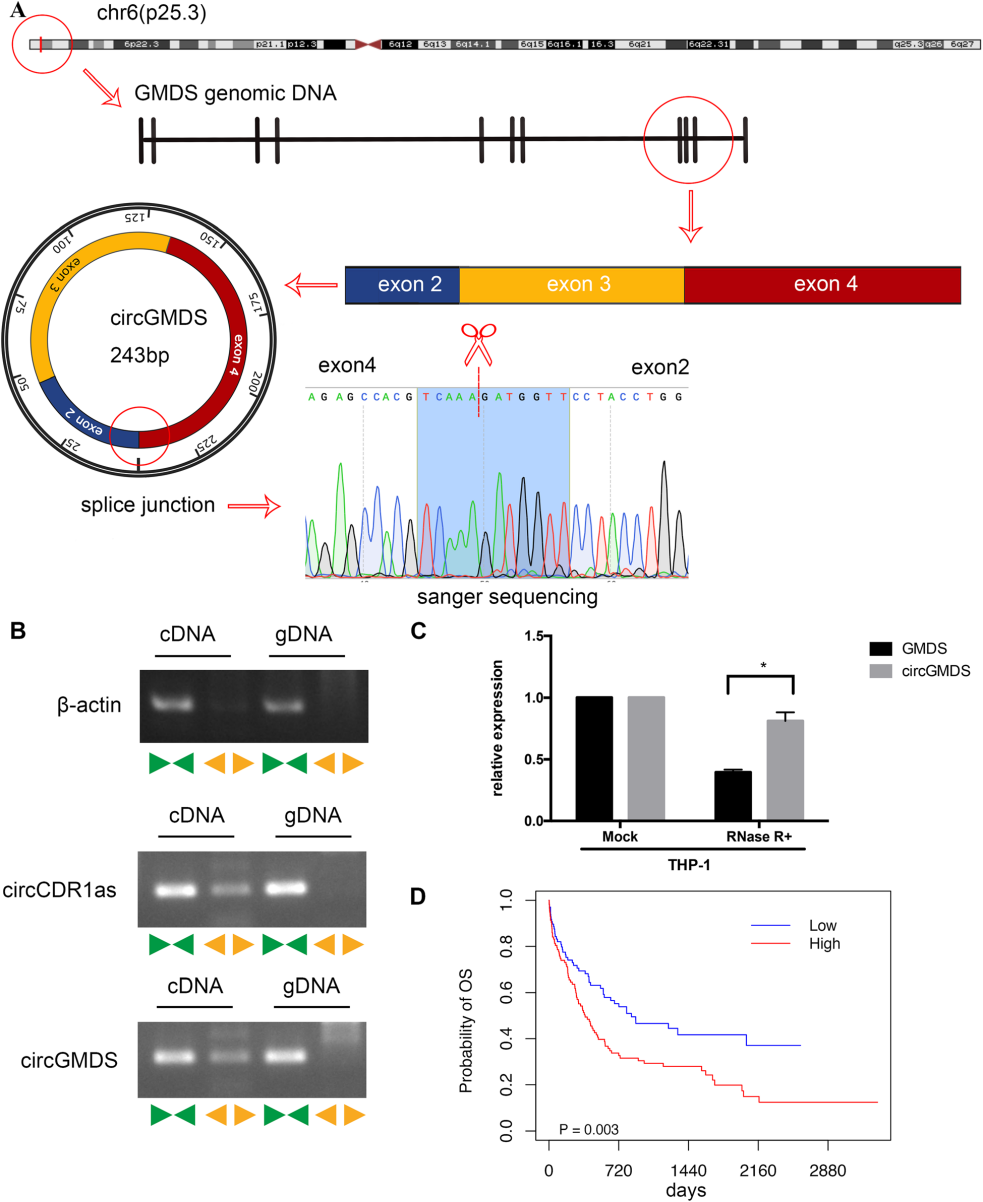
Identification of hsa_circ_0075451(circGMDS). **a** Schematic illustration showing the circularization of *GMDS* exons 2 to 4 forming circGMDS. Red line indicates the special splicing junction of circGMDS. **b** CircGMDS, along with CDR1as and β-actin, was amplified from cDNA or gDNA from THP-1 cells with divergent and convergent primers. Divergent primers amplified circGMDS and CDR1as in cDNA but not genomic DNA (gDNA). β-Actin was used as a negative control. **c** qRT-PCR for the abundance of circGMDS and GMDS mRNA in THP-1 cells treated with RNase R. The amount of circGMDS and GMDS mRNA was normalized to the value measured in the mock treatment. **d** Survival curves of patients with high and low expression of circGMDS in AML patients

hsa_circ_0075451 derives from exon 2, exon 3, and exon 4 of GDP-mannose 4,6-dehydratase (GMDS) and is located on chr6(p25.3) (Fig. 3a-c). We confirmed hsa_circ_0075451 expression in blast cells in the independent cohort of 218 patients. The median of hsa_circ_0075451 transcript levels was 1.14 with the interquartile range from 0.32 to 3.56. To characterize the high expressers, 116 of 218 (53%) AML patients were classified as having a high expression according to the optimal cutoff value estimated by Cutoff Finder. Clinical characteristics of patients with high expression are summarized in Additional file 1:Table S8. Patients with high expression had poorer 3-year survival rate compared to those with low expression (27% vs. 38%, *P* = 0.003, Fig. 3d). There was no statistically significant correlation between hsa_circ_0075451 expression and variables such as age; sex; white blood cell count (WBC); hemoglobin; platelet count; percentage of bone marrow blasts; FAB subtypes; mutations in *FLT3-*ITD, *NPM1*, *CEBPA*, *DNTM3A*, *IDH1*, and *IDH2*; and treatment protocols (Additional file 1:Table S8)*.* In univariable survival analyses, patients with older age, higher white blood cell counts (WBC), and *DNMT3A* mutations were associated with shorter overall survival (Table 2), while those with European LeukemiaNet (ELN) favorable genotype (i.e., mutated *NPM1* without *FLT3-*ITD and *CEBPA* double allele mutations) had a longer OS compared with the counterparts (Table 2). In order to identify the potential confounders or interactive factors, we conducted stratified and interactive analyses. As shown in Additional file 1: Fig. S4, there were no significant interactions among these factors. Even if we take these factors as confounders, hsa_circ_0075451 expression was still an independent prognostic factor in the multivariate analysis after adjusting for age; WBC; European LeukemiaNet (ELN) favorable genotype; mutations in *DNMT3A*, *IDH1*, and *IDH2*; and induction chemotherapy protocols [HR (95%CI), 1.592 (1.067, 2.377); *P* = 0.023; Table 2]. Moreover, we also conducted a landmark analysis by including patients who survived more than 30 days to exclude intense chemotherapy as a cause of early death. We established that the high expression of hsa_circ_0075451 was still independently associated with poor OS [HR (95%CI),1.682 (1.086, 2.607), *P* = 0.02, Additional file 1:Table S9] in the multivariate survival analyses after adjustment for age; WBC; ELN favorable genotype; mutations in *DNMT3A*, *IDH1*, and *IDH2*; and induction chemotherapy protocols.

**Table 2:** Survival analysis of patients in the validated group

Variables	Univariate analysis		Multivariate analysis	
	*P* value	HR (95%CI)	*P* value	HR(95%CI)
**circRNA**	0.003	1.706 (1.195, 2.436)	0.023	1.592 (1.067, 2.377)
**Age**	< 0.001	2.125 (1.484, 3.042)	0.006	1.792 (1.183, 2.714)
**WBC**	0.024	1.003 (1, 1.006)	0.012	1.004 (1.001, 1.007)
**ELN favorable genotype**	0.004	0.532 (0.345, 0.82)	0.089	0.648 (0.393, 1.069)
***DNMT3A***	0.042	1.648 (1.017, 2.668)	0.008	2.031 (1.205, 3.423)
***IDH1***	0.199	1.353 (0.853, 2.148)	0.709	0.887 (0.471, 1.667)
***IDH2***	0.396	0.772 (0.424, 1.404)	0.265	0.706 (0.383, 1.302)
**Treatment**				
**HAA vs. DA**	0.044	0.598 (0.363, 0.987)	0.165	0.652 (0.356, 1.192)
**IA vs. DA**	< 0.001	0.485 (0.325, 0.723)	0.011	0.554 (0.351, 0.875)

CircRNA represents hsa_circ_0075451 high expressers vs. low expressers. European LeukemiaNet (ELN) favorable genotype represents *NPM1* mutant and *FLT3*-ITD negative or double allele *CEBPA* mutations

*WBC* white blood cell count, *HAA* homoharringtonin-based treatment (homoharringtonin 2 mg/m^2^/day for 3 days, cytarabine 75 mg/m^2^ twice daily for 7 days, aclarubicin 12 mg/m^2^ daily for 7 days) regiment, *DA* daunorubicin (45 mg/m^2^ daily for 3 days and cytarabine 100 mg/m^2^ daily for 7 days), *IA* idarubicin (8–10 mg/m^2^ daily for 3 days and cytarabine 100 mg/m^2^ daily for 7 days)

Because the independent predictive value of hsa_circ_0075451 was known, we explore whether hsa_circ_0075451 added prognostical value to the well-established clinical and molecular factors. First of all, we entered clinical factors like age, WBC, and molecular factors (including ELN favorable genotype and *DNMT3A*, *IDH1*, and *IDH2* mutations) into Cox regression model 1. Next, we added this circRNA signature into model 1 to construct model 2. Akaike information criterion (AIC) decreased from 1003 of model 1 to 999 of model 2, and predictive error also significantly decreased compared the model 1 and model 2 by ANOVA (chi-square *χ*^2^ = 5.34, *P* = 0.02). At last, the area under the ROC curve for this circRNA signature alone was 0.57, that for clinical and molecular predictors combined was 0.65, and that for both was 0.66 for predicting 3-year overall survival. Thus, this circRNA signature had a weak predictive ability for overall survival (Additional file 1: Fig. S5).

### In silico prediction of hsa_circ_0075451-regulated network

**Fig. 4:**
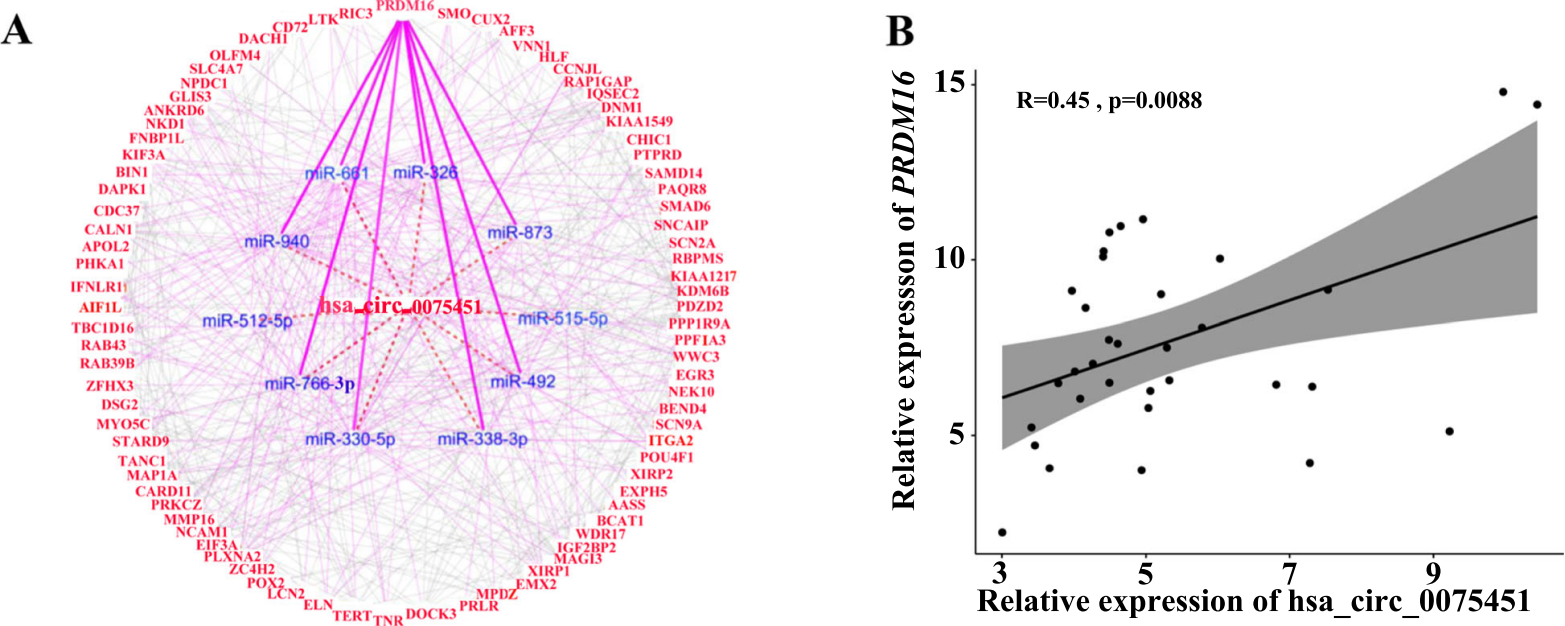
The regulatory network of circRNA-miRNA-mRNA in AML. **a** This network consists of hsa_circ_0075451, 10 miRNAs, and 84 genes. In this network, red labels represent upregulated genes in the patients with high expression of hsa_circ_0075451, and blue ones represent downregulated miRNAs. Gray lines represent the protein and protein interactive relationship of the encoding genes, purple lines represent miRNAs interacting with genes, and lines represent hsa_circ_0075451 sponging miRNAs, and bold purple lines represent 8 miRNAs’ loss of ability to inhibit expression of *PRDM16*. **b** Correlation relationship of the expression of hsa_circ_0075451 and *PRDM16* in this study.

We analyzed the mRNA expression profile in 7 CN-AML patients with high and 7 cases with low expression of hsa_circ_0075451. Clinical characteristics of these patients are summarized in Additional file 1:Table S10. Gene expression profiling for the high vs. low group identified 775 differentially expressed genes using an FDR correction *P* < 0.05 and a threshold for log_2_ fold change (log_2_CPM) > |1|(Additional file 1: Fig. S6 and Table S11). These differentially expressed genes are involved in 50 GO terms and 17 KEGG pathways, such as sequence-specific DNA, snoRNA, regulatory region nucleic acid and RNA polymerase binding, Wnt signaling pathway, sarcosine oxidase activity, and arginine and proline metabolism (Additional file 1:Table S12). In addition, we identified 15 miRNAs that potentially interacted with hsa_circ_0075451 in the CircInteractome database (Additional file 1: Fig. S7). By means of miRNA-mRNA integrative analysis in multiple databases (Additional file 1: Fig. S3), we found 133 genes upregulated in high expressers and predicted to be targeted by the interesting miRNAs. We selected miRNAs targeting the 3′ part UTR of genes (Additional file 1: Fig. S3) and hub genes to construct the regulatory network. The hub genes were defined as more than 3 genes clustering together using the STRING database. Finally, we found 10 miRNAs targeted 84 hub genes in the circRNA-miRNA-mRNA network (Fig. 4). Specifically, hsa_circ_0075451 binds to the miRNAs of miR-515-5p, miR-873, miR-766-3p, miR-940, miR-661, miR-492, miR-330-5p, miR-326, miR-512-5p, and miR-338-3p genes. Notably, *PRDM16* was predicted to be targeted by miR-873, miR-940, miR-766-3p, miR-661, miR-492, miR-330-5p, miR-338-3p, and miR-326. The positive correlation between the expression of *PRDM16* and hsa_circ_0075451 in the 14 cases was also validated in 34 CN-AML patients (Fig. 4b). Additionally, the expressions of each miR are positively correlated with each other, while these miRs are negatively associated with the expression of hsa_circ_0075451 in 9 AML cell lines (Additional file 1: Fig. S8). Although we focus on 84 hub genes instead of 775 differentially expressed genes, their critical GO terms are closely similar (Additional file 1: Table S13). Moreover, the activation of branched-chain amino acid (BCAA; valine, leucine, and isoleucine, KEGG ID: 82954) pathway and hedgehog signaling pathway (KEGG ID: 83063) as previously reported with potential biological and clinical significance [19, 20] is prominent in this final analysis (Additional file 1: Table S13), implying the novel drug glasdegib targeting leukemia stem cells via this regulatory network.

### The regulatory axis of hsa_circ_0075451 -| miR-326/ miR-330-5p -| *PRDM16*

**Fig. 5:**
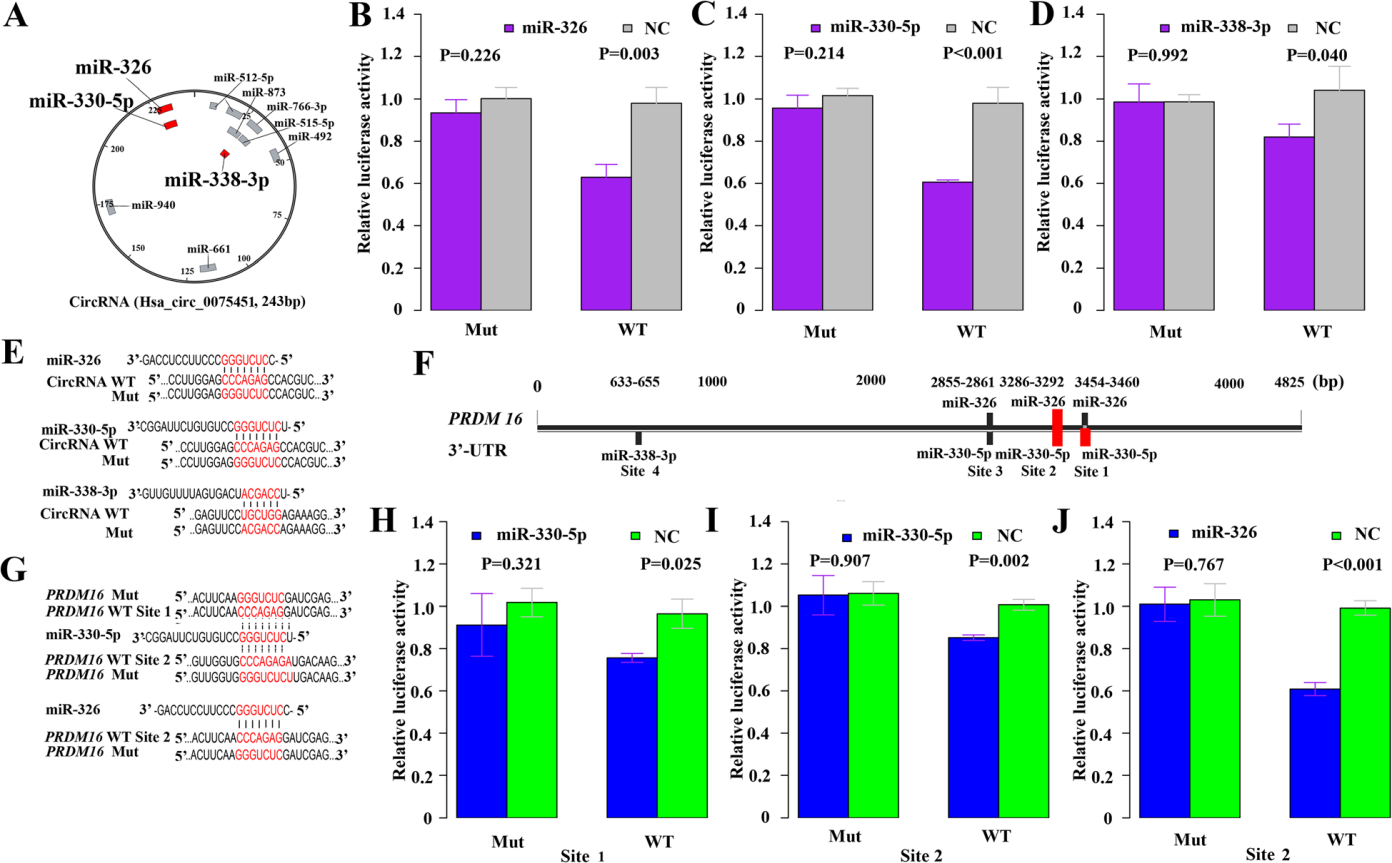
The regulatory axis of hsa_circ_0075451 -| miR-326/miR-330-5p | *PRDM16*. Hsa_circ_0075451 has putative binding sites of ten miRNAs (**a**). Dual luciferase reporter assays show evidence of three miRNAs (miR-326, miR-330-5p, and miR-338-3p; **a**, red bars) interacting with hsa_circ_0075451. Luciferase activity was analyzed in cells cotransfected with each miRNA and hsa_circ_0075451-WT or hsa_circ_0075451-MUT (**b**–**d**). The binding sequences of 3 miRNAs and circRNA are illustrated (**e**). The putative binding sites (**f**) and sequences (**g**) of miR-326, miR-330-5p, and miR-338-3p are located in the 3′-UTR of *PRDM16*. Dual luciferase reporter assays were performed to assess the interaction between miRNAs and *PRDM16*. Two binding sites of miR-330-5p and one of miR-326 (**f**, red bars) are validated to exist in 3′-UTR of *PRMD16* (**h**–**j**).

hsa_circ_0075451 has the potential ability to sponge ten miRNAs in the above silico analysis (Fig. 5a). Next, luciferase reporter assays were performed to verify their interactions. As a result, the luciferase activities decreased significantly in cells cotransfected with miR-326, miR-330-5p, and miR-338-3p mimics and hsa_circ_0075451 wild-type sequences compared with each miRNA cotransfected with hsa_circ_0075451 mutant sequences (Fig. 5b–d). Their interactive sequences are shown in Fig. 5e. However, other miRNAs like miR-766-3p, miR-515-5p, miR-873, miR-940, miR-661, miR-492, and miR-512-5p were not interacted with hsa_circ_0075451 (Additional file 1: Fig. S9). As shown in Fig. 5f, the 3′-UTR of *PRDM16* contains four binding sites of miR-326, miR-330-5p, and miR-338-3p. Interestingly, miR-326 and miR-330-5p share three binding sites (sites 1–3), and their binding sequences are illustrated in Fig. 5g. When cells were cotransfected with miR-330-5p and wild-type binding site 1 and site 2, the luciferase activity was significantly decreased (Fig. 5h, i). Meanwhile, we only observed the decreased luciferase activity when cotransfection of miR-326 mimics and the binding site 2 (Fig. 5j). There was no significant difference in luciferase activity in cells cotransfected with miR-338-3p and site 4, miR-326 and site 1/3, and miR-330-5p and site 3 (Additional file 1: Fig.S10). Thus, we found out that hsa_circ_0075451 can regulate the *PRDM16* expression via mutual interactions with miR-326/miR-330-5p in the dual luciferase assay. In order to further validate these interactions, we further transfected shRNAs targeting the junction sites of circGMDS into THP-1 and OCI-AML2 cells. These shRNAs significantly decreased expression of hsa_circ_0075451 together with increased miR-330-5p and miR-326 expression in the hsa_circ_0075451 knockdown experiment (Additional file 1: Fig.S11). Moreover, RNA FISH assay revealed that hsa_circ_0075451 and miR-330-50/miR-326 were mainly colocalized in the cytoplasm (Additional file 1: Fig. S12). In summary, the above results indicated that hsa_circ_0075451 can directly bind to miR-330-5p and miR-326, thereby affecting the expression of *PRDM16*.

### Hsa_circ_0075451 expression is associated with a unique metabolic feature

We used bone marrow blasts (1 × 10^7^ cells) with high (*n* = 7) and low (*n* = 7) hsa_circ_0075451 expression to assess the metabolic differences. The detailed clinical information is summarized in Additional file 1:Table S14. We found 16 metabolic signatures with statistically significant changes. As shown in Additional file 1: Fig. S13, dodecanoic acid along with isoleucine, fructose, glutamine, tryptophan, and others had significant changes. Consistent with the regulated genes, low levels of dodecanoic acid occurred in high expressers. The differently expressed metabolites were involved in 22 metabolic pathways such as valine, arginine and proline metabolism, sphingolipid metabolism, starch and sucrose metabolism, primary bile acid biosynthesis, and so on (Additional file 1:Table S15).

## Discussion

Acute myeloid leukemia (AML) is rising in the population with dismal outcome. It is urgent to identify the high-risk patients under the standard chemotherapy at the time of the initial diagnosis. Currently, the well-established biomarkers include clinical and genetic features. For instance, chromosomal abnormalities have been proved as an effective risk stratification tool. However, approximately 50% of AML patients have normal karyotypes. They are currently defined as CN-AML patients and can be further stratified by mutated genes [21, 22]. However, these biomarkers often interact or cluster with each other. For example, *NPM1* mutations coexist with mutations in *FLT3*-ITD and/or *DNMT3A* and/or *IDH1/2*. Consequently, the predictive value of *NPM1* mutations would be confounded by these factors. Thus, identifying independent predictors is becoming more and more important in clinical practices. circRNAs are believed to be novel biomarkers in several solid tumors. But very little is known on whether it can be used as an independent predictor for AML patients.

In this study, we enrolled 30 CN-AML patients with poor survival and selected 30 cases with long-term survival by matching the well-established factors such as age, WBC, and mutations in *FLT3*-ITD, *CEBPA*, *NPM1*, *DNMT3A*, *IDH1*, and *IDH2* genes. By the propensity score analysis, we excluded the confounders and identified 308 circular RNAs. We further focused on a GO term of carbon-oxygen lyase activity (GO:0016835) and identified hsa_circ_0075451 as a potential survival predictor. In order to confirm the independent prognostic value, we estimated the sample size and enrolled enough patients to perform stratification analysis and multivariate analysis in an independent cohort of CN-AML patients. Based on these analyses, we proved hsa_circ_0075451 to be a reliable and independent predictor in the training and validated cohort.

In addition, via the analysis of the different expression of targeted genes combining with sequences interaction analyzed in silico, we constructed the circRNA-miRNA-mRNA regulatory network (Fig. 4). hsa_circ_0075451 might sponge 10 miRNAs to upregulate 84 genes, which are involved in multiply pathways that may lead to leukemia progression. A wealth of evidence has suggested that 10 miRNAs (miR-512-5p, miR-330-5p, miR-326, miR-338-3p, miR-515-5p, miR-873, miR-766-3p, miR-940, miR-661, and miR-492) can function as tumor suppressors in different types of cancer such as head and neck squamous cell carcinoma, melanoma, gastric cancer, breast cancer, colon cancer, ovarian cancer, human non-small cell lung cancer, and cervical cancer [23–30]. Furthermore, some miRNAs have the ability to inhibit some oncogenes of *EXPH5*, *DAPK1*, *ITGA2*, *PLXNA2*, *SLC4A7*, *DSG2*, *PPP1R9A*, *KIAA1549*, and *TANC1* in the RAS signaling pathway [31]; *PTPRD*, *MMP16*, *AFF3*, *IFNLR1*, and *PRDM16* in the nuclear factor kappa B signaling pathway [32]; *CD72* and *CARD11* in the BCR signaling pathway [33]; *TERT*, *EIF3A*, *PRKCZ*, *ITGA2*, *CDC37*, *TNR*, and *PRLR* in the PI3K-Akt signaling pathway; and *SMO* and *KIF3A* in the hedgehog signaling pathway [34]. Others might inhibit genes of *FNBP1L*, *ZC4H2*, *MMP16*, *PPP1R9A*, *AFF3*, *MAGI3*, *KIAA1217*, *ZFHX3*, *TANC1*, *PRKCZ*, and *PDZD2* which were identified as a prostate cancer dependency-regulating RNA splicing [35]; *FNBP1L*, *SLC4A7*, *DSG2*, *MAGI3*, *KIAA1217*, *TANC1*, *PRKCZ*, *NCAM1*, and *OLFM4* involving in cell-to-cell interactions that facilitate cell adhesion and inversion [36]; and *LCN2* (*NGAL*) and *OLFM4* (*GW112*) selectively express in colon cancer and serve as selective targets [37]. Notably, some genes are involved in epigenetic and metabolic changes. For example, *BCAT1* enhances branched-chain amino acid production and promotes myeloid leukemia progression [38]. While *AASS* controls the first two steps in the lysine degradation pathway, lysine modification takes part in the transcript regulation reported in the pan-cancer analysis [39]. *PRDM16* acts as a transcription coregulator that controls the development of brown adipocytes and also involves in the GO:0046974 term of histone methyltransferase activity (H3-K9 specific). Recently, *PRDM16* was shown to play an important role in the pathogenesis of MDS and AML [40]. Finally, we identified a regulatory axis of hsa_circ_0075451 -| miR-330-5p/miR-326-| *PRDM16* in AML cells by the dual luciferase report assay. Taken together, these target genes specifically for *PRMD16* coexist in multiple functions of transduction signaling, transcription regulation, epigenetic modification, and metabolic change. Thus, we further measured the cellular metabolites to understand the metabolic features of high expressers. As a result, we found highly expressed hsa_circ_0075451 was associated with higher levels of cellular energy sources such as fructose, methionine, glutamine, arginine, serine, and isoleucine and lower levels of dodecanoic acid. These results suggest that blasts with upregulated hsa_circ_0075451 expression can affect fatty acid synthesis using other metabolites like BCAA as substrates. Therefore, this may explain the reason that hsa_circ_0075451 causes the poor survival may be via sponging these miRNAs and in turn leading to overexpression of oncogenes. Recent study identified fructose as a crucial factor in blasts’ survival and drug resistance [41]. In our study, cellular fructose was increased in patients with highly expressed circRNA, implying another underlying mechanism of overexpressed fructose in AML.

There are also some limitations to this study. Firstly, the training group might not be large enough to identify all of the aberrant circRNAs. Secondly, patients in the training group were selected from the prospective clinical trial, while the validated group was enrolled from the single center based on the retrospective study. Thirdly, the selected patients for mRNA profiling and metabolic analyses were not from the same cases. Therefore, upstream changes of mRNAs coding enzymes might not translate exactly into the downstream changes of metabolites. In spite of some limitations, this is one of the few studies to identify independent and reliable circRNAs with prognostic significance by the discovery and validation study design.

## Conclusion

In this study, we identified hsa_circ_0075451 as an independent predictor in CN-AML.

## Supplementary Information

**Additional file 1: FigS1.** [Distribution of propensity score analysis in 75 patients with the high quality of bone marrow samples]. **FigS2.** [Sequences inserted into pmirGLO reporter plasmid confirmed by Sanger sequencing]. **FigS3.** [Flowchart of constructing circRNA-miRNA-mRNA regulatory network]. **FigS4.** [Hazard ratios of altered hsa_circ_0075451 expression on overall survival by stratified analyses]. **FigS5.** [The predictive power of CircRNA (hsa_circ_0075451) and clinical and molecular factors]. **FigS6.** [Differentially expressed genes between high and low hsa_circ_0075451 expression]. **FigS7.** [In silico analysis of 15 miRNAs potentially interacted with hsa_circ_0075451 in the CircInteractome database]. **FigS8**. [Correlation relationship between hsa_circ_ 0075451 and microRNAs in 9 AML cell lines]. **FigS9.** [Dual luciferase reporter assays to test the interactive relationship between hsa_circ_0075451 and miRNAs]. **FigS10.** [Dual luciferase reporter assays to test the interactive relationship between 3’-UTR of *PRMD16* and miRNAs]. **FigS11.** [ShRNA interference assay was used to further confirm the regulated relationship of hsa_circ_0075451 -| miR-326/ miR-330-5p -| *PRDM16*]. **FigS12.** [RNA FISH for hsa_circ_0075451(circGMDS) and miR-330-5p/miR-326 was detected in OCI-AML2 cells]. **FigS13.** [Metabolic changes in patients with aberrant hsa_circ_0075451 expression]. **Table S1.** [ Clinical characteristics of patients in the training group]. **Table S2.** [Primers of cRNA /miRNAs]. **Table S3.** [The sequences using for dual luciferase reporter assay]. **Table S4.** [Aberrantly expression of the circular RNA signatures between favorable and unfavorable group]. **Table S5.** [CircRNAs related to outcome validated by Cox regression analysis]. **Table S6.** [Gene Ontology and pathway analysis of survival associated circRNAs]. **Table S7.** [Different expression of circRNAs which encoding genes enriched in the GO:0016835 term]. **Table S8.** [Characteristics of CN-AML patients with high and low hsa_circ_0075451 expression in the validated cohort]. **Table S9.** [Landmark analysis of CN-AML patients]. **Table S10.** [Clinical characteristics of patients for mRNA profiling analysis]. **Table S11.** [mRNAs expression between high and low hsa_circ_0075451]. **Table S12.** [Gene enrichment analysis of the differentially expressed genes]. **Table S13.** [KEGG pathways and GO enrichment analyses in the 84 hub genes.]. **Table S14.** [Clinical characteristics of patients for the metabolomic analysis]. **Table S15.** [Pathways enriched analysis].

**Additional file 2.** : Dataset 1.

**Additional file 3.** : Dataset 2.

**Additional file 4.** : Dataset 3.

## Data Availability

The datasets used in this study are available in the supplementary data.

## Abbreviations

CN-AML: Cytogenetically normal acute myeloid leukemia
ELN: European LeukemiaNet
GO: Gene Ontology
OS: Overall survival
