# Supplementary material for: Development and validation of a novel circular RNA as an independent prognostic factor in acute myeloid leukemia
Source: BMC Med. 2021 Feb 1;19:28. doi: 10.1186/s12916-020-01898-y (PMC7849103; doi:10.1186/s12916-020-01898-y)
Supplement: Supplementary file 1 — Additional file 1: FigS1. [Distribution of propensity score analysis in 75 patients with the high quality of bone marrow samples]. FigS2. [Sequences inserted into pmirGLO reporter plasmid confirmed by Sanger sequencing]. FigS3. [Flowchart of constructing circRNA-miRNA-mRNA regulatory network]. FigS4. [Hazard ratios of altered hsa_circ_0075451 expression on overall survival by stratified analyses]. FigS5. [The predictive power of CircRNA (hsa_circ_0075451) and clinical and molecular factors]. FigS6. [Differentially expressed genes between high and low hsa_circ_0075451 expression]. FigS7. [In silico analysis of 15 miRNAs potentially interacted with hsa_circ_0075451 in the CircInteractome database]. FigS8. [Correlation relationship between hsa_circ_ 0075451 and microRNAs in 9 AML cell lines]. FigS9. [Dual luciferase reporter assays to test the interactive relationship between hsa_circ_0075451 and miRNAs]. FigS10. [Dual luciferase reporter assays to test the interactive relationship between 3’-UTR of PRMD16 and miRNAs]. FigS11. [ShRNA interference assay was used to further confirm the regulated relationship of hsa_circ_0075451 -| miR-326/ miR-330-5p -| PRDM16]. FigS12. [RNA FISH for hsa_circ_0075451(circGMDS) and miR-330-5p/miR-326 was detected in OCI-AML2 cells]. FigS13. [Metabolic changes in patients with aberrant hsa_circ_0075451 expression]. Table S1. [ Clinical characteristics of patients in the training group]. Table S2. [Primers of cRNA /miRNAs]. Table S3. [The sequences using for dual luciferase reporter assay]. Table S4. [Aberrantly expression of the circular RNA signatures between favorable and unfavorable group]. Table S5. [CircRNAs related to outcome validated by Cox regression analysis]. Table S6. [Gene Ontology and pathway analysis of survival associated circRNAs]. Table S7. [Different expression of circRNAs which encoding genes enriched in the GO:0016835 term]. Table S8. [Characteristics of CN-AML patients with high and low hsa_circ_0075451 expression in [file 12916_2020_1898_MOESM1_ESM.docx]

**Circular RNA as an independent prognostic factor in acute myeloid leukemia**

**Supplementary methods**

**Treatment protocols**

Patients with cytogenetically normal AML (CN-AML) in training group were enrolled from the clinical trial (ChiCTR-IPR-17012643). Circular RNA expression profiling was analyzed in bone marrow samples from these 60 patients as the training group. Treatment protocols were seen in the website of the clinical trial. Patients from the validated cohort received Idarubicin/Ara-C (IA)-, Donorubicin/Ara-C (DA) - or Homoharringtonine and Aclarubicin/Ara-C (HAA)-based induction chemotherapy, and consolidation chemotherapy based on the treating physician’s choice in an individualized manner. Upon recovery of the periphery blood count, a bone marrow aspirate was performed to assess the status of the response. Patients who achieved complete remission received consolidation therapy. None of the patients included in this analysis underwent hematopoietic stem cell transplantation (HSCT) after achievement of the first complete remission (CR). For patients with PR or blast decrease in bone marrow within 60%, the same DA or IA or HAA regimen was repeated. In the consolidation therapy, older patients were treated with homoharringtonine (HHT) and cytarabine (HA), aclarubicin and and cytarabine (AA) or mitoxantrone and cytarabine (MA) decided by the physicians in an individualized manner. Doses of every drug were showed as following: HHT was administered at a dose of 2 mg/m^2^ for 3 days; Ara-c was given at a dose of 75 mg/m^2^ twice daily for 7 days; aclarubicin was administered at a dose of 12 mg/m^2^ daily for 7 days; mitoxantrone was given at a dose of 8 mg/m^2^ for 3 days. Antibiotic and antifungal treatment and supportive care were given according to common guidelines.

**Cytogenetic and gene mutation analysis**

Cytogenetic and molecular studies were performed centrally at ZIH molecular laboratories. Mononuclear cells were isolated from the BM samples by Ficoll-Hypaque density-gradient centrifugation and DNA and RNA were extracted as described previously^[12](#_ENREF_12)^. Mutation analyses of *NPM1*, *FLT3*ITD, *CEBPA*, *DNMT3A*, *IDH1* and *IDH2* were carried out as described previously^[12](#_ENREF_12)^.

**Serum metabolomic profiles**

Metabolomic profiling of serum samples was performed using GC-TOFMS platforms as previously described^[13](#_ENREF_13), [14](#_ENREF_14)^. A total of 71 metabolites were identified by the comparison with the internal library built with the standard reference compounds. The intensities of these metabolites were used to further analyze. T test analysis was used to test for metabolites significantly different among between each two groups.

Sample Preparation and GC-TOFMS Analysis

Bone marrow samples collected at disease diagnosis were stored frozen at −80 °C until use. Each 10^7 of bone marrow blasts was added into a 1.5 mL of tube followed by the addition of 400 μL of acetone for protein precipitation. The mixture was stirred by vortex for 30 s and centrifuged at 10

000 rpm for 10 min. A 400-μL supernatant was transferred to a 500 μL of glass tube and dried under vacuum. The dried analytes were dissolved in 80 μL of methoxylamine hydrochloride (15 mg/mL, dissolved in pyridine) for 90 min at 30 °C and then silylated with 80 μL N,O-bis-trimethylsilyl-trifluoroacetamideand Trimethylchlorosilane (in a ratio of 99:1) (Supelco) for 2 h at 70 °C. Each 70-μL aliquot of hexane was added to the derivatization bottles. After the sample was stirred for 1 min and kept at room temperature for an hour, 1-μL aliquot of the solution was injected into a PerkinElmer gas chromatography coupled with a TurboMass-Autosystem XL mass spectrometer (PerkinElmer, Inc.) in the splitless mode. A DB-5MS capillary column coated with 5% Diphenyl cross-linked 95% dimethylpolysiloxane (30 m × 250 μm i.d., 0.25-μm film thickness; Agilent J&W Scientific, Folsom, CA) was used for separation. Both the injection temperature and the interface temperature were set to 260°C, and the ion source temperature was adjusted to 200°C. Initial GC oven temperature was set at 80°C for 2 min after injection, and was raised up to 285°C with 5°C/min and maintained at 285°C for 7 min. Helium at a flow rate of 1 mL/min was used as the carrier gas. The measurements were made with electron impact ionization (70 eV) in the full scan mode (m/z 30−550). A total of 71 metabolites were identified by the comparison with the internal library built with the standard reference compounds.

**Gene expression arrays**

Fourteen BM samples of CN-AML patients were used to assess the mRNA expression profiling. Total RNA from the fresh frozen samples was isolated by Trizol reagent (Invitrogen life technologies). RNA quality was evaluated using a Nanodrop ND-1000 (Thermo Fisher Scientific, Waltham, MA, USA). Transcriptome high throughput sequencing was done by Cloud-Seq Biotech (Shanghai, China). Briefly, total RNA was used for removing the rRNAs using Ribo-Zero rRNA Removal Kits (Illumina, USA) following the manufacturer's instructions. RNA libraries were constructed by using rRNA-depleted RNAs with TruSeq Stranded Total RNA Library Prep Kit (Illumina, USA) according to the manufacturer’s instructions. Libraries were controlled for quality and quantified using the BioAnalyzer 2100 system (Agilent Technologies, USA). Paired-end reads were harvested from Illumina HiSeq 4000 sequencer, and were quality controlled by Q30. After 3’ adaptor-trimming and low quality reads removing by "cutadapt" software (v1.9.3). The high quality trimmed reads were used to mRNA analyses. The high quality reads were aligned to the human reference genome (UCSC hg19) with hisat2 software. Then, guided by the Ensembl gtf gene annotation file, cuffdiff software (part of cufflinks) was used to get the FPKM as the expression profiles of mRNA. GO and KEGG Pathway enrichment analysis were also performed based on the differentially expressed mRNAs.

**Fluorescence in situ hybridization**

Cy3-labeled probes were specific to circGMDS and fam-labeled probes were specific to miR-330-5p and miR-326. The probes were designed and synthesized by Genepharma (Shanghai, China), and the signals of the probes were detected by a Fluorescent In Situ Hybridization Kit (Genepharma, Shanghai, China) in OCI-AML2 cells according to the manufacturer’s instructions. The images were acquired on Nikon A1R confocal microscope (Nikon, Japan).

**ShRNA interference assay**

To construct circGMDS knockdown plasmids, three shRNA sequences and a scramble shRNA NC were designed, synthesized and cloned into PGMLV-SC5 vector by Genomeditech (Shanghai, China), The sequences were as follows: NC 5’-TTCTCCGAACGTGTCACGT-3’ ; sh1 5’-ACCATCTTTGACGTGGCTC-3’ ; sh2 5’- GGTAGGAACCATCTTTGAC -3’;sh3 5’-CACGTCAAAGATGGTTCCT-3’. Transfection and lentivirus collection was carried out using ProFection® Mammalian Transfection System (Promega, USA) according to the manufacturer’s instructions. Total RNA and were collected 72h after transfection.

**Supplementary Figure Legends**


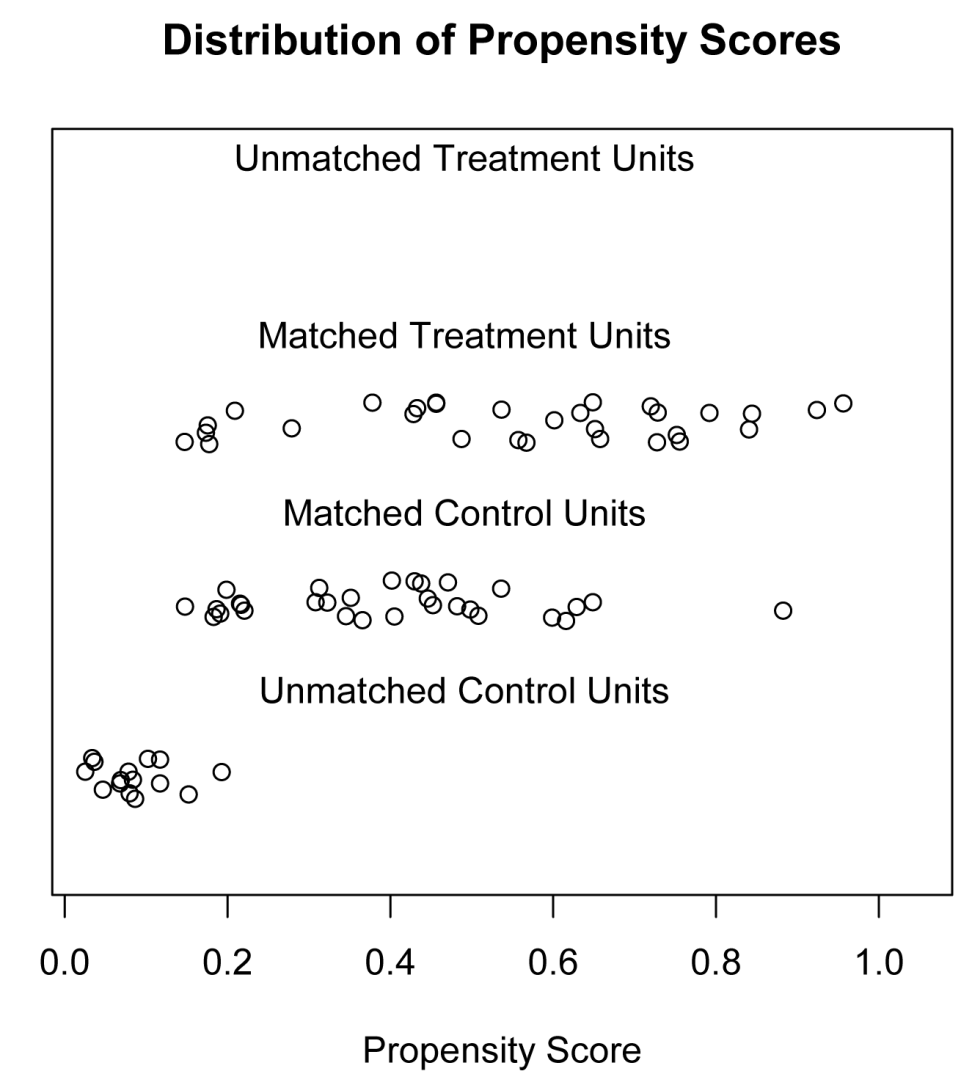


Fig S1. Distribution of propensity score analysis in 75 patients with the high quality of bone marrow samples. Treatment units represent 30 CN-AML patients with survival time less than 2 years, and control units represent 45 CN-AML with survival time more than 2 years.


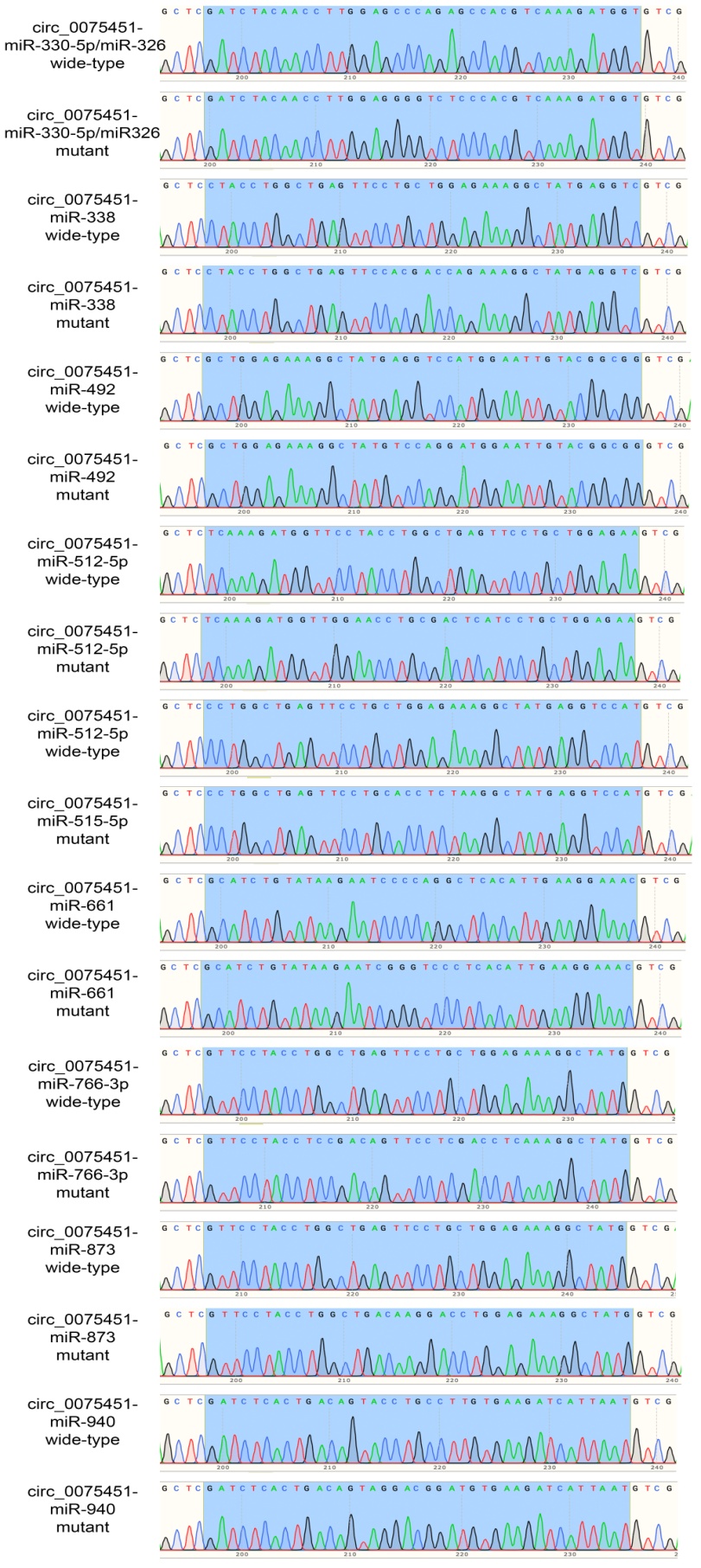


Fig S2. Sequences inserted into pmirGLO reporter plasmid confirmed by Sanger sequencing


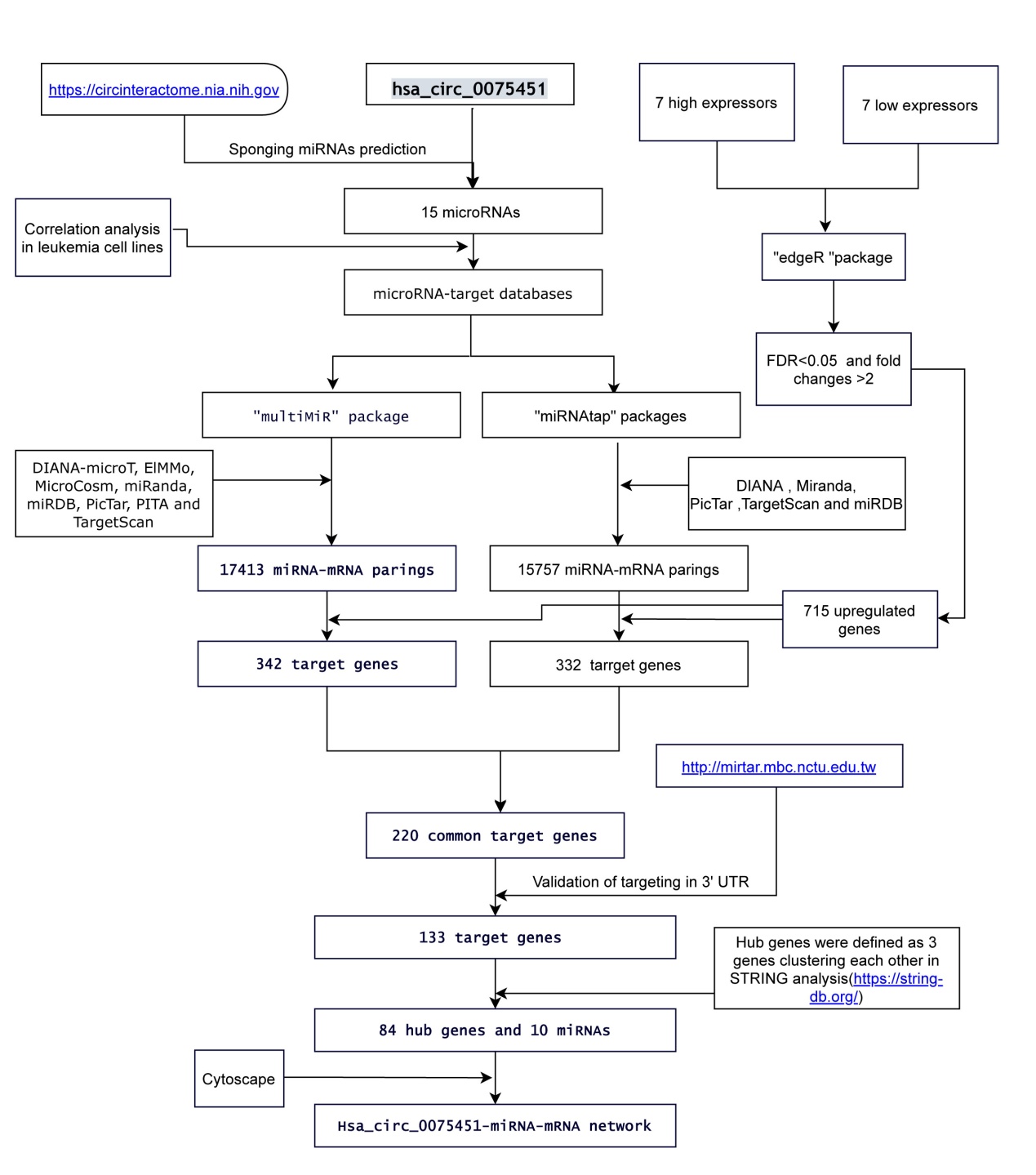


Fig S3. Flowchart of constructing circRNA-miRNA-mRNA regulatory network. CircRNAs can sponge miRNAs leading to upregulate expression of genes. Firstly, we predicted the sponging miRNAs by the online database of “Circular RNA Interactome” ([https://circinteractome.nia.nih.gov](https://circinteractome.nia.nih.gov/)). As a result, 15 microRNAs are predicted to be targeted by hsa_circ_0075451 (Figure S3). Their expression values positively correlate with each other and negatively correlate with hsa_circ_0075451 (Figure S8). Secondly, the potential target genes of these 15 microRNAs are predicted by the “multiMiR” and “miRNAtap” packages. These two packages contain DIANA, EIMMo, microcosm, miRanda, miRDB, Pictar, PITA and TargetScan database with different parameters. We determined 17413 and 15757 miRNA-mRNA parings respectively by the “multiMiR” and “miRNAtap” packages. Thirdly, we filtered the target genes using the 715 upregulated genes derived from high circ_0075451 expressors. Consequently, we obtained 342 and 332 target genes from the different prediction methods. Among these target genes, we found 220 genes commonly existed in these two methods. Notably, target sites in 3’ part of UTR are well-known to have the strong inhibiting ability of miRNAs. Therefore, we validated the target seeds in 3’ part of UTR of genes by the online database “miRTAR” ([http://mirtar.mbc.nctu.edu.tw](http://mirtar.mbc.nctu.edu.tw/)). Here, we found 133 target genes and 10 miRNAs, which link 352 miRNA-mRNA parings. Fourthly, we selected hub genes which cluster with more than 3 genes using STRING database, and constructed the circRNA-miRNA-mRNA regulatory network within 84 hub genes and 10 miRNAs using the cytoscape.


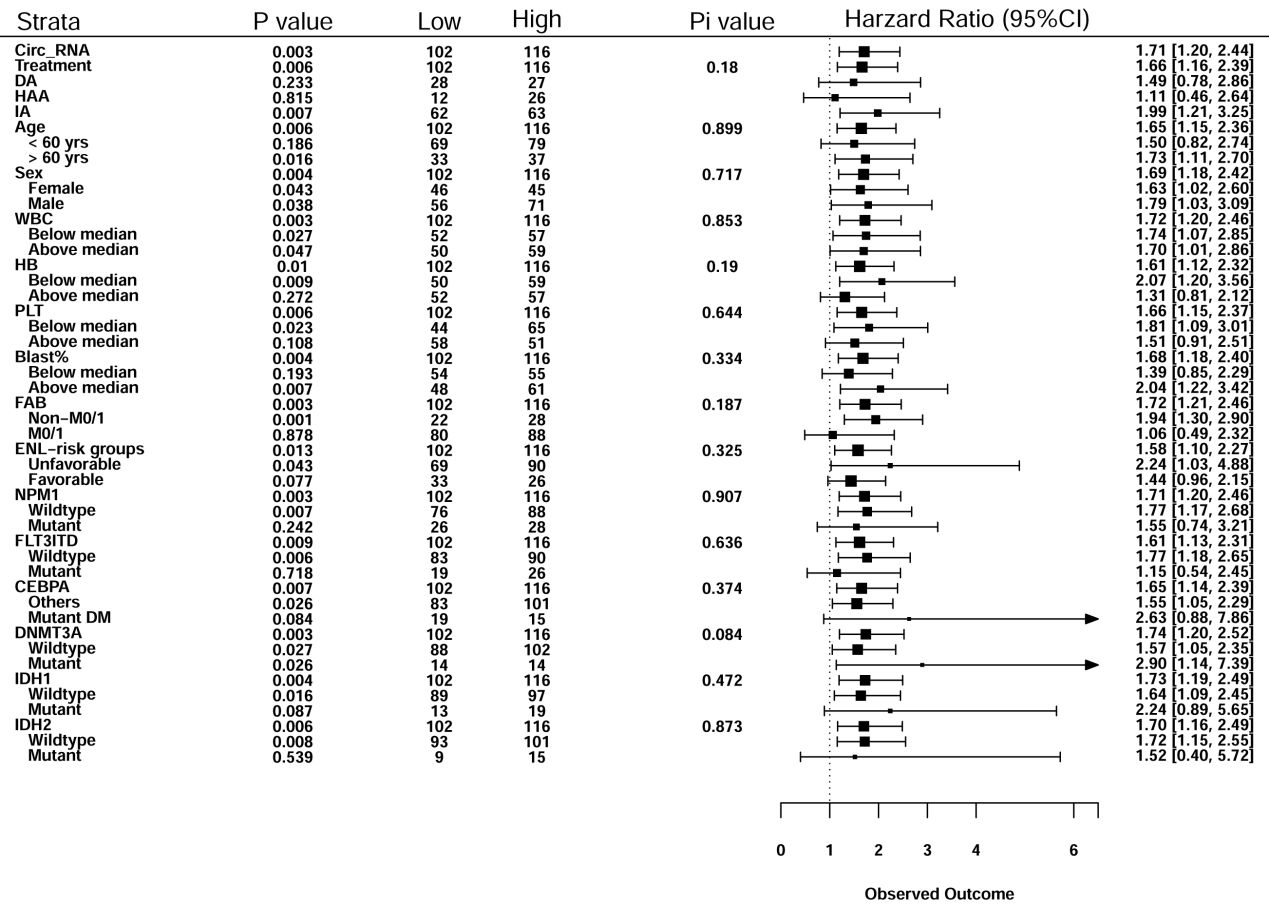


Fig S4. Hazard ratios (HR) of altered hsa_circ_0075451 expression on overall survival by stratified analyses. “P value” represents the p-values after adjustment analyses for each factor, and “Pi value” represents the p-values of interaction between altered levels of hsa_circ_0075451 expression and each factor. The prognostic value of hsa_circ_0075451 expression is significant in univariate analysis (Circ_RNA) for OS. The adjustment p-values do not strikingly change after adjustment for each factor, implying each adjusted factor does not significantly alter the interpretation of the prognostic value of hsa_circ_0075451 expression. The p-values of interaction (Pi value) between hsa_circ_0075451 expression and each factor are more than 0.05, indicating no apparent interactive factors exist.


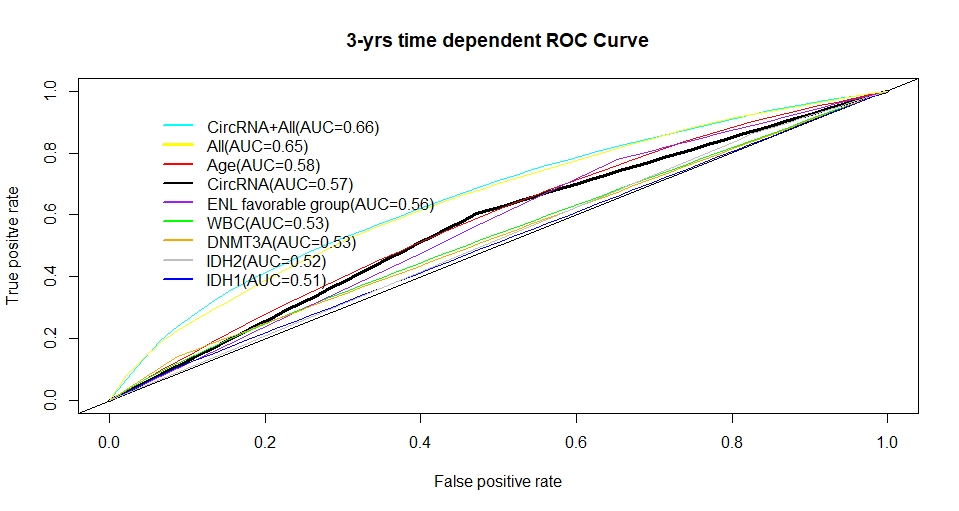


Fig S5. The predictive power of CircRNA (hsa_circ_0075451) and clinical and molecular factors. All presents clinical and molecular factors combined. AUC; area under the survival ROC curves.


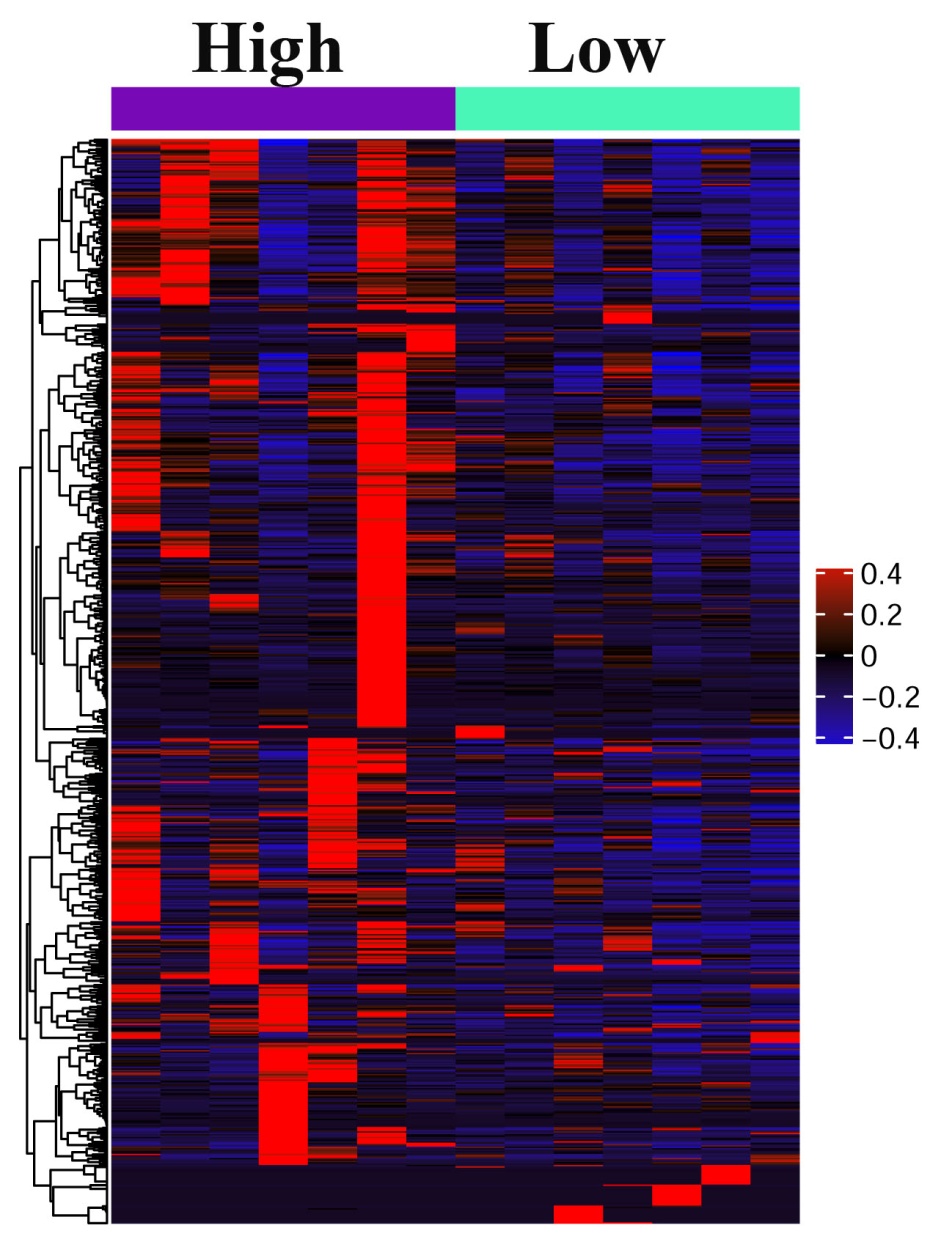


Fig S6. Differentially expressed genes between high and low hsa_circ_0075451 expression. Red colors represent high expression and blue represent low expression.


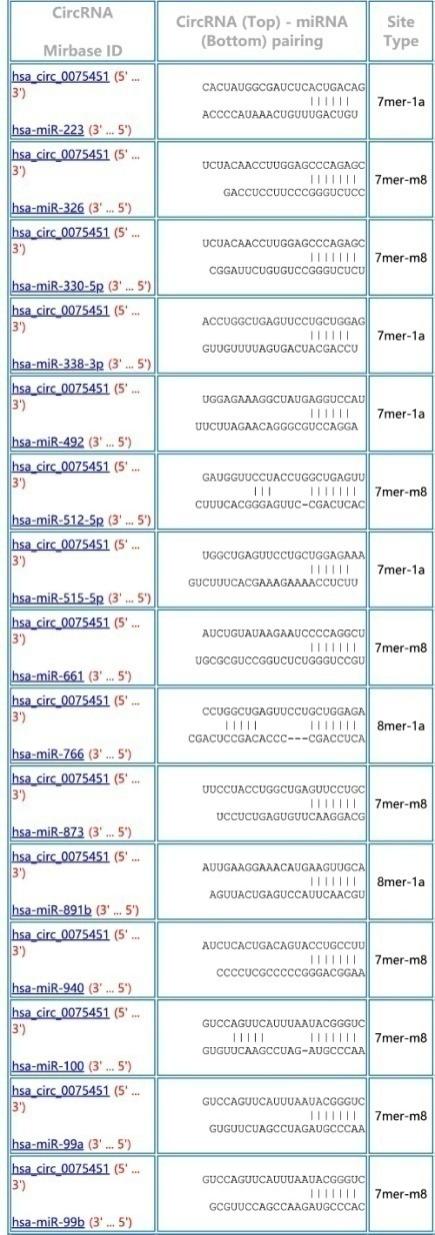


Fig S7. In silico analysis of 15 miRNAs potentially interacted with hsa_circ_0075451 in the CircInteractome database.


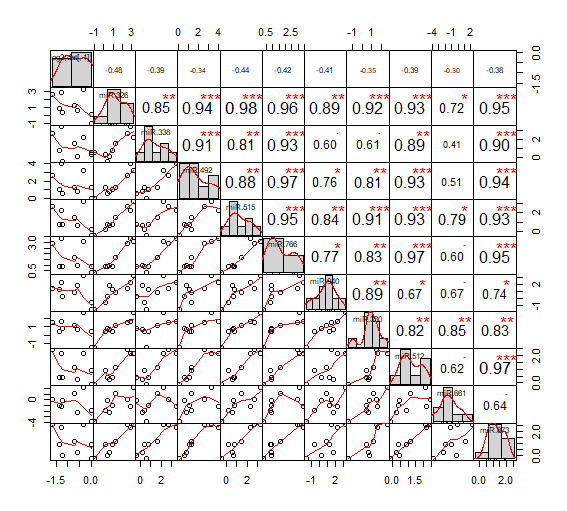


Fig S8. Correlation relationship between hsa_circ_ 0075451 and microRNAs in 9 AML cell lines. The expressions of each miR are positively correlated with each other while these miRs are negatively associated with the expression of hsa_circ_0075451.


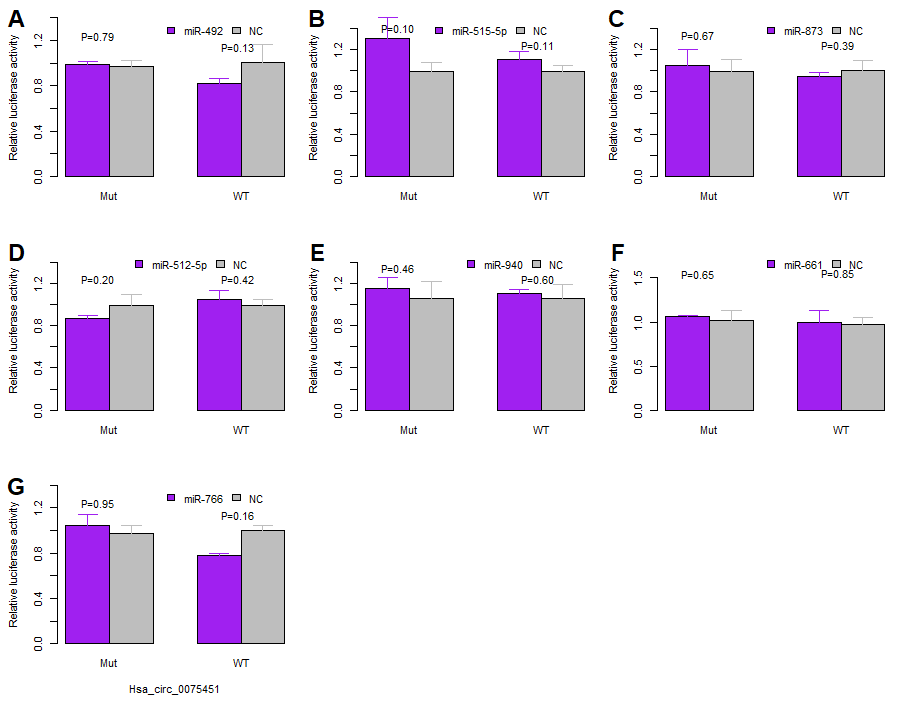


Fig S9. Dual luciferase reporter assays to test the interactive relationship between hsa_circ_0075451 and miRNAs.


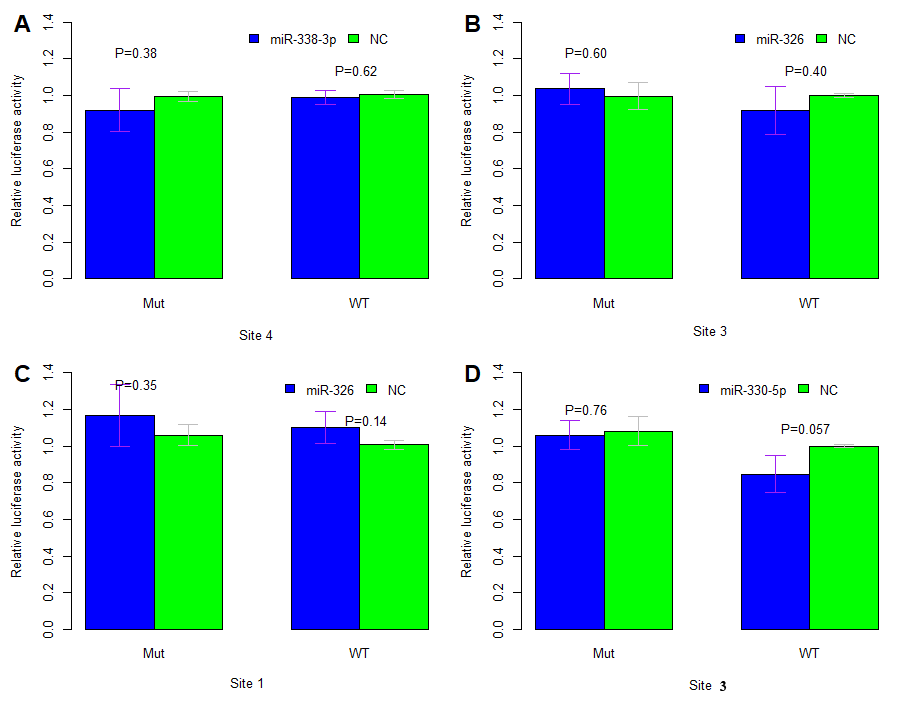


Fig S10. Dual luciferase reporter assays to test the interactive relationship between 3’-UTR of *PRMD16* and miRNAs.


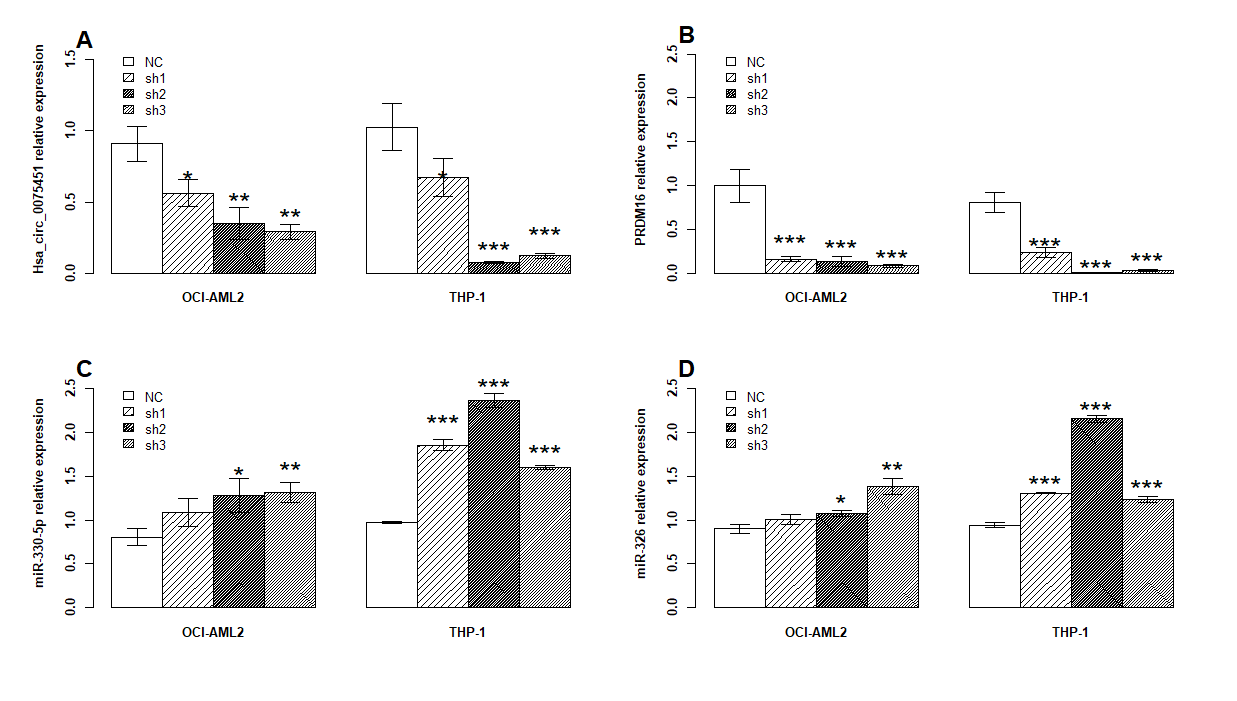


Fig S11. ShRNA interference assay was used to further confirm the regulated relationship of hsa_circ_0075451 -| miR-326/ miR-330-5p -| *PRDM16*. Hsa_circ_0075451 shRNAs (sh1,sh2 and sh3) were transfected to knock down in THP-1 and OCI-AML2 cells. These shRNAs significantly decreased the expression of hsa_circ_0075451 (A) and PRDM16(B). Next, we detected the levels of the candidate miRNAs miR-330-5p and miR-326, and the results revealed that miR-330-5p(C) and miR-326(D) were upregulated significantly by sh2 and sh3 in the two cell lines, and miR-326 was also upregulated significantly by sh1 in THP-1 cell lines. Expressions of circGMDS, *PRDM16*, miR-330-5p, miR-326 were measured by qRT-PCR. Data are mean ± SD, n = 3.


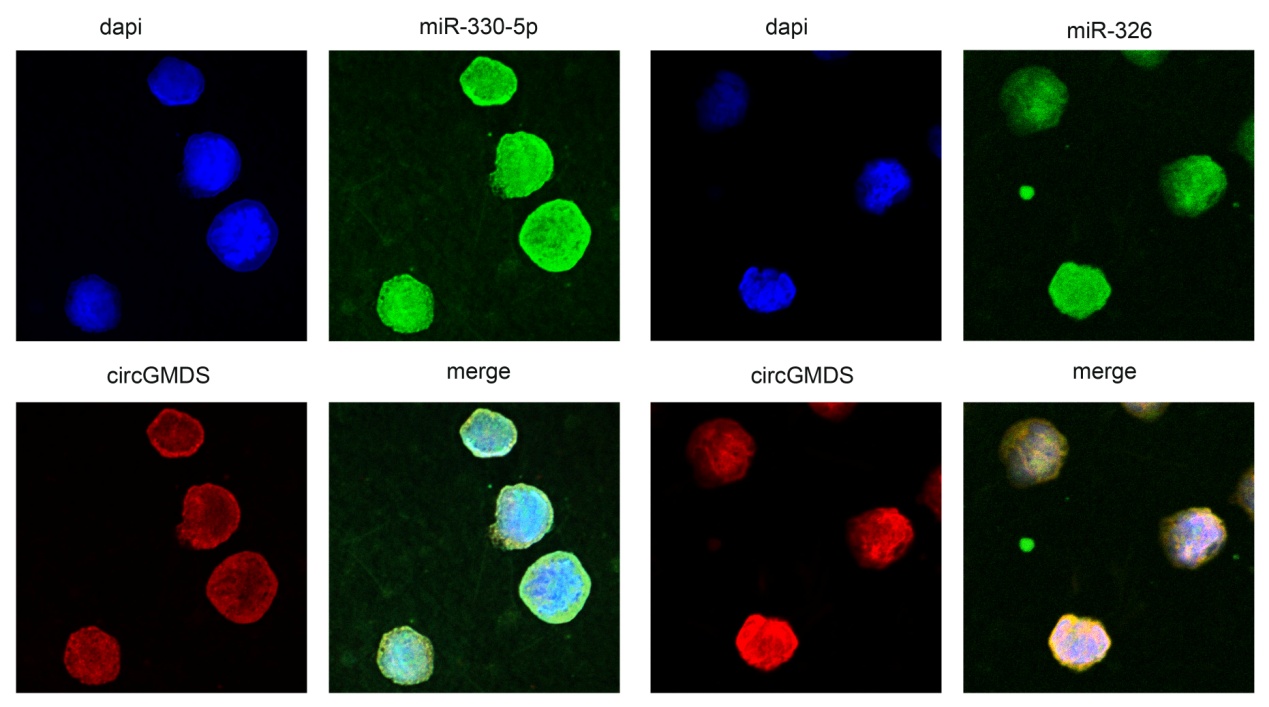


Fig S12. RNA FISH for hsa_circ_0075451**(**circGMDS) and miR-330-5p/miR-326 was detected in OCI-AML2 cells. Nuclei was stained blue (DAPI), circGMDS was stained red, and miR-330-5p/miR-326 were stained green.


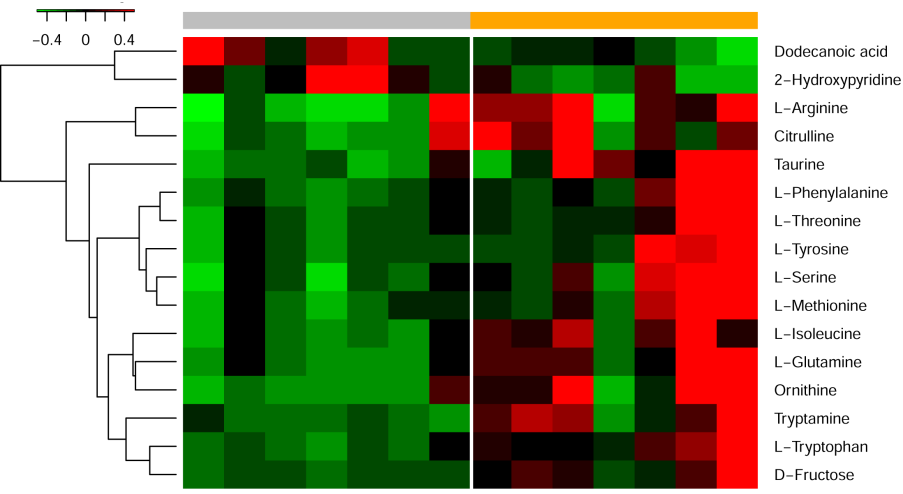


Fig S13. Metabolic changes in patients with aberrant hsa_circ_0075451 expression. Gray bar indicates low expressers, while brown bar indicates high expressers.

Table S1. Clinical characteristics of patients in the training group

| Variable | Survival >2 yrs | Survival < 2 yrs | P value |
| --- | --- | --- | --- |
| Number | 30 | 30 |  |
| Sex, male, n(%) | 16 (53.3) | 16 (53.3) | 1.000 |
| Age, median(range),years | 47.50 [36.50, 52.00] | 48.50 [41.25, 53.00] | 0.554 |
| WBC, median(IQR),×10^9/L^1^ | 13.00 [7.43, 22.77] | 28.65 [9.25, 49.35] | 0.165 |
| HB, median(IQR), g/L^2^ | 84.00 [66.00, 92.00] | 83.00 [72.50, 113.00] | 0.299 |
| PLT, median(IQR),×10^9/L^3^ | 39.00 [28.00, 50.00] | 45.50 [23.75, 89.75] | 0.343 |
| BM blast, median(IQR),%^4^ | 56.50 [40.50, 70.75] | 72.00 [47.00, 84.75] | 0.067 |
| FAB classification, n(%)^5^ |  |  | 0.882 |
| M0 | 2 ( 6.7) | 3 (10.0) | |
| M1 | 3 (10.0) | 4 (13.3) | |
| M2 | 12 (40.0) | 14 (46.7) | |
| M4 | 3 (10.0) | 1 ( 3.3) | |
| M5 | 9 (30.0) | 8 (26.7) | |
| M6 | 1 ( 3.3) | 0 ( 0.0) | |
| Gene mutations, n(%) | |  |  |
| *FLT3*-ITD | 4 (13.3) | 10 (33.3) | 0.125 |
| *NPM1* | 9 (30.0) | 8 (26.7) | 1.000 |
| *CEBPA*^DM6^ | 3 (10.0) | 1 ( 3.3) | 0.612 |
| *DNMT3A* | 2 ( 6.7) | 6 (20.0) | 0.254 |
| *IDH1* | 5 (16.7) | 7 (23.3) | 0.748 |
| *IDH2* | 4 (13.3) | 7 (23.3) | 0.506 |

Abbreviations: ^1^WBC, white blood cell counts; ^2^HB, hemoglobin; ^3^PLT, platelet counts; ^4^BM, bone marrow; ^5^ FAB, Franch-American-British;^6^DM, double allele. IQR, interquantile.

Table S2. Primers of cRNA /miRNAs

| CRNA/miRNA | Primer (5’-3’) |
| --- | --- |
| Hsa_circ_0075451 |  |
| convergent F | 5’- TGCACTATGGCGATCTCACTG-3’ |
| convergent R | 5’- ACTCAGCGAGGTCAAAGGAAA-3’. |
| GMDS mRNA |  |
| divergent F | 5’-ACTGACAGTACCTGCCTTGTG-3’ |
| divergent R | 5’-ATCAACTCTGTGAAGTTCT-3’ |
| CDR1as |  |
| divergent F | 5’- ACGTCTCCAGTGTGCTGA -3’ |
| divergent R | 5’- CTTGACACAGGTGCCATC -3’ |
| convergent F | 5’- TTTCCGATGGCACCTGTGTCAAG -3’ |
| convergent R | 5’- CTGGAAGACCTTGAGATTATTGGAAGAC -3’ |
| beta-actin |  |
| divergent F | 5’- AAAGGCGAGGCTCTGTGCT-3’ |
| divergent R | 5’- GGGCTTACCTGTACACTGACTTGA -3’ |
| convergent F | 5’- CATGTACGTTGCTATCCAGGC -3’ |
| convergent R | 5’- CTCCTTAATGTCACGCACGAT -3’ |
| U6 | TGCGGGTGCTCGCTTCGGCAGC |
| miR-515-5p | TTCTCCAAAAGAAAGCACTTTCTG |
| miR-326 | CCTCTGGGCCCTTCCTCCAG-3 |
| miR-873 | GCAGGAACTTGTGAGTCTCCT |
| miR-766-3p | ACTCCAGCCCCACAGCCTCAGC |
| miR-940 | AAGGCAGGGCCCCCGCTCCCC |
| miR-661 | TGCCTGGGTCTCTGGCCTGCGCGT |
| miR-492 | AGGACCTGCGGGACAAGATTCTT |
| miR-330-5p | TCTCTGGGCCTGTGTCTTAGGC |
| miR-512-5p | CACTCAGCCTTGAGGGCACTTTC |
| miR-338-3p | TCCAGCATCAGTGATTTTGTTG |

Table S3. The sequences using for dual luciferase reporter assay

|  | Sequence (5’-3’) |
| --- | --- |
| PRDM16-miR-330-5p/miR-326 wt1 | CCCAGCAGTGTTGGTGCCCAGAGATGACAAGGGCCAGGGA |
| PRDM16-miR-330-5p/miR-326 mut1 | CCCAGCAGTGTTGGTGGGGTCTCTTGACAAGGGCCAGGGA |
| PRDM16-miR-330-5p/miR-326 wt2 | ATATCACTGACTTCAACCCAGAGGATCGAGCCCCTGCACC |
| PRDM16-miR-330-5p/miR-326 mut2 | ATATCACTGACTTCAAGGGTCTCGATCGAGCCCCTGCACC |
| PRDM16-miR-330-5p/miR-326 wt3 | CCAAGACCTCCACGTCCCCAGAGTCCAGCCCTGGAAATTC |
| PRDM16-miR-330-5p/miR-326 mut3 | CCAAGACCTCCACGTCGGGTCTCTCCAGCCCTGGAAATTC |
| PRDM16-miR-338-3p wt | TCTTTATACTGTAGATAATGGAGAAATTTTCTATCTCTGT |
| PRDM16-miR-338-3p mut | TCTTTATACAGTTCATATAGGTCAATAAAACAATCTCTGT |
| Circ-miR-330-5p/miR-326 wt | GATCTACAACCTTGGAGCCCAGAGCCACGTCAAAGATGGT |
| Circ-miR-330-5p/miR-326 mut | GATCTACAACCTTGGAGGGGTCTCCCACGTCAAAGATGGT |
| Circ-miR-338-3p wt | CTACCTGGCTGAGTTCCTGCTGGAGAAAGGCTATGAGGTC |
| Circ-miR-338-3p mut | CTACCTGGCTGAGTTCCACGACCAGAAAGGCTATGAGGTC |
| Circ-miR-492 wt | GCTGGAGAAAGGCTATGAGGTCCATGGAATTGTACGGCGG |
| Circ-miR-492 mut | GCTGGAGAAAGGCTATGTCCAGGATGGAATTGTACGGCGG |
| Circ-miR-512-5p wt | TCAAAGATGGTTCCTACCTGGCTGAGTTCCTGCTGGAGAA |
| Circ-miR-512-5p mut | TCAAAGATGGTTGGAACCTGCGACTCATCCTGCTGGAGAA |
| Circ-miR-515-5p wt | CCTGGCTGAGTTCCTGCTGGAGAAAGGCTATGAGGTCCAT |
| Circ-miR-515-5p mut | CCTGGCTGAGTTCCTGCACCTCTAAGGCTATGAGGTCCAT |
| Circ-miR-661 wt | GCATCTGTATAAGAATCCCCAGGCTCACATTGAAGGAAAC |
| Circ-miR-661 mut | GCATCTGTATAAGAATCGGGTCCCTCACATTGAAGGAAAC |
| Circ-miR-766-5p wt | GTTCCTACCTGGCTGAGTTCCTGCTGGAGAAAGGCTATG |
| Circ-miR-766-5p mut | GTTCCTACCTCCGACAGTTCCTCGACCTCAAAGGCTATG |
| Circ-miR-873 wt | GTTCCTACCTGGCTGAGTTCCTGCTGGAGAAAGGCTATG |
| Circ-miR-873 mut | GTTCCTACCTGGCTGACAAGGACCTGGAGAAAGGCTATG |
| Circ-miR-940 wt | GATCTCACTGACAGTACCTGCCTTGTGAAGATCATTAAT |
| Circ-miR-940 mut | GATCTCACTGACAGTAGGACGGATGTGAAGATCATTAAT |

| miRNA mimics | sequence (5’-3’) |
| --- | --- |
| Has-miR-940 | AAGGCAGGGCCCCCGCUCCCC |
| Has-miR-512-5p | CACUCAGCCUUGAGGGCACUUUC |
| Has-miR-766-3p | ACUCCAGCCCCACAGCCUCAGC |
| Has-miR-330-5p | UCUCUGGGCCUGUGUCUUAGGC |
| Has-miR-338-3p | UCCAGCAUCAGUGAUUUUGUUG |
| Has-miR-492 | AGGACCUGCGGGACAAGAUUCUU |
| Has-miR-515-5p | UUCUCCAAAAGAAAGCACUUUCUG |
| Has-miR-873 | GCAGGAACUUGUGAGUCUCCU |
| Has-miR-326 | CCUCUGGGCCCUUCCUCCAG |
| Has-miR-661 | UGCCUGGGUCUCUGGCCUGCGCGU |
| Control | UUCUCCGAACGUGUCACTUTT |

Table S4. Aberrantly expression of the circular RNA signatures between favorable and unfavorable group.

| CircRNA ID | circBase ID | Gene Name | Log FC | P Value |
| --- | --- | --- | --- | --- |
| chrM:1701-15411- | | JA429830 | -5.19 | 1.37E-19 |
| chr2:153001402-153004814- | hsa_circ_0117653 | STAM2 | -4.17 | 1.14E-12 |
| chr3:196094899-196129890- | hsa_circ_0068648 | UBXN7 | -4.12 | 4.22E-12 |
| chr8:103845284-103846975- | hsa_circ_0135372 | AZIN1 | -4.07 | 4.22E-12 |
| chr1:153791267-153792211- | hsa_circ_0110835 | GATAD2B | -4.02 | 3.18E-11 |
| chr2:135887565-135893502+ | hsa_circ_0008036 | RAB3GAP1 | -4.02 | 6.56E-12 |
| chr13:30826068-30857928- | hsa_circ_0100209 | KATNAL1 | -3.91 | 3.97E-11 |
| chr16:72984368-72994093- | hsa_circ_0003702 | ZFHX3 | -3.8 | 9.98E-11 |
| chr2:61605498-61622368- | hsa_circ_0120642 | USP34 | -3.8 | 1.44E-11 |
| chr11:5247942-5248197- | | HBB | -3.8 | 1.77E-15 |
| chr16:47531310-47596406+ | hsa_circ_0105444 | PHKB | -3.78 | 1.98E-12 |
| chr19:58372744-58379560+ | | ZNF587 | -3.77 | 2.55E-10 |
| chr13:41096690-41134997- | | FOXO1 | -3.75 | 9.84E-10 |
| chr5:70848943-70858347+ | hsa_circ_0129508 | BDP1 | -3.7 | 7.76E-11 |
| chr1:244715624-244724447+ | hsa_circ_0006080 | C1orf101 | -3.66 | 2.83E-09 |
| chr7:157864119-157864499- | | PTPRN2 | -3.66 | 9.98E-11 |
| chr8:143425327-143427253- | hsa_circ_0006060 | TSNARE1 | -3.63 | 7.16E-09 |
| chr22:22055265-22065197- | hsa_circ_0001208 | YPEL1 | -3.61 | 2.83E-10 |
| chr8:131092148-131104389- | hsa_circ_0005718 | ASAP1 | -3.55 | 4.64E-09 |
| chr1:1747195-1749314- | hsa_circ_0009360 | GNB1 | -3.54 | 2.55E-10 |
| chr11:63517463-63517670+ | hsa_circ_0022603 | RTN3 | -3.53 | 4.37E-10 |
| chr21:37736391-37744829+ | hsa_circ_0115949 | MORC3 | -3.53 | 1.44E-11 |
| chr3:10088264-10091189+ | hsa_circ_0064220 | FANCD2 | -3.51 | 1.44E-11 |
| chr1:246907378-246927659+ | hsa_circ_0112872 | SCCPDH | -3.5 | 6.00E-09 |
| chr6:71136273-71162274+ | hsa_circ_0132164 | FAM135A | -3.45 | 2.09E-08 |
| chr5:145197457-145205763- | hsa_circ_0006528 | PRELID2 | -3.45 | 2.15E-10 |
| chr2:231940225-231951895+ | hsa_circ_0001110 | PSMD1 | -3.45 | 4.87E-10 |
| chr16:53269097-53281415+ | hsa_circ_0105547 | CHD9 | -3.44 | 1.26E-08 |
| chr7:5939915-5940563+ | | CCZ1 | -3.43 | 3.48E-08 |
| chrM:1701-14055- | | JA429830 | -3.42 | 6.59E-10 |
| chr19:14153265-14153601+ | hsa_circ_0109143 | IL27RA | -3.4 | 1.27E-07 |
| chrM:1701-12394- | | JA429830 | -3.4 | 6.59E-10 |
| chr16:70566382-70573110+ | hsa_circ_0105873 | SF3B3 | -3.39 | 5.83E-08 |
| chr7:138957068-138958820+ | hsa_circ_0082648 | UBN2 | -3.37 | 9.81E-08 |
| chr2:71653590-71658558+ | hsa_circ_0120911 | ZNF638 | -3.36 | 1.66E-07 |
| chr4:185593327-185603490+ | hsa_circ_0125926 | PRIMPOL | -3.35 | 9.81E-08 |
| chr3:125266250-125271523- | | OSBPL11 | -3.31 | 1.66E-07 |
| chr2:24181171-24222780+ | hsa_circ_0119477 | UBXN2A | -3.31 | 2.81E-07 |
| chr1:245180544-245246990+ | hsa_circ_0112831 | EFCAB2 | -3.29 | 4.80E-07 |
| chr5:177649356-177652430- | hsa_circ_0075256 | PHYKPL | -3.28 | 5.23E-08 |
| chr6:32487251-32522570- | | HLA-DRB5 | -3.28 | 6.18E-10 |
| chr6:146214351-146216113- | hsa_circ_0078139 | SHPRH | -3.25 | 6.60E-08 |
| chr11:2991033-2993473- | hsa_circ_0020822 | NAP1L4 | -3.25 | 2.17E-11 |
| chrY:7209156-7239930+ | hsa_circ_0006322 | PRKY | -3.24 | 1.66E-07 |
| chr2:98165882-98166077- | | ANKRD36B | -3.23 | 2.57E-08 |
| chr11:62298022-62298231- | | AHNAK | -3.22 | 8.23E-07 |
| chr1:93142715-93170301- | hsa_circ_0114498 | EVI5 | -3.21 | 2.57E-08 |
| chr11:3789811-3794969- | hsa_circ_0020915 | NUP98 | -3.19 | 8.23E-07 |
| chr2:183998294-184024380+ | hsa_circ_0057301 | NUP35 | -3.18 | 4.80E-07 |
| chr7:139827242-139833456- | hsa_circ_0133555 | KDM7A | -3.18 | 4.80E-07 |
| chr10:103557737-103560157- | hsa_circ_0092436 | MGEA5 | -3.18 | 4.12E-08 |
| chr4:144336630-144361535+ | hsa_circ_0125422 | GAB1 | -3.14 | 6.66E-10 |
| chr22:32874968-32883815+ | hsa_circ_0062983 | FBXO7 | -3.14 | 1.06E-07 |
| chr1:91403042-91406866- | hsa_circ_0114427 | ZNF644 | -3.13 | 8.23E-07 |
| chr17:81006347-81006661- | hsa_circ_0000819 | B3GNTL1 | -3.13 | 3.48E-08 |
| chr9:86292642-86301070- | hsa_circ_0003715 | UBQLN1 | -3.13 | 1.85E-10 |
| chr14:91757339-91760662- | hsa_circ_0102879 | CCDC88C | -3.13 | 1.11E-09 |
| chr11:120343759-120348235+ | hsa_circ_0009021 | ARHGEF12 | -3.12 | 1.06E-07 |
| chr7:139083345-139094400+ | hsa_circ_0133534 | LUC7L2 | -3.11 | 4.28E-13 |
| chr3:23997528-24006653+ | | NR1D2 | -3.1 | 4.07E-09 |
| chr5:179134133-179137066+ | hsa_circ_0075298 | CANX | -3.09 | 2.78E-07 |
| chr8:39079129-39114862+ | hsa_circ_0084055 | ADAM32 | -3.08 | 1.42E-06 |
| chr4:1905943-1920350+ | hsa_circ_0003112 | WHSC1 | -3.08 | 1.68E-09 |
| chr15:50592986-50596330- | hsa_circ_0007983 | GABPB1 | -3.08 | 1.61E-08 |
| chr3:105542967-105572508- | hsa_circ_0121400 | CBLB | -3.08 | 2.18E-08 |
| chr9:336582-340321+ | | DOCK8 | -3.06 | 2.46E-06 |
| chr15:50288874-50311173- | | ATP8B4 | -3.05 | 1.66E-07 |
| chr7:65444386-65445396- | hsa_circ_0007622 | GUSB | -3.04 | 2.18E-08 |
| chr10:126176991-126186697+ | hsa_circ_0003766 | LHPP | -3.03 | 2.18E-08 |
| chr15:83481855-83488240+ | | WHAMM | -3 | 7.40E-07 |
| chr2:53941521-53943880- | hsa_circ_0003959 | ASB3 | -3 | 6.15E-12 |
| chr3:56597712-56601081+ | hsa_circ_0124281 | CCDC66 | -2.99 | 4.27E-06 |
| chr11:3147703-3150399- | | OSBPL5 | -2.98 | 1.42E-06 |
| chr12:22635491-22659753- | hsa_circ_0098155 | C2CD5 | -2.98 | 1.46E-09 |
| chrX:117528018-117540989+ | hsa_circ_0139626 | WDR44 | -2.95 | 4.12E-08 |
| chr16:88052034-88071617+ | hsa_circ_0040819 | BANP | -2.95 | 8.13E-08 |
| chr5:149921132-149925048+ | hsa_circ_0004261 | NDST1 | -2.94 | 2.46E-06 |
| chrX:40573030-40574472- | hsa_circ_0140272 | MED14 | -2.94 | 1.25E-05 |
| chr1:27432376-27436268- | hsa_circ_0007283 | SLC9A1 | -2.94 | 6.66E-10 |
| chr3:43616301-43618753- | hsa_circ_0065004 | ANO10 | -2.88 | 7.48E-06 |
| chr8:124346118-124351686- | hsa_circ_0085460 | ATAD2 | -2.88 | 6.22E-09 |
| chr13:32835753-32841496+ | hsa_circ_0029926 | FRY | -2.86 | 1.32E-05 |
| chr17:3717616-3719564- | hsa_circ_0041462 | C17orf85 | -2.85 | 6.76E-06 |
| chr12:27077288-27081838- | hsa_circ_0025694 | ASUN | -2.85 | 1.32E-05 |
| chr15:34544371-34547593- | hsa_circ_0103323 | SLC12A6 | -2.85 | 2.46E-06 |
| chr4:129873924-129925031- | hsa_circ_0125328 | SCLT1 | -2.84 | 3.29E-06 |
| chr2:48701822-48718309+ | hsa_circ_0008358 | PPP1R21 | -2.83 | 7.41E-05 |
| chr11:108186550-108188248+ | hsa_circ_0024230 | ATM | -2.83 | 5.23E-08 |
| chr7:129821463-129832663- | hsa_circ_0082352 | TMEM209 | -2.83 | 3.29E-06 |
| chr7:128622302-128633968- | hsa_circ_0003318 | TNPO3 | -2.83 | 1.32E-05 |
| chr5:179132680-179136060+ | hsa_circ_0075292 | CANX | -2.81 | 7.48E-06 |
| chr4:141446574-141464740+ | hsa_circ_0125400 | ELMOD2 | -2.81 | 1.32E-05 |
| chr8:124089351-124096580+ | hsa_circ_0085438 | TBC1D31 | -2.79 | 3.41E-12 |
| chr6:170110332-170121207- | hsa_circ_0078755 | PHF10 | -2.79 | 2.33E-05 |
| chr1:78097520-78107340- | hsa_circ_0012986 | ZZZ3 | -2.79 | 3.07E-06 |
| chr1:59002181-59004978- | | DAB1 | -2.79 | 1.25E-09 |
| chr7:87516159-87526654+ | | DBF4 | -2.78 | 7.48E-06 |
| chr4:57344545-57349436+ | hsa_circ_0126686 | SRP72 | -2.77 | 3.11E-07 |
| chr7:27824782-27825108+ | hsa_circ_0079668 | TAX1BP1 | -2.77 | 1.22E-06 |
| chr13:46090278-46099186+ | hsa_circ_0008937 | COG3 | -2.77 | 3.29E-06 |
| chr15:35182416-35189906- | hsa_circ_0103341 | AQR | -2.76 | 1.93E-06 |
| chr6:28239632-28240564+ | hsa_circ_0131616 | ZSCAN26 | -2.75 | 2.33E-05 |
| chr13:95813443-95818621- | hsa_circ_0007470 | ABCC4 | -2.75 | 1.93E-06 |
| chr4:48371866-48396670+ | hsa_circ_0126526 | SLAIN2 | -2.75 | 7.48E-06 |
| chr10:112640991-112650428+ | hsa_circ_0092663 | PDCD4 | -2.74 | 2.33E-05 |
| chr3:44871463-44881948+ | hsa_circ_0002912 | KIF15 | -2.74 | 4.14E-05 |
| chr3:11399892-11468400+ | hsa_circ_0008210 | ATG7 | -2.73 | 7.41E-05 |
| chr5:76342172-76342502+ | hsa_circ_0129665 | AGGF1 | -2.73 | 2.33E-05 |
| chr7:91924203-91974380+ | hsa_circ_0081009 | ANKIB1 | -2.73 | 1.92E-06 |
| chr1:114367764-114377061- | | PTPN22 | -2.72 | 4.91E-05 |
| chr1:186394255-186420406+ | hsa_circ_0111538 | LOC102724919 | -2.7 | 4.14E-05 |
| chr20:45891032-45905539- | hsa_circ_0007026 | ZMYND8 | -2.7 | 3.65E-09 |
| chr18:21946856-21957499- | hsa_circ_0108108 | OSBPL1A | -2.7 | 2.53E-05 |
| chr14:104053611-104056766+ | hsa_circ_0004965 | KLC1 | -2.69 | 4.14E-05 |
| chrX:3735586-3736744- | hsa_circ_0140252 | LOC389906 | -2.69 | 5.45E-06 |
| chr11:35747472-35747711+ | hsa_circ_0095778 | TRIM44 | -2.69 | 4.14E-05 |
| chr17:71231615-71233134+ | hsa_circ_0045537 | C17orf80 | -2.69 | 7.41E-05 |
| chr2:209209835-209212747+ | hsa_circ_0001097 | PIKFYVE | -2.69 | 7.98E-10 |
| chr15:72810408-72853890+ | hsa_circ_0000629 | ARIH1 | -2.67 | 7.41E-05 |
| chr2:32339707-32353548+ | hsa_circ_0053413 | SPAST | -2.66 | 7.41E-05 |
| chr6:17646298-17649531- | hsa_circ_0007370 | NUP153 | -2.66 | 3.55E-08 |
| chr13:77798586-77818086- | hsa_circ_0100910 | MYCBP2 | -2.66 | 1.93E-06 |
| chr10:76598441-76603236+ | hsa_circ_0005164 | KAT6B | -2.66 | 9.82E-11 |
| chr9:91943575-91943801+ | hsa_circ_0005432 | SECISBP2 | -2.65 | 7.41E-05 |
| chrY:14813939-14821476+ | hsa_circ_0140731 | USP9Y | -2.65 | 7.41E-05 |
| chr9:33953283-33956144- | hsa_circ_0003945 | UBAP2 | -2.65 | 9.99E-06 |
| chr18:29210922-29218723- | hsa_circ_0047346 | B4GALT6 | -2.64 | 4.14E-05 |
| chr1:40656444-40668286+ | hsa_circ_0007302 | RLF | -2.64 | 7.16E-05 |
| chr13:50025689-50065046+ | hsa_circ_0100672 | SETDB2 | -2.64 | 2.53E-05 |
| chrX:135814261-135829751- | hsa_circ_0139810 | ARHGEF6 | -2.64 | 7.41E-05 |
| chr1:172525009-172539870+ | hsa_circ_0111166 | SUCO | -2.63 | 6.88E-07 |
| chr4:170498079-170511960- | hsa_circ_0125768 | NEK1 | -2.63 | 3.11E-07 |
| chr5:96314842-96350799+ | | LNPEP | -2.63 | 4.25E-05 |
| chrM:15336-15491+ | | cytochrome b | -2.62 | 1.28E-06 |
| chr1:27087347-27089776+ | hsa_circ_0006913 | ARID1A | -2.62 | 7.41E-05 |
| chr12:123702923-123707674- | hsa_circ_0029200 | MPHOSPH9 | -2.61 | 7.41E-05 |
| chr2:230701564-230744844- | hsa_circ_0007381 | TRIP12 | -2.61 | 2.33E-05 |
| chr21:34787195-34793992+ | hsa_circ_0115844 | IFNGR2 | -2.61 | 1.93E-07 |
| chr1:40872408-40887786+ | hsa_circ_0113346 | SMAP2 | -2.61 | 4.27E-06 |
| chr9:14639894-14680160- | hsa_circ_0008952 | ZDHHC21 | -2.59 | 3.87E-06 |
| chr7:56049903-56062681+ | hsa_circ_0080251 | GBAS | -2.59 | 0.000133 |
| chr3:47098311-47139571- | hsa_circ_0065153 | SETD2 | -2.57 | 2.33E-05 |
| chr17:45438744-45492285+ | hsa_circ_0106984 | EFCAB13 | -2.57 | 1.93E-06 |
| chr10:7262373-7318951- | hsa_circ_0017627 | SFMBT2 | -2.57 | 9.05E-06 |
| chr3:17226601-17279911- | hsa_circ_0122730 | TBC1D5 | -2.56 | 3.24E-05 |
| chr10:32854486-32912745+ | hsa_circ_0093561 | CCDC7 | -2.55 | 7.41E-05 |
| chr3:172003716-172016577+ | hsa_circ_0002026 | FNDC3B | -2.55 | 0.000242 |
| chr5:52942063-52954454+ | hsa_circ_0129114 | NDUFS4 | -2.55 | 0.000121 |
| chr7:132973676-133002144+ | hsa_circ_0082439 | EXOC4 | -2.55 | 4.14E-05 |
| chr3:196807922-196846401- | hsa_circ_0007203 | DLG1 | -2.54 | 4.27E-06 |
| chr5:137281591-137290065- | hsa_circ_0128059 | FAM13B | -2.53 | 4.25E-05 |
| chr10:94268485-94297307- | hsa_circ_0094473 | IDE | -2.53 | 3.24E-05 |
| chr12:11273609-11281962- | hsa_circ_0097432 | PRB4 | -2.52 | 7.71E-07 |
| chrM:959-15403+ | | DQ582265 | -2.52 | 6.52E-06 |
| chr2:70441481-70444126- | hsa_circ_0055116 | TIA1 | -2.51 | 0.000133 |
| chr5:34922322-34923339+ | hsa_circ_0128896 | BRIX1 | -2.51 | 3.29E-06 |
| chr22:40666126-40669604+ | hsa_circ_0063408 | TNRC6B | -2.51 | 0.000242 |
| chr3:127801326-127806641- | hsa_circ_0001335 | RUVBL1 | -2.5 | 0.000133 |
| chr4:1902353-1941505+ | hsa_circ_0005881 | WHSC1 | -2.5 | 2.53E-09 |
| chr4:83795764-83803093- | hsa_circ_0003716 | SEC31A | -2.5 | 0.00044 |
| chr9:86293356-86301070- | hsa_circ_0087357 | UBQLN1 | -2.5 | 1.17E-11 |
| chr15:50330965-50339661- | hsa_circ_0035197 | ATP8B4 | -2.49 | 2.12E-06 |
| chr6:29913850-29977935+ | | HLA-J | -2.48 | 0.000242 |
| chr11:120916383-120924441+ | hsa_circ_0000365 | TBCEL | -2.48 | 0.000133 |
| chr17:65941525-65960520+ | hsa_circ_0045470 | BPTF | -2.48 | 0.000807 |
| chr7:131058469-131084192+ | hsa_circ_0003551 | MKLN1 | -2.47 | 0.000121 |
| chr1:98144651-98187227- | hsa_circ_0114604 | DPYD | -2.47 | 0.000242 |
| chr15:66007816-66031213- | hsa_circ_0035937 | DENND4A | -2.47 | 7.16E-05 |
| chr5:77684661-77755185+ | hsa_circ_0129697 | SCAMP1 | -2.46 | 0.000206 |
| chr3:119219542-119232566+ | hsa_circ_0006884 | TIMMDC1 | -2.46 | 9.87E-10 |
| chr16:11988811-11991892- | hsa_circ_0037911 | GSPT1 | -2.46 | 3.13E-09 |
| chr2:54114396-54120936- | hsa_circ_0120326 | PSME4 | -2.45 | 0.000121 |
| chr12:62892699-62895478+ | hsa_circ_0027364 | MON2 | -2.45 | 0.000242 |
| chr4:95494502-95507595+ | hsa_circ_0070467 | PDLIM5 | -2.45 | 4.37E-06 |
| chr9:17309056-17342442+ | hsa_circ_0138343 | CNTLN | -2.45 | 1.51E-05 |
| chr1:25666965-25679465+ | hsa_circ_0010927 | TMEM50A | -2.44 | 9.05E-06 |
| chr2:98192842-98197031- | | ANKRD36B | -2.44 | 0.000133 |
| chr1:193110980-193121574+ | hsa_circ_0111574 | CDC73 | -2.44 | 0.000188 |
| chrM:3323-3598+ | | TVAS5 | -2.44 | 0.000133 |
| chr1:246719875-246720836- | hsa_circ_0004005 | TFB2M | -2.44 | 0.00044 |
| chr16:31373157-31374074+ | hsa_circ_0039161 | ITGAX | -2.43 | 6.88E-07 |
| chr2:231624674-231658046+ | hsa_circ_0003898 | CAB39 | -2.43 | 0.000206 |
| chrM:959-15382+ | | DQ582265 | -2.43 | 1.80E-08 |
| chr5:112321532-112337371+ | hsa_circ_0073608 | DCP2 | -2.43 | 2.74E-07 |
| chr1:225140372-225195246+ | hsa_circ_0016601 | DNAH14 | -2.43 | 1.25E-05 |
| chr15:66044717-66048810- | hsa_circ_0035956 | DENND4A | -2.43 | 9.98E-08 |
| chr18:9570155-9577189- | hsa_circ_0007808 | PPP4R1 | -2.42 | 6.00E-06 |
| chr10:7409611-7412337- | hsa_circ_0017647 | SFMBT2 | -2.42 | 0.00044 |
| chr22:42912017-42973104- | hsa_circ_0001242 | RRP7A | -2.42 | 7.16E-05 |
| chr9:74522737-74523046- | | ABHD17B | -2.41 | 5.23E-05 |
| chr1:63944435-63955889- | hsa_circ_0113868 | ITGB3BP | -2.41 | 0.00044 |
| chr8:54682178-54714456- | hsa_circ_0136756 | ATP6V1H | -2.38 | 2.01E-05 |
| chr6:144858718-144864006+ | hsa_circ_0130908 | UTRN | -2.38 | 1.32E-08 |
| chr6:117013218-117047785+ | hsa_circ_0006146 | KPNA5 | -2.38 | 0.00035 |
| chr14:31626046-31626509- | hsa_circ_0101614 | HECTD1 | -2.38 | 0.000121 |
| chr9:33971649-33996331- | hsa_circ_0086730 | UBAP2 | -2.38 | 7.16E-05 |
| chr2:203818728-203820481+ | hsa_circ_0003493 | CARF | -2.38 | 0.000121 |
| chr1:93677658-93698150+ | hsa_circ_0114519 | CCDC18 | -2.38 | 0.00044 |
| chr8:18725184-18730243- | hsa_circ_0136098 | PSD3 | -2.38 | 0.000121 |
| chr11:77333545-77336863- | hsa_circ_0008790 | CLNS1A | -2.37 | 0.000807 |
| chr15:44788578-44789336+ | hsa_circ_0007705 | CTDSPL2 | -2.37 | 0.000121 |
| chr15:41339606-41348904- | hsa_circ_0034662 | INO80 | -2.37 | 2.53E-05 |
| chr15:43086842-43109295- | hsa_circ_0003838 | TTBK2 | -2.37 | 6.88E-07 |
| chr8:71495399-71510490- | hsa_circ_0084748 | TRAM1 | -2.37 | 0.000206 |
| chr2:32858943-32875300+ | hsa_circ_0053865 | TTC27 | -2.36 | 0.000121 |
| chr17:61829300-61831878- | hsa_circ_0107415 | CCDC47 | -2.36 | 0.00044 |
| chr18:2732263-2752550+ | hsa_circ_0108174 | SMCHD1 | -2.35 | 0.000206 |
| chr22:30374431-30387659+ | hsa_circ_0002954 | MTMR3 | -2.34 | 0.00044 |
| chr19:9763629-9764554- | | ZNF562 | -2.34 | 0.001489 |
| chr4:154315414-154318485+ | hsa_circ_0006225 | MND1 | -2.34 | 0.000206 |
| chr2:45670976-45671466- | | SRBD1 | -2.34 | 0.00044 |
| chr2:61710092-61720188- | hsa_circ_0120656 | XPO1 | -2.34 | 3.24E-05 |
| chr12:129293333-129299615- | hsa_circ_0006689 | SLC15A4 | -2.34 | 7.71E-07 |
| chr21:46275125-46281186- | hsa_circ_0001200 | PTTG1IP | -2.33 | 3.54E-07 |
| chr4:88084710-88116842- | hsa_circ_0127236 | KLHL8 | -2.33 | 0.00035 |
| chr7:30395344-30451689+ | | ZNRF2 | -2.33 | 0.00043 |
| chr5:177046079-177059739- | hsa_circ_0004138 | LOC202181 | -2.33 | 0.000242 |
| chr2:68364431-68374699- | hsa_circ_0120794 | WDR92 | -2.32 | 0.000206 |
| chr1:168007609-168037701+ | hsa_circ_0015132 | DCAF6 | -2.32 | 0.00044 |
| chr10:15875629-15885271- | hsa_circ_0005825 | FAM188A | -2.32 | 1.45E-07 |
| chr12:110820648-110834257- | hsa_circ_0028190 | ANAPC7 | -2.32 | 0.000807 |
| chr6:31777876-31779762- | | HSPA1L | -2.31 | 3.30E-05 |
| chr2:61709515-61720188- | hsa_circ_0120651 | XPO1 | -2.31 | 0.00044 |
| chr1:21267984-21276601- | | EIF4G3 | -2.31 | 0.000807 |
| chr1:15860732-15863309+ | hsa_circ_0000018 | DNAJC16 | -2.3 | 0.000807 |
| chr13:42385361-42393522- | hsa_circ_0004711 | VWA8 | -2.3 | 5.70E-07 |
| chrM:959-15403- | | JA429830 | -2.3 | 2.33E-05 |
| chr8:43013718-43028886+ | hsa_circ_0136638 | HGSNAT | -2.29 | 0.001489 |
| chr1:35879573-35881315+ | hsa_circ_0004709 | ZMYM4 | -2.29 | 1.15E-07 |
| chr3:183465460-183480067+ | hsa_circ_0122973 | YEATS2 | -2.29 | 0.000807 |
| chr2:217005909-217026771+ | hsa_circ_0058175 | XRCC5 | -2.29 | 0.000363 |
| chr12:100491143-100492319- | hsa_circ_0027851 | UHRF1BP1L | -2.29 | 0.00035 |
| chr1:161748034-161772062+ | hsa_circ_0007360 | ATF6 | -2.28 | 1.28E-06 |
| chr2:26505713-26505919+ | hsa_circ_0119637 | HADHB | -2.28 | 0.001489 |
| chr11:14852244-14882912+ | hsa_circ_0095427 | PDE3B | -2.28 | 5.49E-05 |
| chr3:171944661-171969331+ | hsa_circ_0067985 | FNDC3B | -2.28 | 0.000121 |
| chr4:57344545-57351030+ | hsa_circ_0126687 | SRP72 | -2.27 | 0.004141 |
| chr7:72604106-72607065+ | hsa_circ_0007927 | GTF2IP4 | -2.27 | 0.001489 |
| chr7:38282029-38289173- | | TRGC2 | -2.27 | 7.16E-05 |
| chr4:24556348-24572470- | | DHX15 | -2.26 | 0.000597 |
| chr9:102814699-102822454- | hsa_circ_0137538 | ERP44 | -2.26 | 9.99E-06 |
| chr20:13550154-13568017- | hsa_circ_0114699 | TASP1 | -2.26 | 0.001489 |
| chr12:110456150-110467440+ | | ANKRD13A | -2.25 | 0.001023 |
| chr10:11971864-11994248- | hsa_circ_0092831 | UPF2 | -2.25 | 0.001489 |
| chr2:61552510-61561106- | hsa_circ_0120625 | USP34 | -2.24 | 0.001489 |
| chr3:196541322-196545027+ | hsa_circ_0123230 | PAK2 | -2.24 | 1.93E-06 |
| chr13:28830429-28855516+ | hsa_circ_0029853 | PAN3 | -2.24 | 6.32E-06 |
| chr1:230798887-230807386+ | hsa_circ_0016867 | COG2 | -2.24 | 0.001023 |
| chr2:20454624-20455931- | hsa_circ_0005952 | PUM2 | -2.24 | 0.000597 |
| chr1:42730786-42789498- | hsa_circ_0113380 | FOXJ3 | -2.24 | 0.000597 |
| chr4:125584440-125600060- | hsa_circ_0125239 | G062788 | -2.24 | 0.001489 |
| chr7:157864299-157864499- | | PTPRN2 | -2.24 | 7.41E-05 |
| chr4:48686690-48712715- | hsa_circ_0002021 | FRYL | -2.23 | 1.45E-05 |
| chr1:41651793-41660071- | hsa_circ_0005303 | SCMH1 | -2.23 | 0.00035 |
| chr6:107515026-107519102- | hsa_circ_0130184 | PDSS2 | -2.23 | 0.000807 |
| chr8:142146608-142148236+ | hsa_circ_0135933 | DENND3 | -2.23 | 0.001489 |
| chr2:172782047-172809519+ | hsa_circ_0008032 | HAT1 | -2.22 | 1.86E-07 |
| chr2:172305191-172314585+ | hsa_circ_0005904 | DCAF17 | -2.22 | 0.000206 |
| chr17:80721841-80730383+ | hsa_circ_0005281 | TBCD | -2.22 | 0.000242 |
| chr7:73752778-73753334+ | hsa_circ_0001717 | CLIP2 | -2.22 | 0.000807 |
| chr19:18285850-18286507+ | hsa_circ_0005571 | IFI30 | -2.22 | 2.22E-06 |
| chr1:236969444-236979843+ | hsa_circ_0004039 | MTR | -2.22 | 5.70E-07 |
| chr14:31348017-31348691+ | hsa_circ_0031432 | COCH | -2.22 | 0.000121 |
| chr2:61709515-61717911- | hsa_circ_0054853 | XPO1 | -2.22 | 8.34E-09 |
| chr8:74585342-74621397- | hsa_circ_0006915 | STAU2 | -2.22 | 0.00035 |
| chr4:83742190-83750211- | hsa_circ_0127075 | SEC31A | -2.21 | 0.001023 |
| chr17:27993885-28030080- | | SSH2 | -2.21 | 4.65E-06 |
| chr2:170411634-170413807- | hsa_circ_0002377 | FASTKD1 | -2.21 | 9.05E-06 |
| chr19:4408900-4409756+ | hsa_circ_0048607 | CHAF1A | -2.21 | 0.000121 |
| chr5:139889222-139889766+ | hsa_circ_0001542 | ANKHD1 | -2.21 | 0.001489 |
| chr2:61389977-61391675+ | hsa_circ_0005338 | C2orf74 | -2.21 | 0.001023 |
| chr5:156766065-156768165+ | hsa_circ_0128436 | CYFIP2 | -2.2 | 0.001023 |
| chr18:13029662-13042333+ | | CEP192 | -2.2 | 0.00044 |
| chr7:151927008-151935911- | hsa_circ_0008206 | KMT2C | -2.19 | 0.001489 |
| chr1:1735858-1737977- | hsa_circ_0000007 | GNB1 | -2.19 | 4.42E-05 |
| chr5:176671196-176675325+ | hsa_circ_0004473 | NSD1 | -2.19 | 0.000597 |
| chr5:86627165-86633908+ | hsa_circ_0007507 | RASA1 | -2.19 | 0.003022 |
| chr19:58372744-58373938+ | | ZNF587 | -2.19 | 0.000135 |
| chrX:1401571-1409402+ | hsa_circ_0006648 | CSF2RA | -2.19 | 0.000597 |
| chr1:120572529-120594393- | hsa_circ_0002128 | NOTCH2 | -2.19 | 0.002766 |
| chr2:15693550-15698758- | hsa_circ_0005556 | NBAS | -2.19 | 0.001489 |
| chrM:1709-14377- | | JA429830 | -2.18 | 0.000133 |
| chr14:51208300-51219451- | hsa_circ_0102064 | NIN | -2.18 | 0.000807 |
| chr2:98430434-98435184- | hsa_circ_0121254 | TMEM131 | -2.18 | 0.001756 |
| chr20:47691322-47707559+ | hsa_circ_0001168 | CSE1L | -2.18 | 5.49E-05 |
| chr14:92555074-92563182- | hsa_circ_0102918 | ATXN3 | -2.18 | 0.002766 |
| chr2:85262139-85262970+ | hsa_circ_0121085 | KCMF1 | -2.18 | 0.001489 |
| chr9:5021963-5055788+ | hsa_circ_0008571 | JAK2 | -2.17 | 0.000206 |
| chr3:183361268-183382827+ | hsa_circ_0122951 | KLHL24 | -2.17 | 0.003022 |
| chr14:57740962-57747140+ | hsa_circ_0032052 | AP5M1 | -2.17 | 0.001023 |
| chrM:1696-3571+ | | TVAS5 | -2.16 | 0.001489 |
| chr14:45475211-45495075+ | hsa_circ_0031755 | FAM179B | -2.16 | 0.001489 |
| chr15:44941064-44952814- | hsa_circ_0035104 | SPG11 | -2.16 | 0.002766 |
| chr11:63960550-63965446+ | hsa_circ_0022631 | STIP1 | -2.16 | 4.25E-05 |
| chr2:10059170-10059949+ | hsa_circ_0008204 | TAF1B | -2.15 | 0.001489 |
| chr1:197552282-197581106- | hsa_circ_0015778 | DENND1B | -2.14 | 0.002766 |
| chr2:215609791-215646233- | hsa_circ_0058042 | BARD1 | -2.14 | 0.003022 |
| chr2:32117061-32157204- | hsa_circ_0003001 | MEMO1 | -2.14 | 0.002766 |
| chr9:86297866-86301070- | hsa_circ_0005142 | UBQLN1 | -2.14 | 5.68E-07 |
| chr2:43625110-43657441- | hsa_circ_0054303 | THADA | -2.13 | 0.002766 |
| chr19:19703315-19710174- | hsa_circ_0109271 | PBX4 | -2.13 | 0.000206 |
| chr7:37250991-37264635- | hsa_circ_0134243 | ELMO1 | -2.13 | 0.001023 |
| chrM:13887-14055- | | JA760602 | -2.13 | 0.00145 |
| chr1:23376880-23385660+ | hsa_circ_0003889 | KDM1A | -2.13 | 3.54E-07 |
| chr10:70246901-70253327- | hsa_circ_0003459 | SLC25A16 | -2.13 | 0.00517 |
| chr7:152480272-152498816+ | hsa_circ_0006890 | ACTR3B | -2.13 | 0.002578 |
| chr5:94204038-94248681- | hsa_circ_0005540 | MCTP1 | -2.13 | 0.003022 |
| chr17:60106902-60111345- | hsa_circ_0107373 | MED13 | -2.13 | 4.25E-05 |
| chr6:42571326-42574389+ | hsa_circ_0003177 | UBR2 | -2.12 | 2.90E-06 |
| chrX:117700537-117733191+ | hsa_circ_0139642 | DOCK11 | -2.12 | 7.16E-05 |
| chr1:58999621-59004978- | hsa_circ_0113708 | DAB1 | -2.12 | 0.002766 |
| chr15:32815230-32825569- | | XLOC_l2_004867 | -2.12 | 2.08E-05 |
| chr6:86227474-86251761- | hsa_circ_0077237 | SNX14 | -2.11 | 0.00517 |
| chr3:169854207-169896726- | hsa_circ_0002622 | PHC3 | -2.11 | 1.11E-05 |
| chr12:97303530-97313903+ | hsa_circ_0099620 | NEDD1 | -2.11 | 0.001489 |
| chr10:98667022-98711953+ | hsa_circ_0019316 | LCOR | -2.11 | 0.000223 |
| chr5:177058367-177059739- | | LOC202181 | -2.1 | 0.001023 |
| chr11:32611093-32617593+ | hsa_circ_0003119 | EIF3M | -2.09 | 0.003657 |
| chr6:110036281-110064975+ | hsa_circ_0077607 | FIG4 | -2.09 | 0.002766 |
| chr1:145646115-145663367+ | hsa_circ_0110705 | RNF115 | -2.09 | 6.00E-06 |
| chr1:145227041-145248894+ | hsa_circ_0002262 | NOTCH2NL | -2.09 | 0.002766 |
| chr2:169547544-169574488+ | hsa_circ_0007024 | CERS6 | -2.09 | 5.49E-05 |
| chr20:35695127-35696589- | hsa_circ_0060238 | RBL1 | -2.08 | 3.51E-05 |
| chr11:68363565-68370960+ | hsa_circ_0008391 | PPP6R3 | -2.08 | 0.001489 |
| chr17:45678974-45691091+ | hsa_circ_0002347 | NPEPPS | -2.08 | 0.002766 |
| chr14:31067689-31071337+ | hsa_circ_0101533 | G2E3 | -2.08 | 0.002766 |
| chr3:150280329-150286079+ | hsa_circ_0067729 | EIF2A | -2.08 | 0.00517 |
| chr13:77791975-77807398- | hsa_circ_0030511 | MYCBP2 | -2.08 | 0.000807 |
| chr6:34574332-34614575- | hsa_circ_0009095 | C6orf106 | -2.07 | 9.31E-06 |
| chr8:95839491-95844402+ | hsa_circ_0084941 | INTS8 | -2.07 | 9.82E-06 |
| chr13:41207284-41219623- | | FOXO1 | -2.07 | 0.002766 |
| chr2:190656516-190682906+ | hsa_circ_0001083 | PMS1 | -2.07 | 6.17E-06 |
| chr1:8601273-8617582- | hsa_circ_0002158 | RERE | -2.07 | 0.000123 |
| chr5:50045985-50093067+ | hsa_circ_0072431 | PARP8 | -2.07 | 0.001577 |
| chr4:76725287-76726456+ | hsa_circ_0008757 | USO1 | -2.06 | 0.002766 |
| chr2:32889370-32903989+ | hsa_circ_0053875 | TTC27 | -2.06 | 0.000242 |
| chr18:45394694-45423180- | hsa_circ_0047612 | SMAD2 | -2.06 | 0.002766 |
| chr17:61838274-61843554- | hsa_circ_0045220 | CCDC47 | -2.06 | 2.16E-06 |
| chr5:38967263-38968132- | hsa_circ_0072329 | RICTOR | -2.06 | 0.002766 |
| chr2:15601325-15651474- | hsa_circ_0052767 | NBAS | -2.06 | 0.001756 |
| chr1:235840391-235860572- | hsa_circ_0017077 | LYST | -2.06 | 4.65E-06 |
| chr1:35936464-35944813- | hsa_circ_0011571 | KIAA0319L | -2.05 | 0.000362 |
| chr1:52301803-52306186- | | NRD1 | -2.05 | 0.001756 |
| chr7:99952766-99957160+ | hsa_circ_0007821 | STAG3L5P-PVRIG2P-PILRB | -2.05 | 0.001756 |
| chr4:17816476-17816981+ | hsa_circ_0001395 | NCAPG | -2.05 | 1.42E-07 |
| chr6:35586873-35648199- | | FKBP5 | -2.05 | 9.99E-06 |
| chr8:108306164-108359325- | hsa_circ_0135455 | ANGPT1 | -2.04 | 5.49E-05 |
| chr14:91808724-91810001- | hsa_circ_0032959 | CCDC88C | -2.04 | 0.003022 |
| chr9:34241183-34242106+ | hsa_circ_0006212 | UBAP1 | -2.03 | 0.002766 |
| chr4:107216251-107230146- | hsa_circ_0003673 | TBCK | -2.03 | 0.001577 |
| chr3:98568305-98600611- | hsa_circ_0066631 | DCBLD2 | -2.03 | 2.25E-05 |
| chr12:56742313-56743420- | hsa_circ_0008085 | STAT2 | -2.03 | 0.003022 |
| chr2:172782047-172823470+ | hsa_circ_0057072 | HAT1 | -2.03 | 0.002578 |
| chr18:2738395-2739518+ | hsa_circ_0003207 | SMCHD1 | -2.03 | 0.000462 |
| chrM:1701-15335- | | JA429830 | -2.02 | 0.000242 |
| chr2:61512010-61522425- | hsa_circ_0120606 | USP34 | -2.02 | 8.10E-05 |
| chr7:158566032-158590767- | hsa_circ_0133845 | ESYT2 | -2.02 | 1.73E-05 |
| chr1:89414774-89435150- | | CCBL2 | -2.02 | 0.00517 |
| chr4:88084631-88116842- | | KLHL8 | -2.02 | 0.00517 |
| chr9:3647338-3651867+ | hsa_circ_0138738 | RP11-509J21.1 | -2.02 | 0.005209 |
| chr14:51710574-51716483+ | hsa_circ_0009104 | TMX1 | -2.02 | 0.00517 |
| chr14:35331250-35331528- | hsa_circ_0006137 | BAZ1A | -2.02 | 1.39E-07 |
| chr14:70170112-70171459+ | hsa_circ_0032353 | SUSD6 | -2.01 | 0.001577 |
| chr5:179007948-179020646+ | hsa_circ_0128714 | RUFY1 | -2.01 | 0.000807 |
| chr14:45414233-45415131- | hsa_circ_0031754 | KLHL28 | -2.01 | 0.000807 |
| chr9:370230-372286+ | hsa_circ_0086193 | DOCK8 | -2 | 6.46E-05 |
| chr3:131186935-131190123- | hsa_circ_0004559 | MRPL3 | -2 | 0.00517 |
| chr2:197653928-197657876- | hsa_circ_0057602 | GTF3C3 | -2 | 0.001577 |
| chr1:150933039-150934634+ | hsa_circ_0005995 | SETDB1 | -2 | 0.003022 |
| chr6:90564536-90566918+ | hsa_circ_0132666 | CASP8AP2 | -2 | 0.003022 |
| chr8:11162350-11177347+ | hsa_circ_0083335 | MTMR9 | -2 | 0.01941 |
| chr17:70732789-70735041- | | SLC39A11 | -2 | 0.000807 |
| chr2:201798599-201802689- | hsa_circ_0118619 | ORC2 | -2 | 0.009732 |
| chr4:79747191-79782622+ | hsa_circ_0127024 | BMP2K | -2 | 0.00044 |
| chr7:98563319-98569568+ | hsa_circ_0081311 | TRRAP | -2 | 2.08E-05 |
| chr13:21179174-21199988+ | hsa_circ_0100027 | IFT88 | -1.99 | 0.001489 |
| chr6:29910550-29974592+ | | HLA-G | -1.99 | 0.00044 |
| chr11:34104372-34111003+ | hsa_circ_0021639 | CAPRIN1 | -1.99 | 0.001788 |
| chr7:129665998-129688984- | hsa_circ_0007735 | ZC3HC1 | -1.99 | 0.005209 |
| chr7:148516070-148516779- | hsa_circ_0133657 | EZH2 | -1.99 | 0.002766 |
| chr15:65773853-65780156- | hsa_circ_0104287 | DPP8 | -1.99 | 0.002766 |
| chr18:20570900-20576425+ | hsa_circ_0047135 | RBBP8 | -1.99 | 0.000597 |
| chr6:76412361-76421132+ | hsa_circ_0077096 | SENP6 | -1.99 | 3.03E-05 |
| chr18:29210922-29238052- | hsa_circ_0047347 | B4GALT6 | -1.99 | 0.00517 |
| chr2:20478344-20527139- | hsa_circ_0052852 | PUM2 | -1.98 | 6.00E-06 |
| chr9:130206308-130207528+ | hsa_circ_0006984 | ZNF79 | -1.98 | 5.75E-05 |
| chr14:92592466-92609638+ | hsa_circ_0102923 | CPSF2 | -1.98 | 0.002766 |
| chr1:33311303-33318767+ | | S100PBP | -1.98 | 0.00517 |
| chr2:198388348-198400354+ | hsa_circ_0057680 | MOB4 | -1.98 | 0.00517 |
| chr13:45578440-45594563+ | hsa_circ_0100568 | GPALPP1 | -1.98 | 0.00517 |
| chr4:83375875-83378191+ | hsa_circ_0003451 | ENOPH1 | -1.97 | 0.00517 |
| chrM:1680-8899+ | | TVAS5 | -1.97 | 0.000742 |
| chr5:171479933-171488272- | | STK10 | -1.96 | 0.001756 |
| chr2:165548731-165552346- | hsa_circ_0117890 | COBLL1 | -1.96 | 0.000137 |
| chrX:44935942-44950109+ | hsa_circ_0140322 | KDM6A | -1.96 | 0.00517 |
| chr10:5827815-5842668- | hsa_circ_0003351 | GDI2 | -1.96 | 0.000242 |
| chr6:86267694-86277295- | hsa_circ_0008991 | SNX14 | -1.96 | 0.003022 |
| chr15:66030045-66031213- | hsa_circ_0035949 | DENND4A | -1.96 | 0.000223 |
| chr1:78183552-78184326- | hsa_circ_0012992 | USP33 | -1.96 | 0.00517 |
| chr3:185318553-185331196+ | hsa_circ_0123061 | SENP2 | -1.96 | 0.003022 |
| chr21:40581921-40601362- | hsa_circ_0116017 | BRWD1 | -1.96 | 0.00517 |
| chr6:86216951-86246642- | | SNX14 | -1.95 | 0.003022 |
| chr2:99802640-99812219+ | hsa_circ_0001051 | MRPL30 | -1.95 | 0.000823 |
| chr22:37328807-37330036+ | hsa_circ_0063179 | CSF2RB | -1.95 | 0.00517 |
| chr1:169256541-169293738- | | NME7 | -1.95 | 0.000363 |
| chr17:60106902-60140583- | hsa_circ_0107374 | MED13 | -1.95 | 0.00044 |
| chr9:134518626-134526336- | hsa_circ_0089254 | RAPGEF1 | -1.95 | 0.003022 |
| chr7:74615371-74618329- | hsa_circ_0005908 | GTF2IP1 | -1.95 | 0.005209 |
| chr1:52961099-52975384- | hsa_circ_0113602 | ZCCHC11 | -1.95 | 0.005209 |
| chr7:33057051-33075600- | hsa_circ_0079799 | NT5C3A | -1.94 | 0.018448 |
| chr7:30494753-30496658- | hsa_circ_0079701 | NOD1 | -1.94 | 0.00517 |
| chr12:27867713-27878600+ | hsa_circ_0098280 | MRPS35 | -1.94 | 0.00517 |
| chr1:145640883-145650540+ | hsa_circ_0110703 | NBPF10 | -1.94 | 0.001023 |
| chr2:219204506-219206867+ | hsa_circ_0058230 | PNKD | -1.94 | 0.006889 |
| chr8:93929157-93941802- | hsa_circ_0004561 | TRIQK | -1.93 | 0.003022 |
| chr12:90013776-90029028- | hsa_circ_0099456 | ATP2B1 | -1.93 | 6.76E-06 |
| chr6:32522396-32549262- | | HLA-DRB5 | -1.93 | 0.003022 |
| chr2:20507739-20532824- | hsa_circ_0004836 | PUM2 | -1.93 | 0.001756 |
| chr2:74273405-74275538+ | hsa_circ_0006251 | TET3 | -1.93 | 0.000594 |
| chr1:111434014-111435155+ | hsa_circ_0000105 | CD53 | -1.93 | 0.000294 |
| chr6:5404775-5431405+ | hsa_circ_0075533 | FARS2 | -1.92 | 8.10E-05 |
| chr12:28408514-28460682+ | hsa_circ_0098286 | CCDC91 | -1.92 | 0.000163 |
| chr21:38792601-38853128+ | hsa_circ_0006832 | DYRK1A | -1.92 | 0.00517 |
| chr5:130987495-131013565- | hsa_circ_0073854 | FNIP1 | -1.91 | 0.009732 |
| chr11:119002245-119003929+ | hsa_circ_0006932 | HINFP | -1.91 | 0.009732 |
| chr6:71242863-71248104+ | hsa_circ_0132180 | FAM135A | -1.91 | 0.005209 |
| chr15:50294350-50339661- | | ATP8B4 | -1.91 | 0.001756 |
| chr5:49694941-49724061- | hsa_circ_0129104 | EMB | -1.91 | 0.005209 |
| chr9:36369716-36376124- | hsa_circ_0087023 | RNF38 | -1.91 | 0.004215 |
| chr17:45695716-45696530+ | hsa_circ_0004622 | NPEPPS | -1.91 | 0.001577 |
| chr15:94899366-94928754+ | | MCTP2 | -1.91 | 2.16E-07 |
| chrM:1692-3790+ | | TVAS5 | -1.9 | 0.002039 |
| chr15:68438154-68466230+ | hsa_circ_0104355 | PIAS1 | -1.9 | 0.001756 |
| chr10:11984664-11990507- | | UPF2 | -1.9 | 0.005209 |
| chr15:59323003-59381958+ | | RNF111 | -1.9 | 0.003022 |
| chr8:68007528-68018210+ | hsa_circ_0136956 | CSPP1 | -1.9 | 0.008992 |
| chr15:65268813-65275931- | hsa_circ_0035873 | SPG21 | -1.9 | 0.004215 |
| chr20:2967411-2969120+ | hsa_circ_0001127 | PTPRA | -1.9 | 0.000965 |
| chr14:55817099-55821940+ | hsa_circ_0102165 | GSE61474_XLOC_018327 | -1.9 | 0.008992 |
| chr12:111990084-111993723- | hsa_circ_0002457 | ATXN2 | -1.9 | 4.20E-07 |
| chr8:67740889-67748309+ | hsa_circ_0084652 | SGK3 | -1.89 | 0.00517 |
| chr11:63961661-63965446+ | hsa_circ_0096090 | STIP1 | -1.89 | 0.00517 |
| chr1:169272383-169293738- | | NME7 | -1.89 | 0.000212 |
| chr11:76207259-76239510+ | hsa_circ_0007583 | C11orf30 | -1.89 | 0.009732 |
| chr5:176618885-176631293+ | hsa_circ_0075157 | NSD1 | -1.89 | 0.002039 |
| chr4:25848916-25849486- | hsa_circ_0126072 | SEL1L3 | -1.89 | 0.005209 |
| chr1:114391162-114397671- | hsa_circ_0000111 | PTPN22 | -1.89 | 4.83E-05 |
| chr22:26000344-26040632+ | hsa_circ_0062647 | ADRBK2 | -1.89 | 0.005209 |
| chr12:26568236-26596583- | hsa_circ_0025648 | ITPR2 | -1.89 | 0.00517 |
| chr14:34993903-35005481- | hsa_circ_0101695 | EAPP | -1.89 | 0.009732 |
| chr8:141357856-141370292- | hsa_circ_0001826 | TRAPPC9 | -1.88 | 0.000523 |
| chr17:38318006-38319154+ | hsa_circ_0043509 | CASC3 | -1.88 | 0.009732 |
| chr15:55621922-55626205+ | | PIGB | -1.88 | 0.009732 |
| chr4:38091553-38119813+ | hsa_circ_0007486 | TBC1D1 | -1.88 | 0.012401 |
| chr20:45855946-45865260- | hsa_circ_0115299 | ZMYND8 | -1.88 | 0.002578 |
| chr5:55256230-55264224- | hsa_circ_0007304 | IL6ST | -1.88 | 0.009732 |
| chr22:24509533-24530382+ | hsa_circ_0062593 | CABIN1 | -1.88 | 0.002766 |
| chr17:76388557-76394432+ | hsa_circ_0107717 | PGS1 | -1.87 | 0.005209 |
| chr20:6011931-6012726+ | hsa_circ_0059409 | CRLS1 | -1.87 | 0.009732 |
| chr10:7811179-7822270- | hsa_circ_0002990 | KIN | -1.87 | 0.000965 |
| chr1:98144651-98165103- | hsa_circ_0004161 | DPYD | -1.87 | 6.46E-05 |
| chr17:80721841-80739597+ | hsa_circ_0000818 | TBCD | -1.87 | 0.000306 |
| chr20:17928130-17937681- | hsa_circ_0001977 | SNX5 | -1.87 | 0.000113 |
| chr6:15615498-15638035- | hsa_circ_0008418 | DTNBP1 | -1.87 | 0.006889 |
| chr15:50215576-50226379- | | ATP8B4 | -1.87 | 0.005209 |
| chr16:53878067-53922863+ | hsa_circ_0039398 | FTO | -1.87 | 0.009732 |
| chr6:32489682-32549615- | | HLA-DRB1 | -1.87 | 0.008992 |
| chr13:41400642-41411021- | hsa_circ_0030049 | TPTE2P5 | -1.86 | 0.018448 |
| chr10:104851321-104854212- | hsa_circ_0019779 | NT5C2 | -1.86 | 0.000123 |
| chr7:92881966-92905617+ | hsa_circ_0135110 | VPS50 | -1.86 | 0.000591 |
| chr7:102743498-102769239- | hsa_circ_0132837 | NAPEPLD | -1.86 | 0.00517 |
| chr7:92885821-92888944+ | | CCDC132 | -1.86 | 0.002766 |
| chr7:158586341-158590767- | hsa_circ_0133847 | ESYT2 | -1.86 | 0.003022 |
| chr4:139981478-139994721- | hsa_circ_0007137 | ELF2 | -1.86 | 0.000823 |
| chr9:88574708-88611492+ | hsa_circ_0139233 | NAA35 | -1.86 | 0.001756 |
| chr6:159001972-159029782+ | hsa_circ_0001662 | TMEM181 | -1.85 | 0.009732 |
| chr4:77051809-77065626- | hsa_circ_0070036 | NUP54 | -1.85 | 0.005209 |
| chr9:125760876-125777915+ | hsa_circ_0137865 | RABGAP1 | -1.85 | 0.002766 |
| chr16:89961446-89967202+ | hsa_circ_0002631 | TCF25 | -1.85 | 0.002766 |
| chr7:131060183-131084192+ | hsa_circ_0001746 | MKLN1 | -1.85 | 3.60E-08 |
| chr13:100181721-100190117+ | hsa_circ_0099700 | TM9SF2 | -1.85 | 0.00517 |
| chr7:90001469-90007525+ | hsa_circ_0007090 | GTPBP10 | -1.85 | 0.00517 |
| chr1:52959283-52962855- | hsa_circ_0113600 | ZCCHC11 | -1.85 | 0.011249 |
| chr12:51402259-51404549- | hsa_circ_0008802 | SLC11A2 | -1.85 | 0.000109 |
| chr3:167293706-167376114- | hsa_circ_0122623 | WDR49 | -1.84 | 0.001756 |
| chr14:50298769-50301167- | hsa_circ_0031814 | NEMF | -1.84 | 0.009732 |
| chr20:21306917-21338430+ | hsa_circ_0059580 | XRN2 | -1.84 | 0.009732 |
| chr16:81929412-81930687+ | | PLCG2 | -1.84 | 0.00517 |
| chr15:43044172-43045445- | | TTBK2 | -1.84 | 0.009732 |
| chr12:122332645-122341013+ | hsa_circ_0008670 | PSMD9 | -1.84 | 0.00044 |
| chr19:8612920-8613201- | hsa_circ_0110084 | MYO1F | -1.84 | 0.000672 |
| chr15:75703833-75705386- | hsa_circ_0036353 | SIN3A | -1.84 | 2.45E-08 |
| chr7:151478238-151483627- | | PRKAG2 | -1.83 | 0.009732 |
| chr10:103432672-103436193- | hsa_circ_0000255 | FBXW4 | -1.83 | 8.15E-06 |
| chr2:29124853-29140861+ | | WDR43 | -1.83 | 0.006889 |
| chr17:45405635-45422464+ | hsa_circ_0044241 | EFCAB13 | -1.83 | 0.018448 |
| chr7:77236552-77256992+ | hsa_circ_0080849 | PTPN12 | -1.83 | 0.000223 |
| chr15:32789690-32825569- | | WHAMMP1 | -1.82 | 0.005209 |
| chr5:88243625-88244032+ | hsa_circ_0129945 | MEF2C-AS1 | -1.82 | 0.0012 |
| chr3:136191255-136196254- | hsa_circ_0008750 | STAG1 | -1.82 | 0.001023 |
| chr17:29183973-29206505+ | | ATAD5 | -1.82 | 0.006889 |
| chr12:31850295-31862359- | hsa_circ_0098391 | AMN1 | -1.82 | 0.009732 |
| chr20:25000646-25004277- | hsa_circ_0114850 | ACSS1 | -1.82 | 0.00517 |
| chr3:12976948-12983365- | hsa_circ_0064388 | IQSEC1 | -1.81 | 0.008992 |
| chr20:61833639-61835159- | hsa_circ_0004858 | YTHDF1 | -1.81 | 0.000823 |
| chr3:48491443-48495818+ | hsa_circ_0065384 | ATRIP | -1.81 | 0.00517 |
| chr20:47567860-47569421+ | hsa_circ_0060664 | ARFGEF2 | -1.81 | 0.008992 |
| chr2:220431552-220433046- | hsa_circ_0007065 | OBSL1 | -1.81 | 0.018333 |
| chr20:25612794-25615463+ | hsa_circ_0059685 | ZNF337-AS1 | -1.8 | 0.026813 |
| chr3:196842798-196846401- | hsa_circ_0001383 | DLG1 | -1.8 | 0.001028 |
| chr2:32323867-32341281+ | hsa_circ_0119763 | SPAST | -1.8 | 0.005209 |
| chr18:74638993-74672811+ | hsa_circ_0005776 | ZNF236 | -1.8 | 0.008992 |
| chr5:132428392-132435344+ | hsa_circ_0127958 | HSPA4 | -1.79 | 0.000523 |
| chr2:179400459-179407088+ | hsa_circ_0004305 | TTN-AS1 | -1.79 | 0.000336 |
| chr15:73052748-73067438- | hsa_circ_0004033 | ADPGK | -1.79 | 0.018333 |
| chr1:169288397-169293738- | hsa_circ_0111101 | NME7 | -1.79 | 0.000772 |
| chr14:31585442-31590711- | hsa_circ_0031465 | HECTD1 | -1.79 | 0.026813 |
| chr19:8601136-8604912- | hsa_circ_0000886 | MYO1F | -1.79 | 0.009732 |
| chr5:177053437-177059739- | hsa_circ_0007932 | LOC202181 | -1.79 | 0.000223 |
| chr18:19236822-19239304- | hsa_circ_0047086 | ABHD3 | -1.79 | 0.001295 |
| chr6:118953616-118985684- | | CEP85L | -1.79 | 0.001756 |
| chr6:90461150-90472249- | hsa_circ_0003814 | MDN1 | -1.79 | 0.012401 |
| chr12:69983265-69987393+ | hsa_circ_0002940 | CCT2 | -1.78 | 1.32E-07 |
| chr6:56989532-57006893+ | hsa_circ_0007587 | ZNF451 | -1.78 | 0.003208 |
| chr3:179131200-179138715- | hsa_circ_0122823 | GNB4 | -1.78 | 0.000294 |
| chr3:31617888-31641951+ | hsa_circ_0003338 | STT3B | -1.77 | 0.011249 |
| chr5:50055477-50093067+ | hsa_circ_0072433 | PARP8 | -1.77 | 0.000123 |
| chr6:17665470-17669777- | hsa_circ_0007268 | NUP153 | -1.77 | 0.009732 |
| chr7:23643705-23651172+ | hsa_circ_0133979 | CCDC126 | -1.77 | 0.000188 |
| chr9:80537077-80546772- | hsa_circ_0139027 | GNAQ | -1.77 | 0.009732 |
| chr9:88190230-88248289- | hsa_circ_0139171 | AGTPBP1 | -1.77 | 2.56E-05 |
| chr12:938228-939110+ | hsa_circ_0005616 | WNK1 | -1.77 | 0.018448 |
| chr1:14104913-14109326+ | hsa_circ_0010029 | PRDM2 | -1.76 | 4.32E-05 |
| chr5:179020479-179021964+ | hsa_circ_0128717 | RUFY1 | -1.76 | 0.008992 |
| chr1:29313943-29314417+ | hsa_circ_0011167 | EPB41 | -1.76 | 0.018333 |
| chr14:53187579-53187908+ | | PSMC6 | -1.76 | 0.00517 |
| chr13:46090278-46093229+ | hsa_circ_0003401 | COG3 | -1.76 | 0.000158 |
| chr17:41456120-41456630- | hsa_circ_0106914 | LINC00910 | -1.76 | 0.001023 |
| chr10:12009340-12056183- | hsa_circ_0017711 | UPF2 | -1.76 | 0.004215 |
| chr10:27420788-27425314- | hsa_circ_0005125 | YME1L1 | -1.75 | 0.018109 |
| chr18:196637-199316+ | hsa_circ_0007706 | USP14 | -1.75 | 0.007916 |
| chr16:46705617-46710604- | hsa_circ_0039205 | VPS35 | -1.75 | 0.015529 |
| chr17:46925426-46926739+ | | CALCOCO2 | -1.75 | 0.035219 |
| chr2:135010666-135028121+ | hsa_circ_0002480 | MGAT5 | -1.75 | 0.000194 |
| chr13:45910671-45911523- | hsa_circ_0100578 | TPT1 | -1.75 | 0.026813 |
| chr2:24787164-24866991+ | hsa_circ_0119571 | NCOA1 | -1.75 | 0.001577 |
| chr4:89570991-89579642+ | hsa_circ_0006387 | HERC3 | -1.75 | 0.018448 |
| chr2:29129326-29137074+ | hsa_circ_0002025 | WDR43 | -1.75 | 0.026813 |
| chr10:7409611-7423911- | hsa_circ_0017648 | SFMBT2 | -1.74 | 0.001295 |
| chrX:72797243-72804408+ | hsa_circ_0091072 | CHIC1 | -1.74 | 0.000965 |
| chr1:10463128-10464336+ | hsa_circ_0110291 | PGD | -1.74 | 0.018448 |
| chr2:44021596-44037663+ | hsa_circ_0120109 | DYNC2LI1 | -1.74 | 0.018448 |
| chr3:133894453-133914026- | hsa_circ_0003633 | RYK | -1.74 | 1.23E-05 |
| chr17:61655831-61666599+ | hsa_circ_0107400 | DCAF7 | -1.74 | 0.018448 |
| chr17:54926057-54934026+ | hsa_circ_0003108 | DGKE | -1.74 | 0.035219 |
| chr6:150063526-150064851- | hsa_circ_0001652 | NUP43 | -1.74 | 0.018448 |
| chrM:1692-9159+ | | TVAS5 | -1.74 | 0.018448 |
| chr7:98564664-98569568+ | hsa_circ_0081314 | TRRAP | -1.74 | 0.015529 |
| chr18:51797730-51810383+ | hsa_circ_0047720 | POLI | -1.74 | 0.000706 |
| chr5:41794105-41807540- | hsa_circ_0004873 | OXCT1 | -1.74 | 0.015529 |
| chr3:150834125-150840761+ | hsa_circ_0122389 | MED12L | -1.73 | 0.00157 |
| chr22:29090020-29091861- | hsa_circ_0004811 | CHEK2 | -1.73 | 0.0012 |
| chr17:1703151-1704318- | hsa_circ_0004018 | SMYD4 | -1.73 | 0.015529 |
| chr2:29006773-29011675+ | hsa_circ_0007439 | PPP1CB | -1.73 | 3.19E-05 |
| chr21:34799191-34805178+ | hsa_circ_0008725 | IFNGR2 | -1.73 | 0.030141 |
| chr6:34949467-34951202+ | hsa_circ_0131646 | ANKS1A | -1.73 | 0.000965 |
| chr3:47079156-47108608- | hsa_circ_0004692 | SETD2 | -1.73 | 8.96E-06 |
| chr3:52946554-52947633- | hsa_circ_0066143 | SFMBT1 | -1.73 | 0.009732 |
| chr14:73445569-73460065- | hsa_circ_0102547 | ZFYVE1 | -1.73 | 0.00517 |
| chr7:76739255-76742800- | hsa_circ_0006338 | FAM185BP | -1.72 | 6.08E-05 |
| chr5:179976931-179980471+ | hsa_circ_0008836 | CNOT6 | -1.72 | 0.000116 |
| chr17:60061532-60062451- | hsa_circ_0002220 | MED13 | -1.72 | 2.67E-05 |
| chr6:20739750-20781496+ | hsa_circ_0131448 | CDKAL1 | -1.72 | 0.009732 |
| chr3:184603898-184618760+ | hsa_circ_0068367 | VPS8 | -1.72 | 0.026813 |
| chr5:77423854-77461496- | hsa_circ_0001501 | AP3B1 | -1.72 | 0.026813 |
| chr6:38224185-38256237- | hsa_circ_0076253 | BTBD9 | -1.72 | 0.001756 |
| chr20:34309662-34313077- | hsa_circ_0001148 | RBM39 | -1.72 | 0.002662 |
| chr6:99912480-99916494- | hsa_circ_0004999 | USP45 | -1.72 | 0.004215 |
| chr19:47421745-47440665+ | hsa_circ_0000943 | ARHGAP35 | -1.72 | 0.000362 |
| chr3:52771602-52775515- | hsa_circ_0001309 | NEK4 | -1.72 | 3.44E-05 |
| chr17:47375468-47390207- | | ZNF652 | -1.71 | 0.008992 |
| chr7:33055282-33075600- | | NT5C3A | -1.71 | 0.000492 |
| chr3:122471438-122496753- | hsa_circ_0121643 | HSPBAP1 | -1.71 | 0.006889 |
| chr6:35586873-35614627- | hsa_circ_0001600 | FKBP5 | -1.71 | 0.026813 |
| chr1:243433394-243471479+ | hsa_circ_0112747 | SDCCAG8 | -1.71 | 0.035219 |
| chr1:155686797-155695810+ | hsa_circ_0014611 | DAP3 | -1.71 | 0.001788 |
| chr20:35689506-35690673- | hsa_circ_0060236 | RBL1 | -1.71 | 0.005209 |
| chr7:148543562-148544397- | hsa_circ_0006357 | EZH2 | -1.71 | 0.000827 |
| chr11:77824932-77832220- | hsa_circ_0007767 | ALG8 | -1.71 | 0.002766 |
| chr7:72617463-72618644+ | hsa_circ_0002284 | GTF2IP4 | -1.71 | 0.00043 |
| chr7:48407391-48416169+ | | ABCA13 | -1.71 | 0.015529 |
| chr2:61712903-61717911- | hsa_circ_0005050 | XPO1 | -1.7 | 0.00022 |
| chr5:6623327-6625782- | hsa_circ_0007380 | NSUN2 | -1.7 | 0.009732 |
| chr2:100623094-100625394- | hsa_circ_0001055 | AFF3 | -1.7 | 0.009732 |
| chr2:69581621-69590802- | hsa_circ_0003808 | GFPT1 | -1.7 | 0.009732 |
| chr11:85707869-85712201- | hsa_circ_0002513 | PICALM | -1.7 | 0.005209 |
| chr12:27143383-27152609- | hsa_circ_0007478 | TM7SF3 | -1.7 | 0.035219 |
| chr6:32522525-32549391- | | HLA-DRB5 | -1.7 | 0.011249 |
| chr12:123078823-123109235+ | hsa_circ_0097650 | KNTC1 | -1.7 | 0.002578 |
| chr14:39627489-39628754- | hsa_circ_0002395 | TRAPPC6B | -1.7 | 0.035219 |
| chr10:116879949-116889297+ | hsa_circ_0020093 | ATRNL1 | -1.7 | 0.005209 |
| chr3:132217970-132219757+ | hsa_circ_0121937 | DNAJC13 | -1.69 | 0.008992 |
| chr3:179096129-179109892+ | hsa_circ_0122818 | MFN1 | -1.69 | 0.003957 |
| chr13:77785302-77807398- | | MYCBP2 | -1.69 | 0.015529 |
| chr1:11184555-11193254- | hsa_circ_0006576 | MTOR | -1.69 | 0.035219 |
| chr16:47143394-47165936- | hsa_circ_0003520 | NETO2 | -1.69 | 0.035219 |
| chr3:149563798-149613357+ | hsa_circ_0003956 | RNF13 | -1.69 | 0.035219 |
| chr10:115636280-115644139+ | hsa_circ_0092728 | NHLRC2 | -1.69 | 0.000274 |
| chr4:36212012-36216102- | hsa_circ_0069396 | ARAP2 | -1.69 | 0.000672 |
| chr16:66642212-66643906+ | hsa_circ_0008450 | CMTM3 | -1.69 | 0.035219 |
| chr4:99027104-99055610- | hsa_circ_0127396 | STPG2 | -1.69 | 0.015529 |
| chr1:213037067-213062599+ | hsa_circ_0000183 | FLVCR1 | -1.69 | 0.035219 |
| chr7:155499554-155538296+ | | RBM33 | -1.69 | 0.035219 |
| chrM:1701-6557- | | JA429830 | -1.69 | 0.001489 |
| chr1:67405711-67428843+ | | MIER1 | -1.68 | 2.67E-05 |
| chr7:131071879-131084192+ | hsa_circ_0001747 | MKLN1 | -1.68 | 0.00043 |
| chr6:159004986-159010814+ | hsa_circ_0001663 | TMEM181 | -1.68 | 1.26E-06 |
| chr6:16326625-16328701- | hsa_circ_0007132 | ATXN1 | -1.68 | 0.000273 |
| chr12:64803742-64815251+ | hsa_circ_0098970 | XPOT | -1.68 | 0.018448 |
| chr11:75590923-75623083+ | hsa_circ_0023637 | UVRAG | -1.68 | 0.000672 |
| chr17:15974723-15979016- | hsa_circ_0042162 | NCOR1 | -1.68 | 0.018448 |
| chr14:21825356-21829372- | hsa_circ_0000522 | SUPT16H | -1.68 | 0.008469 |
| chr10:28824491-28879761+ | hsa_circ_0018051 | WAC | -1.68 | 0.011249 |
| chr3:121215655-121217517- | hsa_circ_0121608 | POLQ | -1.68 | 0.005209 |
| chr13:53232534-53239868+ | hsa_circ_0100746 | SUGT1 | -1.68 | 0.000965 |
| chr9:132569467-132571311+ | hsa_circ_0138134 | TOR1B | -1.68 | 0.018448 |
| chr11:44129233-44135851+ | hsa_circ_0009018 | EXT2 | -1.68 | 0.001756 |
| chr18:21644104-21663045+ | hsa_circ_0047270 | TTC39C | -1.68 | 0.005043 |
| chr1:35569896-35570370+ | hsa_circ_0113143 | ZMYM1 | -1.68 | 0.015529 |
| chr9:70863722-70883894+ | | CBWD3 | -1.68 | 0.035219 |
| chr12:112509699-112516545- | hsa_circ_0028334 | NAA25 | -1.68 | 0.046229 |
| chr19:8619361-8620680- | hsa_circ_0049083 | MYO1F | -1.67 | 0.000363 |
| chr5:137533888-137537844- | hsa_circ_0128082 | CDC23 | -1.67 | 0.005209 |
| chr21:30421082-30422499+ | hsa_circ_0061366 | USP16 | -1.67 | 0.018448 |
| chr3:18504972-18568818+ | hsa_circ_0123034 | SATB1-AS1 | -1.67 | 0.035219 |
| chr13:100909849-100962162+ | hsa_circ_0099729 | PCCA | -1.67 | 0.001756 |
| chr11:47444125-47444524- | hsa_circ_0006723 | PSMC3 | -1.67 | 0.018448 |
| chr6:3076998-3081350+ | hsa_circ_0003886 | RIPK1 | -1.67 | 0.018448 |
| chr1:15860732-15874923+ | hsa_circ_0009118 | DNAJC16 | -1.67 | 0.015529 |
| chr19:8619361-8619627- | hsa_circ_0049082 | MYO1F | -1.67 | 0.003208 |
| chr10:76349040-76360251+ | hsa_circ_0018900 | ADK | -1.67 | 0.015529 |
| chr3:56703728-56707753- | hsa_circ_0001316 | FAM208A | -1.67 | 0.009732 |
| chr8:103850972-103855975- | hsa_circ_0003304 | AZIN1 | -1.67 | 0.018448 |
| chr14:31778140-31795548- | hsa_circ_0031528 | HEATR5A | -1.66 | 0.018448 |
| chr1:235850249-235860572- | hsa_circ_0017078 | LYST | -1.66 | 0.00226 |
| chr1:54506429-54509198- | hsa_circ_0012634 | TMEM59 | -1.66 | 0.000772 |
| chr15:66021410-66053776- | hsa_circ_0035947 | DENND4A | -1.66 | 0.035219 |
| chr17:17165280-17168295- | hsa_circ_0042253 | COPS3 | -1.66 | 0.018448 |
| chr9:33960824-33996331- | hsa_circ_0086724 | UBAP2 | -1.66 | 0.005043 |
| chr11:34470739-34485782+ | | CAT | -1.66 | 0.018448 |
| chr7:151891094-151935911- | | KMT2C | -1.66 | 0.018448 |
| chr12:121002875-121009094+ | hsa_circ_0005043 | RNF10 | -1.65 | 0.005209 |
| chr1:235952001-235964397- | hsa_circ_0112544 | LYST | -1.65 | 0.012401 |
| chr2:209138308-209153542+ | hsa_circ_0057975 | PIKFYVE | -1.65 | 0.003022 |
| chr3:169824620-169847340- | hsa_circ_0067894 | PHC3 | -1.65 | 0.035219 |
| chr5:37697757-37703189+ | hsa_circ_0129035 | WDR70 | -1.65 | 0.018448 |
| chr6:42582809-42600642+ | hsa_circ_0131757 | UBR2 | -1.65 | 0.046671 |
| chr19:52887102-52888995+ | hsa_circ_0109865 | ZNF880 | -1.65 | 0.003022 |
| chr15:59963382-59964938- | hsa_circ_0035537 | BNIP2 | -1.65 | 0.001641 |
| chr1:213302870-213349835+ | hsa_circ_0007772 | RPS6KC1 | -1.65 | 0.002039 |
| chrX:62917005-62944591- | hsa_circ_0008423 | ARHGEF9 | -1.65 | 0.035219 |
| chr15:62228796-62273677- | hsa_circ_0035591 | VPS13C | -1.65 | 0.001756 |
| chr12:31595709-31600703- | hsa_circ_0008445 | DENND5B | -1.65 | 0.046229 |
| chr3:142274719-142279296- | hsa_circ_0122280 | ATR | -1.65 | 0.001756 |
| chr5:50073903-50093067+ | hsa_circ_0072437 | PARP8 | -1.65 | 7.96E-06 |
| chr19:42775926-42778729+ | | CIC | -1.64 | 0.005209 |
| chr2:10559860-10560261+ | hsa_circ_0000976 | HPCAL1 | -1.64 | 0.000706 |
| chr3:127429419-127441409- | hsa_circ_0007358 | MGLL | -1.64 | 0.035219 |
| chr4:54265897-54310270+ | hsa_circ_0069747 | FIP1L1 | -1.64 | 8.60E-05 |
| chr22:43032485-43032852- | hsa_circ_0116678 | CYB5R3 | -1.64 | 0.009732 |
| chrX:122820398-122831601- | hsa_circ_0139689 | THOC2 | -1.64 | 0.018448 |
| chr9:20413719-20448264- | hsa_circ_0138442 | MLLT3 | -1.63 | 0.035219 |
| chr17:45447803-45473343+ | hsa_circ_0106987 | EFCAB13 | -1.63 | 0.008992 |
| chr2:32323865-32341281+ | hsa_circ_0119759 | SPAST | -1.63 | 8.67E-06 |
| chr10:12021056-12056183- | hsa_circ_0017713 | UPF2 | -1.63 | 0.002039 |
| chr12:125397053-125397736- | | UBC | -1.63 | 0.008992 |
| chr11:9225207-9229179- | hsa_circ_0004099 | DENND5A | -1.63 | 0.00134 |
| chr16:24104112-24105618+ | hsa_circ_0038652 | PRKCB | -1.63 | 0.008992 |
| chr15:43102812-43132631- | hsa_circ_0002466 | TTBK2 | -1.62 | 0.000239 |
| chr21:40619627-40622815- | hsa_circ_0116045 | BRWD1 | -1.62 | 0.008992 |
| chr19:22157531-22171711- | hsa_circ_0109301 | ZNF208 | -1.62 | 0.018448 |
| chr11:3720312-3722069- | | NUP98 | -1.62 | 0.015529 |
| chr1:231500073-231503382- | hsa_circ_0112381 | EGLN1 | -1.62 | 0.035219 |
| chr1:151400299-151403317- | hsa_circ_0008704 | POGZ | -1.62 | 0.046229 |
| chrX:17024344-17040394+ | hsa_circ_0139981 | REPS2 | -1.61 | 0.026813 |
| chr2:61725808-61753656- | hsa_circ_0054893 | XPO1 | -1.61 | 0.006889 |
| chr1:229602381-229623360- | hsa_circ_0112333 | NUP133 | -1.61 | 0.026813 |
| chr11:17981017-18031686- | hsa_circ_0095511 | SERGEF | -1.61 | 0.011249 |
| chr7:139796355-139820329- | hsa_circ_0133544 | KDM7A | -1.61 | 0.01941 |
| chrX:2326786-2343345- | hsa_circ_0002818 | DHRSX | -1.61 | 0.009732 |
| chr6:47541800-47549801+ | hsa_circ_0131877 | CD2AP | -1.61 | 0.004215 |
| chr1:171493960-171502100+ | hsa_circ_0111134 | PRRC2C | -1.61 | 0.015529 |
| chr5:132227856-132232932- | hsa_circ_0073901 | AFF4 | -1.61 | 0.004215 |
| chr10:103558599-103560157- | hsa_circ_0019611 | MGEA5 | -1.61 | 0.000539 |
| chr6:117037382-117045538+ | hsa_circ_0130446 | KPNA5 | -1.61 | 0.012843 |
| chr19:651611-652884- | | RNF126 | -1.6 | 0.008992 |
| chr13:100953714-101020828+ | hsa_circ_0099734 | PCCA | -1.6 | 0.046229 |
| chr17:45221249-45229284- | hsa_circ_0044231 | CDC27 | -1.6 | 0.035219 |
| chr13:111857636-111885640+ | hsa_circ_0030914 | ARHGEF7 | -1.6 | 0.026813 |
| chrM:1692-7127+ | | TVAS5 | -1.6 | 0.000918 |
| chrM:1692-15491+ | | TVAS5 | -1.6 | 0.035219 |
| chr7:155093276-155094127+ | hsa_circ_0133744 | INSIG1 | -1.6 | 0.035219 |
| chr4:73984405-73991029- | hsa_circ_0004148 | ANKRD17 | -1.6 | 0.015529 |
| chr12:10539502-10546466- | | KLRC4-KLRK1 | -1.6 | 0.035219 |
| chr14:92555074-92560175- | hsa_circ_0102917 | ATXN3 | -1.6 | 0.011249 |
| chr7:5680785-5692141- | hsa_circ_0079284 | RNF216 | -1.6 | 0.008469 |
| chr12:42768665-42768876+ | hsa_circ_0025905 | PPHLN1 | -1.59 | 0.015529 |
| chr2:203155046-203160560+ | hsa_circ_0008327 | NOP58 | -1.59 | 0.035219 |
| chr3:48581013-48587667- | hsa_circ_0065394 | PFKFB4 | -1.59 | 0.018448 |
| chr12:49333438-49334971- | hsa_circ_0003770 | ARF3 | -1.59 | 0.002398 |
| chr14:50732076-50760964- | hsa_circ_0031842 | L2HGDH | -1.59 | 0.004215 |
| chr3:113114597-113135474- | hsa_circ_0121475 | CFAP44 | -1.59 | 0.018448 |
| chr12:112086984-112087575+ | hsa_circ_0097338 | BRAP | -1.59 | 0.003208 |
| chr8:70667659-70674110- | hsa_circ_0001809 | SLCO5A1 | -1.58 | 0.004215 |
| chr16:18851020-18853776- | hsa_circ_0008216 | SMG1 | -1.58 | 0.011249 |
| chr4:1893893-1936989+ | | WHSC1 | -1.58 | 0.000212 |
| chr4:15818134-15826639+ | hsa_circ_0125616 | CD38 | -1.58 | 0.046229 |
| chr9:20907149-20933102+ | hsa_circ_0086564 | FOCAD | -1.58 | 0.035219 |
| chr1:44750507-44785416- | hsa_circ_0005716 | ERI3 | -1.58 | 0.046229 |
| chr3:129599152-129599402- | hsa_circ_0001340 | TMCC1 | -1.58 | 0.001869 |
| chr6:41839302-41859613- | hsa_circ_0131738 | USP49 | -1.58 | 0.018448 |
| chrX:95990757-96018119+ | | DIAPH2 | -1.58 | 0.005043 |
| chr11:66407171-66411611+ | | RBM4 | -1.58 | 0.029794 |
| chr7:156619299-156629579- | hsa_circ_0005939 | LMBR1 | -1.58 | 0.010014 |
| chr10:26792126-26800835+ | | APBB1IP | -1.58 | 0.018448 |
| chr14:56078737-56086030+ | hsa_circ_0032029 | KTN1 | -1.58 | 0.00134 |
| chr11:17352449-17359081+ | hsa_circ_0095506 | NUCB2 | -1.58 | 0.01941 |
| chr6:117010483-117026323+ | hsa_circ_0130438 | KPNA5 | -1.57 | 6.08E-05 |
| chr1:78268955-78272786+ | hsa_circ_0013007 | FAM73A | -1.57 | 0.026813 |
| chrM:959-8468+ | | DQ582265 | -1.56 | 0.002244 |
| chr5:34175550-34182972- | hsa_circ_0128879 | RP11-1023L17.1 | -1.56 | 0.046229 |
| chr5:171661126-171661362- | hsa_circ_0128603 | UBTD2 | -1.56 | 0.035219 |
| chr9:110068660-110074018+ | hsa_circ_0087861 | RAD23B | -1.56 | 0.035219 |
| chr2:24733838-24807429+ | hsa_circ_0006934 | NCOA1 | -1.56 | 0.029794 |
| chr19:843461-844034+ | | PRTN3 | -1.56 | 0.035219 |
| chr1:21091870-21100103- | hsa_circ_0002238 | HP1BP3 | -1.56 | 0.000147 |
| chr12:46230372-46233279+ | hsa_circ_0025951 | ARID2 | -1.56 | 0.008992 |
| chr14:24735636-24737825- | hsa_circ_0007750 | RABGGTA | -1.56 | 0.035219 |
| chr8:67747998-67752482+ | | SGK3 | -1.56 | 0.015529 |
| chr6:2836090-2836257- | hsa_circ_0131620 | SERPINB1 | -1.56 | 0.035219 |
| chr7:140476712-140508795- | hsa_circ_0007178 | BRAF | -1.56 | 0.018333 |
| chr22:28290543-28293880- | hsa_circ_0116365 | PITPNB | -1.56 | 0.00517 |
| chr3:27420740-27493989- | hsa_circ_0064616 | SLC4A7 | -1.56 | 0.026813 |
| chr22:30374431-30375041+ | hsa_circ_0062808 | MTMR3 | -1.56 | 0.008992 |
| chr5:138994171-139003046+ | hsa_circ_0074229 | UBE2D2 | -1.55 | 0.029794 |
| chr6:18256592-18258636- | hsa_circ_0008846 | DEK | -1.55 | 0.005571 |
| chr11:46837749-46842819- | hsa_circ_0021929 | CKAP5 | -1.55 | 0.035219 |
| chr7:11021999-11030474+ | hsa_circ_0133015 | PHF14 | -1.55 | 0.012401 |
| chr9:4833154-4860901+ | hsa_circ_0138825 | RCL1 | -1.55 | 0.046229 |
| chr5:179688684-179714067- | hsa_circ_0128744 | MAPK9 | -1.55 | 0.004215 |
| chr13:20304379-20356931- | hsa_circ_0029614 | PSPC1 | -1.55 | 0.006889 |
| chr1:155365250-155408859- | hsa_circ_0110879 | ASH1L | -1.55 | 0.008992 |
| chr18:51797730-51800460+ | hsa_circ_0007180 | POLI | -1.54 | 9.43E-05 |
| chr18:51686135-51731527- | hsa_circ_0005584 | MBD2 | -1.54 | 0.00134 |
| chrY:1351571-1359402+ | hsa_circ_0005021 | CSF2RA | -1.54 | 0.015529 |
| chr10:104352339-104359301+ | hsa_circ_0003779 | SUFU | -1.54 | 0.035219 |
| chr1:93575787-93595005+ | | MTF2 | -1.54 | 0.026813 |
| chr6:13641431-13644961- | hsa_circ_0130781 | RANBP9 | -1.54 | 0.008992 |
| chr10:103552596-103573363- | hsa_circ_0092433 | MGEA5 | -1.54 | 0.008992 |
| chrM:1692-5360+ | | TVAS5 | -1.54 | 0.002161 |
| chr1:156303338-156304709- | hsa_circ_0004680 | CCT3 | -1.53 | 0.030141 |
| chr18:44526020-44526886+ | hsa_circ_0108513 | KATNAL2 | -1.53 | 0.011249 |
| chr3:124456415-124463050+ | hsa_circ_0006118 | UMPS | -1.53 | 0.000742 |
| chr12:109539707-109541416+ | hsa_circ_0028097 | UNG | -1.53 | 0.035219 |
| chr9:134305477-134314448+ | hsa_circ_0089195 | PRRC2B | -1.53 | 0.026813 |
| chr11:47380395-47381591- | hsa_circ_0000302 | SPI1 | -1.53 | 0.006228 |
| chr18:18566911-18572898- | hsa_circ_0108000 | ROCK1 | -1.53 | 0.046229 |
| chr9:77769840-77783182+ | |  | -1.53 | 0.009732 |
| chr15:89656963-89659752+ | hsa_circ_0003679 | ABHD2 | -1.52 | 0.035219 |
| chr6:147636651-147646220+ | hsa_circ_0130967 | STXBP5 | -1.52 | 0.035219 |
| chr1:54266366-54298264- | hsa_circ_0009070 | NDC1 | -1.52 | 0.035219 |
| chr10:86198038-86237420+ | hsa_circ_0094299 | CCSER2 | -1.52 | 0.046229 |
| chr16:81878955-81888192+ | | PLCG2 | -1.52 | 0.035219 |
| chr1:156444900-156446994- | hsa_circ_0014737 | MEF2D | -1.52 | 0.011249 |
| chr19:7184327-7184648- | hsa_circ_0048965 | INSR | -1.52 | 0.026813 |
| chr3:47663697-47719801- | hsa_circ_0065249 | SMARCC1 | -1.52 | 0.000805 |
| chr20:34526619-34528969+ | hsa_circ_0115073 | PHF20 | -1.52 | 0.035219 |
| chr5:43292576-43297268- | hsa_circ_0008621 | HMGCS1 | -1.52 | 0.008992 |
| chr6:24777450-24781849+ | | GMNN | -1.52 | 0.035219 |
| chr11:82984690-82989872- | hsa_circ_0096576 | CCDC90B | -1.52 | 0.046229 |
| chr13:25478064-25487229- | hsa_circ_0029775 | CENPJ | -1.51 | 0.015529 |
| chr8:124243540-124243997- | hsa_circ_0007779 | C8orf76 | -1.51 | 0.029794 |
| chr4:87967318-87968834+ | | AFF1 | -1.51 | 0.029794 |
| chr3:183435439-183470041+ | hsa_circ_0068175 | YEATS2 | -1.51 | 0.018448 |
| chr1:174219613-174274265+ | | RABGAP1L | -1.51 | 0.005209 |
| chr7:158552177-158566087- | hsa_circ_0083210 | ESYT2 | -1.51 | 0.035219 |
| chr1:1192372-1192690- | hsa_circ_0008063 | UBE2J2 | -1.51 | 0.035219 |
| chr1:40654727-40668286+ | hsa_circ_0006373 | RLF | -1.5 | 0.005873 |
| chr10:94653106-94715471+ | hsa_circ_0094493 | EXOC6 | -1.5 | 0.046229 |
| chr10:46214754-46215403- | | FAM21FP | -1.5 | 0.009732 |
| chr8:21835281-21837714+ | hsa_circ_0136150 | XPO7 | -1.5 | 0.009732 |
| chrM:2250-14529- | | JA429830 | -1.5 | 0.009732 |
| chr17:16004564-16005119- | hsa_circ_0042174 | NCOR1 | -1.5 | 0.000274 |
| chr3:27453133-27465643- | hsa_circ_0002901 | SLC4A7 | -1.5 | 0.046229 |
| chr1:93198067-93202184- | hsa_circ_0007950 | EVI5 | -1.5 | 0.026813 |
| chr6:43023283-43024183- | hsa_circ_0001608 | MRPL2 | -1.49 | 0.012842 |
| chr3:32586368-32587457- | hsa_circ_0123513 | DYNC1LI1 | -1.49 | 0.007916 |
| chr5:172359439-172362313+ | hsa_circ_0004004 | ERGIC1 | -1.49 | 0.018448 |
| chr2:191520703-191537878+ | hsa_circ_0008961 | NAB1 | -1.49 | 0.026813 |
| chr12:111991962-111993723- | hsa_circ_0000439 | ATXN2 | -1.49 | 0.029794 |
| chr18:21860806-21957499- | hsa_circ_0108097 | OSBPL1A | -1.49 | 0.018333 |
| chr12:32760891-32764217+ | hsa_circ_0000390 | FGD4 | -1.49 | 0.048219 |
| chr2:136360070-136437894+ | hsa_circ_0056560 | R3HDM1 | -1.49 | 0.018333 |
| chr1:158979793-159002481+ | | IFI16 | -1.49 | 0.018448 |
| chr11:33307959-33327555+ | | HIPK3 | -1.49 | 0.006889 |
| chr17:25631806-25636298+ | hsa_circ_0008470 | WSB1 | -1.48 | 0.001028 |
| chr7:140476712-140494267- | hsa_circ_0006460 | BRAF | -1.48 | 0.007916 |
| chr17:34912923-34916711+ | hsa_circ_0005510 | GGNBP2 | -1.48 | 0.000539 |
| chr6:17661884-17676001- | hsa_circ_0003576 | NUP153 | -1.48 | 0.046229 |
| chr2:190717381-190719854+ | hsa_circ_0118387 | PMS1 | -1.48 | 0.015529 |
| chr12:8866407-8906689+ | hsa_circ_0025373 | RIMKLB | -1.48 | 0.008992 |
| chr3:37327505-37337710+ | | GOLGA4 | -1.48 | 0.046229 |
| chr11:34952951-35006275+ | hsa_circ_0021708 | PDHX | -1.48 | 0.046229 |
| chr3:172003716-172028671+ | hsa_circ_0002422 | FNDC3B | -1.48 | 0.008992 |
| chr5:145634506-145638156+ | hsa_circ_0006087 | RBM27 | -1.48 | 0.012843 |
| chr6:17624792-17649531- | hsa_circ_0075705 | NUP153 | -1.48 | 0.011249 |
| chr6:160467530-160469575+ | hsa_circ_0131235 | IGF2R | -1.48 | 0.007916 |
| chr3:129177442-129188260+ | hsa_circ_0121833 | IFT122 | -1.48 | 0.030141 |
| chr9:98740343-98766983+ | hsa_circ_0008720 | ERCC6L2 | -1.48 | 0.003957 |
| chr15:22835916-22849121+ | hsa_circ_0103110 | TUBGCP5 | -1.48 | 0.046229 |
| chr2:24778884-24816590+ | | NCOA1 | -1.47 | 0.046229 |
| chr4:156617908-156643344+ | hsa_circ_0125604 | GUCY1A3 | -1.47 | 0.008697 |
| chr17:62579579-62582288- | hsa_circ_0045322 | SMURF2 | -1.47 | 0.009732 |
| chr18:196637-204692+ | hsa_circ_0005653 | USP14 | -1.47 | 0.048219 |
| chr17:16040625-16042500- | hsa_circ_0042175 | NCOR1 | -1.47 | 0.018448 |
| chr12:121853962-121861320+ | hsa_circ_0097554 | RNF34 | -1.47 | 0.046229 |
| chr15:44776422-44778898+ | hsa_circ_0103618 | CTDSPL2 | -1.47 | 0.046229 |
| chrM:9291-9515+ | | MT-CO3 | -1.46 | 0.004448 |
| chr2:220429964-220433046- | hsa_circ_0058404 | OBSL1 | -1.46 | 0.018448 |
| chr17:41256139-41256973- | hsa_circ_0106911 | BRCA1 | -1.46 | 0.035219 |
| chr20:46262792-46268795+ | hsa_circ_0115330 | NCOA3 | -1.46 | 0.035219 |
| chr11:121391366-121403261+ | hsa_circ_0095226 | SORL1 | -1.46 | 0.026813 |
| chr2:172305191-172309723+ | hsa_circ_0057049 | DCAF17 | -1.46 | 0.035219 |
| chr19:34699834-34706566+ | hsa_circ_0050461 | LSM14A | -1.46 | 0.046229 |
| chr5:61642957-61645947+ | hsa_circ_0072593 | KIF2A | -1.46 | 0.00134 |
| chr11:85723324-85742653- | hsa_circ_0023940 | PICALM | -1.46 | 0.003208 |
| chr6:163876311-163956157+ | hsa_circ_0131264 | QKI | -1.46 | 0.035219 |
| chr14:74364774-74370813+ | hsa_circ_0102582 | ZNF410 | -1.45 | 0.035219 |
| chr6:24405396-24418806+ | hsa_circ_0075834 | MRS2 | -1.45 | 0.015529 |
| chr18:44470543-44483598- | hsa_circ_0108510 | PIAS2 | -1.45 | 0.003022 |
| chr8:101271335-101300495- | hsa_circ_0135307 | RNF19A | -1.45 | 0.035219 |
| chr8:109240496-109254142- | hsa_circ_0085323 | EIF3E | -1.45 | 0.00517 |
| chr4:123848789-123859404+ | hsa_circ_0125231 | SPATA5 | -1.44 | 0.046229 |
| chr9:140646783-140652463+ | hsa_circ_0001904 | EHMT1 | -1.44 | 9.37E-05 |
| chr13:41133646-41134997- | hsa_circ_0030042 | FOXO1 | -1.44 | 0.035219 |
| chr9:123751324-123753558- | hsa_circ_0137815 | C5 | -1.44 | 0.035219 |
| chr5:80911292-81006587- | hsa_circ_0129839 | SSBP2 | -1.44 | 0.008469 |
| chr1:108690901-108703915- | hsa_circ_0004270 | SLC25A24 | -1.44 | 0.000166 |
| chr7:6859397-6861995- | hsa_circ_0079410 | CCZ1B | -1.43 | 0.035219 |
| chr12:32751431-32764217+ | hsa_circ_0025843 | FGD4 | -1.43 | 7.34E-05 |
| chr15:66806326-66813543+ | hsa_circ_0104338 | ZWILCH | -1.43 | 0.046229 |
| chr6:90461150-90463341- | hsa_circ_0001619 | MDN1 | -1.43 | 0.004248 |
| chr12:90003714-90029028- | hsa_circ_0027707 | ATP2B1 | -1.43 | 0.030141 |
| chr1:197141290-197161029- | hsa_circ_0111630 | ZBTB41 | -1.43 | 0.035219 |
| chr2:24759584-24807429+ | hsa_circ_0119553 | NCOA1 | -1.43 | 0.035219 |
| chr2:20482708-20527139- | hsa_circ_0118857 | PUM2 | -1.43 | 5.62E-05 |
| chr17:80521230-80529746+ | hsa_circ_0000817 | FOXK2 | -1.43 | 0.029794 |
| chr11:3794862-3797251- | hsa_circ_0004960 | NUP98 | -1.43 | 0.000462 |
| chr15:90982564-90986710+ | hsa_circ_0000651 | IQGAP1 | -1.43 | 0.008707 |
| chr7:5963018-5963593+ | hsa_circ_0007177 | CCZ1 | -1.43 | 0.000283 |
| chr1:167921038-167944253+ | hsa_circ_0000154 | DCAF6 | -1.43 | 0.029794 |
| chr15:64496640-64497148- | hsa_circ_0035828 | CSNK1G1 | -1.42 | 0.018333 |
| chr1:44713611-44804994- | hsa_circ_0012138 | ERI3 | -1.42 | 0.035219 |
| chr1:176012322-176015460- | hsa_circ_0005357 | RFWD2 | -1.42 | 0.046229 |
| chrX:117874980-117900939+ | | IL13RA1 | -1.42 | 0.026813 |
| chr13:28588589-28624359- | hsa_circ_0100161 | FLT3 | -1.42 | 0.00157 |
| chr12:54905587-54911722+ | | NCKAP1L | -1.42 | 0.018333 |
| chr15:94888358-94945248+ | | MCTP2 | -1.42 | 0.035219 |
| chr2:54278095-54284497+ | hsa_circ_0004462 | ACYP2 | -1.42 | 0.018903 |
| chr4:103225474-103246155- | hsa_circ_0124882 | SLC39A8 | -1.42 | 0.002076 |
| chr2:69565030-69590802- | hsa_circ_0055048 | GFPT1 | -1.42 | 0.015529 |
| chr1:202418117-202471133+ | hsa_circ_0111717 | PPP1R12B | -1.42 | 0.018333 |
| chr3:47651556-47719801- | hsa_circ_0065244 | SMARCC1 | -1.42 | 0.000669 |
| chr8:124265562-124268411- | hsa_circ_0135655 | ZHX1 | -1.41 | 0.035219 |
| chr9:33953283-33973235- | hsa_circ_0003141 | UBAP2 | -1.41 | 0.046229 |
| chr1:41578955-41608784- | hsa_circ_0000063 | SCMH1 | -1.41 | 0.018333 |
| chr16:74685818-74695349- | hsa_circ_0040498 | RFWD3 | -1.41 | 0.011249 |
| chr13:103275227-103280274+ | hsa_circ_0008881 | TPP2 | -1.41 | 0.003957 |
| chr15:52073241-52075025+ | hsa_circ_0005566 | TMOD2 | -1.41 | 0.005209 |
| chr6:82923944-82924546- | hsa_circ_0132391 | IBTK | -1.41 | 0.018903 |
| chr10:103552596-103570071- | hsa_circ_0019607 | MGEA5 | -1.41 | 2.27E-05 |
| chr1:247016393-247051819- | hsa_circ_0017319 | AHCTF1 | -1.41 | 0.015529 |
| chr5:37120318-37121886- | | C5orf42 | -1.4 | 0.026813 |
| chr18:29218607-29246335- | hsa_circ_0108197 | B4GALT6 | -1.4 | 0.000793 |
| chr13:52992127-53000201- | hsa_circ_0030340 | VPS36 | -1.4 | 0.004141 |
| chr11:18536225-18537711- | hsa_circ_0021464 | TSG101 | -1.4 | 0.018333 |
| chr22:32874968-32881196+ | hsa_circ_0001222 | FBXO7 | -1.4 | 0.029794 |
| chr11:108137898-108138069+ | hsa_circ_0007694 | ATM | -1.4 | 0.002718 |
| chr10:1125951-1126416+ | hsa_circ_0004277 | WDR37 | -1.4 | 0.007916 |
| chr4:76885288-76903190- | hsa_circ_0070022 | SDAD1 | -1.4 | 0.046671 |
| chr9:128099297-128099870+ | hsa_circ_0003270 | GAPVD1 | -1.4 | 0.012842 |
| chr3:138382748-138403645- | hsa_circ_0067520 | PIK3CB | -1.39 | 0.046229 |
| chr2:87279937-87282923- | hsa_circ_0055548 | LOC285074 | -1.39 | 0.008469 |
| chr6:76412361-76419346+ | hsa_circ_0077095 | SENP6 | -1.39 | 0.029794 |
| chr2:24962301-24965055+ | hsa_circ_0119596 | NCOA1 | -1.39 | 0.035219 |
| chr18:48444480-48466756+ | hsa_circ_0108584 | ME2 | -1.39 | 0.026813 |
| chr16:68191772-68225678+ | | NFATC3 | -1.39 | 0.008084 |
| chr22:22123484-22162135- | hsa_circ_0062436 | MAPK1 | -1.38 | 0.00517 |
| chr12:42748963-42792796+ | hsa_circ_0006189 | PPHLN1 | -1.38 | 0.046229 |
| chr10:70723047-70726959+ | hsa_circ_0008523 | DDX21 | -1.37 | 0.01941 |
| chr14:50136242-50141145- | hsa_circ_0004904 | POLE2 | -1.37 | 0.012842 |
| chr17:29550462-29554624+ | hsa_circ_0003586 | NF1 | -1.37 | 0.003957 |
| chr2:64189192-64211153- | hsa_circ_0054958 | VPS54 | -1.37 | 0.015529 |
| chr14:104120901-104121162+ | hsa_circ_0004107 | KLC1 | -1.37 | 0.004215 |
| chr11:77330651-77340944- | hsa_circ_0008342 | CLNS1A | -1.36 | 0.001271 |
| chr13:28840852-28845003+ | hsa_circ_0000469 | PAN3 | -1.36 | 0.040426 |
| chr3:149613260-149639014+ | hsa_circ_0006801 | RNF13 | -1.36 | 0.004901 |
| chr13:42040959-42042974+ | hsa_circ_0100479 | RGCC | -1.36 | 0.048219 |
| chr1:24766662-24771734+ | hsa_circ_0010909 | NIPAL3 | -1.36 | 0.026813 |
| chrX:73071958-73072109- | | XIST | -1.36 | 0.018109 |
| chr5:169230062-169267856+ | hsa_circ_0074954 | DOCK2 | -1.36 | 0.01937 |
| chr5:107521818-107559929- | hsa_circ_0127477 | FBXL17 | -1.36 | 0.035219 |
| chr15:66007816-66048810- | hsa_circ_0035938 | DENND4A | -1.36 | 0.00297 |
| chr15:66048478-66053776- | hsa_circ_0104325 | DENND4A | -1.36 | 0.002662 |
| chr17:9843443-9850300- | hsa_circ_0042079 | GAS7 | -1.36 | 0.010292 |
| chr3:195086211-195112876- | hsa_circ_0001996 | ACAP2 | -1.35 | 0.043761 |
| chr16:10524468-10525312+ | hsa_circ_0037807 | ATF7IP2 | -1.35 | 0.004197 |
| chr2:96550088-96551868- | | ANKRD36C | -1.35 | 0.018448 |
| chr4:71628236-71634378+ | hsa_circ_0002048 | RUFY3 | -1.35 | 0.005571 |
| chr5:70840184-70849147+ | hsa_circ_0129503 | BDP1 | -1.35 | 0.035219 |
| chr15:75693071-75715164- | hsa_circ_0104552 | SIN3A | -1.35 | 0.026813 |
| chr15:66727365-66729230+ | hsa_circ_0008798 | MAP2K1 | -1.35 | 0.048219 |
| chr16:18809247-18810156- | hsa_circ_0004833 | ARL6IP1 | -1.35 | 0.029794 |
| chr6:108888375-108890945+ | hsa_circ_0130221 | FOXO3 | -1.35 | 0.040426 |
| chr5:151166164-151183557+ | hsa_circ_0128355 | G3BP1 | -1.35 | 0.035219 |
| chr10:12123471-12155063+ | hsa_circ_0017731 | DHTKD1 | -1.35 | 0.046671 |
| chr17:47388674-47389404- | hsa_circ_0003258 | ZNF652 | -1.35 | 0.003208 |
| chr7:66273873-66275834+ | hsa_circ_0005592 | RABGEF1 | -1.34 | 0.018333 |
| chr16:27357788-27364017+ | hsa_circ_0038724 | IL4R | -1.34 | 0.02771 |
| chr20:34304662-34313077- | hsa_circ_0001147 | RBM39 | -1.34 | 0.00096 |
| chr10:70514471-70517134+ | hsa_circ_0018557 | CCAR1 | -1.34 | 0.015529 |
| chr6:74190398-74191967+ | hsa_circ_0004973 | MTO1 | -1.34 | 0.046671 |
| chr3:195016437-195022903- | hsa_circ_0002382 | ACAP2 | -1.34 | 0.01937 |
| chr2:89476005-89544456- | |  | -1.34 | 0.000827 |
| chr2:100046302-100052403- | hsa_circ_0003100 | REV1 | -1.34 | 0.029794 |
| chr12:110450930-110467440+ | hsa_circ_0097224 | ANKRD13A | -1.34 | 0.035219 |
| chr5:74130251-74137504- | hsa_circ_0004405 | FAM169A | -1.33 | 0.000742 |
| chr13:21729832-21732264- | hsa_circ_0100051 | SKA3 | -1.33 | 0.035219 |
| chr2:227729320-227732034+ | hsa_circ_0058493 | RHBDD1 | -1.33 | 4.44E-05 |
| chr20:35315895-35317187- | hsa_circ_0005523 | NDRG3 | -1.33 | 0.046229 |
| chr11:3752621-3774638- | hsa_circ_0000274 | NUP98 | -1.33 | 0.007465 |
| chr22:46125305-46136418+ | hsa_circ_0001247 | ATXN10 | -1.33 | 7.16E-05 |
| chr3:171965323-172028671+ | hsa_circ_0005700 | FNDC3B | -1.33 | 0.012842 |
| chr10:94632916-94679807+ | hsa_circ_0094488 | EXOC6 | -1.33 | 0.01941 |
| chr2:215645284-215661841- | hsa_circ_0058055 | BARD1 | -1.33 | 0.047316 |
| chr12:29446233-29474857+ | hsa_circ_0098299 | FAR2 | -1.33 | 0.035219 |
| chrX:147733520-147744289+ | hsa_circ_0091669 | AFF2 | -1.33 | 0.00137 |
| chr15:86118362-86129054+ | hsa_circ_0104792 | AKAP13 | -1.33 | 0.035219 |
| chr6:7226700-7232140+ | | RREB1 | -1.32 | 0.046229 |
| chr12:27148170-27156323- | hsa_circ_0098248 | TM7SF3 | -1.32 | 0.029794 |
| chr3:197592294-197602646+ | hsa_circ_0008351 | LRCH3 | -1.32 | 0.014362 |
| chr5:147774277-147805264+ | hsa_circ_0074472 | FBXO38 | -1.32 | 0.024189 |
| chr8:131172110-131199531- | hsa_circ_0005189 | ASAP1 | -1.32 | 0.025972 |
| chr1:234742704-234743598- | | IRF2BP2 | -1.32 | 0.046229 |
| chr15:94910834-94928754+ | | MCTP2 | -1.31 | 0.002247 |
| chr6:34935016-34937943+ | hsa_circ_0076078 | ANKS1A | -1.31 | 0.025945 |
| chr7:6854395-6862991- | hsa_circ_0003943 | CCZ1B | -1.31 | 0.046229 |
| chr9:123199573-123222945- | hsa_circ_0088282 | CDK5RAP2 | -1.31 | 0.035219 |
| chr3:171360630-171410232- | hsa_circ_0067954 | PLD1 | -1.31 | 0.030141 |
| chr3:133907681-133914026- | hsa_circ_0005768 | RYK | -1.31 | 0.048219 |
| chr15:59373135-59373483+ | hsa_circ_0035479 | RNF111 | -1.31 | 0.026813 |
| chr3:149563798-149619949+ | hsa_circ_0004565 | RNF13 | -1.31 | 0.004266 |
| chr11:68359044-68367962+ | hsa_circ_0001968 | PPP6R3 | -1.31 | 0.012401 |
| chr19:38621138-38621412+ | | SIPA1L3 | -1.31 | 0.026813 |
| chr2:61721029-61722748- | hsa_circ_0006033 | XPO1 | -1.31 | 0.026813 |
| chr12:96717726-96728643- | hsa_circ_0002762 | CDK17 | -1.31 | 0.008469 |
| chr8:42202471-42207583+ | hsa_circ_0136552 | POLB | -1.31 | 0.008992 |
| chr15:81271107-81274523- | hsa_circ_0002255 | G028499 | -1.31 | 0.00023 |
| chr8:133634857-133673873- | hsa_circ_0135795 | LRRC6 | -1.3 | 0.007465 |
| chr14:76638186-76647192+ | hsa_circ_0003819 | GPATCH2L | -1.3 | 0.048219 |
| chr9:36351112-36376124- | hsa_circ_0087015 | RNF38 | -1.3 | 0.008697 |
| chr3:17051166-17056403+ | hsa_circ_0001274 | PLCL2 | -1.3 | 0.001197 |
| chr12:83250789-83324324+ | hsa_circ_0099364 | TMTC2 | -1.3 | 0.048219 |
| chrX:117895101-117900939+ | | IL13RA1 | -1.3 | 0.048219 |
| chr16:15758593-15771806+ | hsa_circ_0105072 | NDE1 | -1.3 | 0.005209 |
| chr21:40600426-40601362- | hsa_circ_0001195 | BRWD1 | -1.3 | 0.000123 |
| chr15:50940884-50955243- | hsa_circ_0007775 | TRPM7 | -1.29 | 0.024189 |
| chrX:3735586-3740621- | | LOC389906 | -1.29 | 0.035219 |
| chr3:169831148-169867032- | hsa_circ_0067897 | PHC3 | -1.29 | 0.048219 |
| chr1:213037067-213061936+ | hsa_circ_0016408 | FLVCR1 | -1.29 | 0.048219 |
| chr10:98703870-98711953+ | hsa_circ_0008102 | LCOR | -1.29 | 0.005552 |
| chr9:140705913-140712590+ | hsa_circ_0001906 | EHMT1 | -1.29 | 0.004121 |
| chr3:196098767-196129890- | hsa_circ_0068651 | UBXN7 | -1.29 | 0.046671 |
| chr2:152285298-152303089+ | hsa_circ_0056714 | RIF1 | -1.28 | 0.026813 |
| chr3:71064700-71102924- | hsa_circ_0001320 | FOXP1 | -1.28 | 0.011113 |
| chr6:154520802-154544377- | hsa_circ_0131108 | IPCEF1 | -1.28 | 0.008707 |
| chr20:43623731-43629994+ | hsa_circ_0060530 | STK4 | -1.28 | 0.043761 |
| chr11:119144578-119145663+ | hsa_circ_0000362 | CBL | -1.28 | 0.00145 |
| chr21:34804484-34805178+ | hsa_circ_0001185 | IFNGR2 | -1.27 | 1.70E-05 |
| chr5:70888753-70900295+ | hsa_circ_0072904 | MCCC2 | -1.27 | 0.005371 |
| chr10:120488807-120489922- | hsa_circ_0020134 | CACUL1 | -1.27 | 0.046671 |
| chr10:70513609-70551098+ | hsa_circ_0094023 | CCAR1 | -1.27 | 0.046671 |
| chr5:76745585-76760634- | hsa_circ_0008861 | WDR41 | -1.27 | 0.000126 |
| chr5:175716657-175717958+ | hsa_circ_0075092 | SIMC1 | -1.26 | 0.000408 |
| chr14:89041037-89044484+ | hsa_circ_0003164 | ZC3H14 | -1.26 | 0.046229 |
| chr22:41277774-41278181+ | hsa_circ_0008360 | XPNPEP3 | -1.26 | 0.002161 |
| chr3:180651122-180667131+ | hsa_circ_0122866 | FXR1 | -1.25 | 0.029794 |
| chrX:154736559-154766779- | hsa_circ_0006355 | TMLHE | -1.25 | 0.010396 |
| chr14:90390857-90398971- | hsa_circ_0102829 | EFCAB11 | -1.25 | 0.043761 |
| chr4:119026173-119064839+ | hsa_circ_0125149 | NDST3 | -1.25 | 0.018333 |
| chr14:73614503-73614814+ | hsa_circ_0003848 | PSEN1 | -1.25 | 0.004967 |
| chr7:158672375-158698782+ | hsa_circ_0083228 | WDR60 | -1.25 | 0.01941 |
| chr15:59205698-59209198- | | SLTM | -1.25 | 0.015529 |
| chr12:1480999-1553916+ | hsa_circ_0006747 | ERC1 | -1.25 | 0.008577 |
| chr8:61484605-61504528+ | hsa_circ_0007581 | RAB2A | -1.24 | 0.011113 |
| chr4:3188324-3190820+ | hsa_circ_0126192 | HTT | -1.24 | 0.001275 |
| chr20:35812583-35812776+ | hsa_circ_0005759 | RPN2 | -1.24 | 0.026813 |
| chr5:80063752-80088663+ | hsa_circ_0073177 | MSH3 | -1.24 | 0.002786 |
| chr21:17150223-17191165+ | hsa_circ_0115654 | USP25 | -1.24 | 0.012843 |
| chr20:18523663-18535034+ | hsa_circ_0006612 | SEC23B | -1.24 | 0.046229 |
| chr2:37283583-37284623- | hsa_circ_0054086 | HEATR5B | -1.24 | 0.048219 |
| chr1:78183552-78191447- | hsa_circ_0000087 | USP33 | -1.23 | 0.009401 |
| chr22:46739159-46742441+ | hsa_circ_0116802 | TRMU | -1.23 | 0.046229 |
| chr9:36351112-36353328- | hsa_circ_0006515 | RNF38 | -1.23 | 0.005946 |
| chr2:136505837-136528305+ | hsa_circ_0056589 | UBXN4 | -1.23 | 0.048219 |
| chr1:25666965-25669564+ | hsa_circ_0000032 | TMEM50A | -1.23 | 0.003054 |
| chr5:138994171-138994551+ | hsa_circ_0006716 | UBE2D2 | -1.23 | 0.049001 |
| chr15:51743842-51745838- | hsa_circ_0035276 | DMXL2 | -1.23 | 0.006034 |
| chr7:151932902-151945705- | hsa_circ_0133714 | KMT2C | -1.22 | 0.005571 |
| chr1:63269390-63300523+ | hsa_circ_0113857 | ATG4C | -1.22 | 0.02771 |
| chr16:16101673-16196574+ | | ABCC1 | -1.22 | 0.004215 |
| chr5:14601074-14602441+ | hsa_circ_0128250 | FAM105A | -1.22 | 0.026813 |
| chr12:42778742-42792796+ | hsa_circ_0025908 | PPHLN1 | -1.22 | 0.046229 |
| chr15:42553156-42560230- | hsa_circ_0034803 | TMEM87A | -1.22 | 0.005209 |
| chr1:35846860-35854683+ | hsa_circ_0000051 | ZMYM4 | -1.22 | 0.000152 |
| chr4:103635595-103657592- | hsa_circ_0124902 | MANBA | -1.22 | 0.046671 |
| chr4:113483527-113511030- | hsa_circ_0125030 | ZGRF1 | -1.22 | 0.013402 |
| chr6:74175932-74176329+ | hsa_circ_0007874 | MTO1 | -1.22 | 0.047316 |
| chr12:27143383-27156323- | hsa_circ_0005824 | TM7SF3 | -1.21 | 0.030141 |
| chr22:35727289-35727488+ | | TOM1 | -1.21 | 0.029794 |
| chr6:13579683-13601181+ | hsa_circ_0075648 | SIRT5 | -1.21 | 0.029794 |
| chr4:1902353-1936989+ | hsa_circ_0001387 | WHSC1 | -1.21 | 0.003259 |
| chr3:171969050-172025291+ | hsa_circ_0067991 | FNDC3B | -1.21 | 0.001218 |
| chr3:156411809-156413814+ | hsa_circ_0006999 | TIPARP | -1.2 | 0.030991 |
| chr8:71071740-71075089- | hsa_circ_0001810 | NCOA2 | -1.2 | 0.004197 |
| chr17:33689758-33690845- | hsa_circ_0043110 | SLFN11 | -1.2 | 0.01941 |
| chr15:74963796-74967483- | hsa_circ_0000633 | EDC3 | -1.2 | 0.018333 |
| chr7:6624647-6624891+ | hsa_circ_0079385 | ZDHHC4 | -1.2 | 0.002662 |
| chr3:119222379-119222868+ | hsa_circ_0008394 | TIMMDC1 | -1.2 | 0.002968 |
| chr1:25666965-25683344+ | hsa_circ_0112900 | TMEM50A | -1.2 | 0.01937 |
| chr13:100953714-100962162+ | hsa_circ_0002664 | PCCA | -1.2 | 0.01941 |
| chr4:39734979-39757359+ | | UBE2K | -1.19 | 0.046229 |
| chr1:12061458-12062160+ | hsa_circ_0006470 | MFN2 | -1.19 | 0.00157 |
| chr10:93754292-93768032+ | hsa_circ_0008148 | BTAF1 | -1.19 | 0.008084 |
| chr2:32602656-32620661+ | hsa_circ_0053441 | BIRC6 | -1.18 | 0.048219 |
| chr2:24777258-24787299+ | hsa_circ_0119555 | NCOA1 | -1.18 | 0.029217 |
| chr10:26789748-26792203+ | hsa_circ_0006063 | APBB1IP | -1.18 | 0.012842 |
| chr20:32207323-32216241+ | hsa_circ_0059862 | CBFA2T2 | -1.18 | 0.046229 |
| chr12:123829805-123834988- | hsa_circ_0002510 | SBNO1 | -1.18 | 0.004266 |
| chr2:44428325-44436466+ | hsa_circ_0004916 | PPM1B | -1.18 | 0.002578 |
| chr1:84644860-84663507+ | hsa_circ_0114305 | PRKACB | -1.18 | 0.008504 |
| chr2:136360070-136362586+ | hsa_circ_0056558 | R3HDM1 | -1.18 | 0.001089 |
| chr10:70196768-70229920- | hsa_circ_0018524 | DNA2 | -1.18 | 0.012578 |
| chr19:48733695-48744320- | | CARD8 | -1.18 | 0.01941 |
| chr5:68669657-68670538+ | hsa_circ_0129459 | RAD17 | -1.18 | 0.030141 |
| chr20:44593997-44594413- | | ZNF335 | -1.17 | 0.01718 |
| chr4:108603171-108615162- | hsa_circ_0005965 | PAPSS1 | -1.17 | 0.029794 |
| chr14:102486230-102489217+ | hsa_circ_0008683 | DYNC1H1 | -1.17 | 0.046671 |
| chr17:647676-651272- | hsa_circ_0107503 | GEMIN4 | -1.17 | 0.01941 |
| chr5:82832826-82850857+ | hsa_circ_0073239 | VCAN | -1.17 | 0.001683 |
| chr17:73238417-73239651- | hsa_circ_0003684 | GGA3 | -1.17 | 0.011655 |
| chr15:63988323-64008672- | hsa_circ_0035796 | HERC1 | -1.17 | 4.63E-05 |
| chr5:137342679-137356886- | hsa_circ_0007235 | FAM13B | -1.17 | 0.030141 |
| chr2:111423840-111431942- | hsa_circ_0056040 | BUB1 | -1.17 | 0.026813 |
| chr9:70863722-70900956+ | | CBWD3 | -1.17 | 0.046229 |
| chr7:77210744-77236618+ | hsa_circ_0080836 | PTPN12 | -1.17 | 0.015529 |
| chr1:10155519-10165802+ | hsa_circ_0005199 | UBE4B | -1.16 | 0.042586 |
| chr15:49528048-49531564+ | hsa_circ_0008488 | GALK2 | -1.16 | 0.003927 |
| chr14:39782544-39796226+ | hsa_circ_0031749 | CTAGE5 | -1.16 | 0.007916 |
| chr10:89685270-89712016+ | | PTEN | -1.16 | 0.046671 |
| chr19:20718856-20721359- | hsa_circ_0109282 | ZNF737 | -1.16 | 0.048219 |
| chr17:25630393-25636298+ | hsa_circ_0007716 | WSB1 | -1.15 | 0.01718 |
| chr2:44436349-44445676+ | hsa_circ_0009062 | PPM1B | -1.15 | 0.001562 |
| chr1:153640031-153641033- | hsa_circ_0110830 | ILF2 | -1.15 | 0.030141 |
| chr1:155891166-155891709- | hsa_circ_0008998 | KIAA0907 | -1.15 | 0.042586 |
| chr7:99672758-99673253- | hsa_circ_0081386 | ZNF3 | -1.15 | 0.006889 |
| chr15:93498653-93510743+ | hsa_circ_0104929 | CHD2 | -1.15 | 0.015529 |
| chr17:58012554-58018304+ | | RPS6KB1 | -1.14 | 0.048219 |
| chr3:52238709-52240767+ | hsa_circ_0002338 | ALAS1 | -1.14 | 0.004746 |
| chr9:88190230-88211364- | hsa_circ_0005202 | AGTPBP1 | -1.14 | 0.013402 |
| chr2:24021008-24056948- | hsa_circ_0052943 | ATAD2B | -1.14 | 0.018903 |
| chr20:57574398-57574864- | | CTSZ | -1.14 | 0.048219 |
| chr17:27613010-27614734- | hsa_circ_0106467 | NUFIP2 | -1.14 | 0.004236 |
| chr8:74585342-74601048- | hsa_circ_0001811 | STAU2 | -1.14 | 0.0003 |
| chr12:29491464-29494749- | hsa_circ_0098304 | ERGIC2 | -1.14 | 0.040426 |
| chr13:28771322-28794515+ | hsa_circ_0006597 | PAN3 | -1.14 | 0.004197 |
| chr12:110376185-110377052- | hsa_circ_0097219 | GIT2 | -1.13 | 0.046229 |
| chr16:72122886-72124685- | hsa_circ_0040414 | TXNL4B | -1.13 | 0.014836 |
| chr19:19756683-19757156- | hsa_circ_0050256 | ATP13A1 | -1.13 | 0.012843 |
| chr13:21974512-21999817- | hsa_circ_0029708 | ZDHHC20 | -1.13 | 0.012158 |
| chr3:167319915-167322256- | hsa_circ_0122624 | WDR49 | -1.13 | 0.030991 |
| chr4:54292039-54310270+ | hsa_circ_0001413 | FIP1L1 | -1.13 | 0.019289 |
| chr9:88233898-88248289- | hsa_circ_0001868 | AGTPBP1 | -1.13 | 7.69E-05 |
| chr3:196118684-196134264- | hsa_circ_0123212 | UBXN7 | -1.13 | 0.018333 |
| chr2:102035313-102038934- | hsa_circ_0007859 | RFX8 | -1.13 | 0.02771 |
| chr17:30267305-30300250+ | hsa_circ_0005804 | SUZ12 | -1.12 | 0.02771 |
| chr15:50168475-50226379- | | ATP8B4 | -1.12 | 0.011655 |
| chr1:235582788-235590554+ | hsa_circ_0006474 | TBCE | -1.12 | 0.046229 |
| chr2:44190727-44202356- | hsa_circ_0054391 | LRPPRC | -1.12 | 0.008697 |
| chr12:6442235-6443410- | hsa_circ_0025128 | TNFRSF1A | -1.12 | 0.01056 |
| chr3:195415404-195416309+ | hsa_circ_0008267 | LINC00969 | -1.12 | 0.002313 |
| chr14:99723808-99724176- | hsa_circ_0033144 | BCL11B | -1.12 | 0.023796 |
| chr10:22002701-22016857+ | hsa_circ_0007084 | MLLT10 | -1.12 | 0.01941 |
| chr2:68717322-68772444+ | hsa_circ_0001023 | APLF | -1.12 | 0.001058 |
| chr18:12356693-12371690- | hsa_circ_0046965 | AFG3L2 | -1.12 | 0.044005 |
| chr1:9991949-9992956- | hsa_circ_0009677 | LZIC | -1.12 | 0.048219 |
| chr22:36696251-36696920- | | MYH9 | -1.11 | 0.048219 |
| chrX:149761067-149787612+ | hsa_circ_0139897 | MTM1 | -1.11 | 0.016204 |
| chr2:197943427-197954756- | hsa_circ_0008443 | ANKRD44 | -1.11 | 0.048219 |
| chr16:50783487-50788335+ | hsa_circ_0039348 | CYLD | -1.11 | 0.046229 |
| chr22:32180800-32188803+ | hsa_circ_0116466 | DEPDC5 | -1.11 | 0.028722 |
| chr10:98376396-98416691- | | PIK3AP1 | -1.11 | 0.046229 |
| chrM:1680-11960+ | | TVAS5 | -1.1 | 0.004277 |
| chr19:12790271-12791139- | hsa_circ_0000893 | DHPS | -1.1 | 0.029794 |
| chr6:77981057-77983024- | hsa_circ_0132345 | G071718 | -1.1 | 0.001084 |
| chr3:136162129-136221621- | hsa_circ_0122078 | STAG1 | -1.1 | 0.010014 |
| chr1:93676359-93682303+ | hsa_circ_0002304 | CCDC18 | -1.1 | 0.02771 |
| chr6:29856890-29911700+ | | HLA-G | -1.1 | 0.0004 |
| chr6:42902213-42903408+ | hsa_circ_0008801 | CNPY3 | -1.09 | 0.037082 |
| chr3:130649260-130660543+ | hsa_circ_0005797 | ATP2C1 | -1.09 | 0.02771 |
| chr10:76729418-76748870+ | hsa_circ_0094190 | KAT6B | -1.09 | 0.029217 |
| chr8:17868746-17869336+ | hsa_circ_0083465 | PCM1 | -1.09 | 0.000722 |
| chr20:13509080-13550233- | hsa_circ_0114682 | TASP1 | -1.09 | 0.029794 |
| chr10:51853028-51853677+ | hsa_circ_0002261 | FAM21A | -1.09 | 0.046229 |
| chrM:13856-14125- | | JA760602 | -1.08 | 0.004266 |
| chr14:58785260-58796887+ | hsa_circ_0000542 | ARID4A | -1.08 | 0.005183 |
| chr17:45740455-45745776+ | hsa_circ_0002854 | KPNB1 | -1.08 | 0.043761 |
| chr9:88257742-88327481- | hsa_circ_0139210 | AGTPBP1 | -1.08 | 0.029217 |
| chr5:142264863-142311690+ | hsa_circ_0074362 | ARHGAP26 | -1.08 | 0.012892 |
| chr4:2956151-2958538- | hsa_circ_0068958 | NOP14 | -1.08 | 0.030141 |
| chr8:52744004-52773806- | hsa_circ_0136720 | PCMTD1 | -1.08 | 0.008982 |
| chr9:88211277-88248289- | hsa_circ_0007162 | AGTPBP1 | -1.08 | 0.000925 |
| chr7:33397467-33427756+ | hsa_circ_0008336 | BBS9 | -1.07 | 0.046671 |
| chr2:38536521-38546161- | hsa_circ_0002918 | ATL2 | -1.07 | 0.00683 |
| chr1:167734820-167745403+ | hsa_circ_0000153 | MPZL1 | -1.07 | 0.043761 |
| chr2:68717322-68805135+ | hsa_circ_0120801 | APLF | -1.07 | 0.018903 |
| chr2:32142995-32145979- | hsa_circ_0006790 | MEMO1 | -1.07 | 0.008202 |
| chr4:107092252-107133992- | hsa_circ_0007540 | TBCK | -1.07 | 0.048219 |
| chr5:142421381-142437312+ | hsa_circ_0128196 | ARHGAP26 | -1.07 | 0.048219 |
| chr1:58971732-59004982- | hsa_circ_0002316 | OMA1 | -1.07 | 0.000225 |
| chr2:26587170-26596497+ | hsa_circ_0005029 | EPT1 | -1.06 | 0.005571 |
| chrM:5054-5349+ | | MT-ND2 | -1.06 | 0.004215 |
| chr16:4516154-4519466- | hsa_circ_0007788 | NMRAL1 | -1.05 | 0.014973 |
| chr6:35586873-35610620- | hsa_circ_0001599 | FKBP5 | -1.05 | 0.024189 |
| chr1:58971732-59002413- | hsa_circ_0000072 | OMA1 | -1.05 | 0.002674 |
| chr6:111067329-111095140- | hsa_circ_0130325 | CDK19 | -1.05 | 0.024189 |
| chr8:42812237-42814457+ | hsa_circ_0084162 | HOOK3 | -1.05 | 0.035219 |
| chr15:50875286-50884823- | hsa_circ_0035228 | TRPM7 | -1.05 | 0.02771 |
| chr7:8043538-8110761+ | hsa_circ_0134778 | GLCCI1 | -1.04 | 0.047316 |
| chr5:167915607-167924353+ | hsa_circ_0001551 | RARS | -1.04 | 0.038524 |
| chr9:33986758-33998862- | hsa_circ_0001852 | UBAP2 | -1.04 | 0.012578 |
| chr11:77402204-77404656- | hsa_circ_0004831 | RSF1 | -1.04 | 0.016601 |
| chr19:58904343-58904854+ | hsa_circ_0005598 | RPS5 | -1.04 | 0.048219 |
| chr17:59853762-59857762- | hsa_circ_0006968 | BRIP1 | -1.04 | 0.037588 |
| chr8:100146860-100205285+ | hsa_circ_0135257 | VPS13B | -1.03 | 0.018109 |
| chr21:40578034-40584633- | hsa_circ_0116009 | BRWD1 | -1.03 | 0.002189 |
| chr12:2790820-2791778- | hsa_circ_0098282 | CACNA1C-AS1 | -1.03 | 0.047316 |
| chr1:33413823-33415375- | hsa_circ_0000048 | RNF19B | -1.03 | 0.003044 |
| chr19:52873040-52896041+ | hsa_circ_0109854 | ZNF880 | -1.03 | 0.026813 |
| chr12:77242013-77243250+ | hsa_circ_0099231 | ZDHHC17 | -1.02 | 0.015529 |
| chrX:79962926-79975155- | hsa_circ_0001936 | BRWD3 | -1.02 | 0.001966 |
| chr7:26724355-26766625- | | SKAP2 | -1.02 | 0.046229 |
| chr7:6840579-6841154- | hsa_circ_0007135 | CCZ1B | -1.01 | 0.004307 |
| chr1:31465237-31468067- | hsa_circ_0000043 | PUM1 | -1.01 | 0.007465 |
| chr18:19345733-19383975+ | hsa_circ_0007504 | MIB1 | -1.01 | 0.018903 |
| chr1:45223228-45223812+ | hsa_circ_0002563 | KIF2C | -1.01 | 0.012842 |
| chr19:50998560-50999865+ | | XLOC_l2_007096 | -1.01 | 0.048219 |
| chr15:31266517-31269158- | hsa_circ_0103279 | MTMR10 | -1.01 | 0.024189 |
| chr10:70227880-70229920- | hsa_circ_0006151 | DNA2 | -1 | 0.00831 |
| chr1:246890194-246907427+ | hsa_circ_0112862 | SCCPDH | -1 | 0.022057 |
| chr1:247302018-247323115- | hsa_circ_0002802 | ZNF124 | -1 | 0.005963 |
| chr1:149239868-149255307+ | hsa_circ_0110719 | RP11-403I13.4 | -1 | 0.048219 |
| chr5:140961879-140963182- | hsa_circ_0008982 | DIAPH1 | -1 | 0.043761 |
| chr19:47767860-47768203+ | hsa_circ_0000944 | CCDC9 | -0.99 | 0.002235 |
| chr5:70331372-70333373- | | NAIP | -0.99 | 0.003039 |
| chr13:20625573-20641530+ | hsa_circ_0029633 | ZMYM2 | -0.99 | 0.015429 |
| chr21:34022494-34025676- | hsa_circ_0061485 | SYNJ1 | -0.98 | 0.029794 |
| chrM:1680-14385+ | | TVAS5 | -0.98 | 0.041819 |
| chr22:46085592-46136418+ | hsa_circ_0008199 | ATXN10 | -0.98 | 0.043761 |
| chr5:145863103-145886770+ | hsa_circ_0128248 | TCERG1 | -0.98 | 0.043761 |
| chr7:17908030-17937069- | hsa_circ_0004671 | SNX13 | -0.97 | 0.015957 |
| chr3:149563798-149639014+ | hsa_circ_0001346 | RNF13 | -0.97 | 0.001345 |
| chr13:77779391-77818086- | hsa_circ_0030509 | MYCBP2 | -0.97 | 0.003592 |
| chr5:107684100-107703654- | hsa_circ_0001518 | FBXL17 | -0.97 | 0.038423 |
| chr9:123914763-123922580+ | hsa_circ_0001884 | CNTRL | -0.96 | 0.029217 |
| chr1:146757975-146759412+ | hsa_circ_0006296 | CHD1L | -0.96 | 0.028197 |
| chr6:146185524-146216113- | hsa_circ_0130934 | SHPRH | -0.95 | 0.021871 |
| chr6:7181357-7189555+ | hsa_circ_0007429 | RREB1 | -0.95 | 0.006801 |
| chr19:52785364-52786666+ | hsa_circ_0052095 | ZNF766 | -0.95 | 0.007344 |
| chr7:102398301-102401858+ | hsa_circ_0007520 | FAM185A | -0.95 | 0.007837 |
| chr11:82874721-82888246+ | hsa_circ_0096567 | PCF11 | -0.94 | 0.046229 |
| chr12:116675273-116675510- | hsa_circ_0028587 | MED13L | -0.94 | 0.018903 |
| chr22:42807413-42807742- | hsa_circ_0001240 | NFAM1 | -0.94 | 0.046671 |
| chr11:120916383-120930794+ | hsa_circ_0003302 | TBCEL | -0.93 | 0.002434 |
| chr7:97820040-97823884+ | hsa_circ_0001725 | LMTK2 | -0.93 | 0.014995 |
| chr2:202163961-202228896- | | ALS2CR12 | -0.93 | 0.043761 |
| chr16:71954642-71957283+ | hsa_circ_0005016 | IST1 | -0.93 | 0.046671 |
| chr1:167935867-167944253+ | hsa_circ_0009109 | DCAF6 | -0.93 | 0.011299 |
| chrM:1680-9522+ | | TVAS5 | -0.93 | 0.001203 |
| chr2:239090706-239093928- | hsa_circ_0001116 | ILKAP | -0.92 | 0.028722 |
| chr15:38794513-38798121- | hsa_circ_0034414 | RASGRP1 | -0.92 | 0.017367 |
| chr13:28608024-28624359- | hsa_circ_0100165 | FLT3 | -0.92 | 0.006801 |
| chr18:9583115-9595100- | hsa_circ_0009022 | PPP4R1 | -0.92 | 0.002619 |
| chr17:62289934-62291602- | hsa_circ_0006479 | TEX2 | -0.91 | 0.009779 |
| chr3:171969050-172028671+ | hsa_circ_0003692 | FNDC3B | -0.91 | 0.005243 |
| chr14:80963812-80997230- | hsa_circ_0007332 | CEP128 | -0.91 | 0.042586 |
| chr1:52959283-52975384- | hsa_circ_0003632 | ZCCHC11 | -0.9 | 0.010086 |
| chr3:50004903-50012825+ | hsa_circ_0065769 | RBM6 | -0.9 | 0.006647 |
| chr4:37633007-37640126- | hsa_circ_0001400 | RELL1 | -0.9 | 0.007426 |
| chrM:959-14067+ | | DQ582265 | -0.9 | 0.004559 |
| chr8:68044186-68049838+ | hsa_circ_0003388 | CSPP1 | -0.89 | 0.037082 |
| chr2:122363277-122363756- | hsa_circ_0007052 | CLASP1 | -0.89 | 0.032667 |
| chr12:27075549-27081838- | hsa_circ_0025693 | ASUN | -0.89 | 0.043761 |
| chr17:975854-1003975- | hsa_circ_0041188 | ABR | -0.89 | 0.026813 |
| chrX:53588717-53589890- | hsa_circ_0090740 | HUWE1 | -0.89 | 0.012578 |
| chr1:27056142-27059283+ | hsa_circ_0008494 | ARID1A | -0.89 | 0.005903 |
| chr11:77386081-77389198- | hsa_circ_0096495 | RSF1 | -0.89 | 0.033913 |
| chr6:90556281-90566918+ | hsa_circ_0001621 | CASP8AP2 | -0.88 | 0.022454 |
| chr4:52729603-52758017+ | hsa_circ_0007646 | DCUN1D4 | -0.88 | 0.012921 |
| chr9:128419930-128434922- | hsa_circ_0001890 | MAPKAP1 | -0.87 | 0.031552 |
| chr3:27478879-27490288- | hsa_circ_0006215 | SLC4A7 | -0.87 | 0.040426 |
| chr12:66603236-66639042+ | hsa_circ_0099010 | IRAK3 | -0.87 | 0.046229 |
| chr16:23999829-24046868+ | hsa_circ_0000681 | PRKCB | -0.86 | 0.018612 |
| chr13:50279757-50280508- | hsa_circ_0007232 | KPNA3 | -0.85 | 0.008577 |
| chr12:42745687-42792796+ | hsa_circ_0004815 | PPHLN1 | -0.84 | 0.043215 |
| chr15:55516087-55527154- | hsa_circ_0003863 | RAB27A | -0.84 | 0.009679 |
| chr3:171965323-172016577+ | hsa_circ_0122727 | FNDC3B | -0.84 | 0.030923 |
| chr5:77385217-77425131- | hsa_circ_0008164 | AP3B1 | -0.84 | 0.039376 |
| chr1:180953813-180962561- | hsa_circ_0007905 | STX6 | -0.83 | 0.009163 |
| chr7:64004085-64004810- | hsa_circ_0002566 | ZNF680 | -0.83 | 0.013813 |
| chr10:12039671-12056183- | hsa_circ_0000213 | UPF2 | -0.83 | 0.002681 |
| chr7:151946961-151948051- | hsa_circ_0005823 | KMT2C | -0.82 | 0.023055 |
| chr4:1902353-1920350+ | hsa_circ_0004156 | WHSC1 | -0.82 | 0.020814 |
| chr7:91924203-91948826+ | hsa_circ_0001723 | ANKIB1 | -0.82 | 0.018597 |
| chr3:190321917-190347287+ | | IL1RAP | -0.82 | 0.048219 |
| chr12:123030723-123032516+ | hsa_circ_0029098 | KNTC1 | -0.82 | 0.048219 |
| chr2:215617171-215661841- | hsa_circ_0002999 | BARD1 | -0.81 | 0.016061 |
| chr2:135010666-135012215+ | hsa_circ_0001068 | MGAT5 | -0.81 | 0.038524 |
| chr1:169947226-170001116- | hsa_circ_0111121 | KIFAP3 | -0.81 | 0.007338 |
| chr9:96233423-96238620+ | hsa_circ_0008193 | FAM120A | -0.81 | 0.048399 |
| chr18:13681604-13682104- | hsa_circ_0003979 | FAM210A | -0.8 | 0.026581 |
| chr1:146724278-146731572+ | hsa_circ_0004220 | CHD1L | -0.8 | 0.030991 |
| chr17:65941525-65972074+ | hsa_circ_0000799 | BPTF | -0.8 | 0.004322 |
| chr20:39721112-39729993+ | hsa_circ_0115215 | TOP1 | -0.8 | 0.026296 |
| chrX:147743429-147744289+ | hsa_circ_0001947 | AFF2 | -0.79 | 0.003039 |
| chr18:19345733-19399607+ | hsa_circ_0000836 | MIB1 | -0.78 | 0.019318 |
| chr3:114069121-114070725- | hsa_circ_0005332 | ZBTB20 | -0.78 | 0.040833 |
| chrX:117676688-117680079+ | hsa_circ_0139638 | DOCK11 | -0.78 | 0.048102 |
| chr14:21971316-21972024- | hsa_circ_0000523 | METTL3 | -0.77 | 0.027524 |
| chr7:158566032-158591763- | hsa_circ_0083220 | ESYT2 | -0.77 | 0.042387 |
| chr3:138244332-138256189- | hsa_circ_0122120 | CEP70 | -0.76 | 0.030923 |
| chr4:39328183-39329376- | hsa_circ_0001403 | RFC1 | -0.75 | 0.012158 |
| chr3:179131200-179137293- | hsa_circ_0001364 | GNB4 | -0.74 | 0.030936 |
| chr15:76566753-76584854- | | ETFA | -0.74 | 0.046229 |
| chr4:106155054-106158508+ | hsa_circ_0070562 | TET2 | -0.73 | 0.027013 |
| chr6:158703295-158735300+ | hsa_circ_0131196 | G073721 | -0.72 | 0.037588 |
| chr20:32207323-32211102+ | hsa_circ_0003426 | CBFA2T2 | -0.72 | 0.019318 |
| chr5:32135678-32143986- | hsa_circ_0001470 | GOLPH3 | -0.72 | 0.040897 |
| chr5:122881111-122893258+ | hsa_circ_0001522 | CSNK1G3 | -0.71 | 0.012272 |
| chr1:115005726-115007010- | hsa_circ_0004127 | TRIM33 | -0.7 | 0.021427 |
| chr6:158994452-159010814+ | hsa_circ_0004587 | TMEM181 | -0.69 | 0.043215 |
| chr2:197872530-197873755- | hsa_circ_0001087 | ANKRD44 | -0.69 | 0.016601 |
| chr11:85722073-85742653- | hsa_circ_0000347 | PICALM | -0.69 | 0.029385 |
| chr1:1583739-1646968- | | CDK11B | -0.69 | 0.034153 |
| chr17:45438744-45456619+ | hsa_circ_0106983 | EFCAB13 | -0.68 | 0.033704 |
| chr4:73950966-73958017- | hsa_circ_0001417 | ANKRD17 | -0.66 | 0.019437 |
| chrX:53430498-53430825- | hsa_circ_0001921 | SMC1A | -0.66 | 0.017367 |
| chr14:32559708-32586493+ | hsa_circ_0031584 | ARHGAP5 | -0.66 | 0.022552 |
| chr10:5815805-5842668- | hsa_circ_0017586 | GDI2 | -0.65 | 0.045292 |
| chr8:52758221-52773806- | hsa_circ_0136721 | PCMTD1 | -0.65 | 0.014465 |
| chr10:7285520-7327916- | hsa_circ_0017636 | SFMBT2 | -0.64 | 0.040897 |
| chr7:40027198-40041630+ | hsa_circ_0001699 | CDK13 | -0.64 | 0.044334 |
| chr2:95814630-95819004- | hsa_circ_0121204 | ZNF514 | -0.63 | 0.023752 |
| chr14:99924616-99932150- | hsa_circ_0000567 | SETD3 | -0.6 | 0.027228 |
| chr15:41961026-41962156+ | hsa_circ_0000591 | MGA | -0.6 | 0.025985 |
| chr15:32814649-32825569- | | WHAMMP1 | -0.58 | 0.040001 |
| chr15:76152219-76165909+ | hsa_circ_0104568 | UBE2Q2 | -0.58 | 0.033707 |
| chr8:48308936-48320523+ | hsa_circ_0001798 | SPIDR | -0.58 | 0.037234 |
| chr17:20107646-20109225+ | hsa_circ_0000745 | SPECC1 | -0.57 | 0.028943 |
| chr3:18419662-18462483- | hsa_circ_0064555 | SATB1 | -0.57 | 0.046556 |
| chr10:126370176-126370948- | hsa_circ_0000267 | FAM53B | -0.55 | 0.03854 |
| chr21:34793787-34805178+ | hsa_circ_0002113 | IFNGR2 | -0.53 | 0.049001 |
| chr11:74521229-74528759+ | hsa_circ_0003206 | RNF169 | 0.497 | 0.049001 |
| chr5:138699448-138700432+ | hsa_circ_0001538 | PAIP2 | 0.503 | 0.031435 |
| chr1:51860053-51913807- | hsa_circ_0012425 | EPS15 | 0.525 | 0.04854 |
| chr12:94562929-94580249+ | hsa_circ_0099504 | PLXNC1 | 0.541 | 0.043733 |
| chr13:28598998-28602425- | hsa_circ_0100164 | FLT3 | 0.554 | 0.039043 |
| chr5:56160561-56161804+ | hsa_circ_0001485 | MAP3K1 | 0.562 | 0.027249 |
| chr15:80412670-80415142+ | hsa_circ_0000643 | ZFAND6 | 0.591 | 0.038288 |
| chr5:50045985-50074484+ | hsa_circ_0072430 | PARP8 | 0.614 | 0.01941 |
| chr1:1158624-1159348- | hsa_circ_0000002 | SDF4 | 0.626 | 0.045596 |
| chr11:66407171-66407594+ | hsa_circ_0000330 | RBM4 | 0.633 | 0.022441 |
| chr21:16386665-16415895- | hsa_circ_0004771 | NRIP1 | 0.651 | 0.040775 |
| chr2:24357989-24369956+ | hsa_circ_0000982 | FAM228B | 0.653 | 0.024366 |
| chr11:35218293-35232996+ | | CD44 | 0.663 | 0.030991 |
| chr17:4186093-4200109- | hsa_circ_0003239 | UBE2G1 | 0.682 | 0.011471 |
| chr2:37227729-37229715- | hsa_circ_0119896 | HEATR5B | 0.692 | 0.021289 |
| chr7:92118607-92119225- | hsa_circ_0081024 | PEX1 | 0.696 | 0.046229 |
| chr7:38282029-38296186- | | TRGC2 | 0.697 | 0.039376 |
| chr14:39648295-39648666+ | hsa_circ_0101802 | PNN | 0.705 | 0.029886 |
| chr15:89656956-89659752+ | hsa_circ_0007099 | ABHD2 | 0.72 | 0.015545 |
| chr1:117944808-117963271+ | hsa_circ_0000118 | MAN1A2 | 0.726 | 0.008958 |
| chr7:5413686-5434226- | | TNRC18 | 0.732 | 0.048219 |
| chr21:37619815-37620866+ | hsa_circ_0001187 | DOPEY2 | 0.739 | 0.013894 |
| chr9:123924134-123928458+ | hsa_circ_0088333 | CNTRL | 0.74 | 0.043988 |
| chr22:25771780-25777545- | hsa_circ_0003102 | LRP5L | 0.742 | 0.020682 |
| chr1:151630711-151655921+ | hsa_circ_0014191 | SNX27 | 0.744 | 0.046229 |
| chr18:29412047-29419420- | hsa_circ_0003805 | TRAPPC8 | 0.757 | 0.021289 |
| chr10:5827105-5842668- | hsa_circ_0005379 | GDI2 | 0.758 | 0.048561 |
| chr5:132400672-132406188+ | hsa_circ_0127950 | HSPA4 | 0.762 | 0.046229 |
| chr16:68155890-68160513+ | hsa_circ_0000711 | NFATC3 | 0.766 | 0.009272 |
| chr2:201721405-201721708- | hsa_circ_0004001 | CLK1 | 0.774 | 0.04458 |
| chr21:38792601-38845182+ | hsa_circ_0005955 | DYRK1A | 0.781 | 0.044601 |
| chr15:76566753-76588078- | hsa_circ_0000638 | ETFA | 0.783 | 0.016528 |
| chr18:77455225-77464917+ | hsa_circ_0006209 | CTDP1 | 0.783 | 0.035147 |
| chr17:81042814-81043199+ | | METRNL | 0.784 | 0.011218 |
| chr12:1136914-1137738+ | hsa_circ_0000373 | ERC1 | 0.785 | 0.018126 |
| chr12:50848097-50855130+ | hsa_circ_0003222 | LARP4 | 0.786 | 0.01941 |
| chr3:152132730-152165562+ | hsa_circ_0122407 | MBNL1 | 0.814 | 0.048219 |
| chr1:28800066-28800663+ | hsa_circ_0002909 | PHACTR4 | 0.823 | 0.017444 |
| chr18:39607407-39629569+ | hsa_circ_0007765 | PIK3C3 | 0.827 | 0.029813 |
| chr18:9208655-9221997+ | hsa_circ_0003652 | ANKRD12 | 0.828 | 0.048493 |
| chr3:171965323-171969331+ | hsa_circ_0006156 | FNDC3B | 0.83 | 0.006028 |
| chr3:71090479-71102924- | hsa_circ_0008234 | FOXP1 | 0.843 | 0.025037 |
| chr10:32197100-32199491- | hsa_circ_0000231 | ARHGAP12 | 0.847 | 0.009863 |
| chr4:39739040-39779430+ | hsa_circ_0069492 | UBE2K | 0.85 | 0.028722 |
| chr2:70406664-70409129- | hsa_circ_0055113 | C2orf42 | 0.852 | 0.018656 |
| chr2:231307652-231314970+ | hsa_circ_0003922 | SP100 | 0.856 | 0.006317 |
| chr3:169694734-169706147+ | hsa_circ_0001358 | SEC62 | 0.857 | 0.00606 |
| chr14:20871536-20874559- | hsa_circ_0005721 | TEP1 | 0.862 | 0.02771 |
| chr7:90355881-90377090+ | hsa_circ_0004015 | CDK14 | 0.863 | 0.030991 |
| chr6:139264650-139265759- | hsa_circ_0004368 | REPS1 | 0.866 | 0.008344 |
| chr7:23224689-23226765+ | hsa_circ_0001683 | NUPL2 | 0.87 | 0.020314 |
| chr9:33960824-33973235- | hsa_circ_0001850 | UBAP2 | 0.875 | 0.022317 |
| chr3:171969050-171979873+ | hsa_circ_0122728 | FNDC3B | 0.879 | 0.008127 |
| chr6:87920169-87928449+ | hsa_circ_0004383 | ZNF292 | 0.881 | 0.00606 |
| chr15:75219107-75221573- | hsa_circ_0005725 | COX5A | 0.881 | 0.001911 |
| chr19:18650181-18650530- | hsa_circ_0000915 | FKBP8 | 0.883 | 0.024189 |
| chr17:57808782-57816308+ | hsa_circ_0006508 | VMP1 | 0.887 | 0.002319 |
| chr16:80718435-80719026- | hsa_circ_0004087 | CDYL2 | 0.89 | 0.005577 |
| chr7:43679048-43680248- | hsa_circ_0001700 | COA1 | 0.896 | 0.014836 |
| chr7:91924203-91981956+ | hsa_circ_0002744 | ANKIB1 | 0.898 | 0.020145 |
| chr11:129979324-129980556+ | hsa_circ_0000372 | APLP2 | 0.899 | 0.008054 |
| chr22:29120965-29121355- | hsa_circ_0002931 | CHEK2 | 0.902 | 0.030923 |
| chr1:155646339-155649303- | hsa_circ_0014606 | YY1AP1 | 0.906 | 0.003568 |
| chr21:44270151-44296877- | hsa_circ_0001196 | WDR4 | 0.91 | 0.031552 |
| chr18:74561482-74563895+ | hsa_circ_0004979 | ZNF236 | 0.912 | 0.007107 |
| chr4:103635595-103647840- | hsa_circ_0001432 | MANBA | 0.913 | 0.004565 |
| chr3:101389974-101391057- | hsa_circ_0003179 | ZBTB11 | 0.916 | 0.00569 |
| chr15:85656608-85669605+ | hsa_circ_0036627 | PDE8A | 0.917 | 0.046671 |
| chr2:215632206-215646233- | hsa_circ_0001098 | BARD1 | 0.921 | 0.0223 |
| chr8:120800578-120810086- | hsa_circ_0135601 | TAF2 | 0.923 | 0.037588 |
| chr6:31238163-31323269- | | HLA-C | 0.924 | 0.004923 |
| chr9:20907149-20933131+ | hsa_circ_0006641 | FOCAD | 0.927 | 0.048219 |
| chr9:128677965-128697886+ | hsa_circ_0003991 | PBX3 | 0.928 | 0.044005 |
| chr20:30954187-30956926+ | hsa_circ_0001136 | ASXL1 | 0.93 | 0.001508 |
| chr4:140058784-140060651- | hsa_circ_0007466 | ELF2 | 0.93 | 0.042586 |
| chr10:70497602-70502326+ | hsa_circ_0006272 | CCAR1 | 0.933 | 0.044005 |
| chr17:35800606-35804870+ | hsa_circ_0043282 | TADA2A | 0.936 | 0.047316 |
| chr4:110412483-110427668+ | hsa_circ_0070635 | SEC24B | 0.937 | 0.046229 |
| chr2:32602656-32631625+ | hsa_circ_0000989 | BIRC6 | 0.94 | 0.022454 |
| chr1:202724399-202733273- | hsa_circ_0004981 | KDM5B | 0.94 | 0.046394 |
| chr3:196533450-196539722+ | hsa_circ_0004950 | PAK2 | 0.941 | 0.043215 |
| chr12:99071203-99080651+ | hsa_circ_0004901 | APAF1 | 0.941 | 0.014995 |
| chr4:128938518-128950008+ | hsa_circ_0070930 | C4orf29 | 0.942 | 0.021871 |
| chr7:716866-751164- | hsa_circ_0008039 | PRKAR1B | 0.943 | 0.034766 |
| chr9:123593609-123595734- | hsa_circ_0088300 | PSMD5 | 0.945 | 0.034766 |
| chr4:77045802-77065626- | hsa_circ_0070033 | NUP54 | 0.947 | 0.025972 |
| chr7:27668990-27672064- | hsa_circ_0003958 | HIBADH | 0.949 | 0.004901 |
| chr15:34548430-34551145- | hsa_circ_0006009 | SLC12A6 | 0.949 | 0.035219 |
| chr9:123924134-123924549+ | hsa_circ_0001885 | CNTRL | 0.952 | 0.010871 |
| chr1:21076216-21100103- | hsa_circ_0000023 | HP1BP3 | 0.954 | 0.025796 |
| chr8:22332467-22333137+ | hsa_circ_0083619 | PPP3CC | 0.955 | 0.017207 |
| chr7:56049903-56051561+ | hsa_circ_0001709 | GBAS | 0.959 | 0.032667 |
| chrM:8928-9190+ | | OK/SW-cl.16 | 0.96 | 0.019561 |
| chr22:47022648-47033857+ | hsa_circ_0001250 | GRAMD4 | 0.962 | 0.013328 |
| chr3:167240103-167254788- | hsa_circ_0122613 | WDR49 | 0.964 | 0.041676 |
| chrX:117788565-117788924+ | hsa_circ_0007733 | DOCK11 | 0.97 | 0.00537 |
| chr13:28608024-28611425- | | FLT3 | 0.973 | 0.01718 |
| chr12:42604157-42604482- | hsa_circ_0000392 | YAF2 | 0.978 | 0.003141 |
| chr6:155095123-155154543+ | hsa_circ_0131121 | SCAF8 | 0.979 | 0.037082 |
| chr1:32495899-32498935+ | hsa_circ_0007777 | KHDRBS1 | 0.986 | 0.033009 |
| chr9:4823548-4833228+ | hsa_circ_0006134 | RCL1 | 0.988 | 0.042387 |
| chr2:61009020-61010344+ | hsa_circ_0054683 | PAPOLG | 0.988 | 0.030141 |
| chr13:46577274-46594692- | hsa_circ_0100603 | ZC3H13 | 0.995 | 0.048219 |
| chr6:42559889-42562042+ | hsa_circ_0001603 | UBR2 | 0.999 | 0.011921 |
| chr16:85667520-85667738+ | hsa_circ_0000722 | GSE1 | 1.001 | 0.000717 |
| chr14:31404369-31425448- | hsa_circ_0031446 | STRN3 | 1.004 | 0.0223 |
| chr14:31097415-31107453+ | hsa_circ_0031417 | SCFD1 | 1.007 | 0.048219 |
| chr5:112321532-112339774+ | hsa_circ_0001520 | DCP2 | 1.007 | 0.007152 |
| chr2:64083440-64085070+ | hsa_circ_0001020 | UGP2 | 1.012 | 0.008744 |
| chr8:98698896-98703416+ | hsa_circ_0002961 | MTDH | 1.013 | 0.009486 |
| chr7:77200395-77230123+ | hsa_circ_0080835 | PTPN12 | 1.013 | 0.023796 |
| chr13:37614542-37625720- | hsa_circ_0000475 | SUPT20H | 1.014 | 0.044005 |
| chr13:114265311-114277601+ | hsa_circ_0031038 | TFDP1 | 1.014 | 0.018903 |
| chr16:17352845-17353355- | hsa_circ_0003559 | XYLT1 | 1.015 | 0.01056 |
| chrX:24190832-24197887+ | hsa_circ_0007108 | ZFX | 1.015 | 0.002674 |
| chr7:128214774-128220148+ | hsa_circ_0002299 | RP11-274B21.1 | 1.017 | 0.046229 |
| chr15:50294350-50311173- | | ATP8B4 | 1.018 | 0.048219 |
| chr10:5838726-5842668- | hsa_circ_0002478 | GDI2 | 1.02 | 0.046671 |
| chr6:29797195-29856519+ | | HLA-G | 1.033 | 0.025972 |
| chr2:106774514-106782539- | hsa_circ_0001060 | UXS1 | 1.035 | 0.004337 |
| chr14:91947920-91952074- | hsa_circ_0003045 | PPP4R3A | 1.037 | 0.002462 |
| chr6:10702602-10702872+ | hsa_circ_0005917 | PAK1IP1 | 1.041 | 0.012843 |
| chr4:148860976-148876525+ | hsa_circ_0001449 | ARHGAP10 | 1.041 | 0.018109 |
| chr12:95602619-95605043- | hsa_circ_0099549 | FGD6 | 1.049 | 0.023214 |
| chr13:21735929-21746820- | hsa_circ_0029696 | SKA3 | 1.05 | 0.012921 |
| chr7:139415731-139416814- | hsa_circ_0001756 | HIPK2 | 1.051 | 0.002763 |
| chr11:82560091-82571159- | hsa_circ_0096558 | PRCP | 1.055 | 0.029794 |
| chr19:34921481-34955036+ | hsa_circ_0050511 | UBA2 | 1.056 | 0.026813 |
| chr3:171426545-171455872- | hsa_circ_0067961 | PLD1 | 1.057 | 0.026813 |
| chr22:41738533-41739580+ | hsa_circ_0001236 | ZC3H7B | 1.06 | 0.046671 |
| chr2:98158542-98160325- | | ANKRD36B | 1.061 | 0.01937 |
| chr1:29481208-29481422- | hsa_circ_0006602 | SRSF4 | 1.063 | 0.000361 |
| chr19:21216262-21216990+ | hsa_circ_0008838 | ZNF430 | 1.065 | 0.006228 |
| chr1:32377297-32385259- | hsa_circ_0004893 | PTP4A2 | 1.065 | 0.026813 |
| chr10:32854486-32873232+ | hsa_circ_0008679 | CCDC7 | 1.066 | 0.004545 |
| chr14:62188227-62188541+ | hsa_circ_0007976 | HIF1A | 1.067 | 0.011655 |
| chr22:41734317-41735195+ | hsa_circ_0007382 | ZC3H7B | 1.07 | 0.029794 |
| chr17:30310018-30315516+ | hsa_circ_0002629 | SUZ12 | 1.07 | 0.005185 |
| chr16:28734485-28746903+ | | EIF3C | 1.072 | 0.008577 |
| chr19:18453669-18454225+ | hsa_circ_0109240 | PGPEP1 | 1.073 | 0.046671 |
| chr17:1264386-1265302- | hsa_circ_0007643 | YWHAE | 1.073 | 0.034031 |
| chr1:21102217-21102498- | hsa_circ_0111829 | HP1BP3 | 1.075 | 0.037082 |
| chr2:99786013-99787892- | hsa_circ_0001050 | MITD1 | 1.076 | 0.007756 |
| chr21:47768926-47769734+ | hsa_circ_0002903 | PCNT | 1.083 | 0.008202 |
| chr2:100078958-100081447- | hsa_circ_0001053 | REV1 | 1.084 | 0.00297 |
| chr19:23316882-23318845+ | hsa_circ_0008122 | ZNF730 | 1.085 | 0.031097 |
| chr2:168920010-168986268- | hsa_circ_0005882 | STK39 | 1.086 | 0.043761 |
| chr10:35772332-35805551+ | hsa_circ_0018200 | CCNY | 1.088 | 0.029794 |
| chr16:53289512-53308214+ | hsa_circ_0039366 | CHD9 | 1.088 | 0.037082 |
| chr6:32521789-32548678- | | HLA-DRB5 | 1.089 | 0.038423 |
| chr4:56877578-56878151+ | hsa_circ_0003985 | CEP135 | 1.09 | 0.033913 |
| chr5:94244956-94259726- | hsa_circ_0008417 | MCTP1 | 1.091 | 0.002054 |
| chr8:124138296-124138927+ | hsa_circ_0085447 | TBC1D31 | 1.092 | 0.046229 |
| chr3:171442486-171455872- | | PLD1 | 1.093 | 0.048219 |
| chr13:20425495-20426330- | hsa_circ_0002673 | ZMYM5 | 1.093 | 0.015957 |
| chr9:138758302-138774924- | hsa_circ_0006814 | CAMSAP1 | 1.095 | 0.025972 |
| chr12:42604157-42604421- | hsa_circ_0004502 | YAF2 | 1.098 | 0.008357 |
| chr10:35349802-35360267- | hsa_circ_0000234 | CUL2 | 1.099 | 0.012842 |
| chr3:33725851-33738425- | hsa_circ_0001280 | CLASP2 | 1.099 | 0.001169 |
| chr9:33948372-33953472- | hsa_circ_0003496 | UBAP2 | 1.1 | 0.003196 |
| chr11:75715049-75728024+ | hsa_circ_0096463 | UVRAG | 1.104 | 0.035219 |
| chr12:46285563-46287504+ | hsa_circ_0004985 | ARID2 | 1.112 | 0.048219 |
| chr13:20638591-20657916+ | hsa_circ_0100006 | ZMYM2 | 1.117 | 0.02771 |
| chr17:59999086-60002460- | hsa_circ_0007759 | INTS2 | 1.118 | 0.033913 |
| chr2:168920010-168931741- | hsa_circ_0003279 | STK39 | 1.119 | 0.010608 |
| chr1:236332006-236343328+ | hsa_circ_0112550 | GPR137B | 1.122 | 0.035219 |
| chr11:62295898-62296299- | | AHNAK | 1.123 | 0.035219 |
| chr1:109427897-109446876+ | hsa_circ_0013386 | GPSM2 | 1.126 | 0.030141 |
| chr19:50902105-50902741+ | hsa_circ_0052011 | POLD1 | 1.128 | 0.046671 |
| chr5:148904608-148913337- | hsa_circ_0128324 | CSNK1A1 | 1.128 | 0.035219 |
| chr2:197860103-197865226- | hsa_circ_0057611 | ANKRD44 | 1.129 | 0.011249 |
| chr17:57430576-57430887+ | hsa_circ_0005600 | YPEL2 | 1.13 | 0.044645 |
| chr14:76633006-76647192+ | hsa_circ_0005267 | GPATCH2L | 1.131 | 0.024189 |
| chr8:27987019-28019595+ | hsa_circ_0001784 | ELP3 | 1.131 | 0.017886 |
| chr2:160229556-160245996- | hsa_circ_0056830 | BAZ2B | 1.132 | 0.046671 |
| chr7:104925456-104937980- | hsa_circ_0003656 | SRPK2 | 1.132 | 0.018903 |
| chr2:242283167-242287606+ | hsa_circ_0119498 | 2-Sep | 1.134 | 0.012401 |
| chr13:96636058-96651561- | hsa_circ_0030632 | UGGT2 | 1.134 | 0.008992 |
| chr2:20507739-20527139- | hsa_circ_0052867 | PUM2 | 1.134 | 0.029217 |
| chr9:33971649-34017187- | hsa_circ_0086732 | UBAP2 | 1.135 | 0.035219 |
| chr6:35586873-35614143- | | FKBP5 | 1.137 | 0.035219 |
| chr13:49742749-49762769+ | | FNDC3A | 1.138 | 0.035219 |
| chr5:56526673-56527148+ | hsa_circ_0001486 | GPBP1 | 1.141 | 0.018109 |
| chr13:78293667-78327493+ | hsa_circ_0000497 | SLAIN1 | 1.141 | 0.002786 |
| chr2:230723488-230744844- | hsa_circ_0003273 | TRIP12 | 1.141 | 0.001786 |
| chr20:50133323-50140649- | hsa_circ_0060849 | NFATC2 | 1.141 | 0.027169 |
| chr5:80832348-80833211- | hsa_circ_0129832 | SSBP2 | 1.142 | 0.048219 |
| chr1:179972309-179975702+ | hsa_circ_0003942 | CEP350 | 1.142 | 0.028337 |
| chr6:137015278-137041727- | hsa_circ_0007798 | MAP3K5 | 1.144 | 0.031552 |
| chr14:50130033-50141145- | hsa_circ_0008002 | POLE2 | 1.144 | 0.00063 |
| chr3:71739161-71777774- | hsa_circ_0124571 | EIF4E3 | 1.145 | 0.029794 |
| chr1:222805507-222819027+ | hsa_circ_0016531 | MIA3 | 1.145 | 0.046229 |
| chr12:22796697-22826594+ | hsa_circ_0008664 | ETNK1 | 1.145 | 0.011113 |
| chr8:124089351-124157095+ | hsa_circ_0135643 | TBC1D31 | 1.148 | 0.014362 |
| chr5:68590620-68607031- | hsa_circ_0072780 | CCDC125 | 1.148 | 0.005194 |
| chr2:61712903-61720188- | hsa_circ_0054861 | XPO1 | 1.149 | 0.035219 |
| chrX:123156381-123176495+ | hsa_circ_0091461 | STAG2 | 1.149 | 0.048219 |
| chr10:26800676-26802589+ | hsa_circ_0093391 | APBB1IP | 1.15 | 0.035219 |
| chr3:149563798-149613347+ | hsa_circ_0067717 | RNF13 | 1.153 | 0.029794 |
| chr3:32774915-32778982+ | hsa_circ_0123528 | CNOT10 | 1.156 | 0.048219 |
| chr8:67747998-67748309+ | hsa_circ_0136930 | SGK3 | 1.156 | 0.029217 |
| chr4:103644028-103647840- | hsa_circ_0006007 | MANBA | 1.157 | 0.033913 |
| chr17:43552466-43555513- | hsa_circ_0003434 | PLEKHM1 | 1.159 | 0.046671 |
| chr17:35800606-35827629+ | hsa_circ_0106716 | TADA2A | 1.159 | 0.029794 |
| chr1:67423742-67428843+ | hsa_circ_0113954 | MIER1 | 1.16 | 0.000367 |
| chr4:144464662-144465125+ | hsa_circ_0001445 | SMARCA5 | 1.16 | 0.000781 |
| chr3:119219542-119236162+ | hsa_circ_0066874 | TIMMDC1 | 1.162 | 0.047316 |
| chr18:45391430-45396935- | hsa_circ_0003694 | SMAD2 | 1.163 | 0.02154 |
| chr1:46105882-46108171- | hsa_circ_0008774 | GPBP1L1 | 1.165 | 0.000169 |
| chr11:118425174-118430579- | hsa_circ_0008157 | IFT46 | 1.165 | 0.043761 |
| chr18:77193579-77227582+ | hsa_circ_0048025 | NFATC1 | 1.166 | 0.018448 |
| chr2:69585461-69590802- | hsa_circ_0002863 | GFPT1 | 1.169 | 0.046229 |
| chr16:9009111-9011013- | hsa_circ_0005152 | USP7 | 1.171 | 0.011786 |
| chr4:15626875-15646280- | hsa_circ_0125593 | FBXL5 | 1.172 | 0.026813 |
| chr5:109049221-109065214+ | hsa_circ_0001519 | MAN2A1 | 1.172 | 0.001271 |
| chr8:133734286-133764243- | hsa_circ_0135800 | TMEM71 | 1.172 | 0.00572 |
| chr15:66641398-66643938- | | TIPIN | 1.172 | 0.029794 |
| chr16:58593708-58594266- | hsa_circ_0000705 | CNOT1 | 1.175 | 0.001253 |
| chr9:134381501-134381840+ | hsa_circ_0001897 | POMT1 | 1.175 | 0.011064 |
| chr5:170610199-170632616+ | hsa_circ_0003718 | RANBP17 | 1.176 | 0.018333 |
| chr2:211018219-211019335- | hsa_circ_0008459 | KANSL1L | 1.176 | 0.000956 |
| chr11:77409532-77413540- | hsa_circ_0000345 | RSF1 | 1.179 | 0.012843 |
| chr3:100013931-100016889+ | hsa_circ_0005605 | TBC1D23 | 1.181 | 0.021871 |
| chr1:235963620-235964397- | hsa_circ_0017092 | LYST | 1.182 | 0.007344 |
| chr15:50878589-50884823- | hsa_circ_0103771 | TRPM7 | 1.182 | 0.001429 |
| chr9:107485149-107485999- | | OR13D3P | 1.186 | 0.048219 |
| chr18:2694525-2698039+ | hsa_circ_0108161 | SMCHD1 | 1.186 | 0.029794 |
| chr11:118453894-118455359+ | hsa_circ_0007383 | ARCN1 | 1.187 | 0.012401 |
| chr5:142416761-142437312+ | hsa_circ_0074368 | ARHGAP26 | 1.188 | 0.002377 |
| chr17:35800606-35800763+ | hsa_circ_0006220 | TADA2A | 1.188 | 0.043761 |
| chr3:169694734-169703653+ | hsa_circ_0122662 | SEC62 | 1.191 | 0.012578 |
| chrM:8473-8625+ | | OK/SW-cl.16 | 1.193 | 0.01937 |
| chr17:26490569-26499644+ | hsa_circ_0003638 | NLK | 1.195 | 0.034766 |
| chr1:176132005-176133027- | hsa_circ_0015377 | RFWD2 | 1.196 | 0.018333 |
| chr2:68613634-68621308+ | hsa_circ_0055014 | PLEK | 1.197 | 0.001197 |
| chr16:31102096-31102663- | hsa_circ_0006719 | RP11-196G11.1 | 1.2 | 0.006526 |
| chr2:45773456-45789895- | hsa_circ_0120145 | SRBD1 | 1.2 | 0.02771 |
| chr12:66620503-66622150+ | hsa_circ_0006708 | IRAK3 | 1.202 | 0.012578 |
| chr9:79130444-79180731+ | | XLOC_007414 | 1.203 | 0.030141 |
| chr11:108199748-108202764+ | hsa_circ_0024234 | ATM | 1.203 | 0.008992 |
| chr9:100767421-100774754+ | | ANP32B | 1.204 | 0.046229 |
| chr11:118360507-118363945+ | hsa_circ_0024444 | KMT2A | 1.205 | 0.012842 |
| chr18:74561482-74583781+ | hsa_circ_0001993 | ZNF236 | 1.205 | 0.004141 |
| chr17:3969741-3976050- | hsa_circ_0004767 | ZZEF1 | 1.209 | 0.002852 |
| chr17:2297337-2298748- | hsa_circ_0106416 | MNT | 1.209 | 0.000209 |
| chr2:32602656-32626691+ | hsa_circ_0007337 | BIRC6 | 1.211 | 0.011249 |
| chr13:28597487-28602425- | hsa_circ_0100163 | FLT3 | 1.212 | 0.000188 |
| chr15:63845914-63855207+ | hsa_circ_0008153 | USP3 | 1.212 | 0.003018 |
| chr7:138203934-138235925+ | hsa_circ_0082580 | TRIM24 | 1.212 | 0.035219 |
| chrM:1680-15728+ | | TVAS5 | 1.213 | 0.025037 |
| chr2:10740979-10747437- | hsa_circ_0007344 | NOL10 | 1.213 | 0.029794 |
| chr2:37264998-37284623- | hsa_circ_0119920 | HEATR5B | 1.216 | 0.046229 |
| chrM:1680-12080+ | | TVAS5 | 1.218 | 0.009732 |
| chr1:169279208-169293738- | hsa_circ_0000156 | NME7 | 1.219 | 0.046671 |
| chr4:88005272-88012981+ | hsa_circ_0070382 | AFF1 | 1.22 | 0.030141 |
| chr3:183368084-183382827+ | hsa_circ_0122953 | KLHL24 | 1.221 | 0.026813 |
| chr9:134305477-134308181+ | hsa_circ_0001895 | PRRC2B | 1.222 | 0.018109 |
| chr17:30498062-30503232+ | hsa_circ_0008604 | RHOT1 | 1.224 | 0.029794 |
| chr4:88116476-88139339- | hsa_circ_0127243 | KLHL8 | 1.224 | 0.01937 |
| chr1:58971732-59000003- | hsa_circ_0006281 | OMA1 | 1.226 | 0.01941 |
| chr6:76344423-76380436+ | hsa_circ_0077084 | SENP6 | 1.227 | 0.048219 |
| chr8:48307538-48320523+ | hsa_circ_0136656 | SPIDR | 1.229 | 0.014293 |
| chr1:78267016-78272786+ | hsa_circ_0114202 | FAM73A | 1.23 | 0.02771 |
| chrM:8626-8785+ | | OK/SW-cl.16 | 1.234 | 0.035219 |
| chr2:32664576-32679022+ | hsa_circ_0119808 | BIRC6 | 1.237 | 0.029794 |
| chr1:175996708-176015460- | hsa_circ_0015359 | RFWD2 | 1.237 | 0.046229 |
| chrX:53641495-53642796- | hsa_circ_0007817 | HUWE1 | 1.24 | 0.001002 |
| chr20:32684463-32686439- | hsa_circ_0007938 | EIF2S2 | 1.241 | 0.005552 |
| chr10:5751493-5756170+ | hsa_circ_0003755 | C10orf18 | 1.242 | 0.029217 |
| chr17:29311635-29315124+ | hsa_circ_0003488 | RNF135 | 1.244 | 0.035219 |
| chr2:162036125-162061304+ | hsa_circ_0005227 | TANK | 1.245 | 0.008707 |
| chr7:127344893-127361454+ | hsa_circ_0003655 | SND1 | 1.246 | 0.008707 |
| chr7:138261118-138264277+ | hsa_circ_0082600 | TRIM24 | 1.247 | 0.040426 |
| chr16:56419831-56423287- | hsa_circ_0000704 | AMFR | 1.248 | 0.004141 |
| chr13:33109906-33111164- | hsa_circ_0100273 | N4BP2L2 | 1.248 | 0.003259 |
| chr11:77823696-77832220- | hsa_circ_0003615 | ALG8 | 1.249 | 0.002968 |
| chr3:105389077-105412432- | hsa_circ_0066715 | CBLB | 1.25 | 0.047316 |
| chr15:44918529-44949461- | hsa_circ_0103654 | SPG11 | 1.251 | 0.046671 |
| chr1:16891302-16893846- | | NBPF1 | 1.253 | 0.000133 |
| chr1:1647590-1647917- | | SLC35E2B | 1.255 | 0.048219 |
| chr5:153377363-153414527- | hsa_circ_0003152 | FAM114A2 | 1.255 | 0.018903 |
| chr1:210522277-210560925+ | hsa_circ_0111809 | HHAT | 1.256 | 0.035219 |
| chr12:936208-939326+ | hsa_circ_0024960 | WNK1 | 1.26 | 0.018333 |
| chr12:96692647-96694138- | hsa_circ_0007881 | CDK17 | 1.26 | 0.037082 |
| chr9:131271155-131277918+ | hsa_circ_0002675 | GLE1 | 1.261 | 0.029794 |
| chr11:47380395-47397283- | | SPI1 | 1.262 | 0.026813 |
| chr4:39734979-39779430+ | hsa_circ_0126277 | UBE2K | 1.262 | 0.035219 |
| chr5:179133259-179147561+ | hsa_circ_0075296 | CANX | 1.263 | 0.035219 |
| chr12:111923069-111924628- | hsa_circ_0005756 | ATXN2 | 1.264 | 0.029794 |
| chr8:131164982-131193126- | hsa_circ_0008934 | ASAP1 | 1.265 | 0.000119 |
| chr1:23356962-23377013+ | hsa_circ_0009061 | KDM1A | 1.265 | 9.08E-05 |
| chr1:180953813-180974599- | hsa_circ_0006796 | STX6 | 1.266 | 0.002953 |
| chr9:138773479-138780119- | hsa_circ_0138230 | CAMSAP1 | 1.271 | 0.048219 |
| chr19:14513409-14515374+ | hsa_circ_0004853 | ADGRE5 | 1.272 | 0.007916 |
| chr17:56081012-56082402- | hsa_circ_0107191 | SRSF1 | 1.273 | 0.026813 |
| chr17:78865520-78867665+ | hsa_circ_0000813 | RPTOR | 1.278 | 0.037082 |
| chr11:34111726-34112225+ | hsa_circ_0000288 | CAPRIN1 | 1.279 | 0.046229 |
| chr17:60106902-60140662- | hsa_circ_0107375 | MED13 | 1.28 | 0.007916 |
| chr4:47537526-47548879+ | hsa_circ_0126502 | ATP10D | 1.28 | 0.048219 |
| chr3:17549966-17618041- | | TBC1D5 | 1.281 | 0.046229 |
| chr7:155457869-155473602+ | hsa_circ_0001771 | RBM33 | 1.281 | 0.002649 |
| chr12:26748398-26755639- | hsa_circ_0098222 | ITPR2 | 1.282 | 0.012843 |
| chr1:230798887-230810870+ | hsa_circ_0016868 | COG2 | 1.285 | 0.046229 |
| chr21:36206707-36231875- | hsa_circ_0002360 | RUNX1 | 1.285 | 2.45E-05 |
| chr10:70530963-70532856+ | hsa_circ_0094027 | CCAR1 | 1.288 | 0.018903 |
| chr19:17387304-17387718+ | hsa_circ_0003253 | BABAM1 | 1.289 | 0.001186 |
| chr8:62546242-62566219- | hsa_circ_0009158 | ASPH | 1.29 | 0.040426 |
| chr16:2607704-2616454+ | hsa_circ_0005466 | PDPK1 | 1.29 | 0.048219 |
| chr12:104376577-104379506+ | hsa_circ_0008166 | TDG | 1.29 | 0.011249 |
| chr12:27867713-27877119+ | hsa_circ_0000384 | MRPS35 | 1.292 | 0.001286 |
| chr13:49771013-49772710+ | hsa_circ_0005263 | FNDC3A | 1.295 | 0.000427 |
| chr10:103783234-103785005- | hsa_circ_0092466 | C10orf76 | 1.296 | 0.035219 |
| chr1:155316174-155317695- | hsa_circ_0014558 | ASH1L | 1.297 | 0.046229 |
| chr6:146202764-146216113- | hsa_circ_0130935 | SHPRH | 1.297 | 0.018448 |
| chr15:66621308-66621737+ | hsa_circ_0035963 | DIS3L | 1.299 | 0.018333 |
| chr11:34101175-34111003+ | hsa_circ_0000286 | CAPRIN1 | 1.3 | 0.035219 |
| chr1:47745913-47748131- | hsa_circ_0000069 | STIL | 1.301 | 0.001197 |
| chr1:86426932-86437076- | | COL24A1 | 1.301 | 0.026813 |
| chr5:50084357-50093067+ | hsa_circ_0072440 | PARP8 | 1.302 | 0.01937 |
| chr15:75215989-75219228- | hsa_circ_0007840 | COX5A | 1.302 | 0.015529 |
| chr15:68467975-68468992+ | hsa_circ_0104358 | PIAS1 | 1.304 | 0.000706 |
| chr17:59853762-59861785- | hsa_circ_0107327 | BRIP1 | 1.305 | 0.046671 |
| chr1:145459652-145460263- | hsa_circ_0110693 | POLR3GL | 1.305 | 0.011249 |
| chr17:41201138-41209152- | hsa_circ_0043949 | BRCA1 | 1.305 | 0.046229 |
| chr15:64495281-64508912- | hsa_circ_0001955 | CSNK1G1 | 1.308 | 0.026813 |
| chr6:105192035-105233194- | hsa_circ_0004397 | HACE1 | 1.309 | 0.015529 |
| chr13:32936660-32937670+ | | BRCA2 | 1.31 | 0.018448 |
| chr19:47998807-48004006- | hsa_circ_0051692 | NAPA | 1.314 | 0.018448 |
| chr5:153413351-153414527- | hsa_circ_0001546 | FAM114A2 | 1.314 | 4.85E-05 |
| chr11:34978931-35013926+ | hsa_circ_0095769 | PDHX | 1.315 | 0.026813 |
| chr15:59204762-59205895- | hsa_circ_0003713 | SLTM | 1.315 | 0.001003 |
| chr1:235944165-235945387- | hsa_circ_0112538 | LYST | 1.315 | 0.018903 |
| chr11:57258697-57259335- | hsa_circ_0005204 | SLC43A1 | 1.316 | 0.008357 |
| chr12:83250789-83251359+ | hsa_circ_0002886 | TMTC2 | 1.318 | 0.000503 |
| chr15:44106699-44107272- | | MFAP1 | 1.32 | 0.030141 |
| chr11:128628010-128642880+ | hsa_circ_0000369 | FLI1 | 1.32 | 0.00052 |
| chr10:22997090-22997257- | | PIP4K2A | 1.321 | 0.026813 |
| chr10:103384502-103436193- | hsa_circ_0005741 | FBXW4 | 1.322 | 0.011249 |
| chr15:65959787-65962526- | hsa_circ_0035923 | DENND4A | 1.323 | 0.018333 |
| chr2:227771509-227779067+ | hsa_circ_0058497 | RHBDD1 | 1.324 | 0.000698 |
| chr7:77407655-77408131+ | hsa_circ_0134710 | RSBN1L | 1.324 | 0.021871 |
| chr10:88211724-88221044- | hsa_circ_0019005 | WAPL | 1.324 | 0.008357 |
| chr16:74493580-74497377- | hsa_circ_0003315 | GLG1 | 1.331 | 0.048219 |
| chr2:24042617-24056948- | | ATAD2B | 1.332 | 0.046229 |
| chr8:17125767-17143936+ | hsa_circ_0008555 | VPS37A | 1.332 | 0.01941 |
| chr3:48960181-48965246+ | hsa_circ_0006838 | ARIH2 | 1.333 | 0.005043 |
| chr13:114806476-114822916- | | RASA3 | 1.333 | 0.008992 |
| chr13:28748409-28794515+ | hsa_circ_0005831 | PAN3 | 1.336 | 0.000162 |
| chr16:11063018-11076848+ | hsa_circ_0004179 | CLEC16A | 1.337 | 0.002221 |
| chr6:82920531-82922510- | hsa_circ_0002041 | IBTK | 1.344 | 0.000909 |
| chr13:114817527-114822949- | hsa_circ_0007514 | RASA3 | 1.344 | 0.006034 |
| chr21:38787792-38794168+ | | DYRK1A | 1.345 | 0.006889 |
| chr4:36230204-36231267- | hsa_circ_0069399 | ARAP2 | 1.348 | 0.00023 |
| chr1:35824526-35827390+ | hsa_circ_0011536 | ZMYM4 | 1.349 | 8.94E-05 |
| chr5:137342679-137346838- | hsa_circ_0128074 | FAM13B | 1.349 | 0.046229 |
| chr4:68396533-68405652- | hsa_circ_0126834 | CENPC | 1.349 | 0.035219 |
| chr20:57014001-57016139+ | hsa_circ_0001173 | VAPB | 1.35 | 0.001286 |
| chr6:22056775-22063469+ | hsa_circ_0131484 | LINC00340 | 1.35 | 0.029794 |
| chr3:171965323-172003774+ | hsa_circ_0001362 | FNDC3B | 1.353 | 0.000792 |
| chr15:101550655-101551042+ | hsa_circ_0037078 | LRRK1 | 1.353 | 0.005552 |
| chr7:140494108-140508795- | hsa_circ_0006961 | BRAF | 1.354 | 0.004141 |
| chr4:3519761-3526778- | | LRPAP1 | 1.354 | 0.037082 |
| chr17:65941525-65944422+ | hsa_circ_0000798 | BPTF | 1.355 | 0.000101 |
| chr2:64083440-64090062+ | hsa_circ_0004227 | UGP2 | 1.356 | 0.018333 |
| chr11:118422501-118430579- | hsa_circ_0007372 | IFT46 | 1.356 | 0.001954 |
| chr1:197552282-197627499- | hsa_circ_0111647 | DENND1B | 1.356 | 0.035219 |
| chr1:111690264-111703918+ | hsa_circ_0000106 | CEPT1 | 1.357 | 0.004197 |
| chr5:39002637-39021238- | hsa_circ_0001475 | RICTOR | 1.358 | 0.048219 |
| chr11:9441958-9446809+ | hsa_circ_0004672 | IPO7 | 1.358 | 0.046671 |
| chr5:14673751-14693203+ | hsa_circ_0128263 | OTULIN | 1.359 | 0.040426 |
| chrM:1680-15647+ | | TVAS5 | 1.36 | 0.047316 |
| chr1:155340295-155429689- | hsa_circ_0014579 | ASH1L | 1.36 | 0.026813 |
| chr12:50821545-50822873+ | hsa_circ_0003873 | LARP4 | 1.36 | 0.048219 |
| chr15:73862491-73866136- | hsa_circ_0036250 | NPTN | 1.361 | 0.00517 |
| chr15:65962117-65962526- | hsa_circ_0035929 | DENND4A | 1.362 | 0.018333 |
| chr16:11940358-11944275- | hsa_circ_0037891 | RSL1D1 | 1.362 | 0.035219 |
| chr8:108296910-108359325- | | ANGPT1 | 1.364 | 0.002662 |
| chr1:145897085-145900490+ | hsa_circ_0008553 | GPR89C | 1.367 | 0.006034 |
| chr2:203817282-203820481+ | hsa_circ_0004919 | CARF | 1.368 | 0.008577 |
| chr14:35269430-35272194- | hsa_circ_0101710 | BAZ1A | 1.37 | 0.018109 |
| chr11:61133517-61135470+ | hsa_circ_0002058 | TMEM138 | 1.37 | 1.33E-05 |
| chr4:88359429-88375607+ | hsa_circ_0070396 | NUDT9 | 1.371 | 0.018448 |
| chr14:93273081-93275864+ | hsa_circ_0102931 | GOLGA5 | 1.371 | 0.024189 |
| chr7:26724355-26729981- | hsa_circ_0079658 | SKAP2 | 1.371 | 0.018333 |
| chr11:47379650-47379816- | | SPI1 | 1.373 | 0.011249 |
| chr12:112884080-112894702+ | hsa_circ_0004186 | PTPN11 | 1.373 | 0.026813 |
| chr15:50751197-50751359+ | | USP8 | 1.373 | 0.029794 |
| chr8:101271335-101287389- | hsa_circ_0135306 | RNF19A | 1.375 | 0.018333 |
| chr9:414782-418207+ | hsa_circ_0086196 | DOCK8 | 1.376 | 0.035219 |
| chr10:112635724-112642855+ | | PDCD4 | 1.376 | 0.018448 |
| chr14:105692406-105693097- | hsa_circ_0006153 | BRF1 | 1.377 | 0.029217 |
| chr20:42242490-42252685+ | hsa_circ_0060443 | IFT52 | 1.378 | 0.018448 |
| chr12:93244887-93246801- | hsa_circ_0099496 | EEA1 | 1.38 | 0.046671 |
| chr15:68434284-68466230+ | hsa_circ_0007088 | PIAS1 | 1.38 | 0.046229 |
| chr3:5212188-5216099+ | hsa_circ_0005046 | ARL8B | 1.38 | 3.33E-05 |
| chr3:160129708-160132305+ | hsa_circ_0122580 | SMC4 | 1.385 | 0.008992 |
| chr16:68224671-68225678+ | hsa_circ_0000713 | NFATC3 | 1.385 | 0.00167 |
| chr16:28112779-28113266- | hsa_circ_0038773 | XPO6 | 1.387 | 0.048219 |
| chr7:32660016-32718742- | | DPY19L1P1 | 1.388 | 0.018333 |
| chr3:17413567-17425507- | hsa_circ_0008286 | TBC1D5 | 1.391 | 0.018333 |
| chr5:132400672-132412590+ | hsa_circ_0002633 | HSPA4 | 1.391 | 0.004029 |
| chr15:52186012-52194233+ | hsa_circ_0035296 | TMOD3 | 1.393 | 0.046229 |
| chr8:18622959-18662408- | hsa_circ_0002111 | PSD3 | 1.394 | 0.003071 |
| chr5:176370336-176378578- | hsa_circ_0004490 | UIMC1 | 1.394 | 0.006011 |
| chr9:33948372-33948585- | hsa_circ_0002976 | UBAP2 | 1.397 | 0.000827 |
| chr12:65042317-65043332+ | | RASSF3 | 1.397 | 0.005873 |
| chr21:34787195-34805178+ | hsa_circ_0002660 | IFNGR2 | 1.398 | 0.004215 |
| chr12:56997143-56999111- | hsa_circ_0004843 | BAZ2A | 1.4 | 0.048219 |
| chr4:106317406-106374799- | hsa_circ_0124939 | PPA2 | 1.4 | 0.026813 |
| chr16:1793333-1798721+ | hsa_circ_0008860 | MAPK8IP3 | 1.4 | 0.035219 |
| chr6:22020568-22056919+ | hsa_circ_0075829 | CASC15 | 1.401 | 0.035219 |
| chr8:131302247-131370389- | hsa_circ_0135747 | ASAP1 | 1.405 | 0.015529 |
| chr7:77210744-77214898+ | hsa_circ_0134689 | PTPN12 | 1.407 | 0.015529 |
| chr12:82763166-82796909+ | | METTL25 | 1.407 | 0.006034 |
| chr11:111907997-111910121+ | hsa_circ_0024295 | DLAT | 1.407 | 0.015529 |
| chr15:76146727-76175765+ | hsa_circ_0036372 | UBE2Q2 | 1.408 | 0.012842 |
| chr4:39875909-39878773- | hsa_circ_0008312 | PDS5A | 1.408 | 0.046229 |
| chr20:62557895-62562375+ | | DNAJC5 | 1.408 | 0.025972 |
| chr7:23552513-23562051- | hsa_circ_0133976 | TRA2A | 1.41 | 0.026813 |
| chr22:32154532-32164849+ | hsa_circ_0062939 | DEPDC5 | 1.41 | 0.012843 |
| chr2:173433469-173460751+ | hsa_circ_0057105 | PDK1 | 1.412 | 0.005571 |
| chr1:246890194-246899357+ | hsa_circ_0112860 | SCCPDH | 1.413 | 0.018448 |
| chr16:14687158-14698083- | hsa_circ_0005627 | PARN | 1.414 | 0.007916 |
| chr5:40767568-40777688- | hsa_circ_0072352 | PRKAA1 | 1.416 | 0.009732 |
| chr3:169831148-169854453- | hsa_circ_0067896 | PHC3 | 1.418 | 0.002662 |
| chr15:35174703-35196706- | hsa_circ_0103337 | AQR | 1.418 | 0.046229 |
| chr3:183432932-183442319+ | hsa_circ_0122963 | YEATS2 | 1.42 | 0.026813 |
| chrX:139865340-139866824+ | hsa_circ_0001946 | CDR1 | 1.42 | 0.004248 |
| chr16:11868092-11873219- | hsa_circ_0037886 | ZC3H7A | 1.42 | 0.015529 |
| chr2:43725972-43755127- | hsa_circ_0002392 | THADA | 1.42 | 0.018333 |
| chr7:129297183-129330386+ | hsa_circ_0082306 | NRF1 | 1.421 | 0.029794 |
| chr18:19144164-19148055- | hsa_circ_0009110 | ESCO1 | 1.423 | 0.007465 |
| chr1:23356962-23366927+ | hsa_circ_0112433 | KDM1A | 1.424 | 0.026813 |
| chr19:1031070-1032695+ | hsa_circ_0004003 | CNN2 | 1.424 | 0.018448 |
| chr2:68364431-68368927- | hsa_circ_0120793 | WDR92 | 1.424 | 0.005043 |
| chr13:79209245-79219132- | hsa_circ_0000498 | RNF219 | 1.424 | 0.012578 |
| chr12:112939948-112944226+ | hsa_circ_0097440 | PTPN11 | 1.427 | 0.026813 |
| chr12:27135706-27143560- | hsa_circ_0098245 | TM7SF3 | 1.429 | 0.046229 |
| chr13:33306238-33309467+ | hsa_circ_0004494 | PDS5B | 1.431 | 4.83E-05 |
| chr12:118650719-118651915- | hsa_circ_0028665 | TAOK3 | 1.432 | 0.011249 |
| chr16:53288350-53308214+ | hsa_circ_0000702 | CHD9 | 1.432 | 0.007916 |
| chr17:17163615-17168295- | hsa_circ_0106281 | COPS3 | 1.432 | 0.046229 |
| chr10:1141138-1141306+ | | WDR37 | 1.433 | 0.018448 |
| chr22:30218324-30228331- | hsa_circ_0062802 | ASCC2 | 1.435 | 0.029794 |
| chr7:72302182-72304508+ | hsa_circ_0080442 | SBDSP1 | 1.435 | 0.048219 |
| chr15:59204762-59213783- | hsa_circ_0104012 | SLTM | 1.435 | 0.006889 |
| chr10:43650345-43671480+ | hsa_circ_0093655 | CSGALNACT2 | 1.435 | 0.018333 |
| chr6:118832461-118887479- | hsa_circ_0077736 | CEP85L | 1.435 | 0.002039 |
| chr7:138936702-138951149+ | hsa_circ_0001754 | UBN2 | 1.436 | 0.018448 |
| chr22:23101560-23135351+ | |  | 1.438 | 0.026813 |
| chr11:75718578-75728024+ | hsa_circ_0023641 | UVRAG | 1.439 | 0.004215 |
| chr5:139865174-139885454+ | hsa_circ_0074265 | ANKHD1 | 1.44 | 0.005209 |
| chr14:32586346-32621734+ | hsa_circ_0005236 | ARHGAP5 | 1.444 | 0.026813 |
| chr2:201997752-202014558+ | hsa_circ_0007530 | CFLAR | 1.446 | 0.005209 |
| chr2:122260743-122287901- | hsa_circ_0002374 | CLASP1 | 1.447 | 0.00052 |
| chr18:54423814-54426184+ | hsa_circ_0000852 | WDR7 | 1.447 | 0.002968 |
| chr17:40879653-40882936- | hsa_circ_0043898 | EZH1 | 1.447 | 0.00167 |
| chr19:3660964-3661999- | hsa_circ_0000871 | PIP5K1C | 1.448 | 0.001291 |
| chr2:24777258-24778924+ | hsa_circ_0119554 | NCOA1 | 1.449 | 0.018333 |
| chr17:16062074-16097953- | hsa_circ_0042179 | NCOR1 | 1.451 | 0.046229 |
| chr19:23556544-23557566- | hsa_circ_0109324 | ZNF91 | 1.457 | 0.006889 |
| chr21:38480692-38520947+ | hsa_circ_0115970 | TTC3 | 1.458 | 0.008469 |
| chr6:3076998-3078169+ | hsa_circ_0001571 | RIPK1 | 1.459 | 0.000178 |
| chr4:3156020-3158926+ | hsa_circ_0126186 | HTT | 1.459 | 0.011249 |
| chr20:8720991-8746005+ | hsa_circ_0115600 | PLCB1 | 1.46 | 0.008992 |
| chr13:74419964-74420510- | hsa_circ_0005783 | KLF12 | 1.46 | 0.018448 |
| chr17:41219625-41226538- | hsa_circ_0043954 | BRCA1 | 1.46 | 0.003208 |
| chr14:96807859-96813678- | hsa_circ_0002223 | ATG2B | 1.463 | 0.008084 |
| chr14:35438376-35441270- | hsa_circ_0008568 | RP11-85K15.2 | 1.464 | 5.46E-05 |
| chrX:14868627-14883702- | hsa_circ_0089912 | FANCB | 1.464 | 0.008992 |
| chr1:146747017-146757164+ | hsa_circ_0013953 | CHD1L | 1.464 | 0.005043 |
| chr9:94058303-94118437- | hsa_circ_0003518 | AUH | 1.466 | 0.046229 |
| chr9:127064215-127089724+ | hsa_circ_0008383 | NEK6 | 1.467 | 0.018448 |
| chr19:1147308-1154401- | hsa_circ_0009130 | SBNO2 | 1.468 | 0.018333 |
| chr22:38641941-38644025- | hsa_circ_0005243 | TMEM184B | 1.469 | 0.012842 |
| chr21:34713305-34721849+ | hsa_circ_0115838 | IFNAR1 | 1.472 | 0.018333 |
| chr9:271627-289581+ | hsa_circ_0086188 | DOCK8 | 1.472 | 0.035996 |
| chr9:134034770-134039531+ | hsa_circ_0089166 | NUP214 | 1.472 | 0.035219 |
| chr15:85081763-85098301- | hsa_circ_0104778 | UBE2Q2P1 | 1.473 | 0.005571 |
| chr2:175957789-175986268- | hsa_circ_0057145 | ATF2 | 1.476 | 0.009732 |
| chr6:47547121-47577040+ | | CD2AP | 1.476 | 0.035219 |
| chrM:13847-13999- | | JA760602 | 1.478 | 0.048219 |
| chr15:52697484-52702673- | hsa_circ_0035336 | MYO5A | 1.478 | 0.035219 |
| chr15:66030045-66048810- | hsa_circ_0035952 | DENND4A | 1.479 | 0.035219 |
| chr14:45700344-45706924- | hsa_circ_0101888 | MIS18BP1 | 1.48 | 0.026813 |
| chr2:10784446-10808849- | hsa_circ_0000977 | NOL10 | 1.481 | 0.035219 |
| chr15:55516087-55520910- | | RAB27A | 1.481 | 0.009732 |
| chr5:137897269-137903411- | hsa_circ_0008356 | HSPA9 | 1.481 | 0.035219 |
| chr18:18586312-18588155- | hsa_circ_0108006 | ROCK1 | 1.482 | 0.011249 |
| chr9:139341307-139342613- | hsa_circ_0138245 | SEC16A | 1.482 | 0.002578 |
| chr5:82491589-82500740+ | hsa_circ_0129853 | XRCC4 | 1.482 | 0.008469 |
| chr5:134010305-134018172+ | hsa_circ_0073970 | SEC24A | 1.482 | 0.035219 |
| chr8:142262490-142263023- | | SLC45A4 | 1.482 | 0.004215 |
| chr8:133726241-133769540- | | TMEM71 | 1.483 | 0.035219 |
| chr7:129824024-129832663- | hsa_circ_0082356 | TMEM209 | 1.484 | 0.048219 |
| chr17:62557618-62559084- | hsa_circ_0008099 | SMURF2 | 1.485 | 0.004236 |
| chr1:155348072-155385714- | hsa_circ_0002897 | ASH1L | 1.486 | 0.011249 |
| chr7:158531714-158560465- | hsa_circ_0133836 | ESYT2 | 1.488 | 0.018448 |
| chr9:37302185-37327831+ | hsa_circ_0087051 | ZCCHC7 | 1.488 | 0.018448 |
| chr18:48444480-48458730+ | hsa_circ_0047700 | ME2 | 1.489 | 0.008084 |
| chr9:5944871-5988545- | hsa_circ_0138868 | KIAA2026 | 1.49 | 0.018903 |
| chr6:108543438-108544249- | | SNX3 | 1.491 | 0.008992 |
| chr13:114806476-114822949- | hsa_circ_0004790 | RASA3 | 1.491 | 0.000546 |
| chr15:49528048-49620327+ | hsa_circ_0005337 | GALK2 | 1.491 | 0.004215 |
| chr2:39294769-39302603- | hsa_circ_0119995 | SOS1 | 1.492 | 0.035219 |
| chr9:134019667-134022971+ | hsa_circ_0089157 | NUP214 | 1.493 | 0.026813 |
| chr2:55559702-55562300- | hsa_circ_0054618 | CCDC88A | 1.493 | 1.02E-05 |
| chr1:51121114-51210447- | hsa_circ_0004619 | FAF1 | 1.493 | 0.008357 |
| chr22:46096162-46136418+ | hsa_circ_0001246 | ATXN10 | 1.493 | 0.020111 |
| chr22:50810449-50832564+ | hsa_circ_0001258 | PPP6R2 | 1.495 | 0.004248 |
| chr10:89653782-89655534+ | hsa_circ_0094342 | PTEN | 1.496 | 0.02771 |
| chr19:11168934-11170863+ | hsa_circ_0005973 | SMARCA4 | 1.498 | 0.018448 |
| chr3:155545971-155560408- | hsa_circ_0122480 | SLC33A1 | 1.501 | 0.004215 |
| chr1:51868107-51913807- | hsa_circ_0012434 | EPS15 | 1.507 | 0.009732 |
| chr17:4575409-4575567- | | PELP1 | 1.508 | 0.018448 |
| chr1:44876256-44878394+ | | RNF220 | 1.509 | 0.000823 |
| chr20:60572606-60589763- | hsa_circ_0115543 | TAF4 | 1.509 | 0.005043 |
| chr9:33944363-33956144- | hsa_circ_0001846 | UBAP2 | 1.51 | 0.001028 |
| chr19:34921481-34929671+ | hsa_circ_0006987 | UBA2 | 1.511 | 4.17E-06 |
| chr12:110463547-110467440+ | hsa_circ_0009075 | ANKRD13A | 1.512 | 0.015529 |
| chr3:33441681-33467233- | hsa_circ_0064735 | UBP1 | 1.513 | 0.018333 |
| chr8:17123416-17143936+ | hsa_circ_0136018 | VPS37A | 1.513 | 0.012842 |
| chr5:74981032-75003700- | hsa_circ_0073058 | POC5 | 1.517 | 0.018448 |
| chr8:124089351-124113247+ | hsa_circ_0135635 | TBC1D31 | 1.518 | 0.001258 |
| chr6:129932678-129939997- | hsa_circ_0006887 | ARHGAP18 | 1.52 | 0.018333 |
| chr11:62295055-62295639- | | AHNAK | 1.521 | 0.015529 |
| chr9:86294690-86301070- | hsa_circ_0008207 | UBQLN1 | 1.522 | 5.72E-05 |
| chr2:28974904-29022299+ | hsa_circ_0119705 | PPP1CB | 1.522 | 0.01937 |
| chr2:158655939-158675071- | hsa_circ_0004955 | ACVR1 | 1.524 | 0.005873 |
| chr5:75737218-75758139+ | | IQGAP2 | 1.524 | 0.015529 |
| chr4:129042981-129083472+ | hsa_circ_0125310 | LARP1B | 1.525 | 0.018448 |
| chr6:157150361-157222659+ | hsa_circ_0008519 | ARID1B | 1.526 | 0.015529 |
| chr16:53967897-53971206+ | hsa_circ_0007068 | FTO | 1.526 | 0.005571 |
| chr10:12277047-12280484+ | hsa_circ_0092974 | CDC123 | 1.528 | 0.002578 |
| chr6:108984658-108986092+ | hsa_circ_0006404 | FOXO3 | 1.528 | 5.82E-05 |
| chr8:103283362-103300494- | hsa_circ_0085197 | UBR5 | 1.528 | 0.029217 |
| chr10:103552596-103567658- | hsa_circ_0019606 | MGEA5 | 1.529 | 0.002161 |
| chr4:151719233-151738409- | hsa_circ_0008618 | LRBA | 1.529 | 0.00145 |
| chr9:82319698-82324614+ | hsa_circ_0087302 | TLE4 | 1.529 | 0.015529 |
| chr3:56606365-56628056+ | hsa_circ_0124289 | CCDC66 | 1.53 | 0.015529 |
| chr1:51001041-51061888- | hsa_circ_0012397 | FAF1 | 1.53 | 0.007916 |
| chr1:203816312-203816815+ | hsa_circ_0016115 | ZC3H11A | 1.531 | 0.001489 |
| chr15:50288874-50294420- | | ATP8B4 | 1.532 | 0.009732 |
| chr13:33222891-33253066+ | hsa_circ_0029941 | PDS5B | 1.534 | 0.003054 |
| chr2:136389288-136437894+ | hsa_circ_0056567 | R3HDM1 | 1.535 | 0.001295 |
| chr15:74911538-74912566+ | hsa_circ_0036287 | CLK3 | 1.535 | 0.046229 |
| chr3:152017194-152018156+ | hsa_circ_0001348 | MBNL1 | 1.537 | 9.88E-05 |
| chr10:12129534-12162266+ | hsa_circ_0017735 | DHTKD1 | 1.537 | 0.009732 |
| chr19:41774128-41787180+ | hsa_circ_0003079 | HNRNPUL1 | 1.537 | 0.000807 |
| chr13:53001124-53009017- | hsa_circ_0100738 | VPS36 | 1.538 | 0.035219 |
| chr19:11289021-11289396- | hsa_circ_0008554 | KANK2 | 1.539 | 0.018333 |
| chr1:44877653-44881895+ | | RNF220 | 1.539 | 7.80E-07 |
| chrM:9032-9186+ | | OK/SW-cl.16 | 1.54 | 0.012401 |
| chr19:34941170-34957919+ | hsa_circ_0050547 | UBA2 | 1.541 | 0.001756 |
| chr2:112817554-112873780+ | hsa_circ_0117017 | TMEM87B | 1.542 | 0.001686 |
| chr7:100410369-100410830- | hsa_circ_0001730 | EPHB4 | 1.544 | 0.003957 |
| chr8:95549331-95550574- | hsa_circ_0006896 | KIAA1429 | 1.544 | 0.002039 |
| chr22:19948722-19951822+ | hsa_circ_0062277 | COMT | 1.545 | 0.035219 |
| chr5:126140468-126147590+ | hsa_circ_0007726 | LMNB1 | 1.547 | 0.005571 |
| chr5:137654908-137665336- | | CDC25C | 1.547 | 0.018448 |
| chr12:102433646-102439897- | hsa_circ_0002349 | CCDC53 | 1.548 | 0.000772 |
| chrX:47082951-47092618+ | | USP11 | 1.548 | 0.037082 |
| chr17:54921380-54923170+ | hsa_circ_0044708 | DGKE | 1.55 | 0.018333 |
| chr4:83793097-83796975- | hsa_circ_0003549 | SEC31A | 1.552 | 3.71E-05 |
| chr7:40027198-40027857+ | hsa_circ_0079929 | CDK13 | 1.552 | 0.004236 |
| chr3:142183939-142189025- | hsa_circ_0122257 | ATR | 1.552 | 0.026813 |
| chr7:104702611-104722244+ | hsa_circ_0132875 | KMT2E | 1.555 | 0.002968 |
| chr10:28872328-28879761+ | hsa_circ_0018054 | WAC | 1.555 | 0.002039 |
| chr7:23015829-23023664- | hsa_circ_0001971 | FAM126A | 1.557 | 0.01718 |
| chr17:30207592-30214332- | hsa_circ_0000757 | UTP6 | 1.559 | 0.009732 |
| chr2:101874253-101879153+ | hsa_circ_0002401 | CNOT11 | 1.56 | 0.003022 |
| chr14:31637504-31638674- | hsa_circ_0101617 | HECTD1 | 1.56 | 0.00517 |
| chr15:64404772-64415745+ | hsa_circ_0002178 | SNX1 | 1.561 | 0.018448 |
| chr22:50181038-50198008- | hsa_circ_0063870 | BRD1 | 1.561 | 0.026813 |
| chr14:76633006-76644385+ | hsa_circ_0005212 | GPATCH2L | 1.561 | 0.046229 |
| chr11:65202524-65211534+ | | NEAT1 | 1.562 | 0.008992 |
| chr10:103902802-103904847+ | hsa_circ_0019682 | PPRC1 | 1.563 | 0.005209 |
| chr15:52192352-52194233+ | hsa_circ_0004773 | TMOD3 | 1.563 | 0.018448 |
| chr8:12955908-12958279- | hsa_circ_0083383 | DLC1 | 1.564 | 0.018448 |
| chr6:137015278-137018525- | hsa_circ_0006554 | MAP3K5 | 1.565 | 0.000591 |
| chr6:150059779-150063706- | hsa_circ_0078241 | NUP43 | 1.565 | 0.001577 |
| chr14:31070976-31077275+ | | G2E3 | 1.565 | 0.026813 |
| chr8:29927158-29927575- | hsa_circ_0004283 | SARAF | 1.566 | 0.035219 |
| chr10:70547684-70548085+ | hsa_circ_0002681 | CCAR1 | 1.567 | 0.000997 |
| chrM:13450-13640+ | | MTND5 | 1.571 | 0.035219 |
| chr12:90035935-90049884- | hsa_circ_0000426 | ATP2B1 | 1.571 | 0.002578 |
| chr10:101658500-101659823- | hsa_circ_0019494 | DNMBP | 1.572 | 0.008992 |
| chr16:4700366-4707364+ | hsa_circ_0007032 | MGRN1 | 1.575 | 0.00134 |
| chr6:47471016-47472494+ | hsa_circ_0007936 | CD2AP | 1.575 | 1.23E-05 |
| chr6:18160114-18171710+ | hsa_circ_0131408 | KDM1B | 1.576 | 0.001028 |
| chr7:91700219-91715000+ | hsa_circ_0135024 | AKAP9 | 1.576 | 0.015529 |
| chr4:1341878-1349033+ | | UVSSA | 1.576 | 0.003022 |
| chr1:115005726-115006178- | hsa_circ_0013637 | TRIM33 | 1.577 | 0.004215 |
| chr8:141582911-141616013- | hsa_circ_0135888 | AGO2 | 1.577 | 0.000242 |
| chr7:142131751-142149267+ | | TCRBV22S1A2N1T | 1.581 | 0.005209 |
| chr11:34084599-34084910+ | hsa_circ_0095748 | CAPRIN1 | 1.585 | 0.00517 |
| chr17:28003838-28011702- | hsa_circ_0042818 | SSH2 | 1.591 | 0.006889 |
| chr12:51689580-51690948- | | BIN2 | 1.591 | 0.011249 |
| chr5:176468127-176477937+ | hsa_circ_0001559 | ZNF346 | 1.593 | 0.00157 |
| chr3:132337478-132350323- | hsa_circ_0005875 | ACAD11 | 1.593 | 0.018448 |
| chr16:23113640-23131567- | hsa_circ_0003672 | USP31 | 1.593 | 0.035219 |
| chr6:42790534-42797910+ | hsa_circ_0076410 | GLTSCR1L | 1.594 | 0.00517 |
| chr19:49416268-49416821+ | hsa_circ_0002084 | NUCB1 | 1.595 | 0.018333 |
| chr20:62421174-62422143- | hsa_circ_0061179 | ZBTB46 | 1.595 | 0.007916 |
| chr12:89860547-89866052- | hsa_circ_0027702 | POC1B | 1.595 | 0.030141 |
| chr3:67546222-67548719- | hsa_circ_0003060 | SUCLG2 | 1.596 | 0.001788 |
| chr7:77236552-77261742+ | hsa_circ_0080850 | PTPN12 | 1.596 | 0.006889 |
| chr2:68717322-68794519+ | hsa_circ_0055021 | APLF | 1.597 | 0.018448 |
| chr6:31239010-31324103- | | HLA-C | 1.598 | 1.07E-05 |
| chr10:82266984-82269227+ | hsa_circ_0008856 | TSPAN14 | 1.599 | 0.00517 |
| chr5:159462147-159478190+ | hsa_circ_0128465 | TTC1 | 1.6 | 0.046229 |
| chr11:65268840-65269225+ | | MALAT1 | 1.602 | 0.018448 |
| chr6:155063090-155099179+ | hsa_circ_0078357 | SCAF8 | 1.603 | 0.006889 |
| chr4:42553215-42558057- | hsa_circ_0069618 | ATP8A1 | 1.605 | 0.003022 |
| chr6:158994452-159029782+ | hsa_circ_0131214 | TMEM181 | 1.607 | 0.000827 |
| chr1:52289336-52299842- | hsa_circ_0007430 | NRD1 | 1.607 | 0.018333 |
| chrM:8928-9089+ | | OK/SW-cl.16 | 1.608 | 6.26E-07 |
| chr12:133428204-133430159- | hsa_circ_0029589 | CHFR | 1.609 | 0.018448 |
| chr16:66764015-66766408- | hsa_circ_0000706 | DYNC1LI2 | 1.609 | 0.000706 |
| chr6:16614591-16615701- | | ATXN1 | 1.609 | 0.009732 |
| chr5:77711344-77717784+ | hsa_circ_0002186 | SCAMP1 | 1.611 | 0.006889 |
| chr1:9931245-9938047- | hsa_circ_0009674 | CTNNBIP1 | 1.611 | 0.000977 |
| chr2:234343026-234347025+ | hsa_circ_0058764 | DGKD | 1.612 | 0.018448 |
| chr1:1586823-1650894- | hsa_circ_0000005 | CDK11B | 1.613 | 0.001788 |
| chr2:32339707-32341281+ | hsa_circ_0002795 | SPAST | 1.613 | 0.002968 |
| chr1:89206671-89251896+ | hsa_circ_0013093 | PKN2 | 1.613 | 0.000129 |
| chr11:82989769-82991303- | hsa_circ_0023865 | CCDC90B | 1.614 | 0.015529 |
| chr17:3608392-3608939- | hsa_circ_0106744 | GSE61474_XLOC_026870 | 1.615 | 0.0012 |
| chr15:58913670-58920082- | hsa_circ_0035450 | ADAM10 | 1.617 | 0.011249 |
| chr14:91211136-91252672- | hsa_circ_0102857 | TTC7B | 1.617 | 0.018448 |
| chr2:144193170-144276933+ | hsa_circ_0117500 | ARHGAP15 | 1.617 | 0.012401 |
| chr13:32910402-32921033+ | hsa_circ_0100252 | BRCA2 | 1.62 | 0.026813 |
| chr3:171426545-171443866- | hsa_circ_0067960 | PLD1 | 1.62 | 0.002662 |
| chr3:32483332-32496034+ | hsa_circ_0007783 | CMTM7 | 1.621 | 0.003208 |
| chr1:117944808-118009049+ | hsa_circ_0000120 | MAN1A2 | 1.625 | 0.018333 |
| chrM:8626-8776+ | | OK/SW-cl.16 | 1.626 | 0.00044 |
| chr2:24360779-24369956+ | hsa_circ_0119531 | FAM228B | 1.626 | 0.002039 |
| chr19:11548697-11548945+ | hsa_circ_0049462 | PRKCSH | 1.627 | 0.018448 |
| chr15:43242460-43258484- | hsa_circ_0008546 | UBR1 | 1.628 | 0.000785 |
| chr6:31234646-31319732- | | HLA-C | 1.629 | 0.003208 |
| chr2:107051093-107051658- | | RGPD3 | 1.63 | 0.00517 |
| chr11:108544194-108564327+ | hsa_circ_0024243 | DDX10 | 1.631 | 0.009732 |
| chr9:5689959-5720750+ | hsa_circ_0005707 | RIC1 | 1.633 | 0.018448 |
| chr1:247039339-247040604- | hsa_circ_0006097 | AHCTF1 | 1.633 | 0.001295 |
| chr3:196857398-196876667- | hsa_circ_0123252 | DLG1 | 1.634 | 0.008992 |
| chr10:104851321-104855737- | | NT5C2 | 1.635 | 0.005209 |
| chr2:128520636-128528578- | hsa_circ_0003192 | WDR33 | 1.636 | 0.001535 |
| chr3:50102464-50106197+ | | RBM6 | 1.636 | 0.006889 |
| chr12:124904503-124915333- | hsa_circ_0029308 | NCOR2 | 1.637 | 0.005873 |
| chr13:50601343-50619368- | | DLEU2 | 1.637 | 0.015529 |
| chr12:69044180-69048032+ | hsa_circ_0027464 | RAP1B | 1.638 | 2.07E-06 |
| chr17:80863812-80869665+ | hsa_circ_0046580 | TBCD | 1.638 | 0.000427 |
| chr2:197021163-197028151- | hsa_circ_0057569 | STK17B | 1.638 | 0.000965 |
| chr3:48491443-48493305+ | hsa_circ_0124003 | ATRIP | 1.646 | 0.009732 |
| chr2:171884849-171902872- | hsa_circ_0004442 | TLK1 | 1.646 | 6.50E-05 |
| chr18:54291519-54293688- | hsa_circ_0108673 | TXNL1 | 1.647 | 0.002766 |
| chr16:14720963-14721193- | hsa_circ_0002771 | PARN | 1.647 | 0.015529 |
| chr4:129857810-129869725- | | SCLT1 | 1.647 | 0.018448 |
| chr15:94983405-95001475+ | hsa_circ_0003333 | MCTP2 | 1.649 | 0.000672 |
| chr7:2482182-2483381+ | | AC004840.9 | 1.649 | 0.018448 |
| chr21:40630413-40646398- | hsa_circ_0061744 | BRWD1 | 1.651 | 0.002766 |
| chr2:55250507-55250695- | | RTN4 | 1.653 | 0.015529 |
| chr9:125616231-125621381- | hsa_circ_0088370 | RC3H2 | 1.654 | 0.048219 |
| chr9:5968019-5988545- | hsa_circ_0138872 | KIAA2026 | 1.655 | 0.000106 |
| chr12:133291444-133294706+ | hsa_circ_0029545 | PGAM5 | 1.656 | 0.008707 |
| chr4:3514406-3526778- | | LRPAP1 | 1.66 | 0.035219 |
| chr6:149893421-149903739+ | hsa_circ_0131014 | GINM1 | 1.661 | 0.008992 |
| chrM:13847-13999+ | | MTND5 | 1.663 | 8.15E-05 |
| chr9:79852918-79853302+ | hsa_circ_0087247 | VPS13A | 1.663 | 3.92E-05 |
| chr11:111948939-111951282+ | hsa_circ_0094994 | C11orf57 | 1.664 | 0.003022 |
| chr11:62303417-62304039- | hsa_circ_0008194 | AHNAK | 1.665 | 0.011249 |
| chr7:32584306-32594208+ | hsa_circ_0001694 | AVL9 | 1.667 | 0.000591 |
| chr21:38792601-38868560+ | hsa_circ_0061694 | DYRK1A | 1.67 | 0.003022 |
| chr4:170501993-170511960- | hsa_circ_0071434 | NEK1 | 1.67 | 0.004215 |
| chr14:31602444-31602881- | hsa_circ_0031485 | HECTD1 | 1.673 | 0.000242 |
| chr12:32133812-32140256+ | hsa_circ_0025836 | KIAA1551 | 1.674 | 7.41E-05 |
| chr7:959605-966280- | hsa_circ_0079091 | ADAP1 | 1.675 | 0.035219 |
| chr8:38803642-38814904+ | hsa_circ_0084029 | PLEKHA2 | 1.675 | 0.008992 |
| chr20:30370052-30385318+ | hsa_circ_0003209 | TPX2 | 1.676 | 0.046229 |
| chr11:1000432-1003804+ | hsa_circ_0020749 | AP2A2 | 1.676 | 0.002578 |
| chr7:127975597-127976096- | hsa_circ_0082171 | RBM28 | 1.677 | 0.002766 |
| chr19:38229804-38231095- | hsa_circ_0109570 | ZNF573 | 1.679 | 0.00044 |
| chr6:156415418-156429747- | |  | 1.68 | 0.003022 |
| chr11:78270585-78282489- | hsa_circ_0023815 | NARS2 | 1.681 | 0.018448 |
| chr19:5131087-5138081+ | hsa_circ_0048712 | KDM4B | 1.683 | 0.012401 |
| chr2:114697536-114699936+ | hsa_circ_0008712 | ACTR3 | 1.683 | 0.01941 |
| chr14:75245150-75249028+ | | YLPM1 | 1.684 | 0.000965 |
| chr12:64038183-64041145- | hsa_circ_0007104 | DPY19L2 | 1.684 | 0.000597 |
| chr4:54280782-54310270+ | hsa_circ_0001412 | FIP1L1 | 1.684 | 0.000965 |
| chr8:42914235-42932507+ | hsa_circ_0084171 | FNTA | 1.685 | 0.008992 |
| chr3:179447977-179448497+ | hsa_circ_0122834 | USP13 | 1.686 | 0.009732 |
| chr16:85701746-85704704+ | hsa_circ_0040738 | GSE1 | 1.686 | 0.00517 |
| chr2:171910271-171939362- | | TLK1 | 1.686 | 0.002766 |
| chr6:76368978-76388643+ | hsa_circ_0132306 | SENP6 | 1.686 | 0.000336 |
| chrX:130883334-130919286- | hsa_circ_0091538 | LOC286467 | 1.688 | 0.001577 |
| chr10:14961737-14977563- | hsa_circ_0004201 | DCLRE1C | 1.689 | 0.009732 |
| chr1:246754814-246755243+ | hsa_circ_0017310 | CNST | 1.69 | 0.000132 |
| chr1:93691890-93698150+ | hsa_circ_0013200 | CCDC18 | 1.69 | 0.035219 |
| chr12:121220458-121229360- | hsa_circ_0004479 | SPPL3 | 1.692 | 1.23E-05 |
| chr18:20516717-20529676+ | hsa_circ_0108054 | RBBP8 | 1.692 | 0.026813 |
| chr14:91804349-91810001- | hsa_circ_0102882 | CCDC88C | 1.692 | 0.046229 |
| chr15:76152219-76175765+ | hsa_circ_0036374 | UBE2Q2 | 1.695 | 0.001577 |
| chr1:224553581-224559125+ | hsa_circ_0000190 | CNIH4 | 1.696 | 0.000965 |
| chr1:93648917-93659301+ | hsa_circ_0114513 | CCDC18 | 1.697 | 0.048219 |
| chr3:196778448-196793625- | hsa_circ_0123233 | DLG1 | 1.699 | 0.001756 |
| chr3:184642650-184742177+ | | VPS8 | 1.699 | 0.00517 |
| chr11:120916383-120918376+ | hsa_circ_0024656 | TBCEL | 1.7 | 0.001489 |
| chr16:21327205-21328447+ | hsa_circ_0002855 | CRYM-AS1 | 1.7 | 0.003208 |
| chr20:13509080-13561628- | hsa_circ_0059469 | TASP1 | 1.701 | 0.009732 |
| chr4:129919029-129925031- | hsa_circ_0008426 | SCLT1 | 1.701 | 0.002039 |
| chr3:182665026-182681837- | hsa_circ_0122912 | DCUN1D1 | 1.701 | 0.026813 |
| chr15:51743842-51747425- | hsa_circ_0035277 | DMXL2 | 1.702 | 0.01937 |
| chr10:73887840-73892939- | hsa_circ_0008195 | ASCC1 | 1.704 | 0.035219 |
| chr1:8601273-8674745- | hsa_circ_0008501 | RERE | 1.708 | 5.82E-05 |
| chr16:28177829-28181230- | hsa_circ_0008223 | XPO6 | 1.709 | 6.08E-05 |
| chr22:38894090-38897285- | hsa_circ_0063331 | DDX17 | 1.71 | 0.005209 |
| chr15:77544709-77576372- | hsa_circ_0104613 | PEAK1 | 1.711 | 0.006889 |
| chr10:22193451-22209892- | hsa_circ_0004306 | DNAJC1 | 1.711 | 0.000363 |
| chr11:46455024-46456587- | hsa_circ_0021843 | AMBRA1 | 1.712 | 0.009732 |
| chr6:18160114-18163155+ | hsa_circ_0131407 | KDM1B | 1.72 | 0.035219 |
| chr7:69364272-69364484+ | hsa_circ_0080414 | AUTS2 | 1.72 | 0.002662 |
| chr9:36597219-36643080+ | hsa_circ_0007658 | MELK | 1.724 | 0.000135 |
| chr8:71033537-71057083- | hsa_circ_0137055 | NCOA2 | 1.724 | 0.005209 |
| chrX:130845679-130919286- | | LOC286467 | 1.725 | 0.00035 |
| chr5:137288317-137290065- | hsa_circ_0008177 | FAM13B | 1.725 | 9.99E-06 |
| chr13:95813443-95822882- | hsa_circ_0006659 | ABCC4 | 1.727 | 0.035219 |
| chr7:138951079-138957186+ | hsa_circ_0005594 | UBN2 | 1.728 | 0.000965 |
| chr5:98215256-98217079- | hsa_circ_0130104 | CHD1 | 1.734 | 0.000539 |
| chr7:104678573-104681470+ | hsa_circ_0007395 | KMT2E | 1.734 | 8.06E-05 |
| chr1:114377518-114397671- | | PTPN22 | 1.735 | 0.002578 |
| chr16:31308835-31309275+ | hsa_circ_0000692 | ITGAM | 1.736 | 0.000239 |
| chr17:53108529-53124515+ | hsa_circ_0107139 | STXBP4 | 1.736 | 0.008992 |
| chr17:37646810-37649143+ | hsa_circ_0106792 | CDK12 | 1.738 | 0.002578 |
| chr1:26594974-26596105+ | hsa_circ_0112926 | CEP85 | 1.739 | 0.000823 |
| chr11:28098554-28113060- | hsa_circ_0007496 | KIF18A | 1.741 | 0.001756 |
| chr12:100598718-100599535+ | hsa_circ_0096948 | ACTR6 | 1.741 | 0.009732 |
| chr4:186096940-186097205- | hsa_circ_0004697 | CFAP97 | 1.741 | 0.018448 |
| chr16:11868092-11876244- | hsa_circ_0005394 | ZC3H7A | 1.742 | 0.026813 |
| chr17:19823349-19827830- | hsa_circ_0106367 | AKAP10 | 1.748 | 0.026813 |
| chr17:30267305-30293215+ | hsa_circ_0042963 | SUZ12 | 1.748 | 0.003039 |
| chr1:193038164-193046180+ | hsa_circ_0111562 | TROVE2 | 1.748 | 0.003022 |
| chr10:35302627-35320547- | hsa_circ_0093600 | CUL2 | 1.749 | 0.035219 |
| chr17:30498062-30510291+ | hsa_circ_0106601 | RHOT1 | 1.749 | 0.003657 |
| chr16:3841982-3843627- | hsa_circ_0105404 | CREBBP | 1.752 | 0.002766 |
| chr15:85180578-85184493+ | hsa_circ_0006899 | SCAND2P | 1.753 | 0.005209 |
| chr6:99347144-99365595- | hsa_circ_0132765 | FBXL4 | 1.755 | 0.015529 |
| chrX:79955456-79960345- | hsa_circ_0007974 | BRWD3 | 1.755 | 0.01937 |
| chr4:166141086-166160051+ | hsa_circ_0071374 | KLHL2 | 1.756 | 0.000823 |
| chrM:14068-14413- | | JA760602 | 1.757 | 0.002039 |
| chr11:77386081-77404656- | hsa_circ_0023701 | RSF1 | 1.758 | 0.000129 |
| chr7:23023563-23030758- | hsa_circ_0001682 | FAM126A | 1.759 | 0.002766 |
| chr8:67504675-67514758- | hsa_circ_0005883 | MYBL1 | 1.76 | 0.001577 |
| chr5:64084778-64100213+ | hsa_circ_0072654 | CWC27 | 1.76 | 0.000223 |
| chr9:97717459-97741709+ | hsa_circ_0139391 | C9orf3 | 1.76 | 0.035219 |
| chr19:19407798-19408153- | hsa_circ_0004066 | SUGP1 | 1.761 | 0.000965 |
| chr1:174241552-174274265+ | hsa_circ_0005089 | RABGAP1L | 1.761 | 0.003022 |
| chr10:93711160-93713630+ | hsa_circ_0019122 | BTAF1 | 1.762 | 0.003022 |
| chr5:139916923-139921866+ | hsa_circ_0008322 | ANKHD1-EIF4EBP3 | 1.762 | 0.011249 |
| chr20:47707279-47711500+ | hsa_circ_0060762 | CSE1L | 1.763 | 0.000363 |
| chr12:93192668-93196510- | hsa_circ_0099481 | EEA1 | 1.763 | 0.005209 |
| chr3:171944661-171965566+ | hsa_circ_0067984 | FNDC3B | 1.766 | 0.035219 |
| chr2:61725808-61761038- | hsa_circ_0054894 | XPO1 | 1.766 | 0.011249 |
| chr4:6925100-6969151+ | hsa_circ_0069101 | TBC1D14 | 1.768 | 0.00043 |
| chr14:91759396-91759801- | | CCDC88C | 1.768 | 0.003039 |
| chr17:77073512-77073946+ | hsa_circ_0008114 | ENGASE | 1.77 | 0.002161 |
| chr5:130857083-130883883- | hsa_circ_0003544 | RAPGEF6 | 1.771 | 0.011249 |
| chr1:150720253-150730456- | hsa_circ_0110764 | CTSS | 1.772 | 0.046229 |
| chr11:9308002-9321248- | hsa_circ_0021156 | TMEM41B | 1.773 | 0.018448 |
| chr6:70447834-70500364- | hsa_circ_0132149 | LMBRD1 | 1.774 | 0.003022 |
| chr2:191765290-191796363+ | hsa_circ_0057523 | GLS | 1.774 | 0.002847 |
| chr15:75706546-75715164- | hsa_circ_0036354 | SIN3A | 1.776 | 0.002766 |
| chr14:39746138-39748741+ | hsa_circ_0000530 | CTAGE5 | 1.777 | 1.73E-05 |
| chr20:34430495-34435356+ | hsa_circ_0005163 | PHF20 | 1.777 | 0.035219 |
| chr16:11988811-11990642- | hsa_circ_0002161 | GSPT1 | 1.78 | 0.000772 |
| chr11:18105082-18111057- | hsa_circ_0002778 | SAAL1 | 1.781 | 4.85E-05 |
| chr3:15073886-15076316+ | hsa_circ_0064460 | NR2C2 | 1.782 | 0.011249 |
| chr7:5778907-5781446- | hsa_circ_0007501 | RNF216 | 1.783 | 0.018448 |
| chr7:30395344-30405911+ | hsa_circ_0134083 | G075174 | 1.784 | 0.000288 |
| chr1:113196220-113209767+ | hsa_circ_0005664 | CAPZA1 | 1.784 | 0.000206 |
| chr9:135171259-135187243- | hsa_circ_0138192 | SETX | 1.786 | 0.006889 |
| chr10:98644160-98667504+ | hsa_circ_0094657 | LCOR | 1.787 | 0.035219 |
| chr18:29432409-29432626- | hsa_circ_0000841 | TRAPPC8 | 1.788 | 0.005209 |
| chr7:105121499-105146720- | hsa_circ_0132912 | PUS7 | 1.789 | 0.009732 |
| chr3:121573534-121591635+ | | EAF2 | 1.791 | 0.003022 |
| chr13:42439872-42442613- | hsa_circ_0000478 | VWA8 | 1.791 | 9.99E-06 |
| chr3:160131261-160149613+ | hsa_circ_0122582 | SMC4 | 1.792 | 0.000672 |
| chr13:36822743-36828280- | hsa_circ_0029998 | CCDC169 | 1.792 | 0.000288 |
| chr15:50330965-50399205- | hsa_circ_0035199 | ATP8B4 | 1.792 | 7.33E-06 |
| chr22:41521868-41527637+ | hsa_circ_0116610 | EP300 | 1.797 | 0.00517 |
| chr5:619105-620376+ | hsa_circ_0071653 | CEP72 | 1.798 | 0.00145 |
| chr11:16339992-16362798- | hsa_circ_0095454 | SOX6 | 1.799 | 0.00517 |
| chr14:60749402-60750255+ | hsa_circ_0032108 | PPM1A | 1.799 | 0.00517 |
| chr16:30492760-30495584+ | | ITGAL | 1.801 | 0.008992 |
| chr14:31626046-31641328- | hsa_circ_0101616 | HECTD1 | 1.804 | 0.009732 |
| chrX:24080533-24086225+ | | EIF2S3 | 1.807 | 0.018448 |
| chr12:11273609-11285966- | hsa_circ_0006488 | PRB4 | 1.807 | 0.000965 |
| chr2:61597456-61622368- | hsa_circ_0005738 | USP34 | 1.807 | 0.001756 |
| chr16:14693761-14721193- | hsa_circ_0037973 | PARN | 1.808 | 0.003022 |
| chr1:243449574-243507633+ | | SDCCAG8 | 1.809 | 0.005209 |
| chr20:33954360-33971936- | hsa_circ_0004994 | UQCC1 | 1.813 | 0.005209 |
| chr11:66947550-66949363+ | hsa_circ_0096253 | KDM2A | 1.814 | 0.004215 |
| chr18:19345733-19353689+ | hsa_circ_0006918 | MIB1 | 1.814 | 0.000807 |
| chr5:67522118-67522837+ | hsa_circ_0006411 | PIK3R1 | 1.815 | 0.000242 |
| chr18:9117836-9126905+ | hsa_circ_0004428 | NDUFV2 | 1.819 | 0.009732 |
| chr3:196449286-196457963+ | hsa_circ_0123222 | PIGX | 1.822 | 0.00517 |
| chr10:93579027-93602148+ | hsa_circ_0094400 | TNKS2 | 1.822 | 0.005209 |
| chr6:18249882-18258636- | hsa_circ_0131432 | DEK | 1.822 | 0.000343 |
| chr2:227729320-227788879+ | hsa_circ_0119258 | RHBDD1 | 1.825 | 0.005209 |
| chr6:144808684-144858853+ | hsa_circ_0130903 | UTRN | 1.825 | 0.00517 |
| chr2:191765290-191769893+ | hsa_circ_0006662 | GLS | 1.826 | 0.001788 |
| chr6:90045021-90053475- | hsa_circ_0077304 | UBE2J1 | 1.827 | 0.01937 |
| chr2:106761646-106782539- | hsa_circ_0004029 | UXS1 | 1.83 | 0.005209 |
| chr9:325671-340321+ | hsa_circ_0086190 | DOCK8 | 1.831 | 0.008992 |
| chr9:79996892-80022523+ | hsa_circ_0008075 | VPS13A | 1.831 | 8.06E-05 |
| chr7:73649862-73661093- | hsa_circ_0134619 | RFC2 | 1.832 | 0.001489 |
| chr3:135979321-135980907+ | hsa_circ_0001341 | PCCB | 1.834 | 3.44E-05 |
| chr7:8095063-8110761+ | hsa_circ_0134785 | GLCCI1 | 1.834 | 0.000242 |
| chr1:155490891-155491409- | hsa_circ_0014592 | ASH1L | 1.834 | 0.000965 |
| chr8:41905896-41907225- | hsa_circ_0002754 | KAT6A | 1.84 | 0.001597 |
| chr10:74468041-74475660+ | hsa_circ_0000246 | MCU | 1.841 | 0.008084 |
| chr4:68442921-68449420+ | | STAP1 | 1.841 | 0.000807 |
| chr12:104856918-104857102+ | | CHST11 | 1.841 | 0.035219 |
| chr17:80417868-80430574+ | hsa_circ_0046419 | NARF | 1.842 | 0.026813 |
| chr15:43748090-43762264- | hsa_circ_0103594 | TP53BP1 | 1.842 | 0.011249 |
| chr1:154145384-154145677- | hsa_circ_0008368 | TPM3 | 1.845 | 0.009732 |
| chr5:64747302-64769779- | hsa_circ_0072688 | ADAMTS6 | 1.846 | 0.001012 |
| chr9:115166275-115181238+ | hsa_circ_0003597 | HSDL2 | 1.846 | 2.22E-05 |
| chr17:27778473-27778698+ | hsa_circ_0002839 | TAOK1 | 1.847 | 0.001489 |
| chr8:54682178-54684727- | hsa_circ_0136755 | ATP6V1H | 1.848 | 0.001489 |
| chr3:47468647-47470160- | hsa_circ_0065217 | SCAP | 1.848 | 0.003022 |
| chr1:172525009-172526934+ | hsa_circ_0015262 | SUCO | 1.849 | 3.03E-05 |
| chr6:166829530-166829724- | | RPS6KA2 | 1.849 | 0.00145 |
| chr3:152132730-152150709+ | hsa_circ_0001349 | MBNL1 | 1.85 | 1.62E-06 |
| chr4:85686963-85724620- | | WDFY3 | 1.85 | 0.000807 |
| chr17:62476406-62481985- | hsa_circ_0107426 | POLG2 | 1.851 | 0.00517 |
| chr10:103557737-103567658- | hsa_circ_0019608 | MGEA5 | 1.852 | 0.001577 |
| chr8:125332327-125339662- | hsa_circ_0085495 | TMEM65 | 1.853 | 0.018448 |
| chr2:173435454-173460751+ | hsa_circ_0006006 | PDK1 | 1.856 | 0.003022 |
| chr6:108202353-108204350- | hsa_circ_0077548 | SEC63 | 1.857 | 0.001295 |
| chr22:46493806-46505788+ | hsa_circ_0008668 | MIRLET7BHG | 1.858 | 0.003039 |
| chr17:16004564-16005071- | hsa_circ_0106250 | NCOR1 | 1.865 | 0.003657 |
| chrM:14254-14434+ | | JA760602 | 1.868 | 0.003022 |
| chr4:103571694-103586009- | hsa_circ_0124887 | MANBA | 1.868 | 0.00044 |
| chr15:40920268-40943017+ | hsa_circ_0103435 | CASC5 | 1.87 | 0.000288 |
| chr1:146727468-146731572+ | hsa_circ_0013947 | CHD1L | 1.871 | 0.035219 |
| chr14:71818568-71884913+ | | SIPA1L1 | 1.872 | 0.001489 |
| chr5:40728447-40747121- | hsa_circ_0007795 | TTC33 | 1.874 | 0.003022 |
| chr15:59179174-59179739- | hsa_circ_0000604 | SLTM | 1.876 | 0.000212 |
| chr14:61262935-61285565+ | hsa_circ_0032116 | MNAT1 | 1.877 | 8.10E-05 |
| chrX:46712911-46713576+ | | RP2 | 1.877 | 0.018448 |
| chr19:5653126-5653431+ | hsa_circ_0109928 | SAFB | 1.877 | 0.005209 |
| chr22:46493806-46494438+ | hsa_circ_0063776 | MIRLET7BHG | 1.878 | 0.003022 |
| chr13:100191700-100196249+ | hsa_circ_0099708 | TM9SF2 | 1.881 | 0.001023 |
| chr4:83799883-83802075- | hsa_circ_0070253 | SEC31A | 1.881 | 0.000591 |
| chr15:56434976-56436681- | hsa_circ_0103941 | RFX7 | 1.884 | 0.001028 |
| chr13:41826781-41828756- | hsa_circ_0100454 | MTRF1 | 1.887 | 0.000137 |
| chr2:24362240-24369956+ | hsa_circ_0008073 | FAM228B | 1.887 | 0.000591 |
| chr10:17401513-17432619- | hsa_circ_0093240 | ST8SIA6 | 1.892 | 0.001577 |
| chr17:62501778-62501934- | | DDX5 | 1.894 | 0.001577 |
| chr5:107630553-107684231- | hsa_circ_0127480 | FBXL17 | 1.896 | 0.005209 |
| chr4:2701407-2702271+ | hsa_circ_0003758 | FAM193A | 1.896 | 0.000274 |
| chr11:581492-592674+ | | PHRF1 | 1.898 | 0.006889 |
| chr8:108306164-108334356- | | ANGPT1 | 1.9 | 0.001023 |
| chr5:171516809-171516996- | | STK10 | 1.903 | 0.018448 |
| chr10:69748420-69804320- | hsa_circ_0009151 | HERC4 | 1.903 | 0.005209 |
| chr12:53410257-53412790+ | hsa_circ_0026496 | EIF4B | 1.904 | 0.003022 |
| chr11:118451961-118464413+ | hsa_circ_0095124 | ARCN1 | 1.906 | 0.003022 |
| chr1:52943380-52947595- | hsa_circ_0012545 | ZCCHC11 | 1.909 | 0.00517 |
| chr6:6248545-6251162- | | F13A1 | 1.909 | 0.001023 |
| chr2:122514816-122519100+ | hsa_circ_0002847 | TSN | 1.909 | 1.74E-06 |
| chr17:953290-995090- | | ABR | 1.909 | 0.001023 |
| chr7:91851216-91855996- | hsa_circ_0135053 | KRIT1 | 1.909 | 0.002766 |
| chrM:3568-3790+ | | TVAS5 | 1.91 | 0.000135 |
| chr5:179136874-179147561+ | hsa_circ_0002051 | CANX | 1.911 | 1.73E-05 |
| chr7:102038067-102047945+ | hsa_circ_0132821 | PRKRIP1 | 1.914 | 6.86E-05 |
| chr11:17153463-17167489- | hsa_circ_0021396 | PIK3C2A | 1.919 | 2.01E-05 |
| chr4:128904094-128942393+ | hsa_circ_0125279 | C4orf29 | 1.921 | 0.00517 |
| chr16:28607104-28619924- | | NPIPL1 | 1.922 | 0.00517 |
| chr11:66974981-66986874+ | hsa_circ_0096256 | KDM2A | 1.922 | 0.003022 |
| chrX:117530933-117538407+ | hsa_circ_0001939 | WDR44 | 1.924 | 0.009732 |
| chr17:3608392-3608898- | hsa_circ_0106743 | GSE61474_XLOC_026870 | 1.924 | 0.009732 |
| chr10:75830428-75834661+ | hsa_circ_0018881 | VCL | 1.924 | 0.000123 |
| chr14:76248830-76249873+ | hsa_circ_0032725 | TTLL5 | 1.926 | 0.001023 |
| chr2:86348602-86355114+ | hsa_circ_0007191 | PTCD3 | 1.926 | 0.00517 |
| chr4:4458918-4473433- | | STX18 | 1.927 | 0.00517 |
| chr22:23613719-23615961+ | hsa_circ_0116261 | BCR | 1.929 | 0.000807 |
| chr12:133083838-133084936+ | hsa_circ_0002826 | FBRSL1 | 1.931 | 0.009732 |
| chrX:109263580-109263891+ | | TMEM164 | 1.931 | 0.018448 |
| chr16:58608513-58609033- | hsa_circ_0006982 | CNOT1 | 1.933 | 0.00044 |
| chr7:72159677-72178756- | hsa_circ_0007603 | TYW1B | 1.935 | 0.00035 |
| chr6:109962721-109965891- | hsa_circ_0130282 | AK9 | 1.936 | 0.00044 |
| chr19:17273160-17273932+ | hsa_circ_0000909 | MYO9B | 1.937 | 8.10E-05 |
| chr12:102150990-102164935- | hsa_circ_0097038 | GNPTAB | 1.938 | 0.000121 |
| chr16:2369582-2369841- | hsa_circ_0037516 | ABCA3 | 1.94 | 0.000242 |
| chr19:39408364-39409453- | hsa_circ_0050954 | SARS2 | 1.95 | 0.000133 |
| chr13:28840855-28845003+ | hsa_circ_0100183 | PAN3 | 1.957 | 0.001489 |
| chr2:48725632-48738607+ | hsa_circ_0006911 | PPP1R21 | 1.96 | 5.32E-05 |
| chr7:77026299-77035422- | | GSAP | 1.961 | 0.000288 |
| chr18:44617545-44623775- | hsa_circ_0108520 | RP11-49K24.4 | 1.962 | 0.000805 |
| chr4:148743878-148744108+ | hsa_circ_0071099 | ARHGAP10 | 1.969 | 0.003022 |
| chr2:113157209-113157771- | hsa_circ_0002706 | RGPD8 | 1.973 | 2.30E-05 |
| chr2:9048751-9098771- | hsa_circ_0000972 | MBOAT2 | 1.973 | 4.15E-06 |
| chr10:105106995-105108756- | hsa_circ_0005052 | PCGF6 | 1.975 | 0.001023 |
| chr12:42711142-42717906- | hsa_circ_0098556 | ZCRB1 | 1.976 | 0.000523 |
| chr9:132740744-132761338- | hsa_circ_0138148 | FNBP1 | 1.976 | 0.001756 |
| chr5:154244752-154252211+ | hsa_circ_0074719 | CNOT8 | 1.976 | 0.002578 |
| chrY:15021271-15024974+ | hsa_circ_0008297 | DDX3Y | 1.98 | 3.51E-05 |
| chr18:60015401-60017170+ | hsa_circ_0047880 | TNFRSF11A | 1.983 | 0.005209 |
| chr8:100515064-100523740+ | hsa_circ_0135264 | VPS13B | 1.984 | 1.73E-05 |
| chr14:45618040-45628483+ | hsa_circ_0101878 | FANCM | 1.985 | 0.000597 |
| chr15:85180578-85181708+ | hsa_circ_0036592 | SCAND2P | 1.985 | 0.002766 |
| chr9:88430794-88444521+ | | LOC389765 | 1.99 | 0.003022 |
| chr17:40497577-40500535- | hsa_circ_0043815 | STAT3 | 1.99 | 0.000597 |
| chr18:13012972-13019205+ | hsa_circ_0107929 | CEP192 | 1.992 | 8.47E-05 |
| chr5:1045029-1045441- | | XLOC_004700 | 1.993 | 0.00517 |
| chr10:1130343-1151207+ | hsa_circ_0007796 | WDR37 | 1.993 | 0.002766 |
| chr22:28306952-28310335- | hsa_circ_0003056 | PITPNB | 1.995 | 0.004215 |
| chr18:19119881-19146167- | hsa_circ_0108033 | ESCO1 | 1.995 | 0.002766 |
| chr9:102722199-102722437+ | hsa_circ_0001876 | STX17 | 1.997 | 0.000223 |
| chr7:158662546-158669382+ | hsa_circ_0001778 | WDR60 | 1.997 | 2.08E-05 |
| chr7:74239462-74248066- | hsa_circ_0005811 | GTF2IRD2 | 2 | 0.001089 |
| chr15:50929619-50955243- | hsa_circ_0103785 | TRPM7 | 2.001 | 0.000206 |
| chr12:26748398-26808785- | | ITPR2 | 2.002 | 0.001489 |
| chr21:45165960-45168961+ | hsa_circ_0008021 | PDXK | 2.002 | 4.15E-06 |
| chr1:113119608-113153625- | hsa_circ_0110466 | ST7L | 2.003 | 7.41E-05 |
| chr6:130374004-130442103+ | | L3MBTL3 | 2.007 | 0.00517 |
| chr2:61710092-61717911- | hsa_circ_0002607 | XPO1 | 2.007 | 4.36E-07 |
| chr2:242694460-242695429+ | hsa_circ_0059132 | D2HGDH | 2.01 | 7.16E-05 |
| chr14:91759396-91759683- | | CCDC88C | 2.014 | 0.00517 |
| chr8:90982592-90990551- | hsa_circ_0084868 | NBN | 2.016 | 0.000137 |
| chr14:70125230-70125430+ | hsa_circ_0032352 | SUSD6 | 2.02 | 0.002766 |
| chr7:92146590-92148392- | hsa_circ_0081028 | PEX1 | 2.021 | 0.002766 |
| chr8:142154247-142178624+ | | DENND3 | 2.022 | 0.00517 |
| chr3:130881252-130889731+ | hsa_circ_0067347 | NEK11 | 2.024 | 0.012578 |
| chr12:125396825-125397736- | | UBC | 2.024 | 5.50E-07 |
| chr6:155108995-155126620+ | hsa_circ_0078374 | SCAF8 | 2.026 | 0.001023 |
| chr5:179996110-179998432+ | hsa_circ_0075366 | CNOT6 | 2.026 | 0.000242 |
| chr6:170162525-170168267+ | hsa_circ_0004237 | ERMARD | 2.026 | 8.69E-06 |
| chr17:19823349-19839743- | hsa_circ_0106369 | AKAP10 | 2.027 | 0.000807 |
| chrX:147732849-147744289+ | hsa_circ_0139872 | AFF2 | 2.028 | 0.000223 |
| chr6:157731958-157735235- | | TMEM242 | 2.028 | 0.001023 |
| chr17:46189393-46190763+ | hsa_circ_0002069 | SNX11 | 2.029 | 1.66E-08 |
| chr9:5825092-5831028- | hsa_circ_0086293 | ERMP1 | 2.035 | 0.000807 |
| chr9:125895124-125946577- | hsa_circ_0137890 | STRBP | 2.038 | 0.000123 |
| chr3:11850991-11871338- | hsa_circ_0002074 | TAMM41 | 2.039 | 0.000597 |
| chrM:13847-14067- | | JA760602 | 2.041 | 0.001597 |
| chr15:52561950-52575089- | hsa_circ_0103875 | MYO5C | 2.041 | 0.00035 |
| chr14:102661275-102664184+ | hsa_circ_0003512 | WDR20 | 2.046 | 3.24E-05 |
| chr3:155637022-155643155+ | hsa_circ_0003017 | GMPS | 2.048 | 0.000288 |
| chr13:49712883-49741439+ | hsa_circ_0100653 | FNDC3A | 2.05 | 0.00044 |
| chr9:114873934-114919893- | hsa_circ_0088054 | SUSD1 | 2.052 | 0.001023 |
| chr14:73459851-73460065- | hsa_circ_0032491 | ZFYVE1 | 2.052 | 0.001023 |
| chr7:6178723-6183827+ | hsa_circ_0079333 | USP42 | 2.053 | 1.92E-06 |
| chr8:108334124-108359325- | | ANGPT1 | 2.057 | 0.000206 |
| chr2:171884849-171917666- | | TLK1 | 2.062 | 0.009732 |
| chr1:9770163-9770654+ | hsa_circ_0009654 | PIK3CD | 2.064 | 0.000523 |
| chr17:76778284-76834832- | | CYTH1 | 2.064 | 0.000807 |
| chr1:6880241-6948959+ | hsa_circ_0002975 | CAMTA1 | 2.067 | 0.001489 |
| chr2:202587766-202589182- | hsa_circ_0004852 | ALS2 | 2.068 | 0.00044 |
| chr7:74541717-74558472+ | | GTF2IRD2 | 2.068 | 7.41E-05 |
| chr5:126140468-126161799+ | hsa_circ_0073747 | LMNB1 | 2.069 | 0.009732 |
| chr7:8043538-8099878+ | hsa_circ_0001678 | GLCCI1 | 2.069 | 0.001577 |
| chr10:27403450-27410377- | hsa_circ_0093432 | YME1L1 | 2.07 | 0.000123 |
| chrX:1592757-1593006- | | CRLF2 | 2.072 | 4.38E-06 |
| chr13:50042000-50057699+ | hsa_circ_0100673 | SETDB2 | 2.073 | 0.001489 |
| chr12:116534474-116549317- | hsa_circ_0000441 | MED13L | 2.075 | 2.86E-10 |
| chr5:132219032-132220811- | hsa_circ_0007945 | AFF4 | 2.076 | 0.005209 |
| chr4:77065302-77065626- | hsa_circ_0070040 | NUP54 | 2.076 | 0.003039 |
| chr2:215617171-215646233- | hsa_circ_0119090 | BARD1 | 2.079 | 0.000223 |
| chr22:32007129-32007826+ | hsa_circ_0062923 | SFI1 | 2.081 | 0.00044 |
| chr7:148851037-148851432+ | hsa_circ_0004351 | ZNF398 | 2.083 | 0.002578 |
| chr5:108281831-108295048+ | hsa_circ_0003032 | FER | 2.085 | 0.00035 |
| chr9:132719639-132757237- | hsa_circ_0089076 | FNBP1 | 2.087 | 0.001489 |
| chr15:64066893-64067848- | hsa_circ_0007112 | HERC1 | 2.096 | 0.000137 |
| chr16:19639959-19659204+ | hsa_circ_0105141 | C16orf62 | 2.097 | 0.000597 |
| chr5:37179463-37181107- | hsa_circ_0128966 | C5orf42 | 2.099 | 0.001756 |
| chr20:40161689-40179999- | hsa_circ_0001159 | CHD6 | 2.099 | 6.60E-06 |
| chr8:67513933-67514758- | hsa_circ_0004863 | MYBL1 | 2.106 | 2.08E-05 |
| chrX:76888695-76912143- | hsa_circ_0140608 | ATRX | 2.108 | 0.009732 |
| chr14:73614503-73640415+ | hsa_circ_0002564 | PSEN1 | 2.109 | 1.90E-08 |
| chr14:31424826-31425448- | hsa_circ_0007656 | STRN3 | 2.11 | 0.003657 |
| chr4:148775827-148803083+ | hsa_circ_0125473 | ARHGAP10 | 2.112 | 0.001756 |
| chr15:40475915-40477843+ | hsa_circ_0008471 | BUB1B | 2.113 | 0.00517 |
| chr14:104245082-104263855- | hsa_circ_0005791 | PPP1R13B | 2.113 | 0.002766 |
| chr3:47079156-47098980- | hsa_circ_0006544 | SETD2 | 2.113 | 0.000807 |
| chr12:28603094-28637098+ | hsa_circ_0098291 | CCDC91 | 2.115 | 0.001577 |
| chr18:43698147-43700026+ | hsa_circ_0047581 | HAUS1 | 2.118 | 0.004215 |
| chr15:43067333-43132631- | hsa_circ_0034863 | TTBK2 | 2.119 | 2.01E-05 |
| chr20:31979948-31981872- | hsa_circ_0059855 | CDK5RAP1 | 2.124 | 0.00517 |
| chr16:53301206-53302038+ | hsa_circ_0008031 | CHD9 | 2.129 | 0.00035 |
| chr13:95768176-95840796- | hsa_circ_0030582 | ABCC4 | 2.135 | 9.05E-06 |
| chr14:75136352-75138186- | hsa_circ_0005030 | AREL1 | 2.137 | 3.24E-05 |
| chr1:224605962-224612356- | hsa_circ_0002322 | WDR26 | 2.139 | 0.00035 |
| chr7:148716084-148718239- | hsa_circ_0001766 | PDIA4 | 2.14 | 0.000212 |
| chr17:20149239-20163607+ | hsa_circ_0003545 | SPECC1 | 2.141 | 0.004236 |
| chr3:37170554-37196147- | | LRRFIP2 | 2.145 | 0.000807 |
| chr6:10935291-10956475+ | hsa_circ_0075625 | SYCP2L | 2.152 | 0.000807 |
| chr17:36517594-36522300+ | hsa_circ_0043328 | SOCS7 | 2.154 | 0.000133 |
| chrX:84322133-84329397+ | hsa_circ_0140676 | APOOL | 2.157 | 5.23E-05 |
| chr12:49890616-49893988+ | hsa_circ_0026154 | SPATS2 | 2.158 | 0.00035 |
| chr13:103279362-103309557+ | hsa_circ_0099783 | TPP2 | 2.159 | 0.000807 |
| chr14:91155884-91196540- | | TTC7B | 2.16 | 0.000807 |
| chr10:98667022-98667504+ | hsa_circ_0007707 | LCOR | 2.161 | 0.001489 |
| chr1:245165423-245185598+ | hsa_circ_0006983 | EFCAB2 | 2.162 | 5.49E-05 |
| chr6:84894905-84913814- | hsa_circ_0132476 | CEP162 | 2.169 | 3.87E-06 |
| chr6:144772506-144780490+ | hsa_circ_0009096 | UTRN | 2.169 | 0.000206 |
| chr17:56404949-56406441- | | BZRAP1 | 2.169 | 0.002039 |
| chr3:183432932-183446639+ | hsa_circ_0068173 | YEATS2 | 2.17 | 0.001756 |
| chr4:154547299-154553990+ | hsa_circ_0071311 | KIAA0922 | 2.17 | 2.16E-06 |
| chr8:17867056-17872349+ | hsa_circ_0136069 | PCM1 | 2.171 | 0.000597 |
| chr21:45499445-45500008+ | hsa_circ_0003476 | TRAPPC10 | 2.171 | 0.000137 |
| chr19:48229069-48229481+ | hsa_circ_0003146 | EHD2 | 2.171 | 0.00044 |
| chr2:38812786-38818790- | hsa_circ_0054144 | HNRNPLL | 2.173 | 0.001023 |
| chr17:40650942-40653322+ | hsa_circ_0043837 | ATP6V0A1 | 2.173 | 2.01E-05 |
| chr3:44986660-45000952- | hsa_circ_0065052 | ZDHHC3 | 2.178 | 7.16E-05 |
| chr2:198388348-198405161+ | hsa_circ_0057681 | MOB4 | 2.185 | 0.000242 |
| chr1:155313105-155316260- | hsa_circ_0014551 | ASH1L | 2.185 | 0.001295 |
| chr5:137904614-137906830- | hsa_circ_0003280 | HSPA9 | 2.186 | 0.00043 |
| chr12:110729825-110734542+ | hsa_circ_0097268 | ATP2A2 | 2.187 | 4.25E-05 |
| chr8:124346118-124350061- | hsa_circ_0085459 | ATAD2 | 2.189 | 0.000121 |
| chr16:19627436-19637545+ | hsa_circ_0004683 | C16orf62 | 2.196 | 5.32E-05 |
| chr3:180665653-180667131+ | hsa_circ_0002004 | FXR1 | 2.198 | 1.92E-06 |
| chr1:78107069-78107340- | hsa_circ_0002717 | ZZZ3 | 2.199 | 0.000133 |
| chr20:61541051-61545758- | | DIDO1 | 2.2 | 0.00044 |
| chr17:953290-1028702- | hsa_circ_0107839 | ABR | 2.201 | 0.000121 |
| chr14:39620950-39628754- | hsa_circ_0031738 | TRAPPC6B | 2.203 | 0.000188 |
| chr14:31562113-31562350+ | hsa_circ_0101581 | AP4S1 | 2.205 | 0.000133 |
| chr17:45479498-45517868+ | | EFCAB13 | 2.205 | 0.002766 |
| chr15:65771218-65772737- | hsa_circ_0035901 | DPP8 | 2.208 | 0.000133 |
| chr17:59853762-59878835- | hsa_circ_0107328 | BRIP1 | 2.208 | 0.000133 |
| chr15:85230856-85234875- | hsa_circ_0036599 | SEC11A | 2.21 | 0.000223 |
| chrM:12376-12564+ | | MT-ND5 | 2.212 | 0.000133 |
| chr8:68015272-68102994+ | | CSPP1 | 2.217 | 0.000133 |
| chr12:50847243-50848200+ | hsa_circ_0026238 | LARP4 | 2.218 | 0.002766 |
| chr10:15858834-15879317- | hsa_circ_0017850 | FAM188A | 2.221 | 0.000332 |
| chr2:227729320-227773594+ | hsa_circ_0058494 | RHBDD1 | 2.224 | 7.41E-05 |
| chr9:17330630-17342442+ | hsa_circ_0086422 | CNTLN | 2.224 | 2.25E-05 |
| chr14:45711237-45716580- | hsa_circ_0101892 | MIS18BP1 | 2.224 | 0.00044 |
| chr3:155628481-155643155+ | hsa_circ_0008184 | GMPS | 2.225 | 2.95E-07 |
| chr4:156696120-156698794+ | hsa_circ_0125610 | GUCY1B3 | 2.232 | 0.002766 |
| chr17:169211-171206- | hsa_circ_0041152 | RPH3AL | 2.233 | 0.000242 |
| chr3:141228410-141259451+ | hsa_circ_0006347 | RASA2 | 2.236 | 4.14E-05 |
| chr2:89100616-89104394+ | hsa_circ_0121140 | ANKRD36BP2 | 2.24 | 0.000805 |
| chr13:25881944-25889605+ | hsa_circ_0100106 | NUPL1 | 2.241 | 5.49E-05 |
| chr1:76200476-76205795+ | hsa_circ_0114117 | ACADM | 2.241 | 0.000242 |
| chr9:36351112-36357939- | | RNF38 | 2.242 | 0.000223 |
| chr10:70513609-70517134+ | hsa_circ_0018556 | CCAR1 | 2.243 | 0.001489 |
| chr16:14738131-14738466+ | hsa_circ_0009065 | BFAR | 2.243 | 6.00E-06 |
| chr8:67755687-67763155+ | hsa_circ_0136933 | SGK3 | 2.245 | 0.000129 |
| chr17:73038276-73038754- | hsa_circ_0006942 | ATP5H | 2.249 | 5.23E-05 |
| chr12:113705648-113707650+ | hsa_circ_0002857 | TPCN1 | 2.25 | 0.000133 |
| chr9:86293356-86297981- | hsa_circ_0001866 | UBQLN1 | 2.25 | 0.001295 |
| chr14:51202234-51208446- | | NIN | 2.266 | 4.14E-05 |
| chr2:136432902-136437894+ | hsa_circ_0001070 | R3HDM1 | 2.269 | 4.25E-05 |
| chr8:98725890-98731417+ | hsa_circ_0003117 | MTDH | 2.269 | 0.000242 |
| chr4:103501692-103504114+ | hsa_circ_0124885 | NFKB1 | 2.272 | 4.14E-05 |
| chr4:103610731-103612114- | hsa_circ_0001429 | MANBA | 2.276 | 0.012842 |
| chr15:76577978-76585041- | hsa_circ_0008094 | ETFA | 2.278 | 1.51E-05 |
| chrX:13767546-13773361+ | hsa_circ_0089903 | OFD1 | 2.279 | 7.41E-05 |
| chr11:36654834-36669705+ | hsa_circ_0021762 | C11orf74 | 2.279 | 0.00044 |
| chr21:37619815-37623582+ | hsa_circ_0007193 | DOPEY2 | 2.28 | 7.82E-06 |
| chr17:25630393-25638642+ | hsa_circ_0005336 | WSB1 | 2.28 | 3.87E-06 |
| chr10:104809464-104816721+ | hsa_circ_0092503 | CNNM2 | 2.287 | 7.41E-05 |
| chr20:3277508-3278822- | hsa_circ_0059286 | C20orf194 | 2.287 | 4.25E-05 |
| chr16:67644727-67655494+ | hsa_circ_0105757 | CTCF | 2.293 | 0.002766 |
| chr18:72342923-72353078+ | hsa_circ_0047958 | ZNF407 | 2.296 | 0.000206 |
| chr11:77812139-77820627- | hsa_circ_0096515 | RNU6-83P | 2.297 | 1.92E-06 |
| chr10:32309950-32310215- | hsa_circ_0093522 | KIF5B | 2.298 | 5.23E-05 |
| chr12:50821545-50824353+ | hsa_circ_0003664 | LARP4 | 2.298 | 2.95E-07 |
| chr19:53091785-53095481+ | hsa_circ_0109879 | ZNF137P | 2.299 | 3.24E-05 |
| chr16:67100585-67116211+ | | CBFB | 2.3 | 0.000242 |
| chr7:26233264-26235529- | hsa_circ_0134021 | HNRNPA2B1 | 2.303 | 4.14E-05 |
| chr19:34685383-34706566+ | hsa_circ_0008030 | LSM14A | 2.305 | 1.45E-05 |
| chr14:68228083-68229536- | hsa_circ_0000545 | ZFYVE26 | 2.31 | 1.62E-06 |
| chr8:42919244-42932507+ | hsa_circ_0008397 | FNTA | 2.314 | 2.53E-05 |
| chr15:59964836-59972507- | hsa_circ_0104035 | BNIP2 | 2.317 | 1.21E-06 |
| chr15:77046149-77067458- | hsa_circ_0104596 | SCAPER | 2.319 | 7.16E-05 |
| chr3:160131261-160141628+ | hsa_circ_0067841 | SMC4 | 2.325 | 2.53E-05 |
| chr6:139228632-139229953- | hsa_circ_0078017 | REPS1 | 2.327 | 0.000363 |
| chr18:9593766-9595100- | hsa_circ_0108984 | PPP4R1 | 2.331 | 0.001489 |
| chr1:211952260-211966532- | hsa_circ_0002274 | LPGAT1 | 2.332 | 1.25E-05 |
| chr10:77306789-77307420+ | hsa_circ_0094207 | C10orf11 | 2.334 | 0.000242 |
| chr15:51827860-51839605- | hsa_circ_0103835 | DMXL2 | 2.339 | 4.37E-06 |
| chr5:93964516-93979140+ | hsa_circ_0130036 | SLF1 | 2.341 | 0.000133 |
| chr3:57618992-57627474- | hsa_circ_0124346 | DENND6A | 2.343 | 4.25E-05 |
| chr4:151727423-151729550- | hsa_circ_0071196 | LRBA | 2.343 | 4.14E-05 |
| chr3:160131261-160137331+ | | SMC4 | 2.349 | 4.25E-05 |
| chrM:14146-14385- | | JA760602 | 2.349 | 0.000133 |
| chr3:56771213-56779490- | hsa_circ_0124321 | ARHGEF3 | 2.35 | 2.73E-05 |
| chr1:21220010-21231464- | hsa_circ_0007214 | EIF4G3 | 2.358 | 0.001489 |
| chr2:201796062-201822856- | hsa_circ_0057737 | ORC2 | 2.36 | 2.33E-05 |
| chr3:105389077-105400662- | hsa_circ_0066714 | CBLB | 2.36 | 1.93E-06 |
| chr12:112087743-112098479- | hsa_circ_0097340 | BRAP | 2.364 | 0.000223 |
| chr12:49445443-49445631- | | KMT2D | 2.364 | 0.000242 |
| chr1:231902886-231906816+ | | DISC1 | 2.365 | 7.41E-05 |
| chr21:38439562-38441924- | hsa_circ_0008160 | PIGP | 2.366 | 0.001489 |
| chr1:150795670-150799093- | hsa_circ_0003116 | ARNT | 2.367 | 3.24E-05 |
| chr16:58594116-58608664- | hsa_circ_0039676 | CNOT1 | 2.376 | 0.00044 |
| chr10:95140976-95148911- | hsa_circ_0005898 | MYOF | 2.378 | 0.000523 |
| chr5:112870002-112874878+ | hsa_circ_0127649 | YTHDC2 | 2.387 | 0.000121 |
| chr16:15162026-15166937- | hsa_circ_0038019 | RRN3 | 2.387 | 0.000242 |
| chr20:30676371-30681819+ | | HCK | 2.398 | 0.00035 |
| chr8:66657642-66695078- | hsa_circ_0084635 | PDE7A | 2.402 | 0.000137 |
| chr14:50262509-50281575- | hsa_circ_0101942 | NEMF | 2.403 | 2.53E-05 |
| chr3:48585965-48587667- | hsa_circ_0124008 | PFKFB4 | 2.406 | 1.51E-05 |
| chr20:19560644-19566188+ | hsa_circ_0114779 | SLC24A3 | 2.407 | 0.000242 |
| chr1:151060666-151079698+ | hsa_circ_0110790 | GABPB2 | 2.411 | 2.01E-05 |
| chr3:48572945-48573896- | hsa_circ_0003091 | PFKFB4 | 2.412 | 4.15E-06 |
| chr15:75702177-75706652- | | SIN3A | 2.413 | 0.000137 |
| chr14:103803014-103806140+ | hsa_circ_0033469 | EIF5 | 2.418 | 0.000121 |
| chr2:61721029-61724142- | hsa_circ_0006493 | XPO1 | 2.42 | 5.32E-05 |
| chr3:49323531-49323758- | hsa_circ_0124041 | USP4 | 2.421 | 7.48E-06 |
| chr10:7325866-7327916- | hsa_circ_0017643 | SFMBT2 | 2.421 | 1.62E-06 |
| chr8:25156460-25168048+ | hsa_circ_0005042 | DOCK5 | 2.425 | 7.41E-05 |
| chr2:29354124-29358532+ | hsa_circ_0006348 | CLIP4 | 2.434 | 7.41E-05 |
| chr7:102960056-102963241- | hsa_circ_0081750 | DNAJC2 | 2.437 | 1.06E-06 |
| chr17:57814814-57816308+ | hsa_circ_0044875 | VMP1 | 2.439 | 0.000135 |
| chr11:85737334-85742653- | hsa_circ_0002433 | PICALM | 2.441 | 2.01E-05 |
| chr16:14700341-14711507- | | PARN | 2.442 | 0.000212 |
| chr2:61722590-61726048- | hsa_circ_0054886 | XPO1 | 2.447 | 1.98E-07 |
| chrX:149983335-149984551- | hsa_circ_0004369 | CD99L2 | 2.456 | 0.000133 |
| chr22:41521868-41537226+ | hsa_circ_0116613 | EP300 | 2.464 | 0.000807 |
| chr18:46783380-46808545- | hsa_circ_0007006 | DYM | 2.469 | 1.99E-06 |
| chr15:102241289-102255166- | hsa_circ_0037108 | TARSL2 | 2.472 | 1.21E-06 |
| chr8:126159323-126207514+ | hsa_circ_0135711 | NSMCE2 | 2.478 | 3.24E-05 |
| chr7:148463653-148464841+ | hsa_circ_0006565 | CUL1 | 2.482 | 2.53E-05 |
| chr1:52870353-52883776+ | | PRPF38A | 2.483 | 7.41E-05 |
| chr10:50098624-50155014+ | | WDFY4 | 2.483 | 2.33E-05 |
| chr9:33311104-33319125+ | hsa_circ_0086645 | NFX1 | 2.483 | 1.99E-06 |
| chr5:54639159-54649097+ | hsa_circ_0129157 | SKIV2L2 | 2.493 | 0.000135 |
| chr9:20819795-20823114+ | hsa_circ_0003295 | FOCAD | 2.5 | 1.43E-08 |
| chr1:29028950-29030841+ | | GMEB1 | 2.501 | 7.48E-06 |
| chr2:61343114-61345251+ | hsa_circ_0007793 | KIAA1841 | 2.507 | 8.23E-07 |
| chr8:131226802-131249240- | hsa_circ_0085611 | ASAP1 | 2.508 | 1.06E-06 |
| chr17:42501717-42552271- | hsa_circ_0044079 | GPATCH8 | 2.509 | 5.45E-06 |
| chr12:25216654-25222365+ | hsa_circ_0025630 | LRMP | 2.511 | 9.30E-06 |
| chr7:5401528-5417655- | | TNRC18 | 2.519 | 1.32E-05 |
| chr1:1588706-1653150- | | CDK11B | 2.519 | 1.09E-07 |
| chr5:179135240-179137066+ | hsa_circ_0128724 | CANX | 2.52 | 4.53E-07 |
| chr11:68334482-68350597+ | hsa_circ_0023231 | PPP6R3 | 2.525 | 3.29E-06 |
| chr11:95546096-95546753+ | hsa_circ_0024067 | CEP57 | 2.526 | 2.94E-07 |
| chr17:30267305-30267505+ | hsa_circ_0042961 | SUZ12 | 2.528 | 4.61E-06 |
| chr19:9767223-9770143- | hsa_circ_0003516 | ZNF562 | 2.531 | 9.98E-08 |
| chr7:151049898-151053308+ | hsa_circ_0133690 | NUB1 | 2.532 | 4.14E-05 |
| chr3:101117705-101121891- | hsa_circ_0121353 | SENP7 | 2.533 | 4.27E-06 |
| chr20:43132456-43135637- | hsa_circ_0004860 | SERINC3 | 2.537 | 2.01E-05 |
| chr12:69107645-69108533+ | hsa_circ_0099092 | NUP107 | 2.542 | 1.06E-06 |
| chr3:52777368-52786352- | hsa_circ_0066113 | NEK4 | 2.544 | 5.45E-06 |
| chr14:35343698-35343868- | | BAZ1A | 2.545 | 7.41E-05 |
| chr6:109466422-109468140+ | hsa_circ_0004131 | CEP57L1 | 2.55 | 1.51E-05 |
| chr12:110566755-110581350+ | hsa_circ_0097245 | IFT81 | 2.552 | 5.10E-07 |
| chr12:70149164-70150443+ | hsa_circ_0099132 | RAB3IP | 2.553 | 0.000133 |
| chr19:5650989-5654467+ | hsa_circ_0109927 | SAFB | 2.553 | 7.40E-07 |
| chr6:132777100-132796801- | hsa_circ_0130729 | STX7 | 2.558 | 4.89E-06 |
| chr1:179989084-179991983+ | hsa_circ_0015482 | CEP350 | 2.569 | 2.33E-05 |
| chr4:83795764-83796975- | hsa_circ_0002671 | SEC31A | 2.57 | 0.000332 |
| chr4:128995615-129003460+ | hsa_circ_0007619 | LARP1B | 2.571 | 3.29E-06 |
| chr4:39739040-39776553+ | hsa_circ_0002590 | UBE2K | 2.573 | 1.15E-07 |
| chr4:148867768-148887990+ | hsa_circ_0006473 | ARHGAP10 | 2.587 | 1.93E-07 |
| chr16:30490412-30492905+ | hsa_circ_0039043 | ITGAL | 2.593 | 2.33E-05 |
| chr13:21987791-21999817- | hsa_circ_0006732 | ZDHHC20 | 2.594 | 2.40E-10 |
| chr13:52992127-53001354- | hsa_circ_0030342 | VPS36 | 2.596 | 5.45E-06 |
| chr10:17730026-17738868+ | hsa_circ_0000222 | STAM | 2.596 | 0.000123 |
| chr3:72842067-72893574- | hsa_circ_0124587 | SHQ1 | 2.599 | 3.29E-06 |
| chr3:47125210-47139571- | hsa_circ_0123904 | SETD2 | 2.603 | 4.27E-06 |
| chr6:42627394-42633983+ | hsa_circ_0131774 | UBR2 | 2.607 | 1.62E-06 |
| chr12:94613792-94621011+ | hsa_circ_0099519 | PLXNC1 | 2.615 | 1.09E-10 |
| chr10:74322653-74326552- | hsa_circ_0006628 | MICU1 | 2.616 | 1.98E-07 |
| chr3:137940768-137942575+ | hsa_circ_0007912 | ARMC8 | 2.618 | 1.27E-07 |
| chr20:32677322-32686439- | | EIF2S2 | 2.622 | 1.51E-05 |
| chr16:1364021-1370518+ | hsa_circ_0000665 | UBE2I | 2.631 | 7.41E-05 |
| chr1:155209407-155209868- | hsa_circ_0014507 | GBA | 2.64 | 1.32E-05 |
| chr6:83806644-83810605+ | hsa_circ_0132413 | DOPEY1 | 2.643 | 1.22E-06 |
| chr1:193065778-193070334- | hsa_circ_0007910 | GLRX2 | 2.649 | 3.29E-06 |
| chr9:33944363-33948585- | hsa_circ_0138658 | UBAP2 | 2.655 | 4.27E-06 |
| chr15:91346751-91347589+ | | BLM | 2.661 | 1.42E-06 |
| chr5:96349352-96364316+ | hsa_circ_0130091 | LNPEP | 2.666 | 1.99E-06 |
| chr4:166231705-166231902+ | hsa_circ_0125713 | KLHL2 | 2.666 | 9.81E-08 |
| chr6:80198801-80203467- | hsa_circ_0132366 | LCA5 | 2.668 | 0.000133 |
| chr17:80521230-80526077+ | hsa_circ_0000816 | FOXK2 | 2.671 | 2.03E-12 |
| chr14:103180760-103187714+ | hsa_circ_0006016 | RCOR1 | 2.673 | 1.22E-06 |
| chr3:27420740-27424741- | hsa_circ_0064614 | SLC4A7 | 2.675 | 0.000133 |
| chrM:6442-6603+ | | BC018860 | 2.68 | 5.49E-05 |
| chr4:129857810-129880932- | hsa_circ_0070959 | SCLT1 | 2.686 | 3.24E-05 |
| chr1:35881067-35881315+ | hsa_circ_0011558 | ZMYM4 | 2.691 | 1.98E-07 |
| chr19:2762504-2769089- | | SGTA | 2.709 | 1.32E-05 |
| chr2:173423436-173460751+ | | PDK1 | 2.719 | 8.23E-07 |
| chr7:73634074-73635064+ | hsa_circ_0080543 | LAT2 | 2.723 | 9.30E-06 |
| chr7:142105140-142162950+ | | TCRBV22S1A2N1T | 2.728 | 1.08E-08 |
| chr10:30625732-30630569- | hsa_circ_0018074 | MTPAP | 2.733 | 4.80E-07 |
| chr14:55647931-55650471- | hsa_circ_0000539 | DLGAP5 | 2.735 | 1.62E-08 |
| chr1:51869091-51874004- | hsa_circ_0012440 | EPS15 | 2.742 | 4.80E-07 |
| chr8:141889570-141900868- | hsa_circ_0006646 | PTK2 | 2.75 | 7.41E-05 |
| chr1:14057495-14068652+ | hsa_circ_0005986 | PRDM2 | 2.751 | 3.37E-08 |
| chr13:33306238-33320238+ | hsa_circ_0029961 | PDS5B | 2.757 | 4.53E-07 |
| chr13:28588589-28589838- | hsa_circ_0100160 | FLT3 | 2.764 | 2.94E-07 |
| chr15:55619729-55626205+ | hsa_circ_0103914 | PIGB | 2.766 | 4.27E-06 |
| chr16:29124371-29127647+ | | RRN3P2 | 2.766 | 4.27E-06 |
| chr1:155891166-155895634- | hsa_circ_0000140 | KIAA0907 | 2.769 | 4.80E-07 |
| chr3:169840379-169896726- | | PHC3 | 2.77 | 4.14E-05 |
| chr14:35565764-35577442- | hsa_circ_0101740 | PPP2R3C | 2.773 | 9.81E-08 |
| chr2:24181171-24199945+ | hsa_circ_0005970 | UBXN2A | 2.775 | 7.40E-07 |
| chr18:77455225-77457988+ | hsa_circ_0000860 | CTDP1 | 2.797 | 1.62E-08 |
| chr9:111812563-111835718- | hsa_circ_0004010 | TMEM245 | 2.802 | 4.12E-08 |
| chr5:137654908-137664218- | hsa_circ_0005537 | CDC25C | 2.808 | 3.29E-06 |
| chr4:79747191-79800045+ | hsa_circ_0127025 | BMP2K | 2.813 | 8.92E-10 |
| chr3:47139445-47144913- | hsa_circ_0001289 | SETD2 | 2.816 | 5.23E-08 |
| chr12:6839568-6839986+ | hsa_circ_0025244 | COPS7A | 2.823 | 2.46E-06 |
| chr7:1937836-1938026- | hsa_circ_0079135 | MAD1L1 | 2.828 | 1.51E-05 |
| chr17:38282436-38282659+ | hsa_circ_0008964 | MSL1 | 2.84 | 4.12E-08 |
| chr3:72890196-72891553- | hsa_circ_0005299 | SHQ1 | 2.855 | 8.23E-07 |
| chr12:121220458-121248689- | hsa_circ_0002179 | SPPL3 | 2.878 | 1.13E-06 |
| chr6:79770195-79770535- | hsa_circ_0003810 | PHIP | 2.883 | 3.09E-11 |
| chr14:68290259-68353921+ | hsa_circ_0102486 | RAD51B | 2.888 | 1.98E-07 |
| chr6:109977959-109996959- | hsa_circ_0130290 | AK9 | 2.89 | 1.66E-07 |
| chr10:93600318-93605697+ | hsa_circ_0094411 | TNKS2 | 2.899 | 9.81E-08 |
| chr2:136527339-136530117+ | | UBXN4 | 2.913 | 8.23E-07 |
| chr3:18419662-18438782- | | SATB1 | 2.929 | 1.42E-06 |
| chr4:166021780-166024248- | hsa_circ_0125711 | TMEM192 | 2.943 | 6.53E-10 |
| chr13:30826068-30829752- | hsa_circ_0100208 | KATNAL1 | 2.948 | 1.06E-07 |
| chrM:13979-14150+ | | MTND5 | 2.963 | 9.89E-08 |
| chr7:156549087-156619438- | hsa_circ_0003511 | LMBR1 | 2.971 | 5.83E-08 |
| chr14:39746138-39796226+ | hsa_circ_0031741 | CTAGE5 | 2.975 | 5.83E-08 |
| chr8:42716888-42725255- | hsa_circ_0084134 | RNF170 | 3.013 | 4.80E-07 |
| chr1:167353094-167358998+ | hsa_circ_0015099 | POU2F1 | 3.079 | 1.69E-09 |
| chr3:9711116-9712854+ | hsa_circ_0001265 | MTMR14 | 3.09 | 6.43E-09 |
| chrM:8985-9190+ | | OK/SW-cl.16 | 3.097 | 9.81E-08 |
| chr2:157406120-157414094+ | hsa_circ_0005732 | GPD2 | 3.119 | 1.02E-08 |
| chr8:30938383-30942762+ | | WRN | 3.145 | 9.81E-08 |
| chr6:86237980-86251761- | hsa_circ_0132510 | SNX14 | 3.151 | 2.57E-09 |
| chr8:37971710-37993284+ | hsa_circ_0008385 | ASH2L | 3.185 | 8.23E-07 |
| chr16:28163980-28167848- | hsa_circ_0007807 | XPO6 | 3.195 | 6.43E-09 |
| chr1:234553830-234608557- | hsa_circ_0112461 | TARBP1 | 3.209 | 2.57E-08 |
| chr20:43607084-43615937+ | hsa_circ_0060522 | STK4 | 3.249 | 1.73E-09 |
| chr6:7602856-7606428+ | hsa_circ_0001574 | SNRNP48 | 3.267 | 5.83E-08 |
| chrX:80064512-80064803- | hsa_circ_0091178 | BRWD3 | 3.277 | 1.73E-09 |
| chr6:2116005-2124965- | hsa_circ_0075451 | GMDS | 3.287 | 2.57E-08 |
| chr13:111857636-111870227+ | hsa_circ_0000503 | ARHGEF7 | 3.293 | 6.79E-10 |
| chr7:157013383-157024021+ | hsa_circ_0004755 | UBE3C | 3.316 | 6.79E-10 |
| chr1:213341201-213349835+ | hsa_circ_0005314 | RPS6KC1 | 3.349 | 8.58E-13 |
| chr9:33293678-33303266+ | | NFX1 | 3.381 | 2.83E-09 |
| chrM:8469-8927+ | | OK/SW-cl.16 | 3.384 | 1.66E-07 |
| chr8:67743489-67748309+ | hsa_circ_0136929 | SGK3 | 3.394 | 9.98E-11 |
| chr16:74949768-74950157- | hsa_circ_0004910 | WDR59 | 3.4 | 2.55E-10 |
| chr1:171537386-171540560+ | hsa_circ_0015232 | PRRC2C | 3.435 | 9.98E-11 |
| chr10:863665-866785- | | LARP4B | 3.436 | 1.02E-11 |
| chr18:8113484-8143777+ | hsa_circ_0002872 | PTPRM | 3.472 | 1.66E-07 |
| chr4:38987956-38995618- | hsa_circ_0069438 | TMEM156 | 3.524 | 1.07E-09 |
| chr4:17503342-17510986- | hsa_circ_0069249 | QDPR | 3.525 | 1.73E-09 |
| chr14:50131344-50131881- | | POLE2 | 3.563 | 6.59E-10 |
| chrM:14136-14377- | | JA760602 | 3.577 | 4.09E-10 |
| chr19:12989196-12989648- | hsa_circ_0109097 | DNASE2 | 3.611 | 6.32E-12 |
| chr20:35689506-35693876- | hsa_circ_0060237 | RBL1 | 3.649 | 4.09E-10 |
| chr6:14579105-14599919- | | RP11-330A16.1 | 3.655 | 1.43E-10 |
| chr16:75445723-75448593- | hsa_circ_0105973 | CFDP1 | 3.843 | 1.79E-14 |
| chr7:139083345-139090533+ | hsa_circ_0133532 | LUC7L2 | 4.3 | 1.76E-12 |
| chr10:5815805-5828013- | hsa_circ_0000210 | GDI2 | 4.984 | 9.63E-18 |

Table S5. CircRNAs related to outcome validated by Cox regression analysis

| CircRNA ID | circBase ID | Gene Name | Beta | P value |
| --- | --- | --- | --- | --- |
| chr10:863665-866785- | | LARP4B | 0.604185 | 0.000204 |
| chr9:86292642-86301070- | hsa_circ_0003715 | UBQLN1 | -0.20635 | 0.04078 |
| chr5:145197457-145205763- | hsa_circ_0006528 | PRELID2 | -0.25565 | 0.018925 |
| chr12:116534474-116549317- | hsa_circ_0000441 | MED13L | 0.179491 | 0.013832 |
| chrM:14136-14377- | | JA760602 | 0.484703 | 0.01367 |
| chr20:35689506-35693876- | hsa_circ_0060237 | RBL1 | 0.314612 | 0.035568 |
| chr2:231940225-231951895+ | hsa_circ_0001110 | PSMD1 | -0.25364 | 0.02148 |
| chr3:119219542-119232566+ | hsa_circ_0006884 | TIMMDC1 | -0.17444 | 0.046978 |
| chr16:28163980-28167848- | hsa_circ_0007807 | XPO6 | 0.408556 | 0.003061 |
| chr7:142105140-142162950+ | | TCRBV22S1A2N1T | 0.333792 | 0.002855 |
| chr6:144858718-144864006+ | hsa_circ_0130908 | UTRN | -0.20658 | 0.013307 |
| chr18:77455225-77457988+ | hsa_circ_0000860 | CTDP1 | 0.565055 | 0.000423 |
| chr15:75703833-75705386- | hsa_circ_0036353 | SIN3A | -0.17135 | 0.018746 |
| chr6:2116005-2124965- | hsa_circ_0075451 | GMDS | 0.307593 | 0.038205 |
| chr7:131060183-131084192+ | hsa_circ_0001746 | MKLN1 | -0.17972 | 0.018789 |
| chrM:8985-9190+ | | OK/SW-cl.16 | 0.904545 | 0.002628 |
| chr4:166231705-166231902+ | hsa_circ_0125713 | KLHL2 | 0.524346 | 0.037092 |
| chrM:13979-14150+ | | MTND5 | 0.252795 | 0.017788 |
| chr1:1588706-1653150- | | CDK11B | 0.203977 | 0.0353 |
| chr1:35879573-35881315+ | hsa_circ_0004709 | ZMYM4 | -0.1919 | 0.034893 |
| chr12:69983265-69987393+ | hsa_circ_0002940 | CCT2 | -0.16003 | 0.024087 |
| chr4:17816476-17816981+ | hsa_circ_0001395 | NCAPG | -0.19716 | 0.010763 |
| chr18:8113484-8143777+ | hsa_circ_0002872 | PTPRM | 0.856543 | 0.005549 |
| chrM:8469-8927+ | | OK/SW-cl.16 | 0.34228 | 0.049594 |
| chr4:57344545-57349436+ | hsa_circ_0126686 | SRP72 | -0.23182 | 0.043862 |
| chr13:33306238-33320238+ | hsa_circ_0029961 | PDS5B | 0.494989 | 0.00175 |
| chr12:125396825-125397736- | | UBC | 0.23118 | 0.03189 |
| chr16:31373157-31374074+ | hsa_circ_0039161 | ITGAX | -0.22893 | 0.028972 |
| chr12:129293333-129299615- | hsa_circ_0006689 | SLC15A4 | -0.24174 | 0.014887 |
| chr8:37971710-37993284+ | hsa_circ_0008385 | ASH2L | 0.68102 | 0.00291 |
| chr1:91403042-91406866- | hsa_circ_0114427 | ZNF644 | -0.26263 | 0.028576 |
| chr15:102241289-102255166- | hsa_circ_0037108 | TARSL2 | 0.462899 | 0.000464 |
| chr13:77798586-77818086- | hsa_circ_0100910 | MYCBP2 | -0.21843 | 0.038436 |
| chr15:50330965-50339661- | hsa_circ_0035197 | ATP8B4 | -0.18786 | 0.034989 |
| chr15:34544371-34547593- | hsa_circ_0103323 | SLC12A6 | -0.28755 | 0.049653 |
| chr9:14639894-14680160- | hsa_circ_0008952 | ZDHHC21 | -0.24164 | 0.005791 |
| chr9:33944363-33948585- | hsa_circ_0138658 | UBAP2 | 0.391635 | 0.011293 |
| chr13:28830429-28855516+ | hsa_circ_0029853 | PAN3 | -0.16962 | 0.048558 |
| chr1:29028950-29030841+ | | GMEB1 | 0.822044 | 0.009768 |
| chr4:48371866-48396670+ | hsa_circ_0126526 | SLAIN2 | -0.32873 | 0.012209 |
| chr5:137288317-137290065- | hsa_circ_0008177 | FAM13B | 0.218797 | 0.047035 |
| chr9:33953283-33956144- | hsa_circ_0003945 | UBAP2 | -0.19681 | 0.049796 |
| chr19:2762504-2769089- | | SGTA | 0.784182 | 0.003257 |
| chr7:5401528-5417655- | | TNRC18 | 0.549047 | 0.012327 |
| chr4:48686690-48712715- | hsa_circ_0002021 | FRYL | -0.19447 | 0.031863 |
| chr21:34804484-34805178+ | hsa_circ_0001185 | IFNGR2 | -0.14002 | 0.035988 |
| chr15:32815230-32825569- | | XLOC_l2_004867 | -0.23552 | 0.014911 |
| chr10:112640991-112650428+ | hsa_circ_0092663 | PDCD4 | -0.31834 | 0.018074 |
| chr2:230701564-230744844- | hsa_circ_0007381 | TRIP12 | -0.26612 | 0.021327 |
| chr6:76412361-76421132+ | hsa_circ_0077096 | SENP6 | -0.21163 | 0.030936 |
| chr2:61710092-61720188- | hsa_circ_0120656 | XPO1 | -0.20864 | 0.048179 |
| chr20:35695127-35696589- | hsa_circ_0060238 | RBL1 | -0.24774 | 0.016077 |
| chr7:26233264-26235529- | hsa_circ_0134021 | HNRNPA2B1 | 0.86483 | 0.004781 |
| chr3:44871463-44881948+ | hsa_circ_0002912 | KIF15 | -0.27283 | 0.031083 |
| chr1:1735858-1737977- | hsa_circ_0000007 | GNB1 | -0.22742 | 0.014282 |
| chr2:227729320-227732034+ | hsa_circ_0058493 | RHBDD1 | -0.14731 | 0.037921 |
| chr15:63988323-64008672- | hsa_circ_0035796 | HERC1 | -0.13955 | 0.040061 |
| chr1:114391162-114397671- | hsa_circ_0000111 | PTPN22 | -0.19699 | 0.016327 |
| chr1:114367764-114377061- | | PTPN22 | -0.20873 | 0.047573 |
| chr20:47691322-47707559+ | hsa_circ_0001168 | CSE1L | -0.21589 | 0.042377 |
| chr6:117010483-117026323+ | hsa_circ_0130438 | KPNA5 | -0.20039 | 0.010028 |
| chr1:98144651-98165103- | hsa_circ_0004161 | DPYD | -0.20632 | 0.039828 |
| chr1:40656444-40668286+ | hsa_circ_0007302 | RLF | -0.2443 | 0.029424 |
| chr12:32751431-32764217+ | hsa_circ_0025843 | FGD4 | -0.22164 | 0.006243 |
| chr16:1364021-1370518+ | hsa_circ_0000665 | UBE2I | 0.760581 | 0.000389 |
| chr8:141889570-141900868- | hsa_circ_0006646 | PTK2 | 0.815918 | 0.004375 |
| chr2:48701822-48718309+ | hsa_circ_0008358 | PPP1R21 | -0.27172 | 0.011082 |
| chr14:35343698-35343868- | | BAZ1A | 0.494228 | 0.016035 |
| chr15:72810408-72853890+ | hsa_circ_0000629 | ARIH1 | -0.35136 | 0.02656 |
| chr9:91943575-91943801+ | hsa_circ_0005432 | SECISBP2 | -0.25165 | 0.038891 |
| chrM:13847-13999+ | | MTND5 | 0.150708 | 0.029503 |
| chr18:51797730-51800460+ | hsa_circ_0007180 | POLI | -0.1959 | 0.04887 |
| chr2:203818728-203820481+ | hsa_circ_0003493 | CARF | -0.27465 | 0.011714 |
| chrX:149983335-149984551- | hsa_circ_0004369 | CD99L2 | 0.778763 | 0.000107 |
| chr12:70149164-70150443+ | hsa_circ_0099132 | RAB3IP | 0.928838 | 0.0013 |
| chr6:80198801-80203467- | hsa_circ_0132366 | LCA5 | 0.96803 | 0.00249 |
| chr19:39408364-39409453- | hsa_circ_0050954 | SARS2 | 0.621388 | 0.016308 |
| chr11:120916383-120924441+ | hsa_circ_0000365 | TBCEL | -0.28758 | 0.028142 |
| chr3:27420740-27424741- | hsa_circ_0064614 | SLC4A7 | 0.581959 | 0.031266 |
| chr13:46090278-46093229+ | hsa_circ_0003401 | COG3 | -0.22001 | 0.015032 |
| chr12:28408514-28460682+ | hsa_circ_0098286 | CCDC91 | -0.2039 | 0.033658 |
| chr5:77684661-77755185+ | hsa_circ_0129697 | SCAMP1 | -0.25758 | 0.039476 |
| chr15:59179174-59179739- | hsa_circ_0000604 | SLTM | 0.310433 | 0.021912 |
| chr7:148716084-148718239- | hsa_circ_0001766 | PDIA4 | 0.254499 | 0.043435 |
| chr2:61712903-61717911- | hsa_circ_0005050 | XPO1 | -0.18827 | 0.024862 |
| chr15:66030045-66031213- | hsa_circ_0035949 | DENND4A | -0.28432 | 0.007447 |
| chr17:169211-171206- | hsa_circ_0041152 | RPH3AL | 0.96206 | 0.000105 |
| chr8:98725890-98731417+ | hsa_circ_0003117 | MTDH | 0.469951 | 0.016801 |
| chr20:19560644-19566188+ | hsa_circ_0114779 | SLC24A3 | 0.603357 | 0.017149 |
| chr17:80721841-80730383+ | hsa_circ_0005281 | TBCD | -0.31194 | 0.025188 |
| chr16:2369582-2369841- | hsa_circ_0037516 | ABCA3 | 0.626535 | 0.045872 |
| chr5:67522118-67522837+ | hsa_circ_0006411 | PIK3R1 | 0.214961 | 0.043748 |
| chr6:16326625-16328701- | hsa_circ_0007132 | ATXN1 | -0.19265 | 0.015162 |
| chr15:40920268-40943017+ | hsa_circ_0103435 | CASC5 | 0.284602 | 0.025467 |
| chr2:179400459-179407088+ | hsa_circ_0004305 | TTN-AS1 | -0.20324 | 0.034521 |
| chr6:117013218-117047785+ | hsa_circ_0006146 | KPNA5 | -0.25485 | 0.012245 |
| chr1:41651793-41660071- | hsa_circ_0005303 | SCMH1 | -0.24847 | 0.021101 |
| chr16:53301206-53302038+ | hsa_circ_0008031 | CHD9 | 0.370527 | 0.030668 |
| chr1:35936464-35944813- | hsa_circ_0011571 | KIAA0319L | -0.19041 | 0.043679 |
| chr7:72617463-72618644+ | hsa_circ_0002284 | GTF2IP4 | -0.21506 | 0.018751 |
| chr22:30374431-30387659+ | hsa_circ_0002954 | MTMR3 | -0.30353 | 0.008469 |
| chr14:45711237-45716580- | hsa_circ_0101892 | MIS18BP1 | 0.595952 | 0.010781 |
| chr22:32007129-32007826+ | hsa_circ_0062923 | SFI1 | 0.56128 | 0.026707 |
| chr6:29910550-29974592+ | | HLA-G | -0.31087 | 0.029699 |
| chr4:83795764-83803093- | hsa_circ_0003716 | SEC31A | -0.24411 | 0.030519 |
| chr10:7409611-7412337- | hsa_circ_0017647 | SFMBT2 | -0.24519 | 0.047795 |
| chr10:95140976-95148911- | hsa_circ_0005898 | MYOF | 0.529456 | 0.007879 |
| chr14:45618040-45628483+ | hsa_circ_0101878 | FANCM | 0.401673 | 0.026072 |
| chr17:40497577-40500535- | hsa_circ_0043815 | STAT3 | 0.346429 | 0.02729 |
| chr2:20454624-20455931- | hsa_circ_0005952 | PUM2 | -0.28002 | 0.036919 |
| chr19:8612920-8613201- | hsa_circ_0110084 | MYO1F | -0.29634 | 0.003767 |
| chr2:10559860-10560261+ | hsa_circ_0000976 | HPCAL1 | -0.19358 | 0.041135 |
| chr17:19823349-19839743- | hsa_circ_0106369 | AKAP10 | 1.08157 | 0.000211 |
| chr17:76778284-76834832- | | CYTH1 | 1.121811 | 0.000291 |
| chr22:23613719-23615961+ | hsa_circ_0116261 | BCR | 0.767385 | 0.00359 |
| chr17:65941525-65960520+ | hsa_circ_0045470 | BPTF | -0.30945 | 0.007722 |
| chr22:41521868-41537226+ | hsa_circ_0116613 | EP300 | 0.519633 | 0.046886 |
| chr6:82920531-82922510- | hsa_circ_0002041 | IBTK | -0.20763 | 0.03672 |
| chr1:224553581-224559125+ | hsa_circ_0000190 | CNIH4 | 0.360032 | 0.022349 |
| chr14:75245150-75249028+ | | YLPM1 | 0.317367 | 0.039808 |
| chr2:61389977-61391675+ | hsa_circ_0005338 | C2orf74 | -0.32571 | 0.015594 |
| chr5:177058367-177059739- | | LOC202181 | -0.25951 | 0.026455 |
| chr1:230798887-230807386+ | hsa_circ_0016867 | COG2 | -0.23101 | 0.034622 |
| chr3:196842798-196846401- | hsa_circ_0001383 | DLG1 | -0.22792 | 0.025885 |
| chr10:7409611-7423911- | hsa_circ_0017648 | SFMBT2 | -0.23318 | 0.026541 |
| chr16:4700366-4707364+ | hsa_circ_0007032 | MGRN1 | 0.275726 | 0.044278 |
| chr3:149563798-149639014+ | hsa_circ_0001346 | RNF13 | -0.15295 | 0.029919 |
| chrX:147733520-147744289+ | hsa_circ_0091669 | AFF2 | -0.17999 | 0.009842 |
| chr11:119144578-119145663+ | hsa_circ_0000362 | CBL | -0.18612 | 0.046204 |
| chr18:9593766-9595100- | hsa_circ_0108984 | PPP4R1 | 0.763801 | 0.00176 |
| chr21:38439562-38441924- | hsa_circ_0008160 | PIGP | 0.567826 | 0.022671 |
| chr10:98667022-98667504+ | hsa_circ_0007707 | LCOR | 0.438522 | 0.02502 |
| chr2:26505713-26505919+ | hsa_circ_0119637 | HADHB | -0.3222 | 0.025167 |
| chr2:61552510-61561106- | hsa_circ_0120625 | USP34 | -0.27101 | 0.027728 |
| chr1:21220010-21231464- | hsa_circ_0007214 | EIF4G3 | 0.572829 | 0.030005 |
| chr7:73649862-73661093- | hsa_circ_0134619 | RFC2 | 0.525543 | 0.031705 |
| chr1:203816312-203816815+ | hsa_circ_0016115 | ZC3H11A | 0.661044 | 0.036065 |
| chr7:72604106-72607065+ | hsa_circ_0007927 | GTF2IP4 | -0.25129 | 0.039585 |
| chr2:85262139-85262970+ | hsa_circ_0121085 | KCMF1 | -0.31452 | 0.044281 |
| chr8:142146608-142148236+ | hsa_circ_0135933 | DENND3 | -0.29883 | 0.045096 |
| chr10:11971864-11994248- | hsa_circ_0092831 | UPF2 | -0.29772 | 0.045221 |
| chr2:44436349-44445676+ | hsa_circ_0009062 | PPM1B | -0.20909 | 0.015818 |
| chr6:150059779-150063706- | hsa_circ_0078241 | NUP43 | 0.372974 | 0.026186 |
| chr5:176618885-176631293+ | hsa_circ_0075157 | NSD1 | -0.286 | 0.029587 |
| chr6:118832461-118887479- | hsa_circ_0077736 | CEP85L | 0.295899 | 0.033029 |
| chr11:120916383-120930794+ | hsa_circ_0003302 | TBCEL | -0.17704 | 0.019309 |
| chr4:156696120-156698794+ | hsa_circ_0125610 | GUCY1B3 | 1.068031 | 0.001047 |
| chr16:67644727-67655494+ | hsa_circ_0105757 | CTCF | 0.88073 | 0.003523 |
| chr17:45479498-45517868+ | | EFCAB13 | 0.653186 | 0.023615 |
| chr6:110036281-110064975+ | hsa_circ_0077607 | FIG4 | -0.33852 | 0.024807 |
| chr15:65773853-65780156- | hsa_circ_0104287 | DPP8 | -0.29893 | 0.027565 |
| chr7:92146590-92148392- | hsa_circ_0081028 | PEX1 | 0.578597 | 0.029245 |
| chr16:89961446-89967202+ | hsa_circ_0002631 | TCF25 | -0.27594 | 0.045087 |
| chr1:58999621-59004978- | hsa_circ_0113708 | DAB1 | -0.29361 | 0.045627 |
| chr14:70125230-70125430+ | hsa_circ_0032352 | SUSD6 | 0.529416 | 0.04879 |
| chr9:88430794-88444521+ | | LOC389765 | 0.671579 | 0.007845 |
| chr22:46493806-46494438+ | hsa_circ_0063776 | MIRLET7BHG | 0.56457 | 0.012568 |
| chr5:86627165-86633908+ | hsa_circ_0007507 | RASA1 | -0.26874 | 0.015216 |
| chr14:91808724-91810001- | hsa_circ_0032959 | CCDC88C | -0.25657 | 0.039665 |
| chr12:56742313-56743420- | hsa_circ_0008085 | STAT2 | -0.30569 | 0.042307 |
| chr18:44470543-44483598- | hsa_circ_0108510 | PIAS2 | -0.25064 | 0.046346 |
| chr14:91759396-91759801- | | CCDC88C | 0.226955 | 0.039891 |
| chrX:147743429-147744289+ | hsa_circ_0001947 | AFF2 | -0.16855 | 0.00987 |
| chr5:70331372-70333373- | | NAIP | -0.13819 | 0.029136 |
| chr17:30498062-30510291+ | hsa_circ_0106601 | RHOT1 | 0.258656 | 0.032819 |
| chr13:52992127-53000201- | hsa_circ_0030340 | VPS36 | -0.22218 | 0.041108 |
| chr15:65268813-65275931- | hsa_circ_0035873 | SPG21 | -0.30768 | 0.027783 |
| chr3:48960181-48965246+ | hsa_circ_0006838 | ARIH2 | 0.334452 | 0.019652 |
| chr8:142154247-142178624+ | | DENND3 | 1.640686 | 9.04E-05 |
| chr4:128904094-128942393+ | hsa_circ_0125279 | C4orf29 | 1.001113 | 0.000135 |
| chr14:91759396-91759683- | | CCDC88C | 0.919047 | 0.001203 |
| chr15:40475915-40477843+ | hsa_circ_0008471 | BUB1B | 0.745667 | 0.002112 |
| chr6:130374004-130442103+ | | L3MBTL3 | 0.772615 | 0.004891 |
| chr2:107051093-107051658- | | RGPD3 | 0.840848 | 0.010951 |
| chr3:131186935-131190123- | hsa_circ_0004559 | MRPL3 | -0.35345 | 0.026644 |
| chr20:31979948-31981872- | hsa_circ_0059855 | CDK5RAP1 | 0.477026 | 0.033124 |
| chr2:198388348-198400354+ | hsa_circ_0057680 | MOB4 | -0.33886 | 0.043392 |
| chr1:78183552-78184326- | hsa_circ_0012992 | USP33 | -0.28852 | 0.045098 |
| chr4:83375875-83378191+ | hsa_circ_0003451 | ENOPH1 | -0.25514 | 0.04737 |
| chr14:58785260-58796887+ | hsa_circ_0000542 | ARID4A | -0.20356 | 0.019799 |
| chr9:3647338-3651867+ | hsa_circ_0138738 | RP11-509J21.1 | -0.3045 | 0.02705 |
| chr3:121215655-121217517- | hsa_circ_0121608 | POLQ | -0.27335 | 0.033263 |
| chr1:40654727-40668286+ | hsa_circ_0006373 | RLF | -0.19301 | 0.043625 |
| chr2:38536521-38546161- | hsa_circ_0002918 | ATL2 | -0.20295 | 0.033024 |
| chr1:31465237-31468067- | hsa_circ_0000043 | PUM1 | -0.16887 | 0.045235 |
| chr6:160467530-160469575+ | hsa_circ_0131235 | IGF2R | -0.25116 | 0.0176 |
| chr16:53288350-53308214+ | hsa_circ_0000702 | CHD9 | 0.276819 | 0.047074 |
| chr18:196637-199316+ | hsa_circ_0007706 | USP14 | -0.24049 | 0.047191 |
| chr10:88211724-88221044- | hsa_circ_0019005 | WAPL | 0.239845 | 0.049358 |
| chr12:1480999-1553916+ | hsa_circ_0006747 | ERC1 | -0.20897 | 0.048996 |
| chr5:43292576-43297268- | hsa_circ_0008621 | HMGCS1 | -0.30012 | 0.020007 |
| chr3:172003716-172028671+ | hsa_circ_0002422 | FNDC3B | -0.25878 | 0.027608 |
| chr16:30492760-30495584+ | | ITGAL | 0.443636 | 0.04182 |
| chrX:76888695-76912143- | hsa_circ_0140608 | ATRX | 1.539173 | 0.000127 |
| chr5:126140468-126161799+ | hsa_circ_0073747 | LMNB1 | 0.929414 | 0.000299 |
| chr2:171884849-171917666- | | TLK1 | 1.106585 | 0.001908 |
| chrX:117530933-117538407+ | hsa_circ_0001939 | WDR44 | 0.84892 | 0.001914 |
| chr12:133083838-133084936+ | hsa_circ_0002826 | FBRSL1 | 0.878552 | 0.002032 |
| chr6:16614591-16615701- | | ATXN1 | 0.898436 | 0.003794 |
| chr1:154145384-154145677- | hsa_circ_0008368 | TPM3 | 0.685633 | 0.00414 |
| chr17:3608392-3608898- | hsa_circ_0106743 | GSE61474_XLOC_026870 | 0.739358 | 0.00723 |
| chrM:1680-12080+ | | TVAS5 | 0.78042 | 0.031882 |
| chr11:76207259-76239510+ | hsa_circ_0007583 | C11orf30 | -0.28453 | 0.042775 |
| chr5:55256230-55264224- | hsa_circ_0007304 | IL6ST | -0.27692 | 0.043717 |
| chr19:8601136-8604912- | hsa_circ_0000886 | MYO1F | -0.31887 | 0.045101 |
| chr6:17665470-17669777- | hsa_circ_0007268 | NUP153 | -0.34183 | 0.04548 |
| chr14:50298769-50301167- | hsa_circ_0031814 | NEMF | -0.33462 | 0.045928 |
| chr2:69581621-69590802- | hsa_circ_0003808 | GFPT1 | -0.29804 | 0.046266 |
| chr7:151478238-151483627- | | PRKAG2 | -0.30446 | 0.04805 |
| chr7:156619299-156629579- | hsa_circ_0005939 | LMBR1 | -0.18744 | 0.027753 |
| chrX:154736559-154766779- | hsa_circ_0006355 | TMLHE | -0.20103 | 0.020605 |
| chr8:61484605-61504528+ | hsa_circ_0007581 | RAB2A | -0.18721 | 0.048746 |
| chr11:62303417-62304039- | hsa_circ_0008194 | AHNAK | 0.50063 | 0.010596 |
| chr16:18851020-18853776- | hsa_circ_0008216 | SMG1 | -0.24699 | 0.02264 |
| chr22:38641941-38644025- | hsa_circ_0005243 | TMEM184B | 0.388885 | 0.025992 |
| chr14:50136242-50141145- | hsa_circ_0004904 | POLE2 | -0.22262 | 0.029777 |
| chr9:128099297-128099870+ | hsa_circ_0003270 | GAPVD1 | -0.20687 | 0.044932 |
| chr3:197592294-197602646+ | hsa_circ_0008351 | LRCH3 | -0.23223 | 0.007034 |
| chr11:82989769-82991303- | hsa_circ_0023865 | CCDC90B | 0.541371 | 0.003917 |
| chr6:99347144-99365595- | hsa_circ_0132765 | FBXL4 | 0.561397 | 0.016032 |
| chr13:50601343-50619368- | | DLEU2 | 0.433671 | 0.026047 |
| chr11:62295055-62295639- | | AHNAK | 0.464373 | 0.02843 |
| chr2:64189192-64211153- | hsa_circ_0054958 | VPS54 | -0.25079 | 0.043974 |
| chr2:55250507-55250695- | | RTN4 | 0.435556 | 0.047855 |
| chr7:91700219-91715000+ | hsa_circ_0135024 | AKAP9 | 0.336213 | 0.048488 |
| chr7:17908030-17937069- | hsa_circ_0004671 | SNX13 | -0.1866 | 0.021776 |
| chr1:117944808-118009049+ | hsa_circ_0000120 | MAN1A2 | 0.40569 | 0.026147 |
| chr7:140476712-140508795- | hsa_circ_0007178 | BRAF | -0.25183 | 0.027482 |
| chr4:129042981-129083472+ | hsa_circ_0125310 | LARP1B | 1.094807 | 7.54E-05 |
| chr6:146202764-146216113- | hsa_circ_0130935 | SHPRH | 1.524874 | 0.000154 |
| chr7:2482182-2483381+ | | AC004840.9 | 1.152978 | 0.000173 |
| chr4:186096940-186097205- | hsa_circ_0004697 | CFAP97 | 1.214156 | 0.000215 |
| chrX:24080533-24086225+ | | EIF2S3 | 1.059668 | 0.003199 |
| chr5:171516809-171516996- | | STK10 | 0.901982 | 0.003353 |
| chr7:5778907-5781446- | hsa_circ_0007501 | RNF216 | 0.727968 | 0.005094 |
| chr2:68717322-68794519+ | hsa_circ_0055021 | APLF | 0.890108 | 0.006439 |
| chr11:78270585-78282489- | hsa_circ_0023815 | NARS2 | 0.596886 | 0.007786 |
| chr9:37302185-37327831+ | hsa_circ_0087051 | ZCCHC7 | 0.67608 | 0.015361 |
| chr19:11548697-11548945+ | hsa_circ_0049462 | PRKCSH | 0.594257 | 0.019513 |
| chr19:1031070-1032695+ | hsa_circ_0004003 | CNN2 | 0.573019 | 0.019626 |
| chr9:5689959-5720750+ | hsa_circ_0005707 | RIC1 | 0.650191 | 0.023283 |
| chr17:4575409-4575567- | | PELP1 | 0.606429 | 0.023885 |
| chr17:45405635-45422464+ | hsa_circ_0044241 | EFCAB13 | -0.31411 | 0.025244 |
| chrX:46712911-46713576+ | | RP2 | 0.651502 | 0.025911 |
| chr10:1141138-1141306+ | | WDR37 | 0.481223 | 0.030517 |
| chr15:64404772-64415745+ | hsa_circ_0002178 | SNX1 | 0.599876 | 0.031289 |
| chr12:133428204-133430159- | hsa_circ_0029589 | CHFR | 0.553825 | 0.032936 |
| chrX:109263580-109263891+ | | TMEM164 | 0.404629 | 0.038339 |
| chr15:52192352-52194233+ | hsa_circ_0004773 | TMOD3 | 0.465277 | 0.039221 |
| chr13:41400642-41411021- | hsa_circ_0030049 | TPTE2P5 | -0.2822 | 0.041892 |
| chrX:122820398-122831601- | hsa_circ_0139689 | THOC2 | -0.29391 | 0.042652 |
| chr12:938228-939110+ | hsa_circ_0005616 | WNK1 | -0.27488 | 0.04407 |
| chr4:89570991-89579642+ | hsa_circ_0006387 | HERC3 | -0.3147 | 0.046992 |
| chr17:17165280-17168295- | hsa_circ_0042253 | COPS3 | -0.29506 | 0.048337 |
| chr10:22002701-22016857+ | hsa_circ_0007084 | MLLT10 | -0.21656 | 0.038571 |
| chr7:23224689-23226765+ | hsa_circ_0001683 | NUPL2 | 0.204064 | 0.035639 |
| chr7:151946961-151948051- | hsa_circ_0005823 | KMT2C | -0.18607 | 0.027512 |
| chr12:95602619-95605043- | hsa_circ_0099549 | FGD6 | 0.221774 | 0.017321 |
| chr15:50940884-50955243- | hsa_circ_0007775 | TRPM7 | -0.21366 | 0.020892 |
| chr17:19823349-19827830- | hsa_circ_0106367 | AKAP10 | 0.5526 | 0.010659 |
| chr3:183432932-183442319+ | hsa_circ_0122963 | YEATS2 | 0.665872 | 0.016008 |
| chr2:111423840-111431942- | hsa_circ_0056040 | BUB1 | -0.30044 | 0.02296 |
| chr17:30267305-30300250+ | hsa_circ_0005804 | SUZ12 | -0.20919 | 0.04266 |
| chr16:18809247-18810156- | hsa_circ_0004833 | ARL6IP1 | -0.26769 | 0.020491 |
| chr17:80521230-80529746+ | hsa_circ_0000817 | FOXK2 | -0.27386 | 0.032341 |
| chr1:156303338-156304709- | hsa_circ_0004680 | CCT3 | -0.23921 | 0.045998 |
| chr17:45438744-45456619+ | hsa_circ_0106983 | EFCAB13 | -0.204 | 0.011853 |
| chr9:97717459-97741709+ | hsa_circ_0139391 | C9orf3 | 2.342374 | 1.48E-06 |
| chr3:171944661-171965566+ | hsa_circ_0067984 | FNDC3B | 1.93797 | 6.31E-06 |
| chr6:18160114-18163155+ | hsa_circ_0131407 | KDM1B | 2.107529 | 6.56E-06 |
| chr10:73887840-73892939- | hsa_circ_0008195 | ASCC1 | 1.621469 | 2.64E-05 |
| chr1:93691890-93698150+ | hsa_circ_0013200 | CCDC18 | 1.340282 | 2.84E-05 |
| chrM:8626-8785+ | | OK/SW-cl.16 | 1.828505 | 3.18E-05 |
| chr12:104856918-104857102+ | | CHST11 | 1.582774 | 9.21E-05 |
| chr4:3514406-3526778- | | LRPAP1 | 1.423773 | 0.000148 |
| chr13:95813443-95822882- | hsa_circ_0006659 | ABCC4 | 1.461374 | 0.000395 |
| chr6:22020568-22056919+ | hsa_circ_0075829 | CASC15 | 0.939953 | 0.000942 |
| chr10:98644160-98667504+ | hsa_circ_0094657 | LCOR | 0.795549 | 0.002909 |
| chr5:137897269-137903411- | hsa_circ_0008356 | HSPA9 | 0.791863 | 0.005665 |
| chr7:959605-966280- | hsa_circ_0079091 | ADAP1 | 0.846456 | 0.008937 |
| chr16:11940358-11944275- | hsa_circ_0037891 | RSL1D1 | 0.789964 | 0.009805 |
| chr1:146727468-146731572+ | hsa_circ_0013947 | CHD1L | 0.698775 | 0.009843 |
| chr7:155499554-155538296+ | | RBM33 | -0.36683 | 0.012447 |
| chr6:2836090-2836257- | hsa_circ_0131620 | SERPINB1 | -0.35944 | 0.015276 |
| chr22:19948722-19951822+ | hsa_circ_0062277 | COMT | 0.857246 | 0.015649 |
| chr3:18504972-18568818+ | hsa_circ_0123034 | SATB1-AS1 | -0.32698 | 0.023626 |
| chr16:66642212-66643906+ | hsa_circ_0008450 | CMTM3 | -0.32823 | 0.024268 |
| chr11:75715049-75728024+ | hsa_circ_0096463 | UVRAG | 0.808458 | 0.02499 |
| chr10:26800676-26802589+ | hsa_circ_0093391 | APBB1IP | 0.550569 | 0.03679 |
| chr14:24735636-24737825- | hsa_circ_0007750 | RABGGTA | -0.31099 | 0.041799 |
| chr2:203155046-203160560+ | hsa_circ_0008327 | NOP58 | -0.30042 | 0.04304 |
| chr10:35302627-35320547- | hsa_circ_0093600 | CUL2 | 0.522297 | 0.043251 |
| chr15:89656963-89659752+ | hsa_circ_0003679 | ABHD2 | -0.3046 | 0.044265 |
| chr2:10784446-10808849- | hsa_circ_0000977 | NOL10 | 0.588483 | 0.045189 |
| chr1:231500073-231503382- | hsa_circ_0112381 | EGLN1 | -0.30772 | 0.046087 |
| chr9:123751324-123753558- | hsa_circ_0137815 | C5 | -0.32263 | 0.04631 |
| chr7:6859397-6861995- | hsa_circ_0079410 | CCZ1B | -0.3268 | 0.047393 |
| chr5:77385217-77425131- | hsa_circ_0008164 | AP3B1 | -0.18604 | 0.033172 |
| chr1:155891166-155891709- | hsa_circ_0008998 | KIAA0907 | -0.21013 | 0.036671 |
| chr22:46085592-46136418+ | hsa_circ_0008199 | ATXN10 | -0.23541 | 0.029211 |
| chr18:12356693-12371690- | hsa_circ_0046965 | AFG3L2 | -0.24052 | 0.025384 |
| chr4:39875909-39878773- | hsa_circ_0008312 | PDS5A | 0.502482 | 0.011511 |
| chr14:89041037-89044484+ | hsa_circ_0003164 | ZC3H14 | -0.27979 | 0.03081 |
| chr15:74911538-74912566+ | hsa_circ_0036287 | CLK3 | 0.522103 | 0.033741 |
| chr11:34952951-35006275+ | hsa_circ_0021708 | PDHX | -0.26466 | 0.045742 |
| chr16:74493580-74497377- | hsa_circ_0003315 | GLG1 | 0.44991 | 0.01091 |
| chr5:80832348-80833211- | hsa_circ_0129832 | SSBP2 | 0.391251 | 0.019499 |
| chr5:39002637-39021238- | hsa_circ_0001475 | RICTOR | 0.335198 | 0.047853 |

Table S6. Gene Ontology and pathway analysis of survival associated circRNAs

| GO ID | Name | p-value |
| --- | --- | --- |
| GO:0051020 | GTPase binding | 7.61E-07 |
| GO:0003723 | RNA binding | 2.02E-06 |
| GO:0005049 | nuclear export signal receptor activity | 9.10E-06 |
| GO:0017016 | Ras GTPase binding | 1.21E-05 |
| GO:0008536 | Ran GTPase binding | 1.76E-05 |
| GO:0031267 | small GTPase binding | 1.89E-05 |
| GO:0140142 | nucleocytoplasmic carrier activity | 4.95E-05 |
| GO:0016772 | transferase activity, transferring phosphorus-containing groups | 1.95E-04 |
| GO:0005085 | guanyl-nucleotide exchange factor activity | 2.17E-04 |
| GO:0005524 | ATP binding | 2.87E-04 |
| GO:0032559 | adenyl ribonucleotide binding | 3.37E-04 |
| GO:0030554 | adenyl nucleotide binding | 3.86E-04 |
| GO:0016835 | carbon-oxygen lyase activity | 4.11E-04 |
| GO:0044389 | ubiquitin-like protein ligase binding | 4.17E-04 |
| GO:0019787 | ubiquitin-like protein transferase activity | 5.30E-04 |
| GO:0045296 | cadherin binding | 5.86E-04 |
| GO:0035639 | purine ribonucleoside triphosphate binding | 6.08E-04 |
| GO:0017111 | nucleoside-triphosphatase activity | 6.15E-04 |
| GO:0032555 | purine ribonucleotide binding | 8.24E-04 |
| GO:0031625 | ubiquitin protein ligase binding | 8.47E-04 |
| GO:0032553 | ribonucleotide binding | 9.80E-04 |
| GO:0004842 | ubiquitin-protein transferase activity | 1.02E-03 |
| GO:0017076 | purine nucleotide binding | 1.02E-03 |
| GO:0003887 | DNA-directed DNA polymerase activity | 1.13E-03 |
| GO:0016462 | pyrophosphatase activity | 1.17E-03 |
| GO:0016817 | hydrolase activity, acting on acid anhydrides | 1.19E-03 |
| GO:0016818 | hydrolase activity, acting on acid anhydrides, in phosphorus-containing anhydrides | 1.19E-03 |
| GO:0061659 | ubiquitin-like protein ligase activity | 1.22E-03 |
| GO:0050839 | cell adhesion molecule binding | 1.72E-03 |
| GO:0017112 | Rab guanyl-nucleotide exchange factor activity | 1.87E-03 |
| Pathways | Name | p-value |
| REACTOME | Antiviral mechanism by IFN-stimulated genes | 1.60E-05 |
| REACTOME | ISG15 antiviral mechanism | 1.60E-05 |
| REACTOME | Cell Cycle, Mitotic | 2.19E-05 |
| REACTOME | Cell Cycle | 4.04E-05 |
| REACTOME | SUMOylation of RNA binding proteins | 6.65E-05 |
| REACTOME | Membrane Trafficking | 9.11E-05 |
| Pathway Interaction Database | CXCR4-mediated signaling events | 1.19E-04 |
| REACTOME | Interferon Signaling | 1.87E-04 |
| REACTOME | M Phase | 1.92E-04 |
| KEGG | RNA transport | 2.07E-04 |
| REACTOME | Mitotic Prometaphase | 2.20E-04 |
| REACTOME | Vesicle-mediated transport | 2.55E-04 |
| MSigDB C2 BIOCARTA (v7.1) | Role of ERBB2 in Signal Transduction and Oncology | 3.16E-04 |
| REACTOME | Mitotic Anaphase | 3.27E-04 |
| REACTOME | Mitotic Metaphase and Anaphase | 3.42E-04 |
| REACTOME | Interleukin-6 signaling | 4.66E-04 |
| REACTOME | Cohesin Loading onto Chromatin | 4.66E-04 |
| MSigDB C2 BIOCARTA (v7.1) | Sumoylation by RanBP2 regulates transcriptional repression | 4.66E-04 |
| Pathway Interaction Database | Sumoylation by RanBP2 regulates transcriptional repression | 4.66E-04 |
| REACTOME | Intra-Golgi traffic | 4.82E-04 |

Table S7. Different expression of circRNAs which encoding genes enriched in the GO:0016835 term.

| CircRNAID | circBaseID | Gene Name | logFC | P Value1 | FDR | P value2 | Beta |
| --- | --- | --- | --- | --- | --- | --- | --- |
| chr17:40497577-40500535- | hsa_circ_0043815 | STAT3 | 1.98994 | 0.000597 | 0.008184 | 0.02729 | 0.346429 |
| chr17:80521230-80529746+ | hsa_circ_0000817 | FOXK2 | -1.42828 | 0.029794 | 0.148004 | 0.032341 | -0.27386 |
| chr2:26505713-26505919+ | hsa_circ_0119637 | HADHB | -2.28203 | 0.001489 | 0.01632 | 0.025167 | -0.3222 |
| chr2:68717322-68794519+ | hsa_circ_0055021 | APLF | 1.59706 | 0.018448 | 0.102579 | 0.006439 | 0.890108 |
| chr3:121215655-121217517- | hsa_circ_0121608 | POLQ | -1.67953 | 0.005209 | 0.039808 | 0.033263 | -0.27335 |
| chr6:2116005-2124965- | hsa_circ_0075451 | GMDS | 3.286526 | 2.57E-08 | 2.70E-06 | 0.038205 | 0.307593 |
| chr6:17665470-17669777- | hsa_circ_0007268 | NUP153 | -1.77092 | 0.009732 | 0.064011 | 0.04548 | -0.34183 |
| chr6:150059779-150063706- | hsa_circ_0078241 | NUP43 | 1.564894 | 0.001577 | 0.016897 | 0.026186 | 0.372974 |
| chr7:23224689-23226765+ | hsa_circ_0001683 | NUPL2 | 0.869976 | 0.020314 | 0.110063 | 0.035639 | 0.204064 |
| chr7:151478238-151483627- | | PRKAG2 | -1.83464 | 0.009732 | 0.064011 | 0.04805 | -0.30446 |

Log FC, p value1 and FDR are results of the differently expression analyses taken patients into 2 groups, while p value2 and Beta are results from Cox regression analysis.

Table S8. Characteristics of CN-AML patients with high and low hsa_circ_0075451 expression in the validated cohort.

| Variable | Low | High | P value |
| --- | --- | --- | --- |
| Number | 102 | 116 |  |
| Sex, male,n(%) | 56 (54.9) | 71 (61.2) | 0.409 |
| Age,median(range),years | 53.50 [40.25, 63.75] | 51.50 [38.75, 63.00] | 0.587 |
| WBC,median(IQR),×10^9/L^1^ | 13.40 [3.70, 52.00] | 16.60 [3.77, 67.10] | 0.433 |
| HB,median(IQR),g/L^2^ | 85.50 [70.25, 106.00] | 84.00 [64.00, 101.00] | 0.088 |
| PLT,median(IQR),×10^9/L^3^ | 60.50 [24.25, 90.00] | 46.00 [26.00, 77.25] | 0.378 |
| BM blast,median(IQR),%^4^ | 66.50 [41.75, 82.00] | 70.00 [49.00, 85.00] | 0.414 |
| FAB classification,n(%)^5^ | |  | 0.069 |
| M0 | 14 (13.7) | 12 (10.3) | |
| M1 | 8 ( 7.8) | 16 (13.8) | |
| M2 | 41 (40.2) | 62 (53.4) | |
| M4 | 6 ( 5.9) | 3 ( 2.6) | |
| M5 | 32 (31.4) | 23 (19.8) | |
| M6 | 1 ( 1.0) | 0 ( 0.0) | |
| Genemutations,n(%) | |  |  |
| FLT3-ITD | 19 (18.6) | 26 (22.4) | 0.508 |
| NPM1 | 26 (26.5) | 28 (25.2) | 0.875 |
| CEBPA^DM6^ | 19 (20.0) | 15 (14.0) | 0.266 |
| DNMT3A | 14 (15.1) | 14 (13.0) | 0.688 |
| IDH1 | 13 (14.4) | 19 (17.3) | 0.699 |
| IDH2 | 9 (10.6) | 15 (14.3) | 0.514 |
| ENL Favorable | 33 (32.4) | 26 (22.4) | 0.126 |
| Treatment (%) | |  | 0.123 |
| DA | 28 (27.5) | 27 (23.3) | |
| HAA | 12 (11.8) | 26 (22.4) | |
| IA | 62 (60.8) | 63 (54.3) | |
| CR | 80 (78.4) | 85 (73.3) | 0.43 |

Abbreviations: ^1^WBC, white blood cell counts; ^2^HB, hemoglobin; ^3^PLT, platelet counts; ^4^BM, bone marrow; ^5^ FAB, Franch-American-British;^6^DM, double allele. IQR, interquantile. ELN (European leukemia Net) favorable genotype represents *NPM1* mutant and *FLT3*-ITD negative or double allele *CEBPA* mutations. CR, complete remission rate. HAA, homoharringtonin-based treatment (homoharringtonin 2 mg/m2 /day for 3 days, cytarabine 75 mg/m2 twice daily for 7 days, aclarubicin 12 mg/m2 daily for 7 days) regiment; DA, daunorubicin 45 mg/m2 daily for 3 days and cytarabine 100 mg/m2 daily for 7 days; IA, idarubicin 8-10 mg/m2 daily for 3 days and cytarabine 100 mg/m2 daily for 7 days.

Table S9. Landmark analysis of CN-AML patients

| **Variables** | **Pvalue** | **HR(95%CI)** |  |
| --- | --- | --- | --- |
| **CircRNA** | 0.02 | 1.682(1.086,2.607) | |
| **Age** | 0.035 | 1.641(1.036,2.599) | |
| **WBC** | 0.212 | 1.002(0.999,1.006) | |
| **ELN favorable genotype** | 0.05 | 0.575(0.33,0.999) | |
| ***DNMT3A*** | 0.009 | 2.132(1.207,3.765) | |
| ***IDH1*** | 0.963 | 0.984(0.505,1.917) | |
| ***IDH2*** | 0.14 | 0.589(0.291,1.189) | |
| **Treatment** | |  |  |
| **HAA vs. DA** | 0.471 | 0.781(0.399,1.529) | |
| **IA vs. DA** | 0.125 | 0.671(0.404,1.117) | |

Abbreviations: CircRNA represents hsa_circ_0075451 high expressers vs. low expressers. WBC, white blood cell counts; ELN (European leukemia Net) favorable genotype represents *NPM1* mutant and *FLT3*-ITD negative or double allele *CEBPA* mutations. HAA, homoharringtonin-based treatment (homoharringtonin 2 mg/m2 /day for 3 days, cytarabine 75 mg/m2 twice daily for 7 days, aclarubicin 12 mg/m2 daily for 7 days) regiment; DA, daunorubicin 45 mg/m2 daily for 3 days and cytarabine 100 mg/m2 daily for 7 days; IA, idarubicin 8-10 mg/m2 daily for 3 days and cytarabine 100 mg/m2 daily for 7 days.

Table S10. Clinical characteristics of patients for mRNA profiling analysis

| Number | Low (N=7) | High (N=7) | P value |
| --- | --- | --- | --- |
| Sex, male, n(%) | 4 ( 57.1) | 5 ( 71.4) | 1.000 |
| Age, median(range),years | 41.00 [27.00, 52.50] | 51.00 [33.50, 54.50] | 0.653 |
| WBC, median(IQR),×10^9/L^1^ | 44.70 [8.75, 56.70] | 9.00 [4.35, 21.60] | 0.18 |
| HB, median(IQR),g/L^2^ | 88.00 [75.00, 95.50] | 74.00 [65.50, 118.00] | 0.848 |
| PLT, median(IQR),×10^9/L^3^ | 53.00 [39.00, 97.00] | 88.00 [36.00, 130.00] | 0.848 |
| BM blast, median(IQR),%^4^ | 57.00 [51.50, 75.50] | 63.00 [56.00, 78.00] | 0.655 |
| FAB classification,n(%)^5^ | |  | 0.662 |
| M0 | 0 ( 0.0) | 1 ( 14.3) | |
| M1 | 1 ( 14.3) | 0 ( 0.0) | |
| M2 | 2 ( 28.6) | 4 ( 57.1) | |
| M4 | 1 ( 14.3) | 1 ( 14.3) | |
| M5 | 3 ( 42.9) | 1 ( 14.3) | |
| Genemutations,n(%) | |  |  |
| FLT3-ITD | 1 ( 14.3) | 2 ( 28.6) | 1.000 |
| NPM1 | 3 ( 42.9) | 1 ( 14.3) | 0.559 |
| CEBPA^DM6^ | 3 ( 42.9) | 0 ( 0.0) | 0.192 |
| DNMT3A | 2 ( 28.6) | 0 ( 0.0) | 0.462 |
| IDH1 | 1 ( 14.3) | 1 ( 14.3) | 1.000 |
| IDH2 | 1 ( 14.3) | 1 ( 14.3) | 1.000 |

Table S11. mRNAs expression between high and low hsa_circ_0075451

| Symbols | Gene IDs | log2FC | log2CPM | P Value | FDR |
| --- | --- | --- | --- | --- | --- |
| MT-CO2 | ENSG00000198712 | 12.77357 | 6.236401 | 7.49E-73 | 7.61E-69 |
| EEF1A2 | ENSG00000101210 | 7.00739 | 5.058517 | 6.69E-47 | 1.94E-43 |
| MTRNR2L2 | ENSG00000271043 | 6.771883 | 4.319907 | 1.98E-42 | 4.47E-39 |
| ADCY2 | ENSG00000078295 | 6.599337 | 4.211811 | 1.26E-40 | 2.32E-37 |
| SCN9A | ENSG00000169432 | 5.636009 | 5.327962 | 1.83E-37 | 3.10E-34 |
| HES4 | ENSG00000188290 | 5.731438 | 4.309606 | 8.01E-36 | 1.25E-32 |
| EMX2 | ENSG00000170370 | 6.505939 | 2.904327 | 8.83E-34 | 1.05E-30 |
| TNR | ENSG00000116147 | 6.169591 | 3.08585 | 5.19E-33 | 5.55E-30 |
| LAMP5 | ENSG00000125869 | 5.442321 | 3.21114 | 2.63E-30 | 2.55E-27 |
| SCHIP1 | ENSG00000151967 | 5.067603 | 3.623911 | 2.40E-29 | 2.21E-26 |
| FAM171A1 | ENSG00000148468 | 4.779661 | 3.559459 | 2.77E-27 | 2.45E-24 |
| TDRD12 | ENSG00000173809 | 8.214178 | 1.682931 | 3.36E-27 | 2.84E-24 |
| GATA3 | ENSG00000107485 | 4.47962 | 4.761627 | 3.81E-27 | 3.09E-24 |
| TNFRSF18 | ENSG00000186891 | 4.872447 | 3.192023 | 1.13E-26 | 8.83E-24 |
| MTRNR2L8 | ENSG00000255823 | 8.116639 | 1.552468 | 2.07E-26 | 1.55E-23 |
| ANGPT2 | ENSG00000091879 | 5.6531 | 1.787374 | 1.18E-24 | 7.26E-22 |
| PI16 | ENSG00000164530 | 6.85776 | 1.160572 | 2.15E-24 | 1.28E-21 |
| CCNJL | ENSG00000135083 | 6.129446 | 1.464684 | 3.14E-24 | 1.77E-21 |
| HES1 | ENSG00000114315 | 5.047579 | 1.924268 | 3.68E-24 | 2.02E-21 |
| ZFP57 | ENSG00000204644 | 7.804413 | 1.451322 | 1.06E-23 | 5.53E-21 |
| NBL1 | ENSG00000158747 | 7.787555 | 1.534395 | 1.43E-23 | 7.27E-21 |
| MRPL30 | ENSG00000185414 | 7.737675 | 1.243966 | 4.15E-23 | 1.96E-20 |
| ELN | ENSG00000049540 | 5.519971 | 1.564144 | 1.66E-22 | 7.68E-20 |
| TMEM136 | ENSG00000181264 | 7.646043 | 1.416891 | 2.02E-22 | 8.71E-20 |
| RP11-691N7.6 | ENSG00000254732 | 4.470613 | 2.62667 | 2.98E-22 | 1.24E-19 |
| BEND7 | ENSG00000165626 | 7.426384 | 1.225708 | 1.23E-20 | 4.17E-18 |
| PTPRD | ENSG00000153707 | 5.048125 | 1.859366 | 1.59E-20 | 5.27E-18 |
| B4GALNT4 | ENSG00000182272 | 4.603495 | 1.563193 | 5.11E-20 | 1.60E-17 |
| CTD-2370N5.3 | ENSG00000265118 | 3.876279 | 2.225436 | 2.85E-18 | 7.23E-16 |
| CTD-2410N18.5 | ENSG00000272772 | 4.77362 | 1.223901 | 7.47E-18 | 1.78E-15 |
| MMP16 | ENSG00000156103 | 4.656208 | 1.540872 | 1.76E-17 | 3.84E-15 |
| CCDC122 | ENSG00000151773 | 4.548899 | 1.291414 | 1.76E-17 | 3.84E-15 |
| CTC-435M10.3 | ENSG00000255730 | 5.252514 | 1.011791 | 2.33E-17 | 4.98E-15 |
| TMEM150B | ENSG00000180061 | 3.829947 | 2.194124 | 2.89E-17 | 6.11E-15 |
| GOLGA8M | ENSG00000188626 | 4.270782 | 1.40709 | 4.48E-17 | 9.19E-15 |
| ZFP91-CNTF | ENSG00000255073 | 3.433618 | 3.477387 | 5.07E-17 | 1.02E-14 |
| CDH9 | ENSG00000113100 | 4.468621 | 1.203353 | 6.58E-17 | 1.30E-14 |
| AC037459.4 | ENSG00000248235 | 4.102078 | 1.493653 | 7.39E-17 | 1.43E-14 |
| IFNB1 | ENSG00000171855 | 4.160213 | 1.501838 | 1.81E-16 | 3.25E-14 |
| FAM156A | ENSG00000182646 | 3.856578 | 1.681893 | 2.38E-16 | 4.24E-14 |
| CNTN1 | ENSG00000018236 | 4.296308 | 1.174256 | 1.27E-15 | 2.04E-13 |
| LRP6 | ENSG00000070018 | 3.224507 | 3.255836 | 1.68E-15 | 2.62E-13 |
| MFAP4 | ENSG00000166482 | 4.028266 | 1.317652 | 2.31E-15 | 3.45E-13 |
| SEPT1 | ENSG00000270466 | 4.085986 | 1.286236 | 4.90E-15 | 6.82E-13 |
| AC135178.1 | ENSG00000198150 | 3.558958 | 1.910746 | 6.23E-15 | 8.43E-13 |
| GRIP1 | ENSG00000155974 | 3.788297 | 1.583958 | 8.54E-15 | 1.13E-12 |
| TMEM51 | ENSG00000171729 | 3.577349 | 1.553725 | 1.30E-14 | 1.59E-12 |
| STAG3 | ENSG00000066923 | 3.078045 | 2.981032 | 1.69E-14 | 2.02E-12 |
| GOLGA8R | ENSG00000186399 | 3.011298 | 3.51306 | 2.36E-14 | 2.72E-12 |
| ASS1 | ENSG00000130707 | 3.628166 | 1.610032 | 2.92E-14 | 3.30E-12 |
| CCDC112 | ENSG00000164221 | 3.10449 | 2.699327 | 5.58E-14 | 5.93E-12 |
| DOCK1 | ENSG00000150760 | 2.805508 | 5.911272 | 6.87E-14 | 7.16E-12 |
| IFIT2 | ENSG00000119922 | 2.781717 | 6.230065 | 9.02E-14 | 9.11E-12 |
| GPC4 | ENSG00000076716 | 2.984027 | 2.349751 | 4.85E-13 | 4.38E-11 |
| PRG3 | ENSG00000156575 | 2.752729 | 4.264364 | 6.68E-13 | 5.90E-11 |
| ZNF223 | ENSG00000267022 | 3.539571 | 1.27002 | 7.38E-13 | 6.49E-11 |
| EPX | ENSG00000121053 | 2.668056 | 5.501188 | 9.37E-13 | 8.07E-11 |
| ICA1L | ENSG00000163596 | 2.838562 | 2.65751 | 2.47E-12 | 2.00E-10 |
| CCDC144A | ENSG00000170160 | 3.244803 | 1.409126 | 2.66E-12 | 2.13E-10 |
| ZNF711 | ENSG00000147180 | 2.666559 | 4.033881 | 3.08E-12 | 2.42E-10 |
| EFCAB5 | ENSG00000176927 | 3.241655 | 1.278792 | 3.81E-12 | 2.88E-10 |
| MT-CO1 | ENSG00000198804 | 2.526491 | 13.17186 | 5.16E-12 | 3.83E-10 |
| ITGA2 | ENSG00000164171 | 2.991429 | 2.219809 | 6.27E-12 | 4.56E-10 |
| TCEAL4 | ENSG00000133142 | 2.578644 | 4.496366 | 6.79E-12 | 4.86E-10 |
| GSTM5 | ENSG00000134201 | 3.375231 | 1.251559 | 6.98E-12 | 4.94E-10 |
| ALOX15 | ENSG00000161905 | 3.010127 | 2.027342 | 9.45E-12 | 6.50E-10 |
| BEND4 | ENSG00000188848 | 2.693776 | 3.219069 | 1.01E-11 | 6.87E-10 |
| FAM213A | ENSG00000122378 | 3.236294 | 1.371392 | 1.31E-11 | 8.64E-10 |
| C19orf68 | ENSG00000185453 | 2.806544 | 2.283833 | 1.60E-11 | 1.05E-09 |
| EXPH5 | ENSG00000110723 | 3.441652 | 1.141309 | 1.81E-11 | 1.16E-09 |
| EIF3C | ENSG00000184110 | 2.502494 | 4.752074 | 1.96E-11 | 1.25E-09 |
| SCN2A | ENSG00000136531 | 3.355619 | 1.013893 | 2.05E-11 | 1.31E-09 |
| CUX2 | ENSG00000111249 | 2.998437 | 1.604251 | 2.19E-11 | 1.39E-09 |
| SLC23A1 | ENSG00000170482 | 3.01573 | 1.402503 | 3.00E-11 | 1.87E-09 |
| TNFRSF4 | ENSG00000186827 | 2.768467 | 2.061305 | 3.52E-11 | 2.14E-09 |
| SNCAIP | ENSG00000064692 | 3.191378 | 1.055071 | 4.16E-11 | 2.50E-09 |
| NDUFAF2 | ENSG00000164182 | 2.977119 | 1.447121 | 4.47E-11 | 2.66E-09 |
| TANC1 | ENSG00000115183 | 2.512796 | 3.865878 | 4.62E-11 | 2.74E-09 |
| DSG2 | ENSG00000046604 | 2.691119 | 2.562156 | 5.56E-11 | 3.22E-09 |
| MPDZ | ENSG00000107186 | 2.505634 | 3.670878 | 8.53E-11 | 4.79E-09 |
| MZB1 | ENSG00000170476 | 2.392088 | 5.253068 | 9.52E-11 | 5.28E-09 |
| IQCJ-SCHIP1 | ENSG00000250588 | 2.407586 | 4.563253 | 1.10E-10 | 5.96E-09 |
| RAB4B-EGLN2 | ENSG00000171570 | 2.698661 | 1.863638 | 1.17E-10 | 6.30E-09 |
| BEX2 | ENSG00000133134 | 2.94815 | 1.524653 | 1.21E-10 | 6.44E-09 |
| CPNE8 | ENSG00000139117 | 2.398534 | 4.71852 | 1.27E-10 | 6.72E-09 |
| TM4SF1 | ENSG00000169908 | 2.714789 | 2.321036 | 1.45E-10 | 7.62E-09 |
| POU4F1 | ENSG00000152192 | 2.361031 | 5.361281 | 1.69E-10 | 8.81E-09 |
| ASGR1 | ENSG00000141505 | 2.774034 | 1.525287 | 2.22E-10 | 1.14E-08 |
| FAM169A | ENSG00000198780 | 2.527895 | 2.518241 | 3.45E-10 | 1.73E-08 |
| XIRP1 | ENSG00000168334 | 2.734355 | 1.569438 | 3.78E-10 | 1.88E-08 |
| ZFHX3 | ENSG00000140836 | 2.28999 | 5.61795 | 4.41E-10 | 2.16E-08 |
| FOXC1 | ENSG00000054598 | 2.340474 | 3.741876 | 5.93E-10 | 2.82E-08 |
| RPS10-NUDT3 | ENSG00000270800 | 2.352297 | 3.667604 | 7.35E-10 | 3.42E-08 |
| SPAG8 | ENSG00000137098 | 2.828857 | 1.275428 | 8.49E-10 | 3.86E-08 |
| RP11-399J13.3 | ENSG00000273003 | 2.397972 | 2.699513 | 8.60E-10 | 3.89E-08 |
| EFCC1 | ENSG00000114654 | 2.909373 | 1.423162 | 9.18E-10 | 4.13E-08 |
| TRIM9 | ENSG00000100505 | 2.625102 | 1.545313 | 1.38E-09 | 5.96E-08 |
| CCDC136 | ENSG00000128596 | 2.351258 | 3.175928 | 1.55E-09 | 6.60E-08 |
| AC069368.3 | ENSG00000249240 | 2.500127 | 2.087809 | 1.67E-09 | 7.06E-08 |
| ASGR2 | ENSG00000161944 | 2.369021 | 2.533491 | 3.19E-09 | 1.28E-07 |
| BHLHE41 | ENSG00000123095 | 2.232496 | 3.601871 | 3.96E-09 | 1.56E-07 |
| FANK1 | ENSG00000203780 | 2.807774 | 1.038107 | 4.41E-09 | 1.72E-07 |
| AK5 | ENSG00000154027 | 2.381957 | 2.273049 | 5.18E-09 | 2.00E-07 |
| FAM47E-STBD1 | ENSG00000118804 | 2.459459 | 1.799329 | 6.28E-09 | 2.35E-07 |
| NPM2 | ENSG00000158806 | 2.445154 | 1.752494 | 7.09E-09 | 2.63E-07 |
| MYRF | ENSG00000124920 | 2.180933 | 3.525442 | 7.56E-09 | 2.80E-07 |
| TCEAL3 | ENSG00000196507 | 2.195681 | 3.298805 | 8.38E-09 | 3.07E-07 |
| RP11-432B6.3 | ENSG00000248710 | 2.49654 | 1.400968 | 1.03E-08 | 3.69E-07 |
| CEL | ENSG00000170835 | 2.426435 | 1.592385 | 1.14E-08 | 4.05E-07 |
| N4BP3 | ENSG00000145911 | 2.109729 | 4.277111 | 1.15E-08 | 4.08E-07 |
| BCL2L2-PABPN1 | ENSG00000258643 | 2.23739 | 2.456306 | 1.40E-08 | 4.87E-07 |
| DKFZP667F0711 | ENSG00000212743 | 2.22506 | 2.394474 | 1.62E-08 | 5.52E-07 |
| PLSCR4 | ENSG00000114698 | 2.484923 | 1.386161 | 2.41E-08 | 7.87E-07 |
| FNBP1L | ENSG00000137942 | 2.046058 | 4.873927 | 2.45E-08 | 7.95E-07 |
| DDC8 | ENSG00000178404 | 2.01035 | 6.402748 | 2.61E-08 | 8.41E-07 |
| MYOZ3 | ENSG00000164591 | 2.227617 | 1.961947 | 3.29E-08 | 1.04E-06 |
| KIAA0125 | ENSG00000226777 | 1.985039 | 6.848429 | 3.56E-08 | 1.11E-06 |
| MS4A4E | ENSG00000214787 | 2.036996 | 4.255639 | 3.66E-08 | 1.14E-06 |
| KIAA1377 | ENSG00000110318 | 2.596028 | 1.131666 | 3.85E-08 | 1.19E-06 |
| ZNF285 | ENSG00000267508 | 2.26014 | 1.903813 | 4.29E-08 | 1.31E-06 |
| EVC | ENSG00000072840 | 2.46741 | 1.203049 | 4.59E-08 | 1.38E-06 |
| SLC16A13 | ENSG00000174327 | 2.45442 | 1.070025 | 4.79E-08 | 1.44E-06 |
| PTPN14 | ENSG00000152104 | 1.985019 | 4.63074 | 6.60E-08 | 1.94E-06 |
| MAP1A | ENSG00000166963 | 1.941975 | 6.393272 | 6.91E-08 | 2.01E-06 |
| BMPR1A | ENSG00000107779 | 2.14836 | 2.30385 | 8.57E-08 | 2.45E-06 |
| EHD2 | ENSG00000024422 | 2.024783 | 3.394633 | 8.63E-08 | 2.46E-06 |
| STX16-NPEPL1 | ENSG00000254995 | 1.960804 | 4.515083 | 8.73E-08 | 2.48E-06 |
| PLXNA2 | ENSG00000076356 | 1.992708 | 3.783435 | 8.88E-08 | 2.52E-06 |
| LRRC16A | ENSG00000079691 | 2.049534 | 2.960371 | 9.98E-08 | 2.81E-06 |
| THSD7A | ENSG00000005108 | 2.032697 | 3.175822 | 1.02E-07 | 2.87E-06 |
| LA16c-431H6.6 | ENSG00000261732 | 2.025598 | 3.064936 | 1.13E-07 | 3.14E-06 |
| UPF3B | ENSG00000125351 | 1.938304 | 4.521297 | 1.21E-07 | 3.32E-06 |
| CCDC58 | ENSG00000160124 | 2.124685 | 1.899528 | 1.33E-07 | 3.62E-06 |
| PLCB4 | ENSG00000101333 | 2.197529 | 1.876835 | 1.52E-07 | 4.10E-06 |
| SCN8A | ENSG00000196876 | 2.216584 | 1.533295 | 1.83E-07 | 4.88E-06 |
| DKFZP779L1853 | ENSG00000269514 | 2.065601 | 2.264365 | 2.16E-07 | 5.73E-06 |
| SNURF | ENSG00000273173 | 1.90948 | 4.275492 | 2.26E-07 | 5.95E-06 |
| NCAM1 | ENSG00000149294 | 1.922399 | 3.70956 | 2.48E-07 | 6.39E-06 |
| PRSS21 | ENSG00000007038 | 1.917859 | 3.625421 | 2.58E-07 | 6.63E-06 |
| GPC2 | ENSG00000213420 | 1.960762 | 3.03154 | 2.81E-07 | 7.16E-06 |
| TNFSF14 | ENSG00000125735 | 2.134252 | 1.660929 | 2.97E-07 | 7.53E-06 |
| LCN2 | ENSG00000148346 | 1.943494 | 3.176749 | 3.60E-07 | 8.97E-06 |
| SLMO1 | ENSG00000141391 | 2.109319 | 1.651125 | 3.67E-07 | 9.10E-06 |
| ZNF709 | ENSG00000196826 | 1.887225 | 3.053704 | 4.32E-07 | 1.04E-05 |
| LPHN3 | ENSG00000150471 | 2.190181 | 1.829858 | 4.41E-07 | 1.06E-05 |
| COL24A1 | ENSG00000171502 | 1.79719 | 6.869351 | 5.15E-07 | 1.23E-05 |
| ABCB1 | ENSG00000085563 | 1.833677 | 4.468168 | 5.42E-07 | 1.28E-05 |
| KIAA1598 | ENSG00000187164 | 1.96871 | 2.448576 | 5.69E-07 | 1.34E-05 |
| RTP4 | ENSG00000136514 | 2.176071 | 1.230672 | 5.97E-07 | 1.39E-05 |
| TTC22 | ENSG00000006555 | 2.160161 | 1.647047 | 6.19E-07 | 1.44E-05 |
| SMAD6 | ENSG00000137834 | 2.025489 | 1.774451 | 7.47E-07 | 1.72E-05 |
| CA11 | ENSG00000063180 | 2.304164 | 1.068559 | 7.81E-07 | 1.78E-05 |
| SAP25 | ENSG00000205307 | 2.024531 | 1.909414 | 8.28E-07 | 1.88E-05 |
| GTF2IRD1 | ENSG00000006704 | 2.23154 | 1.166001 | 8.36E-07 | 1.89E-05 |
| AP000783.1 | ENSG00000254667 | 1.879225 | 3.129987 | 8.53E-07 | 1.92E-05 |
| EMC9 | ENSG00000100908 | 2.112137 | 1.35513 | 8.70E-07 | 1.96E-05 |
| CHIC1 | ENSG00000204116 | 1.837078 | 3.24534 | 9.19E-07 | 2.06E-05 |
| AR | ENSG00000169083 | 1.897703 | 3.082349 | 9.35E-07 | 2.08E-05 |
| ADAMTS2 | ENSG00000087116 | 1.925195 | 2.30955 | 9.93E-07 | 2.19E-05 |
| DOCK3 | ENSG00000088538 | 1.989975 | 2.307195 | 1.00E-06 | 2.21E-05 |
| MYEF2 | ENSG00000104177 | 1.803641 | 3.975766 | 1.11E-06 | 2.42E-05 |
| XRRA1 | ENSG00000166435 | 1.746495 | 5.504679 | 1.25E-06 | 2.71E-05 |
| MPPED2 | ENSG00000066382 | 2.091103 | 1.476642 | 1.29E-06 | 2.79E-05 |
| SAMD14 | ENSG00000167100 | 1.861647 | 2.6369 | 1.34E-06 | 2.88E-05 |
| HOOK1 | ENSG00000134709 | 2.074276 | 1.664186 | 1.35E-06 | 2.91E-05 |
| ZNF253 | ENSG00000256771 | 1.795842 | 3.507722 | 1.41E-06 | 3.02E-05 |
| MDFI | ENSG00000112559 | 1.816451 | 2.769739 | 1.44E-06 | 3.08E-05 |
| KIF16B | ENSG00000089177 | 1.763557 | 3.954228 | 1.54E-06 | 3.23E-05 |
| OLFM4 | ENSG00000102837 | 1.850452 | 2.53026 | 1.95E-06 | 4.02E-05 |
| ESF1 | ENSG00000089048 | 1.729389 | 4.430519 | 2.02E-06 | 4.16E-05 |
| IFIT3 | ENSG00000119917 | 1.721405 | 4.765085 | 2.10E-06 | 4.31E-05 |
| IFNLR1 | ENSG00000185436 | 2.03071 | 1.212664 | 2.31E-06 | 4.72E-05 |
| SPNS3 | ENSG00000182557 | 1.686195 | 6.375946 | 2.40E-06 | 4.83E-05 |
| AL591479.1 | ENSG00000269337 | 1.870908 | 2.381402 | 2.43E-06 | 4.87E-05 |
| ZNF658 | ENSG00000196409 | 2.065418 | 1.136082 | 2.47E-06 | 4.94E-05 |
| ANKRD33B | ENSG00000164236 | 1.693245 | 5.105564 | 2.67E-06 | 5.29E-05 |
| PNPLA1 | ENSG00000180316 | 2.058869 | 1.202746 | 2.69E-06 | 5.32E-05 |
| HCN3 | ENSG00000143630 | 1.926402 | 1.8225 | 2.77E-06 | 5.44E-05 |
| PRKCZ | ENSG00000067606 | 1.80709 | 2.693249 | 2.80E-06 | 5.47E-05 |
| CACNA1C | ENSG00000151067 | 1.798215 | 2.95193 | 2.87E-06 | 5.59E-05 |
| ZNF154 | ENSG00000179909 | 1.714489 | 3.929757 | 3.20E-06 | 6.20E-05 |
| LENG1 | ENSG00000105617 | 1.784116 | 2.784204 | 3.35E-06 | 6.45E-05 |
| SLC4A8 | ENSG00000050438 | 1.730218 | 3.270191 | 3.39E-06 | 6.52E-05 |
| LEF1 | ENSG00000138795 | 1.753687 | 3.167737 | 3.49E-06 | 6.68E-05 |
| RND1 | ENSG00000172602 | 1.771841 | 2.624815 | 3.56E-06 | 6.81E-05 |
| DERL3 | ENSG00000099958 | 1.737996 | 3.058393 | 3.57E-06 | 6.83E-05 |
| TAP2 | ENSG00000250264 | 2.017055 | 1.330712 | 3.60E-06 | 6.86E-05 |
| RAB33A | ENSG00000134594 | 1.964522 | 1.324858 | 3.60E-06 | 6.86E-05 |
| HOXA7 | ENSG00000122592 | 1.693141 | 4.007864 | 3.65E-06 | 6.94E-05 |
| SYNJ2BP | ENSG00000213463 | 1.73957 | 2.98933 | 4.18E-06 | 7.81E-05 |
| CD52 | ENSG00000169442 | 1.658343 | 4.995285 | 4.27E-06 | 7.97E-05 |
| ZNF823 | ENSG00000197933 | 1.808266 | 2.252981 | 4.46E-06 | 8.29E-05 |
| TMEM220 | ENSG00000187824 | 1.71813 | 2.972911 | 4.81E-06 | 8.86E-05 |
| PLA2G4B | ENSG00000243708 | 1.679619 | 3.656989 | 4.87E-06 | 8.95E-05 |
| RBPMS | ENSG00000157110 | 1.670265 | 4.47392 | 4.87E-06 | 8.95E-05 |
| CDR2L | ENSG00000109089 | 2.012375 | 1.207759 | 5.33E-06 | 9.70E-05 |
| ARMC9 | ENSG00000135931 | 1.743313 | 2.484633 | 5.44E-06 | 9.89E-05 |
| GPR126 | ENSG00000112414 | 1.650717 | 4.420561 | 5.87E-06 | 0.000106 |
| GOLIM4 | ENSG00000173905 | 1.621238 | 5.634112 | 5.89E-06 | 0.000106 |
| ZRSR2 | ENSG00000169249 | 1.656908 | 3.855967 | 5.97E-06 | 0.000108 |
| CCDC102B | ENSG00000150636 | 1.782964 | 2.414673 | 5.98E-06 | 0.000108 |
| RPGRIP1L | ENSG00000103494 | 1.734723 | 2.517529 | 6.07E-06 | 0.000109 |
| RTN4R | ENSG00000040608 | 1.823396 | 1.61048 | 6.43E-06 | 0.000114 |
| TM9SF1 | ENSG00000254692 | 2.055261 | 1.461327 | 6.58E-06 | 0.000116 |
| RPL36AL | ENSG00000165502 | 1.608467 | 5.931538 | 6.64E-06 | 0.000117 |
| SPDYA | ENSG00000163806 | 1.662048 | 3.476554 | 7.00E-06 | 0.000123 |
| SH3BP5 | ENSG00000131370 | 1.610181 | 5.171841 | 7.43E-06 | 0.00013 |
| CNNM1 | ENSG00000119946 | 1.920597 | 1.506519 | 7.53E-06 | 0.000131 |
| NEK10 | ENSG00000163491 | 1.758849 | 1.794734 | 8.09E-06 | 0.00014 |
| ZEB1 | ENSG00000148516 | 1.596337 | 5.621107 | 8.21E-06 | 0.000142 |
| EPPK1 | ENSG00000227184 | 1.755918 | 2.167866 | 8.82E-06 | 0.000151 |
| CCDC30 | ENSG00000186409 | 1.633841 | 3.773159 | 9.06E-06 | 0.000155 |
| PAQR8 | ENSG00000170915 | 1.627139 | 3.766264 | 9.26E-06 | 0.000158 |
| TCHH | ENSG00000159450 | 1.759104 | 2.394771 | 1.02E-05 | 0.00017 |
| PHKA1 | ENSG00000067177 | 1.729243 | 2.496062 | 1.03E-05 | 0.000172 |
| SYCP2L | ENSG00000153157 | 1.908403 | 1.508036 | 1.04E-05 | 0.000173 |
| TCEA2 | ENSG00000171703 | 1.625077 | 3.400293 | 1.05E-05 | 0.000175 |
| IRX5 | ENSG00000176842 | 1.678693 | 2.530934 | 1.10E-05 | 0.000182 |
| ZNF521 | ENSG00000198795 | 1.602662 | 4.527788 | 1.14E-05 | 0.000188 |
| MAP7 | ENSG00000135525 | 1.558444 | 5.963153 | 1.23E-05 | 0.000201 |
| INO80B | ENSG00000115274 | 1.579581 | 4.623422 | 1.26E-05 | 0.000207 |
| PEX11B | ENSG00000131779 | 1.63864 | 3.065316 | 1.26E-05 | 0.000207 |
| MRPS14 | ENSG00000120333 | 1.627664 | 3.148993 | 1.27E-05 | 0.000207 |
| PRG2 | ENSG00000186652 | 1.553011 | 6.69969 | 1.29E-05 | 0.00021 |
| MREG | ENSG00000118242 | 1.854505 | 1.318442 | 1.29E-05 | 0.00021 |
| WDR35 | ENSG00000118965 | 1.582256 | 4.395127 | 1.30E-05 | 0.000211 |
| CTD-2116N17.1 | ENSG00000259316 | 1.816654 | 1.63917 | 1.37E-05 | 0.000222 |
| DTHD1 | ENSG00000197057 | 1.765998 | 2.005769 | 1.39E-05 | 0.000224 |
| AVPI1 | ENSG00000119986 | 1.72953 | 1.869826 | 1.49E-05 | 0.00024 |
| RP11-159D12.5 | ENSG00000266086 | 1.713467 | 2.032484 | 1.53E-05 | 0.000245 |
| KIF21A | ENSG00000139116 | 1.713297 | 1.993951 | 1.63E-05 | 0.000258 |
| APBA2 | ENSG00000034053 | 1.591908 | 3.527814 | 1.63E-05 | 0.000258 |
| KIAA1217 | ENSG00000120549 | 1.614351 | 3.177104 | 1.77E-05 | 0.000279 |
| RP11-872D17.8 | ENSG00000254979 | 1.734611 | 1.553463 | 1.83E-05 | 0.000288 |
| TERT | ENSG00000164362 | 1.932376 | 1.108538 | 1.84E-05 | 0.000289 |
| PFDN4 | ENSG00000101132 | 1.578839 | 3.461643 | 1.91E-05 | 0.000299 |
| EPHA1 | ENSG00000146904 | 1.705441 | 1.954204 | 1.93E-05 | 0.000301 |
| IRX3 | ENSG00000177508 | 1.571754 | 3.874405 | 1.93E-05 | 0.000301 |
| SLC37A3 | ENSG00000157800 | 1.713899 | 2.145988 | 1.99E-05 | 0.000311 |
| EIF3CL | ENSG00000205609 | 1.589953 | 3.407586 | 2.06E-05 | 0.00032 |
| IFT74 | ENSG00000096872 | 1.559565 | 3.889478 | 2.07E-05 | 0.000321 |
| AIF1L | ENSG00000126878 | 1.579614 | 3.576789 | 2.10E-05 | 0.000325 |
| CLMN | ENSG00000165959 | 1.526319 | 4.766692 | 2.23E-05 | 0.000341 |
| PPP2R2B | ENSG00000156475 | 1.690259 | 1.75894 | 2.31E-05 | 0.000352 |
| VWDE | ENSG00000146530 | 1.799721 | 1.866474 | 2.36E-05 | 0.000359 |
| SULT1C4 | ENSG00000198075 | 1.692573 | 2.175695 | 2.39E-05 | 0.000363 |
| LILRB4 | ENSG00000186818 | 1.568699 | 3.36732 | 2.57E-05 | 0.000386 |
| CEP290 | ENSG00000198707 | 1.494828 | 5.605114 | 2.76E-05 | 0.000409 |
| PROCA1 | ENSG00000167525 | 1.560295 | 3.06362 | 2.77E-05 | 0.000411 |
| AC026703.1 | ENSG00000181495 | 1.512319 | 4.614732 | 2.79E-05 | 0.000413 |
| RP11-268J15.5 | ENSG00000116883 | 1.539188 | 3.555486 | 2.84E-05 | 0.00042 |
| NBPF24 | ENSG00000203836 | 1.816344 | 1.22027 | 2.89E-05 | 0.000427 |
| POLR3G | ENSG00000113356 | 1.622331 | 2.301993 | 3.18E-05 | 0.000464 |
| F2RL1 | ENSG00000164251 | 1.708227 | 2.068897 | 3.18E-05 | 0.000464 |
| SCRN1 | ENSG00000136193 | 1.497786 | 5.039102 | 3.20E-05 | 0.000466 |
| IGSF10 | ENSG00000152580 | 1.479749 | 5.563995 | 3.38E-05 | 0.000491 |
| SPTLC3 | ENSG00000172296 | 1.530123 | 3.346798 | 3.39E-05 | 0.000493 |
| ITPKA | ENSG00000137825 | 1.65316 | 1.783769 | 3.50E-05 | 0.000507 |
| PRODH | ENSG00000100033 | 1.497449 | 4.558106 | 3.51E-05 | 0.000507 |
| KIF7 | ENSG00000166813 | 1.570846 | 2.984993 | 3.56E-05 | 0.000515 |
| KIFC3 | ENSG00000140859 | 1.498413 | 4.121246 | 3.86E-05 | 0.00055 |
| MAGI3 | ENSG00000081026 | 1.539132 | 3.456608 | 3.88E-05 | 0.000553 |
| TMEM176A | ENSG00000002933 | 1.636962 | 1.864535 | 4.00E-05 | 0.000567 |
| HLF | ENSG00000108924 | 1.63712 | 1.893877 | 4.09E-05 | 0.000579 |
| DACH1 | ENSG00000165659 | 1.494952 | 4.231189 | 4.16E-05 | 0.000587 |
| SERPINI2 | ENSG00000114204 | 1.784974 | 1.213307 | 4.20E-05 | 0.000591 |
| AP006621.5 | ENSG00000255284 | 1.685895 | 1.617732 | 4.33E-05 | 0.000608 |
| BEND6 | ENSG00000151917 | 1.777707 | 1.067674 | 4.42E-05 | 0.000614 |
| KCNJ15 | ENSG00000157551 | 1.782895 | 1.414773 | 4.53E-05 | 0.000625 |
| LPAR1 | ENSG00000198121 | 1.56377 | 2.619931 | 4.63E-05 | 0.000637 |
| IGF2BP2 | ENSG00000073792 | 1.439992 | 7.0922 | 4.77E-05 | 0.000655 |
| MYCN | ENSG00000134323 | 1.443301 | 5.637664 | 4.99E-05 | 0.000682 |
| C15orf38-AP3S2 | ENSG00000250021 | 1.562903 | 2.119885 | 5.06E-05 | 0.00069 |
| CCDC121 | ENSG00000176714 | 1.642765 | 1.575278 | 5.32E-05 | 0.000723 |
| DDTL | ENSG00000099974 | 1.539904 | 2.594346 | 5.35E-05 | 0.000725 |
| SLC25A53 | ENSG00000176274 | 1.582303 | 2.199499 | 5.38E-05 | 0.000729 |
| ZNF334 | ENSG00000198185 | 1.550678 | 2.680592 | 5.44E-05 | 0.000732 |
| GOLGA8O | ENSG00000206127 | 1.479667 | 3.716974 | 5.45E-05 | 0.000734 |
| STARD9 | ENSG00000159433 | 1.430243 | 6.641523 | 5.53E-05 | 0.000743 |
| CXCL3 | ENSG00000163734 | 1.482565 | 3.74545 | 5.56E-05 | 0.000747 |
| FRMD4B | ENSG00000114541 | 1.442956 | 5.14319 | 5.60E-05 | 0.00075 |
| SEMA3C | ENSG00000075223 | 1.689742 | 1.728674 | 5.66E-05 | 0.000758 |
| DSC2 | ENSG00000134755 | 1.455844 | 4.606558 | 5.73E-05 | 0.000763 |
| CALN1 | ENSG00000183166 | 1.473987 | 4.069819 | 5.97E-05 | 0.00079 |
| C9orf139 | ENSG00000180539 | 1.448169 | 4.275246 | 5.98E-05 | 0.000792 |
| TLR9 | ENSG00000239732 | 1.59742 | 2.033764 | 6.44E-05 | 0.000845 |
| ACP6 | ENSG00000162836 | 1.48055 | 3.391801 | 6.67E-05 | 0.000873 |
| PLEC | ENSG00000178209 | 1.406471 | 9.984394 | 6.73E-05 | 0.00088 |
| TIFAB | ENSG00000255833 | 1.494195 | 3.095705 | 6.79E-05 | 0.000886 |
| SLCO5A1 | ENSG00000137571 | 1.487737 | 3.469525 | 6.82E-05 | 0.00089 |
| RAG1 | ENSG00000166349 | 1.700562 | 1.238177 | 7.08E-05 | 0.000922 |
| ZNF626 | ENSG00000188171 | 1.623366 | 2.021679 | 7.56E-05 | 0.000966 |
| LUC7L3 | ENSG00000108848 | 1.396441 | 8.603048 | 7.62E-05 | 0.000972 |
| C1orf122 | ENSG00000197982 | 1.489733 | 2.814919 | 7.87E-05 | 0.001 |
| MTMR11 | ENSG00000014914 | 1.658489 | 1.530689 | 7.94E-05 | 0.001009 |
| ZNF594 | ENSG00000180626 | 1.46275 | 3.491831 | 8.26E-05 | 0.001043 |
| DNM1 | ENSG00000106976 | 1.406861 | 5.154485 | 8.60E-05 | 0.001083 |
| GPR162 | ENSG00000250510 | 1.55648 | 2.076573 | 8.79E-05 | 0.001105 |
| TNFRSF10C | ENSG00000173535 | 1.454908 | 3.290993 | 9.27E-05 | 0.001163 |
| PPFIA3 | ENSG00000177380 | 1.495361 | 2.44528 | 9.28E-05 | 0.001164 |
| GPR135 | ENSG00000181619 | 1.431451 | 3.718837 | 9.75E-05 | 0.001217 |
| ZC3H13 | ENSG00000123200 | 1.377362 | 7.146841 | 9.80E-05 | 0.001221 |
| KLF9 | ENSG00000119138 | 1.387202 | 5.552934 | 9.80E-05 | 0.001221 |
| AC027763.2 | ENSG00000215067 | 1.461306 | 2.776096 | 0.000107 | 0.001324 |
| PDZD2 | ENSG00000133401 | 1.470347 | 2.653771 | 0.000108 | 0.001336 |
| KIF3A | ENSG00000131437 | 1.418032 | 3.34051 | 0.00011 | 0.001356 |
| GOLGA8H | ENSG00000261794 | 1.47009 | 2.56212 | 0.000112 | 0.00138 |
| WDR60 | ENSG00000126870 | 1.377815 | 5.237768 | 0.000112 | 0.00138 |
| CCDC104 | ENSG00000163001 | 1.414153 | 3.601682 | 0.000116 | 0.001415 |
| MPHOSPH8 | ENSG00000196199 | 1.364354 | 6.369073 | 0.000117 | 0.001436 |
| CCDC66 | ENSG00000180376 | 1.379688 | 4.730032 | 0.000118 | 0.001448 |
| FAM133B | ENSG00000234545 | 1.369891 | 5.278328 | 0.000119 | 0.001457 |
| TIGD7 | ENSG00000140993 | 1.459075 | 2.569912 | 0.000123 | 0.001498 |
| CYSLTR2 | ENSG00000152207 | 1.531635 | 1.900326 | 0.000124 | 0.001503 |
| MT-ND2 | ENSG00000198763 | 1.350708 | 15.18945 | 0.000126 | 0.001508 |
| CWC27 | ENSG00000153015 | 1.376244 | 4.498483 | 0.000128 | 0.001534 |
| TMEM79 | ENSG00000163472 | 1.554194 | 1.748181 | 0.000128 | 0.001534 |
| CSRP2 | ENSG00000175183 | 1.473118 | 2.241487 | 0.000132 | 0.001572 |
| ZNF502 | ENSG00000196653 | 1.537539 | 2.204293 | 0.000132 | 0.00158 |
| YY2 | ENSG00000230797 | 1.746527 | 1.013657 | 0.000134 | 0.001603 |
| ZNF138 | ENSG00000197008 | 1.411871 | 3.30115 | 0.000135 | 0.001614 |
| GPRASP1 | ENSG00000198932 | 1.370218 | 4.34708 | 0.000151 | 0.00178 |
| AMN1 | ENSG00000151743 | 1.541006 | 1.760852 | 0.000161 | 0.001887 |
| GPR56 | ENSG00000205336 | 1.33632 | 5.905008 | 0.000163 | 0.001916 |
| PNISR | ENSG00000132424 | 1.326619 | 8.571397 | 0.000167 | 0.001953 |
| MT-ATP6 | ENSG00000198899 | 1.325642 | 8.419117 | 0.000171 | 0.002004 |
| RPL26L1 | ENSG00000037241 | 1.508177 | 1.663405 | 0.000181 | 0.002102 |
| CACNB4 | ENSG00000182389 | 1.397852 | 3.181132 | 0.000182 | 0.00212 |
| MMP2 | ENSG00000087245 | 1.334734 | 5.240565 | 0.000184 | 0.002131 |
| ARMC2 | ENSG00000118690 | 1.457755 | 2.247269 | 0.000184 | 0.00214 |
| RPS24 | ENSG00000138326 | 1.316502 | 8.995773 | 0.000186 | 0.002149 |
| AK9 | ENSG00000155085 | 1.351907 | 4.133334 | 0.000187 | 0.002159 |
| TLR10 | ENSG00000174123 | 1.512834 | 1.686626 | 0.000188 | 0.002172 |
| CACNA1F | ENSG00000102001 | 1.455666 | 2.240592 | 0.000195 | 0.002239 |
| PIPOX | ENSG00000179761 | 1.424798 | 2.232368 | 0.000195 | 0.002239 |
| CCDC23 | ENSG00000177868 | 1.437738 | 2.04278 | 0.000196 | 0.002248 |
| LCT | ENSG00000115850 | 1.543486 | 1.790372 | 0.000197 | 0.002252 |
| SLITRK4 | ENSG00000179542 | 1.358937 | 3.724124 | 0.000206 | 0.002358 |
| MPP6 | ENSG00000105926 | 1.34878 | 3.892533 | 0.000222 | 0.002489 |
| CCDC7 | ENSG00000216937 | 1.347347 | 3.510332 | 0.000224 | 0.002506 |
| RP11-366L20.2 | ENSG00000197301 | 1.580496 | 1.437726 | 0.000225 | 0.002513 |
| RAC3 | ENSG00000169750 | 1.468733 | 1.914057 | 0.000226 | 0.002519 |
| RCN3 | ENSG00000142552 | 1.385835 | 2.983712 | 0.00023 | 0.002557 |
| AASS | ENSG00000008311 | 1.484354 | 2.308856 | 0.000232 | 0.002583 |
| ZNF793 | ENSG00000188227 | 1.354956 | 3.653225 | 0.000236 | 0.002617 |
| NOV | ENSG00000136999 | 1.561517 | 1.33837 | 0.000237 | 0.002629 |
| ANKRD16 | ENSG00000134461 | 1.365422 | 2.917764 | 0.000241 | 0.00267 |
| ZC4H2 | ENSG00000126970 | 1.427831 | 2.180239 | 0.000242 | 0.002678 |
| PTGDS | ENSG00000107317 | 1.41733 | 2.066311 | 0.000244 | 0.002703 |
| TTLL1 | ENSG00000100271 | 1.397051 | 2.354518 | 0.000247 | 0.002735 |
| HMGN3 | ENSG00000118418 | 1.312312 | 4.749334 | 0.000247 | 0.002735 |
| CYP4V2 | ENSG00000145476 | 1.307645 | 4.905998 | 0.000254 | 0.002801 |
| MYO5C | ENSG00000128833 | 1.315709 | 4.759939 | 0.000256 | 0.002829 |
| SLC35G2 | ENSG00000168917 | 1.343052 | 3.016829 | 0.000268 | 0.002945 |
| HIRIP3 | ENSG00000149929 | 1.324903 | 3.967512 | 0.000269 | 0.002956 |
| NPDC1 | ENSG00000107281 | 1.304217 | 5.071408 | 0.000276 | 0.003035 |
| CDCP1 | ENSG00000163814 | 1.353013 | 3.135007 | 0.000278 | 0.003053 |
| NEURL1B | ENSG00000214357 | 1.325059 | 3.740125 | 0.000296 | 0.00323 |
| PODXL | ENSG00000128567 | 1.377662 | 2.711987 | 0.0003 | 0.003275 |
| SLX1B | ENSG00000181625 | 1.49891 | 1.630659 | 0.000303 | 0.003301 |
| SNRPN | ENSG00000128739 | 1.310943 | 3.437429 | 0.000348 | 0.003753 |
| ZNF852 | ENSG00000178917 | 1.282586 | 4.481241 | 0.000349 | 0.003762 |
| CORO6 | ENSG00000167549 | 1.422553 | 1.887847 | 0.00035 | 0.00377 |
| MEA1 | ENSG00000124733 | 1.289931 | 4.166926 | 0.000356 | 0.003828 |
| PSMC3IP | ENSG00000131470 | 1.391064 | 1.850107 | 0.000371 | 0.003919 |
| ING2 | ENSG00000168556 | 1.296432 | 3.724951 | 0.000376 | 0.003962 |
| ZMAT1 | ENSG00000166432 | 1.257185 | 5.919602 | 0.000386 | 0.004055 |
| DCTN6 | ENSG00000104671 | 1.289264 | 3.727532 | 0.000405 | 0.004236 |
| ZC3H8 | ENSG00000144161 | 1.301721 | 3.418182 | 0.000414 | 0.004321 |
| ZNF85 | ENSG00000105750 | 1.317347 | 2.862906 | 0.000435 | 0.004513 |
| C1QTNF6 | ENSG00000133466 | 1.309042 | 3.130597 | 0.000443 | 0.004574 |
| MACROD1 | ENSG00000133315 | 1.330884 | 2.646099 | 0.000445 | 0.004593 |
| FZD7 | ENSG00000155760 | 1.41864 | 1.815536 | 0.00045 | 0.00464 |
| CA8 | ENSG00000178538 | 1.349057 | 2.410704 | 0.00045 | 0.00464 |
| RABL2A | ENSG00000144134 | 1.273953 | 3.875279 | 0.000458 | 0.004702 |
| CLIC2 | ENSG00000155962 | 1.304035 | 3.292701 | 0.000461 | 0.004724 |
| AL031663.2 | ENSG00000269549 | 1.436137 | 1.479367 | 0.00047 | 0.004809 |
| ZNF665 | ENSG00000197497 | 1.335649 | 2.498583 | 0.000471 | 0.004818 |
| CFDP1 | ENSG00000153774 | 1.248815 | 4.775343 | 0.000482 | 0.004932 |
| ZNF577 | ENSG00000161551 | 1.248815 | 4.591852 | 0.000489 | 0.004985 |
| P2RX7 | ENSG00000089041 | 1.25129 | 4.305955 | 0.000491 | 0.004999 |
| CARD11 | ENSG00000198286 | 1.24174 | 5.071989 | 0.000501 | 0.005092 |
| RP9 | ENSG00000164610 | 1.307855 | 2.651628 | 0.000502 | 0.005095 |
| ARL17B | ENSG00000228696 | 1.411647 | 1.852337 | 0.000505 | 0.005125 |
| ERCC5 | ENSG00000134899 | 1.229123 | 5.87778 | 0.000508 | 0.005151 |
| CDH4 | ENSG00000179242 | 1.647385 | 1.111466 | 0.000509 | 0.005156 |
| CCAR1 | ENSG00000060339 | 1.22372 | 6.728128 | 0.000518 | 0.005238 |
| GPATCH4 | ENSG00000160818 | 1.244928 | 4.630721 | 0.000523 | 0.005288 |
| GUCY1A3 | ENSG00000164116 | 1.22298 | 6.475272 | 0.000528 | 0.005332 |
| ZNF354C | ENSG00000177932 | 1.388107 | 2.268207 | 0.000552 | 0.005543 |
| NFATC1 | ENSG00000131196 | 1.22359 | 5.602239 | 0.000553 | 0.005548 |
| GALNT12 | ENSG00000119514 | 1.412853 | 1.698537 | 0.000553 | 0.005549 |
| BCAT1 | ENSG00000060982 | 1.221789 | 5.866346 | 0.000561 | 0.00562 |
| TTC24 | ENSG00000187862 | 1.265216 | 3.801226 | 0.00059 | 0.005881 |
| TNIK | ENSG00000154310 | 1.21965 | 5.279649 | 0.000592 | 0.005902 |
| GKAP1 | ENSG00000165113 | 1.28454 | 2.718722 | 0.000596 | 0.005931 |
| L3MBTL3 | ENSG00000198945 | 1.232881 | 4.238627 | 0.000613 | 0.006083 |
| MITD1 | ENSG00000158411 | 1.268837 | 3.194087 | 0.000616 | 0.006113 |
| CCDC92 | ENSG00000119242 | 1.245922 | 3.706447 | 0.000621 | 0.006159 |
| SC5D | ENSG00000109929 | 1.254079 | 3.498324 | 0.000624 | 0.00618 |
| BIVM-ERCC5 | ENSG00000270181 | 1.208896 | 5.719743 | 0.000641 | 0.00632 |
| H2AFY2 | ENSG00000099284 | 1.298335 | 2.402361 | 0.000642 | 0.00632 |
| GAL3ST4 | ENSG00000197093 | 1.326685 | 2.083684 | 0.000708 | 0.00687 |
| CROCC | ENSG00000058453 | 1.203905 | 5.030728 | 0.00072 | 0.006972 |
| ZNF667 | ENSG00000198046 | 1.209784 | 4.707955 | 0.000732 | 0.007074 |
| C9orf9 | ENSG00000165698 | 1.340351 | 1.812669 | 0.000739 | 0.007136 |
| GRHL1 | ENSG00000134317 | 1.435624 | 1.22609 | 0.000739 | 0.007136 |
| ZNF625 | ENSG00000257591 | 1.360307 | 1.890976 | 0.000747 | 0.0072 |
| OASL | ENSG00000135114 | 1.243039 | 3.169576 | 0.000752 | 0.007244 |
| XIRP2 | ENSG00000163092 | 1.423164 | 1.60729 | 0.000752 | 0.007244 |
| OBSCN | ENSG00000154358 | 1.183179 | 7.599975 | 0.000769 | 0.007392 |
| PNN | ENSG00000100941 | 1.181078 | 8.648376 | 0.000771 | 0.00741 |
| MYO1E | ENSG00000157483 | 1.237626 | 3.187858 | 0.000803 | 0.007685 |
| SPSB1 | ENSG00000171621 | 1.365162 | 1.752356 | 0.000815 | 0.007787 |
| GTF2E1 | ENSG00000153767 | 1.217074 | 3.664652 | 0.000819 | 0.00781 |
| ANP32B | ENSG00000136938 | 1.175093 | 8.195828 | 0.000823 | 0.007846 |
| CCDC62 | ENSG00000130783 | 1.250809 | 2.654254 | 0.000832 | 0.007918 |
| HOXA6 | ENSG00000106006 | 1.190946 | 4.528697 | 0.000832 | 0.007918 |
| CLEC10A | ENSG00000132514 | 1.39253 | 1.516795 | 0.000837 | 0.007965 |
| SMO | ENSG00000128602 | 1.289428 | 2.245925 | 0.000858 | 0.008153 |
| ZNF783 | ENSG00000204946 | 1.178553 | 5.673595 | 0.000861 | 0.008173 |
| C1RL | ENSG00000139178 | 1.17851 | 5.554224 | 0.00088 | 0.008338 |
| SLC52A3 | ENSG00000101276 | 1.402964 | 1.125794 | 0.000883 | 0.008365 |
| BIN1 | ENSG00000136717 | 1.184091 | 4.720191 | 0.000907 | 0.008568 |
| C20orf96 | ENSG00000196476 | 1.182907 | 4.682711 | 0.000913 | 0.008616 |
| EVI2B | ENSG00000185862 | 1.167288 | 6.69127 | 0.000921 | 0.008673 |
| GBP2 | ENSG00000162645 | 1.173935 | 5.416048 | 0.000925 | 0.008703 |
| PIEZO2 | ENSG00000154864 | 1.181302 | 4.126163 | 0.000932 | 0.008767 |
| TRAF3IP3 | ENSG00000009790 | 1.16683 | 6.419045 | 0.000936 | 0.008793 |
| AP003068.23 | ENSG00000254614 | 1.453369 | 1.140127 | 0.000945 | 0.008852 |
| TYSND1 | ENSG00000156521 | 1.202548 | 3.6719 | 0.000952 | 0.008909 |
| ETFB | ENSG00000105379 | 1.179519 | 4.580021 | 0.000955 | 0.008929 |
| NSRP1 | ENSG00000126653 | 1.177062 | 4.840195 | 0.000963 | 0.009001 |
| IL17RE | ENSG00000163701 | 1.227395 | 2.87586 | 0.000991 | 0.009232 |
| TMEM254 | ENSG00000133678 | 1.228854 | 2.934285 | 0.000998 | 0.009281 |
| GLIPR1 | ENSG00000139278 | 1.157414 | 6.996423 | 0.001001 | 0.009301 |
| RP4-583P15.15 | ENSG00000273154 | 1.22495 | 2.595348 | 0.001043 | 0.009621 |
| NSA2 | ENSG00000164346 | 1.163002 | 5.196304 | 0.001049 | 0.009677 |
| ZNF891 | ENSG00000214029 | 1.197566 | 3.370526 | 0.00107 | 0.009843 |
| ROBO3 | ENSG00000154134 | 1.181964 | 4.445289 | 0.001089 | 0.009976 |
| GRAMD1C | ENSG00000178075 | 1.336124 | 1.613036 | 0.001089 | 0.009976 |
| MALRD1 | ENSG00000204740 | 1.283081 | 1.945795 | 0.001095 | 0.009976 |
| PYHIN1 | ENSG00000163564 | 1.182705 | 3.887694 | 0.001102 | 0.009987 |
| CCDC183 | ENSG00000213213 | 1.326579 | 1.818645 | 0.001111 | 0.010063 |
| CABLES1 | ENSG00000134508 | 1.176777 | 4.205443 | 0.001118 | 0.010111 |
| MBLAC1 | ENSG00000214309 | 1.345761 | 1.258903 | 0.00114 | 0.010287 |
| PTP4A3 | ENSG00000184489 | 1.186013 | 3.703008 | 0.00115 | 0.010371 |
| NKD1 | ENSG00000140807 | 1.281771 | 1.913863 | 0.001152 | 0.010381 |
| DST | ENSG00000151914 | 1.145174 | 6.585943 | 0.001156 | 0.010406 |
| BICD1 | ENSG00000151746 | 1.154734 | 5.081208 | 0.00116 | 0.010429 |
| ZNF260 | ENSG00000254004 | 1.1681 | 4.125099 | 0.001164 | 0.010457 |
| CORO7-PAM16 | ENSG00000103426 | 1.241144 | 2.536172 | 0.001211 | 0.010837 |
| KRCC1 | ENSG00000172086 | 1.195598 | 3.234569 | 0.001265 | 0.011282 |
| SLTM | ENSG00000137776 | 1.129448 | 7.640476 | 0.001301 | 0.011566 |
| ARID4A | ENSG00000032219 | 1.128671 | 7.036455 | 0.00132 | 0.011713 |
| PPAN | ENSG00000130810 | 1.131328 | 6.0556 | 0.001343 | 0.011908 |
| NEURL2 | ENSG00000124257 | 1.339337 | 1.426233 | 0.001357 | 0.01201 |
| CDC26 | ENSG00000176386 | 1.183878 | 3.191319 | 0.001367 | 0.012089 |
| EID3 | ENSG00000255150 | 1.130936 | 5.316647 | 0.001396 | 0.012322 |
| DCP1B | ENSG00000151065 | 1.182092 | 3.081145 | 0.001407 | 0.01241 |
| WDR17 | ENSG00000150627 | 1.300571 | 1.664935 | 0.001425 | 0.012552 |
| OSGEPL1 | ENSG00000128694 | 1.249527 | 1.939314 | 0.001457 | 0.012807 |
| TRPV1 | ENSG00000196689 | 1.168583 | 3.18653 | 0.00149 | 0.013066 |
| HIST2H4A | ENSG00000183941 | 1.273021 | 1.926697 | 0.001508 | 0.013174 |
| ZNF580 | ENSG00000213015 | 1.128001 | 5.02962 | 0.001512 | 0.013205 |
| RIC3 | ENSG00000166405 | 1.276544 | 1.823507 | 0.001555 | 0.013529 |
| NEIL2 | ENSG00000154328 | 1.17979 | 2.979797 | 0.001557 | 0.013537 |
| SAMD15 | ENSG00000100583 | 1.275573 | 1.6382 | 0.001567 | 0.013618 |
| MTIF3 | ENSG00000122033 | 1.132722 | 4.061145 | 0.001609 | 0.013925 |
| HOOK2 | ENSG00000095066 | 1.131179 | 4.304231 | 0.001623 | 0.014034 |
| KIAA1549 | ENSG00000122778 | 1.130204 | 4.41311 | 0.001633 | 0.01408 |
| RPS11 | ENSG00000142534 | 1.103436 | 9.049783 | 0.001643 | 0.014163 |
| TSPYL5 | ENSG00000180543 | 1.17909 | 2.876522 | 0.001702 | 0.014619 |
| RP11-111M22.2 | ENSG00000179240 | 1.152494 | 3.240375 | 0.001726 | 0.014814 |
| RAP1GAP | ENSG00000076864 | 1.217347 | 2.381136 | 0.001734 | 0.014875 |
| RBM34 | ENSG00000188739 | 1.108143 | 5.266197 | 0.001758 | 0.015075 |
| ASPHD2 | ENSG00000128203 | 1.369893 | 1.131736 | 0.001786 | 0.015281 |
| DKFZP434E1119 | ENSG00000268635 | 1.368463 | 1.229337 | 0.001786 | 0.015281 |
| BOLA1 | ENSG00000178096 | 1.167956 | 2.62092 | 0.001806 | 0.015405 |
| SMIM10 | ENSG00000184785 | 1.330373 | 1.495494 | 0.001831 | 0.015611 |
| NUFIP1 | ENSG00000083635 | 1.124413 | 3.714955 | 0.001835 | 0.015639 |
| RPL36A | ENSG00000241343 | 1.092395 | 8.268777 | 0.001837 | 0.01565 |
| NEDD8-MDP1 | ENSG00000255526 | 1.286409 | 1.488336 | 0.00184 | 0.015665 |
| PDXP | ENSG00000241360 | 1.256624 | 1.790617 | 0.001844 | 0.01569 |
| RAB39B | ENSG00000155961 | 1.279502 | 1.574071 | 0.001926 | 0.01612 |
| RAB43 | ENSG00000172780 | 1.207647 | 2.05396 | 0.001959 | 0.016276 |
| SLC9A7 | ENSG00000065923 | 1.093086 | 5.538988 | 0.001965 | 0.016311 |
| ARG2 | ENSG00000081181 | 1.298806 | 1.35923 | 0.001972 | 0.016361 |
| CLHC1 | ENSG00000162994 | 1.188301 | 2.342032 | 0.001975 | 0.016375 |
| HNRNPA1L2 | ENSG00000139675 | 1.100604 | 4.844668 | 0.001986 | 0.016424 |
| ZNF853 | ENSG00000236609 | 1.348659 | 1.162064 | 0.001993 | 0.016466 |
| QPRT | ENSG00000103485 | 1.102059 | 4.59961 | 0.001993 | 0.016466 |
| ALKBH2 | ENSG00000189046 | 1.145913 | 3.02066 | 0.002003 | 0.016534 |
| NKD2 | ENSG00000145506 | 1.212663 | 1.730387 | 0.002023 | 0.016695 |
| S100Z | ENSG00000171643 | 1.126199 | 3.550591 | 0.002079 | 0.017117 |
| SIMC1 | ENSG00000170085 | 1.135669 | 3.460134 | 0.0021 | 0.017262 |
| STPG1 | ENSG00000001460 | 1.163418 | 2.263644 | 0.002115 | 0.017374 |
| ITPRIPL1 | ENSG00000198885 | 1.173433 | 2.395475 | 0.002118 | 0.017391 |
| DAPK1 | ENSG00000196730 | 1.078875 | 7.184698 | 0.002119 | 0.017391 |
| LRRCC1 | ENSG00000133739 | 1.129028 | 3.480674 | 0.002167 | 0.017753 |
| EGR3 | ENSG00000179388 | 1.099798 | 4.313094 | 0.002176 | 0.017812 |
| REXO4 | ENSG00000148300 | 1.101219 | 4.174734 | 0.002185 | 0.01786 |
| SEPT6 | ENSG00000125354 | 1.072199 | 8.441435 | 0.002216 | 0.018037 |
| TTLL7 | ENSG00000137941 | 1.312674 | 1.381254 | 0.002233 | 0.018139 |
| CLGN | ENSG00000153132 | 1.272139 | 1.299319 | 0.002233 | 0.018139 |
| KDM6B | ENSG00000132510 | 1.070956 | 8.375833 | 0.00224 | 0.018183 |
| ALDH2 | ENSG00000111275 | 1.101887 | 4.183292 | 0.00226 | 0.018306 |
| TRMT13 | ENSG00000122435 | 1.116825 | 3.576136 | 0.002263 | 0.018309 |
| PPFIBP1 | ENSG00000110841 | 1.079177 | 5.361038 | 0.002264 | 0.018309 |
| RILPL1 | ENSG00000188026 | 1.216908 | 1.890995 | 0.002306 | 0.018612 |
| BDP1 | ENSG00000145734 | 1.068511 | 7.739921 | 0.002313 | 0.018663 |
| HAS3 | ENSG00000103044 | 1.254567 | 1.146214 | 0.002318 | 0.018692 |
| LYRM5 | ENSG00000205707 | 1.101464 | 3.784177 | 0.002403 | 0.019297 |
| NUDT16 | ENSG00000198585 | 1.073502 | 5.203373 | 0.002439 | 0.019562 |
| TNF | ENSG00000232810 | 1.073528 | 5.06382 | 0.002478 | 0.019767 |
| RET | ENSG00000165731 | 1.188242 | 2.126038 | 0.002479 | 0.019767 |
| WBP4 | ENSG00000120688 | 1.089857 | 3.983974 | 0.002497 | 0.019841 |
| RILPL2 | ENSG00000150977 | 1.062264 | 6.482353 | 0.002518 | 0.019982 |
| ZNF441 | ENSG00000197044 | 1.102256 | 3.202664 | 0.002521 | 0.020002 |
| TRH | ENSG00000170893 | 1.068331 | 5.396546 | 0.002573 | 0.020368 |
| PRLR | ENSG00000113494 | 1.159334 | 2.254281 | 0.002593 | 0.02049 |
| ZBTB12 | ENSG00000204366 | 1.128711 | 2.691701 | 0.002611 | 0.020612 |
| IZUMO4 | ENSG00000099840 | 1.202862 | 1.615382 | 0.002647 | 0.020892 |
| ZNF141 | ENSG00000131127 | 1.068614 | 4.964731 | 0.002651 | 0.020909 |
| TCHP | ENSG00000139437 | 1.063832 | 5.169807 | 0.002655 | 0.020909 |
| IL12A | ENSG00000168811 | 1.166051 | 1.891679 | 0.002668 | 0.020972 |
| GAMT | ENSG00000130005 | 1.151612 | 2.260536 | 0.002714 | 0.021281 |
| C6orf164 | ENSG00000203871 | 1.255064 | 1.334864 | 0.00273 | 0.021381 |
| GIMAP7 | ENSG00000179144 | 1.132878 | 2.577346 | 0.002741 | 0.02145 |
| SLC4A7 | ENSG00000033867 | 1.05767 | 5.329779 | 0.002765 | 0.021621 |
| CENPJ | ENSG00000151849 | 1.051055 | 6.402753 | 0.00279 | 0.021767 |
| LTK | ENSG00000062524 | 1.052865 | 5.506119 | 0.002817 | 0.021956 |
| KTN1 | ENSG00000126777 | 1.04732 | 7.298658 | 0.002833 | 0.02206 |
| GBP3 | ENSG00000117226 | 1.068971 | 4.452748 | 0.002847 | 0.022135 |
| HTATSF1 | ENSG00000102241 | 1.048415 | 6.663654 | 0.002848 | 0.022136 |
| PPP1R9A | ENSG00000158528 | 1.075235 | 3.96929 | 0.002853 | 0.022165 |
| GSTM1 | ENSG00000134184 | 1.120349 | 2.622369 | 0.002884 | 0.022377 |
| PPP1R32 | ENSG00000162148 | 1.202973 | 1.74537 | 0.002899 | 0.022478 |
| NUDT13 | ENSG00000166321 | 1.298962 | 1.203747 | 0.002905 | 0.022512 |
| AGAP10 | ENSG00000204172 | 1.245017 | 1.426462 | 0.002921 | 0.022627 |
| CDC37 | ENSG00000105401 | 1.040809 | 7.479356 | 0.002993 | 0.023143 |
| CNKSR2 | ENSG00000149970 | 1.192571 | 1.952535 | 0.003036 | 0.023419 |
| BTLA | ENSG00000186265 | 1.16227 | 1.896409 | 0.003072 | 0.023688 |
| 1-Mar | ENSG00000145416 | 1.059173 | 4.424603 | 0.003077 | 0.023717 |
| AFF3 | ENSG00000144218 | 1.04334 | 5.775434 | 0.003117 | 0.023998 |
| ZSWIM6 | ENSG00000130449 | 1.050575 | 4.944657 | 0.003126 | 0.024054 |
| PAF1 | ENSG00000006712 | 1.041073 | 5.992413 | 0.003127 | 0.024054 |
| L3MBTL4 | ENSG00000154655 | 1.084346 | 3.837023 | 0.003139 | 0.024131 |
| KIAA1407 | ENSG00000163617 | 1.062513 | 4.039784 | 0.003142 | 0.02414 |
| GPATCH2 | ENSG00000092978 | 1.057467 | 4.284994 | 0.003165 | 0.024272 |
| PPP1R3E | ENSG00000235194 | 1.050247 | 4.796904 | 0.003216 | 0.024616 |
| HAPLN3 | ENSG00000140511 | 1.174572 | 1.517818 | 0.003239 | 0.024754 |
| TCF7L2 | ENSG00000148737 | 1.041508 | 5.138561 | 0.003287 | 0.025096 |
| GPX7 | ENSG00000116157 | 1.08798 | 3.15269 | 0.003351 | 0.025437 |
| RTKN2 | ENSG00000182010 | 1.31507 | 1.081792 | 0.003364 | 0.025509 |
| ARGLU1 | ENSG00000134884 | 1.027897 | 7.089417 | 0.003388 | 0.025591 |
| TRPT1 | ENSG00000149743 | 1.100967 | 2.602191 | 0.003391 | 0.025591 |
| LRRC63 | ENSG00000173988 | 1.240305 | 1.079869 | 0.003391 | 0.025591 |
| SVIL | ENSG00000197321 | 1.027491 | 6.777128 | 0.003432 | 0.025591 |
| GATSL3 | ENSG00000239282 | 1.216592 | 1.444791 | 0.003465 | 0.025726 |
| GIMAP1 | ENSG00000213203 | 1.09802 | 2.541381 | 0.003483 | 0.025841 |
| SPATS2L | ENSG00000196141 | 1.115757 | 2.634829 | 0.003507 | 0.025997 |
| THAP5 | ENSG00000177683 | 1.054174 | 3.898799 | 0.003524 | 0.026109 |
| IQSEC2 | ENSG00000124313 | 1.051131 | 3.826711 | 0.003559 | 0.026358 |
| FAM156B | ENSG00000179304 | 1.115569 | 2.439064 | 0.003583 | 0.026509 |
| CD72 | ENSG00000137101 | 1.147155 | 2.090644 | 0.00359 | 0.026549 |
| GLIS3 | ENSG00000107249 | 1.176422 | 1.77053 | 0.003596 | 0.026586 |
| ATP6V1E2 | ENSG00000250565 | 1.077267 | 2.620802 | 0.003671 | 0.027108 |
| S1PR5 | ENSG00000180739 | 1.111426 | 2.677992 | 0.003681 | 0.027157 |
| CMTM1 | ENSG00000089505 | 1.217989 | 1.378131 | 0.003713 | 0.027346 |
| GPALPP1 | ENSG00000133114 | 1.035495 | 4.48964 | 0.003744 | 0.027546 |
| ZNF514 | ENSG00000144026 | 1.027087 | 5.095688 | 0.003765 | 0.027675 |
| EPB41L2 | ENSG00000079819 | 1.030424 | 4.999264 | 0.003768 | 0.027686 |
| LMLN | ENSG00000185621 | 1.054905 | 3.506455 | 0.003774 | 0.027711 |
| RMDN2 | ENSG00000115841 | 1.291426 | 1.120408 | 0.003859 | 0.028222 |
| UBE2E2 | ENSG00000182247 | 1.04228 | 4.126527 | 0.003873 | 0.028299 |
| WWC3 | ENSG00000047644 | 1.036921 | 4.056041 | 0.003914 | 0.028584 |
| BTBD11 | ENSG00000151136 | 1.020545 | 5.17542 | 0.003925 | 0.028641 |
| C14orf79 | ENSG00000140104 | 1.134266 | 1.857695 | 0.003936 | 0.028709 |
| PPAPDC2 | ENSG00000205808 | 1.086543 | 2.773207 | 0.003993 | 0.029057 |
| ZC3HAV1L | ENSG00000146858 | 1.076128 | 2.833795 | 0.004006 | 0.029137 |
| SHD | ENSG00000105251 | 1.028935 | 4.065554 | 0.004029 | 0.029245 |
| NFATC4 | ENSG00000100968 | 1.120086 | 2.086981 | 0.004047 | 0.029311 |
| FAM228B | ENSG00000219626 | 1.017329 | 5.222651 | 0.004056 | 0.029362 |
| SAMD3 | ENSG00000164483 | 1.08647 | 2.59323 | 0.004107 | 0.029644 |
| NOLC1 | ENSG00000166197 | 1.00638 | 7.020511 | 0.004109 | 0.029644 |
| EIF3A | ENSG00000107581 | 1.00373 | 9.239174 | 0.004109 | 0.029644 |
| NR4A2 | ENSG00000153234 | 1.006068 | 7.15366 | 0.004116 | 0.029662 |
| NCL | ENSG00000115053 | 1.001316 | 9.83706 | 0.00419 | 0.030083 |
| ZNF595 | ENSG00000197701 | 1.113904 | 2.433549 | 0.004266 | 0.030558 |
| NBPF20 | ENSG00000203832 | 1.058728 | 3.031932 | 0.004282 | 0.030629 |
| SERPINI1 | ENSG00000163536 | 1.212707 | 1.164479 | 0.004321 | 0.030895 |
| RPL23A | ENSG00000198242 | 0.998412 | 8.198338 | 0.004336 | 0.030997 |
| ITGA6 | ENSG00000091409 | 1.003578 | 6.081825 | 0.004376 | 0.031247 |
| ANO5 | ENSG00000171714 | 1.233809 | 1.427607 | 0.004419 | 0.031508 |
| ZNF630 | ENSG00000221994 | 1.088367 | 2.728233 | 0.004433 | 0.031588 |
| NOP58 | ENSG00000055044 | 0.997808 | 6.502286 | 0.004487 | 0.031915 |
| NEMF | ENSG00000165525 | 0.996206 | 6.929977 | 0.004508 | 0.032036 |
| MAGEF1 | ENSG00000177383 | 1.047368 | 3.086379 | 0.004523 | 0.032129 |
| GATM | ENSG00000171766 | 1.090901 | 2.734137 | 0.004606 | 0.032659 |
| CCDC149 | ENSG00000181982 | 1.022327 | 4.167326 | 0.004618 | 0.032709 |
| LTV1 | ENSG00000135521 | 0.998056 | 5.537874 | 0.004672 | 0.033004 |
| LEO1 | ENSG00000166477 | 1.005075 | 4.769902 | 0.004675 | 0.033014 |
| ZNF619 | ENSG00000177873 | 1.014058 | 4.12448 | 0.004692 | 0.033105 |
| GPA33 | ENSG00000143167 | 1.022424 | 3.844978 | 0.004721 | 0.03329 |
| KRBA2 | ENSG00000184619 | 1.011178 | 4.335789 | 0.004731 | 0.03335 |
| RP5-1021I20.4 | ENSG00000258653 | 1.216826 | 1.096142 | 0.004769 | 0.033535 |
| C21orf49 | ENSG00000205930 | 1.185913 | 1.494639 | 0.004807 | 0.033723 |
| CENPQ | ENSG00000031691 | 1.166782 | 1.459327 | 0.004808 | 0.033723 |
| PTRF | ENSG00000177469 | 1.013103 | 4.286295 | 0.004809 | 0.033723 |
| ACSM3 | ENSG00000005187 | 0.995148 | 5.421599 | 0.004811 | 0.033723 |
| PMF1-BGLAP | ENSG00000260238 | 1.191337 | 1.258262 | 0.004812 | 0.033723 |
| ZBTB24 | ENSG00000112365 | 1.029012 | 3.603858 | 0.004814 | 0.033723 |
| AKAP9 | ENSG00000127914 | 0.98551 | 8.266425 | 0.004857 | 0.034002 |
| ZNF385D | ENSG00000151789 | 1.021297 | 3.661784 | 0.004879 | 0.034122 |
| C1orf21 | ENSG00000116667 | 1.001991 | 4.820961 | 0.004906 | 0.034299 |
| TRIM44 | ENSG00000166326 | 0.98596 | 6.93771 | 0.00493 | 0.034429 |
| ANKHD1-EIF4EBP3 | ENSG00000254996 | 0.984575 | 6.384794 | 0.005039 | 0.035068 |
| CPED1 | ENSG00000106034 | 1.120081 | 1.908781 | 0.005044 | 0.035093 |
| HMGN5 | ENSG00000198157 | 1.10595 | 2.242049 | 0.00516 | 0.035839 |
| BRD8 | ENSG00000112983 | 0.984666 | 5.774793 | 0.005162 | 0.035843 |
| ANKRD6 | ENSG00000135299 | 1.055187 | 2.665102 | 0.005168 | 0.035865 |
| SLC16A2 | ENSG00000147100 | 1.262977 | 1.673657 | 0.005186 | 0.035972 |
| RIMKLB | ENSG00000166532 | 0.985108 | 5.817939 | 0.005191 | 0.035988 |
| LAPTM4B | ENSG00000104341 | 1.008976 | 4.159545 | 0.0052 | 0.036021 |
| RPA4 | ENSG00000204086 | 1.046268 | 2.784052 | 0.005217 | 0.036109 |
| RPL11 | ENSG00000142676 | 0.976168 | 9.051673 | 0.005241 | 0.036224 |
| TBC1D16 | ENSG00000167291 | 0.99174 | 4.789909 | 0.0053 | 0.036558 |
| AC010336.1 | ENSG00000214248 | 1.192592 | 1.073446 | 0.005322 | 0.036686 |
| AP003774.4 | ENSG00000181908 | 1.219871 | 1.107458 | 0.005326 | 0.036686 |
| DBI | ENSG00000155368 | 0.99265 | 4.617003 | 0.005395 | 0.037102 |
| CEBPZ-AS1 | ENSG00000218739 | 0.982092 | 5.382924 | 0.005417 | 0.037242 |
| PUS7 | ENSG00000091127 | 0.99436 | 4.230795 | 0.005473 | 0.037613 |
| ANKRD36 | ENSG00000135976 | 0.973543 | 6.908717 | 0.005489 | 0.037695 |
| IL18RAP | ENSG00000115607 | 1.022899 | 3.055978 | 0.005498 | 0.037744 |
| PACSIN2 | ENSG00000100266 | 0.973336 | 6.576064 | 0.005545 | 0.038056 |
| CDK11A | ENSG00000008128 | 0.975721 | 5.824018 | 0.005568 | 0.038199 |
| JAM3 | ENSG00000166086 | 1.040891 | 2.94492 | 0.005595 | 0.03836 |
| APOBEC3F | ENSG00000128394 | 0.996312 | 4.112249 | 0.005656 | 0.038756 |
| GPANK1 | ENSG00000204438 | 0.97767 | 5.012103 | 0.005669 | 0.038814 |
| DDRGK1 | ENSG00000198171 | 0.976212 | 5.345295 | 0.005703 | 0.039011 |
| C10orf118 | ENSG00000165813 | 0.975771 | 5.303545 | 0.005703 | 0.039011 |
| ERG | ENSG00000157554 | 0.967256 | 6.93701 | 0.005794 | 0.039537 |
| TRAT1 | ENSG00000163519 | 1.044678 | 2.831082 | 0.005803 | 0.039586 |
| TCF7 | ENSG00000081059 | 0.998948 | 3.686143 | 0.005831 | 0.039763 |
| ZNF165 | ENSG00000197279 | 1.066973 | 2.114001 | 0.005883 | 0.040052 |
| C5orf56 | ENSG00000197536 | 0.982269 | 4.715682 | 0.005923 | 0.04022 |
| PPIG | ENSG00000138398 | 0.967464 | 6.184406 | 0.005932 | 0.040238 |
| ZCCHC17 | ENSG00000121766 | 0.986157 | 4.14158 | 0.006005 | 0.040678 |
| PLAG1 | ENSG00000181690 | 1.064211 | 2.701448 | 0.006011 | 0.040691 |
| PTPLA | ENSG00000165996 | 1.001786 | 3.286752 | 0.006038 | 0.040819 |
| IGJ | ENSG00000132465 | 0.966536 | 5.974272 | 0.006039 | 0.040819 |
| ZNF37A | ENSG00000075407 | 0.966496 | 5.823184 | 0.006047 | 0.040849 |
| ACTR3C | ENSG00000106526 | 0.990739 | 3.649577 | 0.006072 | 0.04096 |
| ZNF234 | ENSG00000263002 | 1.001954 | 3.302645 | 0.006077 | 0.04096 |
| KDELC1 | ENSG00000134901 | 1.046759 | 2.389259 | 0.006086 | 0.040966 |
| CNTLN | ENSG00000044459 | 0.975351 | 4.836048 | 0.006089 | 0.040973 |
| YBX1 | ENSG00000065978 | 0.959442 | 7.729472 | 0.00612 | 0.040977 |
| FMO5 | ENSG00000131781 | 0.993089 | 3.536113 | 0.006128 | 0.040977 |
| ZNF589 | ENSG00000164048 | 0.971119 | 4.952034 | 0.006203 | 0.041083 |
| CHMP6 | ENSG00000176108 | 0.972034 | 4.805019 | 0.00622 | 0.041121 |
| RP11-343C2.11 | ENSG00000260914 | 1.044079 | 2.752895 | 0.00622 | 0.041121 |
| EIF2B2 | ENSG00000119718 | 0.975692 | 4.22172 | 0.006274 | 0.041448 |
| ZCCHC9 | ENSG00000131732 | 0.987446 | 3.689494 | 0.006344 | 0.041841 |
| S100PBP | ENSG00000116497 | 0.959753 | 5.918045 | 0.006392 | 0.042133 |
| PRDM16 | ENSG00000142611 | 0.962333 | 5.570085 | 0.006406 | 0.042194 |
| BTN2A2 | ENSG00000124508 | 0.974718 | 4.351606 | 0.006416 | 0.042248 |
| THAP8 | ENSG00000161277 | 1.174686 | 1.081449 | 0.006454 | 0.042444 |
| PDLIM1 | ENSG00000107438 | 0.956282 | 6.029582 | 0.00654 | 0.042968 |
| AL356356.1 | ENSG00000225996 | 1.112525 | 1.785276 | 0.006651 | 0.043549 |
| ARHGAP12 | ENSG00000165322 | 0.959807 | 5.181379 | 0.006684 | 0.043745 |
| SOGA2 | ENSG00000168502 | 0.971565 | 3.934107 | 0.006706 | 0.04386 |
| RPGR | ENSG00000156313 | 0.958383 | 5.174255 | 0.006706 | 0.04386 |
| HRH2 | ENSG00000113749 | 1.028617 | 2.787984 | 0.006755 | 0.043996 |
| VNN1 | ENSG00000112299 | 0.994344 | 3.569692 | 0.006768 | 0.044032 |
| NDUFB10 | ENSG00000140990 | 0.976938 | 3.861126 | 0.006769 | 0.044032 |
| ZCCHC6 | ENSG00000083223 | 0.948726 | 6.82493 | 0.006787 | 0.044135 |
| NKX2-3 | ENSG00000119919 | 0.993342 | 3.293556 | 0.006805 | 0.044236 |
| RPP25 | ENSG00000178718 | 1.141448 | 1.352546 | 0.006828 | 0.04437 |
| SYTL4 | ENSG00000102362 | 0.982846 | 3.940973 | 0.00688 | 0.044634 |
| SAFB2 | ENSG00000130254 | 0.945447 | 7.631897 | 0.006881 | 0.044634 |
| APOL2 | ENSG00000128335 | 0.961278 | 4.660489 | 0.006918 | 0.044805 |
| NBEA | ENSG00000172915 | 0.982544 | 4.001755 | 0.006919 | 0.044805 |
| TMEM106A | ENSG00000184988 | 0.948969 | 6.249431 | 0.006931 | 0.044851 |
| TAF15 | ENSG00000172660 | 0.945091 | 7.431466 | 0.006947 | 0.044893 |
| DET1 | ENSG00000140543 | 1.034692 | 2.363023 | 0.006998 | 0.045156 |
| SART1 | ENSG00000175467 | 0.943398 | 7.364687 | 0.007045 | 0.045435 |
| ELK3 | ENSG00000111145 | 0.949102 | 5.635098 | 0.007061 | 0.045524 |
| HOXA9 | ENSG00000078399 | 0.944341 | 6.264024 | 0.007145 | 0.045876 |
| CCDC169 | ENSG00000242715 | 1.184397 | 1.167809 | 0.007161 | 0.045934 |
| NHSL2 | ENSG00000204131 | 0.952223 | 5.160037 | 0.007172 | 0.045989 |
| IL4I1 | ENSG00000104951 | 1.079019 | 1.599546 | 0.007209 | 0.04621 |
| DNAJC18 | ENSG00000170464 | 1.005971 | 2.713849 | 0.007243 | 0.046395 |
| ZNF296 | ENSG00000170684 | 1.086172 | 1.58908 | 0.007281 | 0.046558 |
| C3 | ENSG00000125730 | 1.068768 | 1.559104 | 0.007319 | 0.046784 |
| UQCR11 | ENSG00000267059 | 0.971107 | 3.714953 | 0.007328 | 0.046829 |
| ZNF415 | ENSG00000170954 | 1.114414 | 1.366108 | 0.007405 | 0.047246 |
| PAXIP1-AS2 | ENSG00000214106 | 0.986517 | 2.981455 | 0.007428 | 0.047378 |
| MAMDC2 | ENSG00000165072 | 0.949716 | 4.907199 | 0.007437 | 0.047417 |
| SRPK2 | ENSG00000135250 | 0.942618 | 5.439137 | 0.007539 | 0.048008 |
| LACC1 | ENSG00000179630 | 0.979159 | 3.395883 | 0.007575 | 0.048188 |
| SRBD1 | ENSG00000068784 | 0.940789 | 5.509737 | 0.007607 | 0.048339 |
| PVRL2 | ENSG00000130202 | 0.936871 | 5.978673 | 0.007697 | 0.048844 |
| SDCCAG8 | ENSG00000054282 | 0.938344 | 5.788236 | 0.007741 | 0.049094 |
| PTMS | ENSG00000159335 | 0.939754 | 5.422663 | 0.007747 | 0.04912 |
| GJC2 | ENSG00000198835 | 1.058861 | 1.796365 | 0.007751 | 0.049131 |
| ZNF830 | ENSG00000198783 | 0.945967 | 4.601087 | 0.007779 | 0.049274 |
| POLR2J2 | ENSG00000267645 | 1.030801 | 2.813895 | 0.007801 | 0.049385 |
| ABTB2 | ENSG00000166016 | 0.935723 | 5.926468 | 0.007836 | 0.049589 |
| CEP63 | ENSG00000182923 | 0.936672 | 5.470036 | 0.007847 | 0.049645 |
| FAM229B | ENSG00000203778 | 1.108649 | 1.196282 | 0.007855 | 0.049679 |
| ZNF382 | ENSG00000161298 | 0.981669 | 2.832396 | 0.007872 | 0.049761 |
| RNPC3 | ENSG00000185946 | 0.935548 | 5.715205 | 0.007873 | 0.049761 |
| UPF3A | ENSG00000169062 | 0.938102 | 5.160066 | 0.007913 | 0.049937 |
| SH3D21 | ENSG00000214193 | 0.942401 | 4.881346 | 0.007918 | 0.049953 |
| OR52I2 | ENSG00000226288 | -3.92895 | -1.00466 | 0.000631 | 0.00622 |
| OR5AU1 | ENSG00000169327 | -3.85735 | -1.01615 | 0.0011 | 0.009976 |
| SYT1 | ENSG00000067715 | -3.81878 | -1.04066 | 0.0011 | 0.009976 |
| OR6N1 | ENSG00000197403 | -3.81878 | -1.04066 | 0.0011 | 0.009976 |
| ZIM2 | ENSG00000269699 | -3.81878 | -1.04066 | 0.0011 | 0.009976 |
| TREH | ENSG00000118094 | -3.79817 | -1.04384 | 0.0011 | 0.009976 |
| FRG2C | ENSG00000172969 | -3.79817 | -1.04384 | 0.0011 | 0.009976 |
| USP17L11 | ENSG00000233136 | -3.79817 | -1.04384 | 0.0011 | 0.009976 |
| CCDC110 | ENSG00000168491 | -3.79601 | -1.02664 | 0.001938 | 0.01612 |
| C10orf85 | ENSG00000177234 | -3.79601 | -1.02664 | 0.001938 | 0.01612 |
| ZSCAN5B | ENSG00000197213 | -3.79601 | -1.02664 | 0.001938 | 0.01612 |
| ZNF99 | ENSG00000213973 | -3.79601 | -1.02664 | 0.001938 | 0.01612 |
| DNAJC22 | ENSG00000178401 | -3.78434 | -1.04604 | 0.001938 | 0.01612 |
| HTR1B | ENSG00000135312 | -3.69985 | -1.07757 | 0.001938 | 0.01612 |
| C16orf89 | ENSG00000153446 | -3.69985 | -1.07757 | 0.001938 | 0.01612 |
| IL20 | ENSG00000162891 | -3.69634 | -1.07811 | 0.001938 | 0.01612 |
| NLRP11 | ENSG00000179873 | -3.68898 | -1.07929 | 0.001938 | 0.01612 |
| ADAD2 | ENSG00000140955 | -3.68266 | -1.08018 | 0.001938 | 0.01612 |
| DNAH3 | ENSG00000158486 | -3.68266 | -1.08018 | 0.001938 | 0.01612 |
| OVOL1 | ENSG00000172818 | -3.68266 | -1.08018 | 0.001938 | 0.01612 |
| PGK2 | ENSG00000170950 | -3.68061 | -1.08049 | 0.001938 | 0.01612 |
| NLRP13 | ENSG00000173572 | -3.68061 | -1.08049 | 0.001938 | 0.01612 |
| C5orf38 | ENSG00000186493 | -3.68061 | -1.08049 | 0.001938 | 0.01612 |
| AP000889.3 | ENSG00000268467 | -3.68061 | -1.08049 | 0.001938 | 0.01612 |
| PCSK2 | ENSG00000125851 | -3.69332 | -1.06104 | 0.003445 | 0.025591 |
| NPR1 | ENSG00000169418 | -3.69332 | -1.06104 | 0.003445 | 0.025591 |
| OLFM3 | ENSG00000118733 | -3.63789 | -1.08718 | 0.003445 | 0.025591 |
| SCGB3A2 | ENSG00000164265 | -3.63789 | -1.08718 | 0.003445 | 0.025591 |
| SLC47A2 | ENSG00000180638 | -3.63789 | -1.08718 | 0.003445 | 0.025591 |
| HOGA1 | ENSG00000241935 | -3.63789 | -1.08718 | 0.003445 | 0.025591 |
| CTC-398G3.6 | ENSG00000267477 | -3.63789 | -1.08718 | 0.003445 | 0.025591 |
| WDR16 | ENSG00000166596 | -3.63658 | -1.08739 | 0.003445 | 0.025591 |
| ERBB4 | ENSG00000178568 | -3.61765 | -1.09058 | 0.003445 | 0.025591 |
| AC018470.1 | ENSG00000268241 | -3.59079 | -1.11249 | 0.003445 | 0.025591 |
| FMO6P | ENSG00000117507 | -3.57065 | -1.11544 | 0.003445 | 0.025591 |
| EDN3 | ENSG00000124205 | -3.57065 | -1.11544 | 0.003445 | 0.025591 |
| TAS2R8 | ENSG00000121314 | -3.55473 | -1.11781 | 0.003445 | 0.025591 |
| CXCL13 | ENSG00000156234 | -3.55473 | -1.11781 | 0.003445 | 0.025591 |
| APOF | ENSG00000175336 | -3.55473 | -1.11781 | 0.003445 | 0.025591 |
| FBXO27 | ENSG00000161243 | -3.59536 | -1.09415 | 0.006185 | 0.040977 |
| GGN | ENSG00000179168 | -3.58221 | -1.09633 | 0.006185 | 0.040977 |
| CADM2 | ENSG00000175161 | -3.52637 | -1.1223 | 0.006185 | 0.040977 |
| DLX5 | ENSG00000105880 | -3.44764 | -1.15164 | 0.006185 | 0.040977 |
| C9orf84 | ENSG00000165181 | -3.42921 | -1.15431 | 0.006185 | 0.040977 |
| G6PC | ENSG00000131482 | -3.41462 | -1.15645 | 0.006185 | 0.040977 |
| KLK6 | ENSG00000167755 | -3.41462 | -1.15645 | 0.006185 | 0.040977 |
| OR2AT4 | ENSG00000171561 | -3.41462 | -1.15645 | 0.006185 | 0.040977 |
| FAM110D | ENSG00000197245 | -3.41462 | -1.15645 | 0.006185 | 0.040977 |
| RD3L | ENSG00000227729 | -3.41462 | -1.15645 | 0.006185 | 0.040977 |
| AC073610.5 | ENSG00000255863 | -3.41462 | -1.15645 | 0.006185 | 0.040977 |
| CLCA4 | ENSG00000016602 | -3.41288 | -1.15671 | 0.006185 | 0.040977 |
| TLX1 | ENSG00000107807 | -3.41288 | -1.15671 | 0.006185 | 0.040977 |
| OVOL2 | ENSG00000125850 | -3.41288 | -1.15671 | 0.006185 | 0.040977 |
| LRRIQ1 | ENSG00000133640 | -3.41288 | -1.15671 | 0.006185 | 0.040977 |
| EMX1 | ENSG00000135638 | -3.41288 | -1.15671 | 0.006185 | 0.040977 |
| KRT20 | ENSG00000171431 | -3.41288 | -1.15671 | 0.006185 | 0.040977 |
| OTOL1 | ENSG00000182447 | -3.41288 | -1.15671 | 0.006185 | 0.040977 |
| BMP7 | ENSG00000101144 | -2.67172 | -1.00103 | 0.006736 | 0.043888 |
| DUSP26 | ENSG00000133878 | -2.65048 | -1.00795 | 0.006736 | 0.043888 |
| ZNF556 | ENSG00000172000 | -2.64561 | -1.00425 | 0.006736 | 0.043888 |

FDR-correlation P <0.05 was regarded as significantly different expression, genes with Log2 FC>0 and Log2 CPM >1 were assigned to upregulated genes, and genes with Log2FC<0 and Log2 CPM<-1 were assigned to downregulated genes.

Table S12. Gene enrichment analysis of the differentially expressed genes

| Category | ID | Names | P value |
| --- | --- | --- | --- |
| KEGG Pathway | 117293 | Arrhythmogenic right ventricular cardiomyopathy (ARVC) | 7.52E-05 |
| KEGG Pathway | 83061 | Wnt signaling pathway | 1.75E-03 |
| KEGG Pathway | 194384 | African trypanosomiasis | 4.35E-03 |
| KEGG Pathway | 82957 | Arginine and proline metabolism | 4.45E-03 |
| KEGG Pathway | 83051 | Cytokine-cytokine receptor interaction | 1.08E-02 |
| KEGG Pathway | 83112 | Thyroid cancer | 1.20E-02 |
| KEGG Pathway | 413351 | Urea cycle | 1.34E-02 |
| KEGG Pathway | 413359 | Creatine pathway | 1.84E-02 |
| KEGG Pathway | 121494 | Dilated cardiomyopathy | 2.24E-02 |
| KEGG Pathway | 83036 | Ribosome | 2.27E-02 |
| KEGG Pathway | 469200 | Legionellosis | 2.82E-02 |
| KEGG Pathway | 83113 | Basal cell carcinoma | 2.82E-02 |
| KEGG Pathway | 93344 | Cardiac muscle contraction | 3.49E-02 |
| KEGG Pathway | 147809 | Chagas disease (American trypanosomiasis) | 4.05E-02 |
| KEGG Pathway | 1272485 | Aldosterone synthesis and secretion | 4.30E-02 |
| KEGG Pathway | 114229 | Hypertrophic cardiomyopathy (HCM) | 4.52E-02 |
| KEGG Pathway | 83065 | Axon guidance | 4.81E-02 |
| GO: Molecular Function | GO:0000981 | DNA-binding transcription factor activity, RNA polymerase II-specific | 2.36E-07 |
| GO: Molecular Function | GO:0001217 | DNA-binding transcription repressor activity | 2.77E-04 |
| GO: Molecular Function | GO:0001227 | DNA-binding transcription repressor activity, RNA polymerase II-specific | 2.77E-04 |
| GO: Molecular Function | GO:0043565 | sequence-specific DNA binding | 4.88E-04 |
| GO: Molecular Function | GO:0004873 | asialoglycoprotein receptor activity | 1.22E-03 |
| GO: Molecular Function | GO:0071820 | N-box binding | 1.22E-03 |
| GO: Molecular Function | GO:0038132 | neuregulin binding | 1.27E-03 |
| GO: Molecular Function | GO:0044212 | transcription regulatory region DNA binding | 1.32E-03 |
| GO: Molecular Function | GO:0050145 | nucleoside monophosphate kinase activity | 1.40E-03 |
| GO: Molecular Function | GO:0001067 | regulatory region nucleic acid binding | 1.47E-03 |
| GO: Molecular Function | GO:0000976 | transcription regulatory region sequence-specific DNA binding | 1.61E-03 |
| GO: Molecular Function | GO:0000977 | RNA polymerase II regulatory region sequence-specific DNA binding | 1.68E-03 |
| GO: Molecular Function | GO:0001012 | RNA polymerase II regulatory region DNA binding | 2.02E-03 |
| GO: Molecular Function | GO:0000978 | RNA polymerase II cis-regulatory region sequence-specific DNA binding | 3.26E-03 |
| GO: Molecular Function | GO:0051959 | dynein light intermediate chain binding | 3.27E-03 |
| GO: Molecular Function | GO:0070740 | tubulin-glutamic acid ligase activity | 3.45E-03 |
| GO: Molecular Function | GO:0004647 | phosphoserine phosphatase activity | 3.45E-03 |
| GO: Molecular Function | GO:1990837 | sequence-specific double-stranded DNA binding | 4.61E-03 |
| GO: Molecular Function | GO:0000987 | cis-regulatory region sequence-specific DNA binding | 4.67E-03 |
| GO: Molecular Function | GO:1990939 | ATP-dependent microtubule motor activity | 5.17E-03 |
| GO: Molecular Function | GO:0004385 | guanylate kinase activity | 5.39E-03 |
| GO: Molecular Function | GO:0003690 | double-stranded DNA binding | 7.57E-03 |
| GO: Molecular Function | GO:0005248 | voltage-gated sodium channel activity | 8.44E-03 |
| GO: Molecular Function | GO:0086083 | cell adhesive protein binding involved in bundle of His cell-Purkinje myocyte communication | 1.10E-02 |
| GO: Molecular Function | GO:0070739 | protein-glutamic acid ligase activity | 1.10E-02 |
| GO: Molecular Function | GO:0030515 | snoRNA binding | 1.13E-02 |
| GO: Molecular Function | GO:0008574 | ATP-dependent microtubule motor activity, plus-end-directed | 1.29E-02 |
| GO: Molecular Function | GO:0003777 | microtubule motor activity | 1.37E-02 |
| GO: Molecular Function | GO:0016776 | phosphotransferase activity, phosphate group as acceptor | 1.40E-02 |
| GO: Molecular Function | GO:0008510 | sodium:bicarbonate symporter activity | 1.61E-02 |
| GO: Molecular Function | GO:0001162 | RNA polymerase II intronic transcription regulatory region sequence-specific DNA binding | 1.61E-02 |
| GO: Molecular Function | GO:0046625 | sphingolipid binding | 1.65E-02 |
| GO: Molecular Function | GO:0070063 | RNA polymerase binding | 1.86E-02 |
| GO: Molecular Function | GO:0019205 | nucleobase-containing compound kinase activity | 2.19E-02 |
| GO: Molecular Function | GO:1990756 | protein binding, bridging involved in substrate recognition for ubiquitination | 2.20E-02 |
| GO: Molecular Function | GO:0098631 | cell adhesion mediator activity | 2.38E-02 |
| GO: Molecular Function | GO:0008331 | high voltage-gated calcium channel activity | 2.55E-02 |
| GO: Molecular Function | GO:0001161 | intronic transcription regulatory region sequence-specific DNA binding | 2.59E-02 |
| GO: Molecular Function | GO:0044213 | intronic transcription regulatory region DNA binding | 2.97E-02 |
| GO: Molecular Function | GO:0003743 | translation initiation factor activity | 3.00E-02 |
| GO: Molecular Function | GO:0003774 | motor activity | 3.03E-02 |
| GO: Molecular Function | GO:0035259 | glucocorticoid receptor binding | 3.37E-02 |
| GO: Molecular Function | GO:0001091 | RNA polymerase II general transcription initiation factor binding | 3.37E-02 |
| GO: Molecular Function | GO:0042163 | interleukin-12 beta subunit binding | 3.56E-02 |
| GO: Molecular Function | GO:0031715 | C5L2 anaphylatoxin chemotactic receptor binding | 3.56E-02 |
| GO: Molecular Function | GO:0004753 | saccharopine dehydrogenase activity | 3.56E-02 |
| GO: Molecular Function | GO:0004055 | argininosuccinate synthase activity | 3.56E-02 |
| GO: Molecular Function | GO:1990238 | double-stranded DNA endodeoxyribonuclease activity | 3.56E-02 |
| GO: Molecular Function | GO:0062065 | box H/ACA snoRNP complex binding | 3.56E-02 |
| GO: Molecular Function | GO:0008115 | sarcosine oxidase activity | 3.56E-02 |
| GO: Molecular Function | GO:0001631 | cysteinyl leukotriene receptor activity | 3.56E-02 |
| GO: Molecular Function | GO:0015067 | amidinotransferase activity | 3.56E-02 |
| GO: Molecular Function | GO:0004555 | alpha,alpha-trehalase activity | 3.56E-02 |
| GO: Molecular Function | GO:0015927 | trehalase activity | 3.56E-02 |
| GO: Molecular Function | GO:0047130 | saccharopine dehydrogenase (NADP+, L-lysine-forming) activity | 3.56E-02 |
| GO: Molecular Function | GO:0050031 | L-pipecolate oxidase activity | 3.56E-02 |
| GO: Molecular Function | GO:0072591 | citrate-L-glutamate ligase activity | 3.56E-02 |
| GO: Molecular Function | GO:0031714 | C5a anaphylatoxin chemotactic receptor binding | 3.56E-02 |
| GO: Molecular Function | GO:0008700 | 4-hydroxy-2-oxoglutarate aldolase activity | 3.56E-02 |
| GO: Molecular Function | GO:0031870 | thromboxane A2 receptor binding | 3.56E-02 |
| GO: Molecular Function | GO:0000016 | lactase activity | 3.56E-02 |
| GO: Molecular Function | GO:1901641 | ITP binding | 3.56E-02 |
| GO: Molecular Function | GO:1990631 | ErbB-4 class receptor binding | 3.56E-02 |
| GO: Molecular Function | GO:0004925 | prolactin receptor activity | 3.56E-02 |
| GO: Molecular Function | GO:0001648 | proteinase activated receptor activity | 3.56E-02 |
| GO: Molecular Function | GO:0008437 | thyrotropin-releasing hormone activity | 3.56E-02 |
| GO: Molecular Function | GO:0001716 | L-amino-acid oxidase activity | 3.56E-02 |
| GO: Molecular Function | GO:0047131 | saccharopine dehydrogenase (NAD+, L-glutamate-forming) activity | 3.56E-02 |
| GO: Molecular Function | GO:0045517 | interleukin-20 receptor binding | 3.56E-02 |
| GO: Molecular Function | GO:0008811 | chloramphenicol O-acetyltransferase activity | 3.56E-02 |
| GO: Molecular Function | GO:0062064 | box C/D snoRNP complex binding | 3.56E-02 |
| GO: Molecular Function | GO:0031724 | CXCR5 chemokine receptor binding | 3.56E-02 |
| GO: Molecular Function | GO:0015068 | glycine amidinotransferase activity | 3.56E-02 |
| GO: Molecular Function | GO:0030731 | guanidinoacetate N-methyltransferase activity | 3.56E-02 |
| GO: Molecular Function | GO:1901640 | XTP binding | 3.56E-02 |
| GO: Molecular Function | GO:0070697 | activin receptor binding | 3.60E-02 |
| GO: Molecular Function | GO:0071936 | coreceptor activity involved in Wnt signaling pathway | 3.60E-02 |
| GO: Molecular Function | GO:0019975 | interleukin-17 binding | 3.60E-02 |
| GO: Molecular Function | GO:0042802 | identical protein binding | 3.64E-02 |
| GO: Molecular Function | GO:0008013 | beta-catenin binding | 3.68E-02 |
| GO: Molecular Function | GO:0070696 | transmembrane receptor protein serine/threonine kinase binding | 3.81E-02 |
| GO: Molecular Function | GO:0001099 | basal RNA polymerase II transcription machinery binding | 3.91E-02 |
| GO: Molecular Function | GO:0001098 | basal transcription machinery binding | 3.91E-02 |
| GO: Molecular Function | GO:0098632 | cell-cell adhesion mediator activity | 4.25E-02 |
| GO: Molecular Function | GO:0001223 | transcription coactivator binding | 4.27E-02 |
| GO: Molecular Function | GO:0005031 | tumor necrosis factor-activated receptor activity | 4.40E-02 |
| GO: Molecular Function | GO:0070016 | armadillo repeat domain binding | 4.40E-02 |
| GO: Molecular Function | GO:0031402 | sodium ion binding | 4.40E-02 |
| GO: Molecular Function | GO:0031369 | translation initiation factor binding | 4.73E-02 |

Table S13. KEGG pathways and GO enrichment analyses in the 84 hub genes.

| ID | Name | P value |
| --- | --- | --- |
| KEGG_83015 | Pantothenate and CoA biosynthesis | 2.51E-03 |
| KEGG_83063 | Hedgehog signaling pathway | 1.64E-02 |
| KEGG_82954 | Valine, leucine and isoleucine biosynthesis | 1.66E-02 |
| KEGG_102279 | Endocytosis | 2.30E-02 |
| KEGG_83081 | B cell receptor signaling pathway | 3.54E-02 |
| KEGG_117293 | Arrhythmogenic right ventricular cardiomyopathy (ARVC) | 3.63E-02 |
| KEGG_83065 | Axon guidance | 3.65E-02 |
| KEGG_413352 | Lysine degradation, lysine => saccharopine => acetoacetyl-CoA | 4.50E-02 |
| KEGG_83068 | ECM-receptor interaction | 4.60E-02 |
| KEGG_83088 | Taste transduction | 4.70E-02 |
| GO:0051015 | actin filament binding | 3.69E-05 |
| GO:0051020 | GTPase binding | 1.67E-04 |
| GO:0003779 | actin binding | 6.70E-04 |
| GO:0004385 | guanylate kinase activity | 9.62E-04 |
| GO:0008092 | cytoskeletal protein binding | 1.73E-03 |
| GO:0000977 | RNA polymerase II regulatory region sequence-specific DNA binding | 3.31E-03 |
| GO:0001012 | RNA polymerase II regulatory region DNA binding | 3.54E-03 |
| GO:0008022 | protein C-terminus binding | 3.95E-03 |
| GO:0004753 | saccharopine dehydrogenase activity | 4.28E-03 |
| GO:0004925 | prolactin receptor activity | 4.28E-03 |
| GO:0047130 | saccharopine dehydrogenase (NADP+, L-lysine-forming) activity | 4.28E-03 |
| GO:0047131 | saccharopine dehydrogenase (NAD+, L-glutamate-forming) activity | 4.28E-03 |
| GO:0005248 | voltage-gated sodium channel activity | 4.66E-03 |
| GO:0044877 | protein-containing complex binding | 4.69E-03 |
| GO:0043565 | sequence-specific DNA binding | 4.69E-03 |
| GO:0000976 | transcription regulatory region sequence-specific DNA binding | 4.76E-03 |
| GO:0003690 | double-stranded DNA binding | 4.79E-03 |
| GO:0050145 | nucleoside monophosphate kinase activity | 5.05E-03 |
| GO:0001217 | DNA-binding transcription repressor activity | 5.16E-03 |
| GO:0001227 | DNA-binding transcription repressor activity, RNA polymerase II-specific | 5.16E-03 |
| GO:0008574 | ATP-dependent microtubule motor activity, plus-end-directed | 5.88E-03 |
| GO:0032452 | histone demethylase activity | 6.76E-03 |
| GO:1990837 | sequence-specific double-stranded DNA binding | 6.90E-03 |
| GO:0098680 | template-free RNA nucleotidyltransferase | 8.51E-03 |
| GO:0004455 | ketol-acid reductoisomerase activity | 8.51E-03 |
| GO:0052655 | L-valine transaminase activity | 8.51E-03 |
| GO:0004084 | branched-chain-amino-acid transaminase activity | 8.51E-03 |
| GO:0003984 | acetolactate synthase activity | 8.51E-03 |
| GO:0052656 | L-isoleucine transaminase activity | 8.51E-03 |
| GO:1903981 | enterobactin binding | 8.51E-03 |
| GO:0030617 | transforming growth factor beta receptor, inhibitory cytoplasmic mediator activity | 8.51E-03 |
| GO:0004160 | dihydroxy-acid dehydratase activity | 8.51E-03 |
| GO:0052654 | L-leucine transaminase activity | 8.51E-03 |
| GO:0051087 | chaperone binding | 1.16E-02 |
| GO:0000978 | RNA polymerase II cis-regulatory region sequence-specific DNA binding | 1.18E-02 |
| GO:0032451 | demethylase activity | 1.20E-02 |
| GO:0044212 | transcription regulatory region DNA binding | 1.21E-02 |
| GO:0001067 | regulatory region nucleic acid binding | 1.25E-02 |
| GO:0004683 | calmodulin-dependent protein kinase activity | 1.26E-02 |
| GO:0070698 | type I activin receptor binding | 1.27E-02 |
| GO:0017159 | pantetheine hydrolase activity | 1.27E-02 |
| GO:0042978 | ornithine decarboxylase activator activity | 1.27E-02 |
| GO:0000987 | cis-regulatory region sequence-specific DNA binding | 1.33E-02 |
| GO:0016776 | phosphotransferase activity, phosphate group as acceptor | 1.38E-02 |
| GO:0048156 | tau protein binding | 1.65E-02 |
| GO:0003968 | RNA-directed 5'-3' RNA polymerase activity | 1.69E-02 |
| GO:1990939 | ATP-dependent microtubule motor activity | 1.71E-02 |
| GO:0019205 | nucleobase-containing compound kinase activity | 1.71E-02 |
| GO:0042802 | identical protein binding | 2.05E-02 |
| GO:0098639 | collagen binding involved in cell-matrix adhesion | 2.11E-02 |
| GO:0003721 | telomerase RNA reverse transcriptase activity | 2.11E-02 |
| GO:0071558 | histone demethylase activity (H3-K27 specific) | 2.11E-02 |
| GO:0086083 | cell adhesive protein binding involved in bundle of His cell-Purkinje myocyte communication | 2.11E-02 |
| GO:0004672 | protein kinase activity | 2.34E-02 |
| GO:0050839 | cell adhesion molecule binding | 2.46E-02 |
| GO:0003774 | motor activity | 2.50E-02 |
| GO:0031749 | D2 dopamine receptor binding | 2.53E-02 |
| GO:0120170 | intraciliary transport particle B binding | 2.53E-02 |
| GO:0008510 | sodium:bicarbonate symporter activity | 2.53E-02 |
| GO:0038064 | collagen receptor activity | 2.53E-02 |
| GO:0042979 | ornithine decarboxylase regulator activity | 2.53E-02 |
| GO:0005525 | GTP binding | 2.73E-02 |
| GO:0004586 | ornithine decarboxylase activity | 2.95E-02 |
| GO:0098634 | cell-matrix adhesion mediator activity | 2.95E-02 |
| GO:0050840 | extracellular matrix binding | 2.96E-02 |
| GO:0098631 | cell adhesion mediator activity | 3.14E-02 |
| GO:0032550 | purine ribonucleoside binding | 3.25E-02 |
| GO:0032549 | ribonucleoside binding | 3.33E-02 |
| GO:0005113 | patched binding | 3.36E-02 |
| GO:0034235 | GPI anchor binding | 3.36E-02 |
| GO:0001883 | purine nucleoside binding | 3.36E-02 |
| GO:0008017 | microtubule binding | 3.46E-02 |
| GO:0001882 | nucleoside binding | 3.60E-02 |
| GO:0019001 | guanyl nucleotide binding | 3.76E-02 |
| GO:0032561 | guanyl ribonucleotide binding | 3.76E-02 |
| GO:0070697 | activin receptor binding | 3.77E-02 |
| GO:0004689 | phosphorylase kinase activity | 3.77E-02 |
| GO:1902944 | aspartic-type endopeptidase activity involved in amyloid precursor protein catabolic process | 3.77E-02 |
| GO:0005244 | voltage-gated ion channel activity | 4.09E-02 |
| GO:0022832 | voltage-gated channel activity | 4.14E-02 |
| GO:0016301 | kinase activity | 4.14E-02 |
| GO:0032453 | histone demethylase activity (H3-K4 specific) | 4.18E-02 |
| GO:0005072 | transforming growth factor beta receptor, cytoplasmic mediator activity | 4.18E-02 |
| GO:0016744 | transferase activity, transferring aldehyde or ketonic groups | 4.18E-02 |
| GO:0031402 | sodium ion binding | 4.18E-02 |
| GO:0016773 | phosphotransferase activity, alcohol group as acceptor | 4.22E-02 |
| GO:0140272 | exogenous protein binding | 4.27E-02 |
| GO:0001618 | virus receptor activity | 4.27E-02 |
| GO:0019900 | kinase binding | 4.34E-02 |
| GO:0017137 | Rab GTPase binding | 4.40E-02 |
| GO:0005518 | collagen binding | 4.58E-02 |
| GO:0070411 | I-SMAD binding | 4.59E-02 |
| GO:0046974 | histone methyltransferase activity (H3-K9 specific) | 4.59E-02 |
| GO:0030023 | extracellular matrix constituent conferring elasticity | 4.59E-02 |
| GO:0017016 | Ras GTPase binding | 4.63E-02 |
| GO:0060089 | molecular transducer activity | 4.63E-02 |
| GO:0005272 | sodium channel activity | 4.79E-02 |
| GO:0034713 | type I transforming growth factor beta receptor binding | 5.00E-02 |
| GO:0017154 | semaphorin receptor activity | 5.00E-02 |
| GO:0070410 | co-SMAD binding | 5.00E-02 |
| GO:0043560 | insulin receptor substrate binding | 5.00E-02 |

Table S14. Clinical characteristics of patients for the metabolomic analysis

| **Variable** | **Low** | **High** | **P value** |
| --- | --- | --- | --- |
| **Number** | **7** | **7** |  |
| **Sex, male,n(%)** | **4(57.1)** | **3(42.9)** | **1** |
| **Age,median(range),years** | **47.00[29.50,55.00]** | **54.00[45.00,62.00]** | **0.304** |
| **WBC,median(IQR),×10^9/L^1^** | **7.00[2.00,13.45]** | **2.10[1.70,11.95]** | **0.609** |
| **HB,median(IQR),g/L^2^** | **85.00[78.50,99.50]** | **81.00[73.00,85.50]** | **0.337** |
| **PLT,median(IQR),×10^9/L^3^** | **70.00[44.50,157.50]** | **98.00[62.00,126.50]** | **0.898** |
| **BMblast,median(IQR),%^4^** | **51.00[40.75,72.00]** | **57.00[28.75,65.50]** | **0.749** |
| **FABclassification,n(%)^5^** |  |  |  |
| **M0** | **1(14.3)** | **2(28.6)** | |
| **M1** | **1(14.3)** | **1(14.3)** | |
| **M2** | **2(28.6)** | **3(42.9)** | |
| **M5** | **2(28.6)** | **1(14.3)** | |
| **M6** | **1(14.3)** | **0(0.0)** | |
| **Genemutations,n(%)** |  |  |  |
| ***FLT3*-ITD** | **1(14.3)** | **1(14.3)** | **1** |
| ***NPM1*** | **1(14.3)** | **1(16.7)** | **1** |
| ***CEBPA*^DM6^** | **2(28.6)** | **0(0.0)** | **0.462** |
| ***DNMT3A*** | **2(28.6)** | **2(33.3)** | **1** |
| **IDH1** | **1(14.3)** | **0(0.0)** | |
| **IDH2** | **1(14.3)** | **3(42.9)** | |

Abbreviations: ^1^WBC, white blood cell counts; ^2^HB, hemoglobin; ^3^PLT, platelet counts; ^4^BM, bone marrow; ^5^ FAB, Franch-American-British;^6^DM, double allele. IQR, interquantile.

Table S15. Pathways enriched analysis

| Pathway Id | pathwayName | P value |
| --- | --- | --- |
| path:00400 | Phenylalanine | 1.20E-01 |
| path:00330 | Arginine and proline metabolism | 4.46E-07 |
| path:02010 | ABC transporters | 4.78E-06 |
| path:00460 | Cyanoamino acid metabolism | 5.26E-06 |
| path:00130 | Ubiquinone and other terpenoid-quinone biosynthesis | 6.05E-06 |
| path:00360 | Phenylalanine metabolism | 7.31E-06 |
| path:00860 | Porphyrin and chlorophyll metabolism | 1.23E-05 |
| path:00120 | Primary bile acid biosynthesis | 2.03E-05 |
| path:00380 | Tryptophan metabolism | 3.44E-05 |
| path:00472 | D-Arginine and D-ornithine metabolism | 9.04E-05 |
| path:00270 | Cysteine and methionine metabolism | 1.93E-04 |
| path:00600 | Sphingolipid metabolism | 4.75E-04 |
| path:00350 | Tyrosine metabolism | 7.12E-04 |
| path:00260 | Glycine | 1.52E+00 |
| path:00520 | Amino sugar and nucleotide sugar metabolism | 1.34E-03 |
| path:04974 | Protein digestion and absorption | 8.72E-03 |
| path:00290 | Valine | 1.68E-01 |
| path:05142 | Chagas disease | 1.50E-02 |
| path:00730 | Thiamine metabolism | 1.60E-02 |
| path:00430 | Taurine and hypotaurine metabolism | 2.04E-02 |
| path:00920 | Sulfur metabolism | 2.54E-02 |
| path:00500 | Starch and sucrose metabolism | 2.61E-02 |
